# Machine Learning Guided Discovery of Non‐Hemolytic Membrane Disruptive Anticancer Peptides

**DOI:** 10.1002/cmdc.202200291

**Published:** 2022-08-05

**Authors:** Elena Zakharova, Markus Orsi, Alice Capecchi, Jean‐Louis Reymond

**Affiliations:** ^1^ Department of Chemistry Biochemistry and Pharmaceutical Sciences University of Bern Freiestrasse 3 3012 Bern Switzerland

**Keywords:** anticancer peptides, chemical space, peptide design, machine learning, genetic algorithm

## Abstract

Most antimicrobial peptides (AMPs) and anticancer peptides (ACPs) fold into membrane disruptive cationic amphiphilic α‐helices, many of which are however also unpredictably hemolytic and toxic. Here we exploited the ability of recurrent neural networks (RNN) to distinguish active from inactive and non‐hemolytic from hemolytic AMPs and ACPs to discover new non‐hemolytic ACPs. Our discovery pipeline involved: 1) sequence generation using either a generative RNN or a genetic algorithm, 2) RNN classification for activity and hemolysis, 3) selection for sequence novelty, helicity and amphiphilicity, and 4) synthesis and testing. Experimental evaluation of thirty‐three peptides resulted in eleven active ACPs, four of which were non‐hemolytic, with properties resembling those of the natural ACP lasioglossin III. These experiments show the first example of direct machine learning guided discovery of non‐hemolytic ACPs.

## Graphical abstract

**Using machine learning models** trained with bioactive peptides from DBAASP, we designed new non‐hemolytic anticancer peptides (ACPs). The subsequently selected hit‐compounds **A1** and **B1** showed IC_50_ activities with low micromolar range against several cancer cell lines, having adopted amphiphilic α‐helical conformations. Further biological evaluations revealed membranolytic and mitochondria targeting properties of selected anticancer peptides.

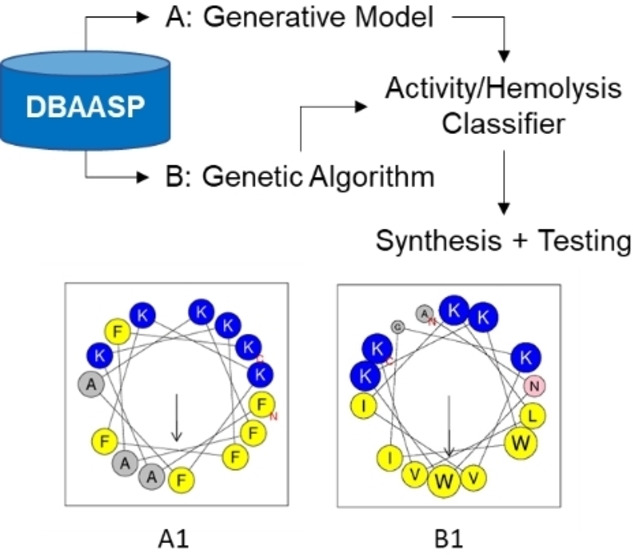

## Introduction

Machine learning (ML) is ideally suited to assist drug design whenever a large corpus of structure‐activity data is available, but the underlying structure‐activity relationships are difficult to rationalize.[^1^, ^2^, ^3^] This is the case for membrane disruptive peptides documented to act against various microorganisms, cancer cells and red blood cells.[^4^, ^5^] Indeed, while most of these peptides form cationic amphiphilic α‐helices,[^6^, ^7^] this design feature is not sufficient to predict activity, let alone the selectivity among different cells and microorganisms. Interestingly however, ML classifiers trained with sequence activity data, which is available in various open‐access databases,[^8^, ^9^, ^10^, ^11^] can distinguish active from inactive and hemolytic from non‐hemolytic peptides with good performance.[^12^, ^13^, ^14^, ^15^] Such ML classifiers have been implemented to discover new membrane disruptive peptides by selecting actives either from random sequences, or from sequences produced by generative ML models trained with activity data. Most of these ML guided peptide designs focused on antimicrobial peptides (AMPs), which is the most frequently reported activity type.[^16^, ^17^, ^18^, ^19^, ^20^]

Similar approaches have also been applied to the much less abundant data on anticancer peptides (ACPs).[^21^, ^22^, ^23^, ^24^, ^25^, ^26^, ^27^, ^28^, ^29^, ^30^, ^31^] However, only two of these provided experimental data in addition to computational validation of the method on pre‐established datasets.[^32^, ^33^] The ACPs identified with the two ML approaches were also hemolytic, and their selectivity had to be improved by iterative mutation and testing. Although both bacterial and cancer cells differ from non‐cancerous cells such as red blood cells by a higher density of negatively charged lipids on the extracellular side of their membranes,[^34^, ^35^, ^36^] the difficulty to obtain non‐hemolytic membrane disruptive ACPs by ML probably reflects the challenge to distinguish between closely related eukaryotic cells rather than between bacteria and eukaryotes, as well as the sparsity of data on ACPs to train ML models.

Herein we report two ML approaches to identify membrane disruptive ACPs supported by data from the database of antimicrobial activity and structure of peptides (DBAASP), which lists sequences and activity information on 18,405 bioactive peptides.[^8^, ^9^] In our first approach, we generated a set of tentative ACPs by sampling a generative recurrent neural network (RNN). The RNN was trained with active sequences from DBAASP, which lists peptides with various activities comprising antimicrobial and anticancer peptides, and fine‐tuned towards ACP generation by transfer learning^[37]^ with a small set of ACPs reported to be active on HeLa cells, an activity which we could easily test experimentally. In a second approach, we used our recently reported PDGA (peptide design genetic algorithm),^[38]^ which evolves random sequences towards any target molecule by rounds of mutation and selection according to a measured similarity, to generate analogs of the known ACP lasioglossin III (LL‐III), also with a reported activity on HeLa cells.^[39]^ In both approaches we filtered the generated peptides using RNN classifiers for activity and hemolysis, which were trained with DBAASP data and previously shown to have good performance compared to other methods.^[15]^ We finally selected sequences based on novelty as well as on their predicted α‐helicity and amphiphilicity to favor the expected membrane disruptive mechanism of action. As detailed below, synthesis and testing of the selected peptides allowed us to identify non‐hemolytic membrane disruptive α‐helical ACPs from both approaches.

## Results and Discussion

**ML guided design**. In our first approach, we selected 53 sequences in DBAASP which were reported to be active against HeLa cells, a common type of cancer cells which can be easily assayed. We used these 53 ACPs in a transfer learning step to fine‐tune our previously reported general RNN generative prior model,^[15]^ which had been trained with 4,774 peptides reported with any type of bioactivity in the database. We then sampled 50,000 sequences from this fine‐tuned generative model. To refine this set, we applied our previously reported RNN activity classifier trained with 6,641 active peptides from DBAASP.^[15]^ This activity classifier labeled approximately 20 % of the sampled sequences (11,458) as potentially active.

**Figure 1:**
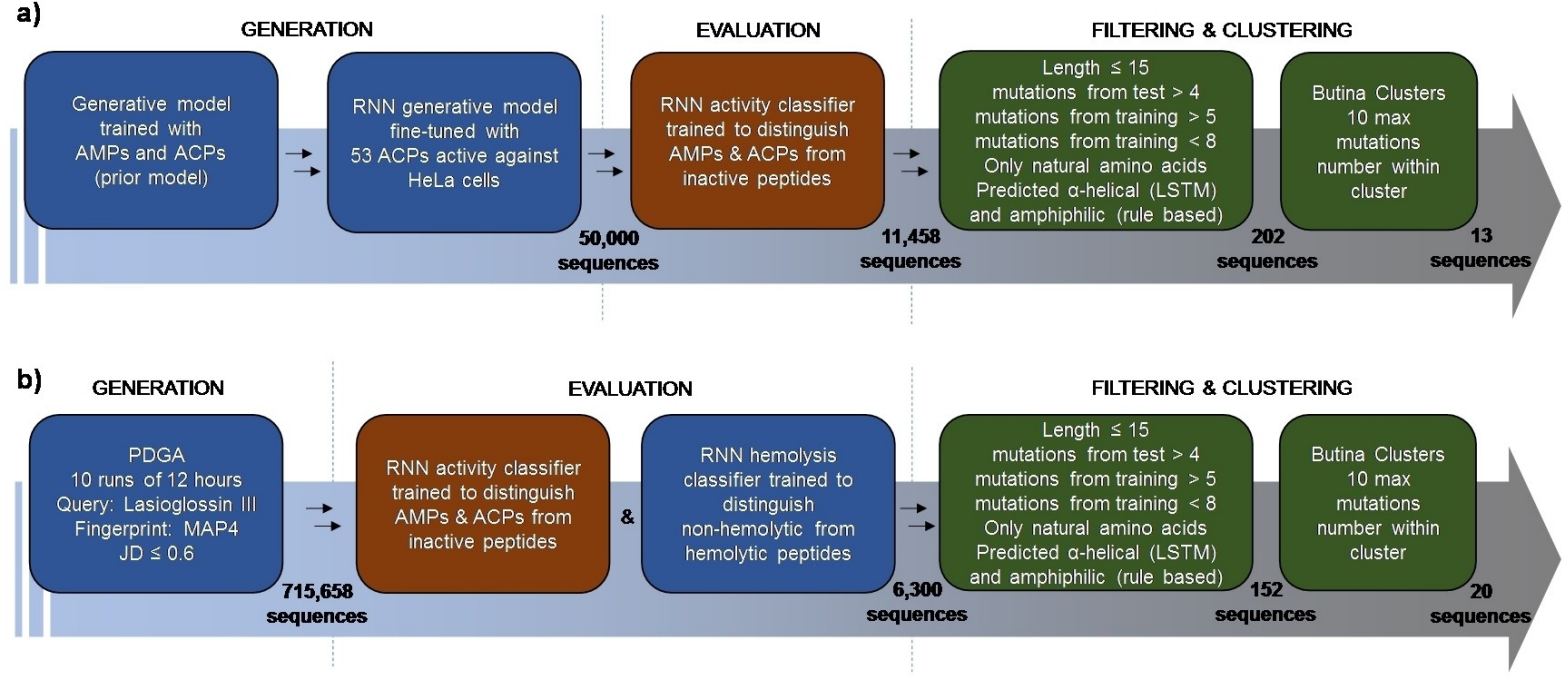
*In silico* generation, evaluation, filtering, and clustering of ACP‐like peptide sequences. **(a)** First approach: a prior generative model trained with AMPs and ACPs was fined‐tuned with 53 ACPs active against Hela cancer cells. 50,000 sequences were sampled from the fined‐tuned model and evaluated using an RNN classifier trained to distinguish ACPs and AMPs from inactive peptides. The 11,458 sequences predicted to be active were further filtered to be short, novel, within the applicability domain of the classifier, containing only natural amino acids, predicted α‐helical and amphiphilic. Finally, the obtained 202 sequences were clustered based on their sequence similarity so that the sequences within each cluster were at a maximum of 10 mutations away from each other, 13 were picked for synthesis. **(b)** Second approach: PDGA was used to find analogs of LL‐III. 10 runs of 12 hours each led to 715,658 unique sequences with a MAP4 Jaccard distance (JD) from the query below or equal to 0.6. The generated sequences were then evaluated using the same activity classifier used in the first approach and a hemolysis classifier trained to distinguish between hemolytic and non‐hemolytic peptides. The 6,300 sequences predicted to be active and non‐hemolytic were then filtered and clustered as in the first approach, and 20 sequences were selected for further synthesis.

Considering that 46 of the 53 ACPs used for transfer learning were predicted to be hemolytic, we did not apply a hemolysis classifier to this set. Nevertheless, we filtered sequences to be short (≤15 residues), as well as novel yet within the classifier's applicability domain (at least 5 mutations relative to test set and 6 to 7 mutations from the training set). We further restricted the selection to sequences containing only L‐enantiomeric residues and predicted to be >80 % α‐helical using helicities predicted by SPIDER3,^[40]^ a neural network trained with data from the Protein Data Bank, and with a calculated hydrophobic moment^[41]^ >0.3 (corresponding to median values of DBAASP active sequences: 0.83 and 0.31). This procedure retained 202 peptide sequences (as anticipated, 94 % of these were predicted hemolytic by our classifier), from which we selected thirteen by clustering for synthesis and evaluation (Figure 1a).

For our second approach, we aimed to select non‐hemolytic ACPs directly. In view of the sparsity of training data for non‐hemolytic ACPs, we focused on LL‐III due to its short length (15 residues) and documented non‐hemolytic ACP activity below 200 μM.[^39^, ^42^, ^43^, ^44^] To generate LL–III analogs, we used PDGA^[38]^ with the molecular fingerprint MAP4 as similarity measure. This fingerprint is well‐suited for virtual screening of molecules of any size range including peptides.^[45]^ The MAP4 driven PDGA generated 715,658 LL‐III analogs, which we then passed through our RNN general activity classifier and our RNN hemolysis classifier, leaving 6,300 sequences (0.88 %) as potentially active and non‐hemolytic. Applying the same sequence length, novelty, predicted helicity and hydrophobic moment criteria as above further reduced the list to 153 peptides, from which we selected 20 sequences by clustering for synthesis and evaluation (Figure 1b). Note that randomly mutating lasioglossin III, although not taking chemical similarity into account as PDGA does, generated a population of sequences that was similarly trimmed town by the activity and hemolysis classifiers (see Supporting Information for details).

**Synthesis and bioactivity testing**. We prepared the 33 selected peptides, as well as the reference ACP LL‐III, by semi‐automated high‐temperature Fmoc solid‐phase peptide synthesis and obtained pure samples by preparative HPLC. All peptides were well‐behaved in terms of aqueous solubility, in line with the presence of two to seven cationic residues (lysine or arginine) in each sequence (Table 1). To measure potential ACP activity, we exposed HeLa cells to 50 μM of each peptide for 72 h and quantified remaining live cells with Alamar Blue. We performed full IC_50_ determination for active sequences under the same conditions and similarly profiled HEK293 cells as a model for non‐cancer cells. Finally, we determined peptide concentrations causing 50 % hemolysis (HC_50_) on human red blood cells (hRBC) by serial 2‐fold dilution.

**Table 1:** Synthesis and activity of peptides.

		Toxicity^[b]^ IC_50_/μM		Hemolysis^[c]^ HC_50_/μM	MIC^[d]^ μg/mL		CD^[e]^	% Vesicle leakage^[f]^	
nr.	Sequence^[a]^	HeLa	HEK 293	hRBC	*PAO1*	*A. baumannii*	% α‐helix	PC	PG
A1	FAKKFFKKFAKFAFK	8.2±0.5	15±0.7	>200	8	8	72	79	43
A2	WFKRILKYLKKLV	8.4±0.5	7.8±0.2	60±8	8	4	66	78	29
A3	WLNALKKILGHLIRH	8.2±0.8	13±0.7	30±5	16	4	79	100	37
A4	KYLKYLVRLVGRLYR	12±1.4	13±1.1	61±10	16	4	68	96	56
A5	WKRIVRIIRWIRKYY	18±0.2	14±0.6	93±4	>16	>16	74	100	46
A6	FAARILRAWFRFLRR	11±2	7.5±0.5	23±2	>16	16	75	93	35
A7	SISRLWHSLLRHLLH	19±1	19±4	23±3	>16	4	76	100	100
A8	KNFKKLMKKVASVL	>50	>50	>400	8	4	51	16	97
A9	SFSKWMGKLKNIFKK	>50	>50	>400	8	8	50	18	32
A10	LLRHCLRRIRDRLV	>50	>50	>400	16	8	70	56	67
A11	KWRSKIKKIMRTFK	>50	>50	>400	16	16	46	11	32
A12	GLLGRLAKLLANS	>50	>50	>400	16	16	49	1	3
A13	VFRQWQKIMRRLVRR	>50	>50	>400	>16	16	49	2	5
LL–III^[g]^	VNWKKILGKIIKVVK	6.0±0.5	15±3	>200	4‐8	4	74	99	72
B1	ANWKKWIGKVIKLVK	5.5±0.8	12±2	>200	4	4	70	99	77
B2	NWKKILGKILDHLAC	7.0±1.4	6.7±0.5	322±27	>16	8	68	94	100
B3	ANWKKILKRLCDI	22±0.5	28±5	166±4	>16	16	71	62	99
B4	NWKKILGKICR	>50	>50	>400	4	4	49	51	99
B5	KNWKKIIKKVVK	>50	>50	>400	4	16	35	11	99
B6	VNVWKKIGRLVKIVK	>50	>50	>400	8	4	60	50	74
B7	NEWKKIKKIIKIVK	>50	>50	>400	16	16	49	24	28
B8	KWRQLGKKIIKVAK	>50	>50	>400	16	16	51	12	99
B9	NWKKIRKLGKVVKKI	>50	>50	>400	16	16	40	28	80
B10	VVNNWKKKIIKVIK	>50	>50	>400	>16	>16	48	3	66
B11	DWHKIGKKVIKVIK	>50	>50	>400	>16	>16	53	14	99
B12	KWNNILGKLGKLAR	>50	>50	>400	>16	>16	46	4	14
B13	NVVGRLGKIVKIVK	>50	>50	>400	>16	>16	46	1	30
B14	NPKVFLKKIIKVVK	>50	>50	>400	>16	>16	54	0	0
B15	ADVWKKVIKVIK	>50	>50	>400	>16	>16	42	2	16
B16	WRGKIGKIIKAVK	>50	>50	>400	>16	>16	60	16	21
B17	NWKKILGRLGEKG	>50	>50	>400	>16	>16	26	0	13
B18	KNWKKIVHDIKNS	>50	>50	>400	>16	>16	38	1	14
B19	NWKKILGKVIDDMKM	>50	>50	>400	>16	>16	58	16	95
B20	DKFSEKLGKIIKIVK	>50	>50	>400	>16	>16	62	5	51
DLL‐III	vnwkkilgkiikvvk	5.0±0.7	14.5±2.0	>200	4	2	74	n.d.	n.d.
DA1	fakkffkkfakfafk	7.9±0.3	15.0±2.3	>200	4	4	71	n.d.	n.d.
DB1	anwkkwigkviklvk	6.2±1.1	13.1±2.8	>200	4	2	71	n.d.	n.d.

[a] All peptides were synthesized with C‐terminal amidation. [b] IC_50_ was determined after 72 h incubation at 37 °C in DMEM high glucose medium supplemented with 10 % FBS. [c] HC_50_ was measured on human red blood cells in 10 mM phosphate buffer saline, pH 7.4, 25 °C. Triton X‐100 was used as a positive control. [d] MIC was determined after incubation for 16–20 h at 37 °C in MH medium. [e] Circular dichroism spectra were measured at concentration 100 μg/mL of peptides in 10 mM phosphate buffer, pH 7.4 in a presence of 5 mM DPC. Percentage of α‐helical structure was calculated by DichroWeb. [f] Fluorescein leakage from phosphatidyl choline (PC) or phosphatidyl glycerol (PG) vesicles was measured in buffer (10 mM TRIS, 107 mM NaCl, pH 7.4) in the presence of 10 μg/mL of peptides. 0.012 % Triton in buffer was used as positive control. [g] Parent peptide *lasioglossin* III (LL‐III) used for PDGA was synthesized for comparison. n.d.– not determined.

Seven of the thirteen peptides in the RNN generated series showed substantial ACP activity on HeLa cells, with IC_50_ values in the range 8–19 μM. However, the peptides lacked selectivity against HEK293 cells as a model of non‐cancer cells. Furthermore, except for the most active peptides, they were all strongly hemolytic, in line with the fact that most ACPs used for transfer learning were predicted to be hemolytic. In the PDGA generated series by contrast, only 3 of the 20 LL‐III analogs showed activity against HeLa cells, again with IC_50_ values in the low micromolar range, and an approximately 2‐fold selectivity against HEK293 cells, in line with the properties of the parent ACP LL‐III. In this case however, the peptides were all non‐ or only weakly hemolytic, presumably reflecting the effect of the hemolysis classifier on the selection. Additional testing of the three most active ACPs identified, namely A1, B1 and B2, showed comparable single digit micromolar activities against MCF‐7 (breast cancer) and MB‐MDA‐231 (triple‐negative breast cancer) cell lines and 2‐fold selectivity against MCF‐10a (non‐cancerous breast) cells line (Table S2).

Considering that many ACPs are also antibacterial,^[46]^ we determined minimum inhibitory concentrations (MIC) on *Pseudomonas aeruginosa* (PAO1) and *Acinetobacter baumannii*, two Gram‐negative bacteria which are often sensitive to membrane disruptive AMPs.[^15^, ^47^, ^48^] Indeed, all but one of the synthesized peptides from the first series showed substantial antibacterial activities (MIC≤16 μg/mL) independent of their hemolytic activities. In the second series, 8 of the 20 peptides as well as LL‐III also showed substantial antibacterial activity.

Taken together, the bioactivity data showed that both approaches led to new, active and partly non‐hemolytic ACPs. However, the hemolysis classifier apparently worked against ACP activity since applying this classifier in the PDGA set resulted in fewer active sequences compared to the RNN generated set used without hemolysis classifier. The most active ACPs discovered were the non‐hemolytic peptides A1 from the RNN generated series and B1 from the PDGA series, which showed a comparable activity to the reference ACP LL‐III.

**Figure 2:**
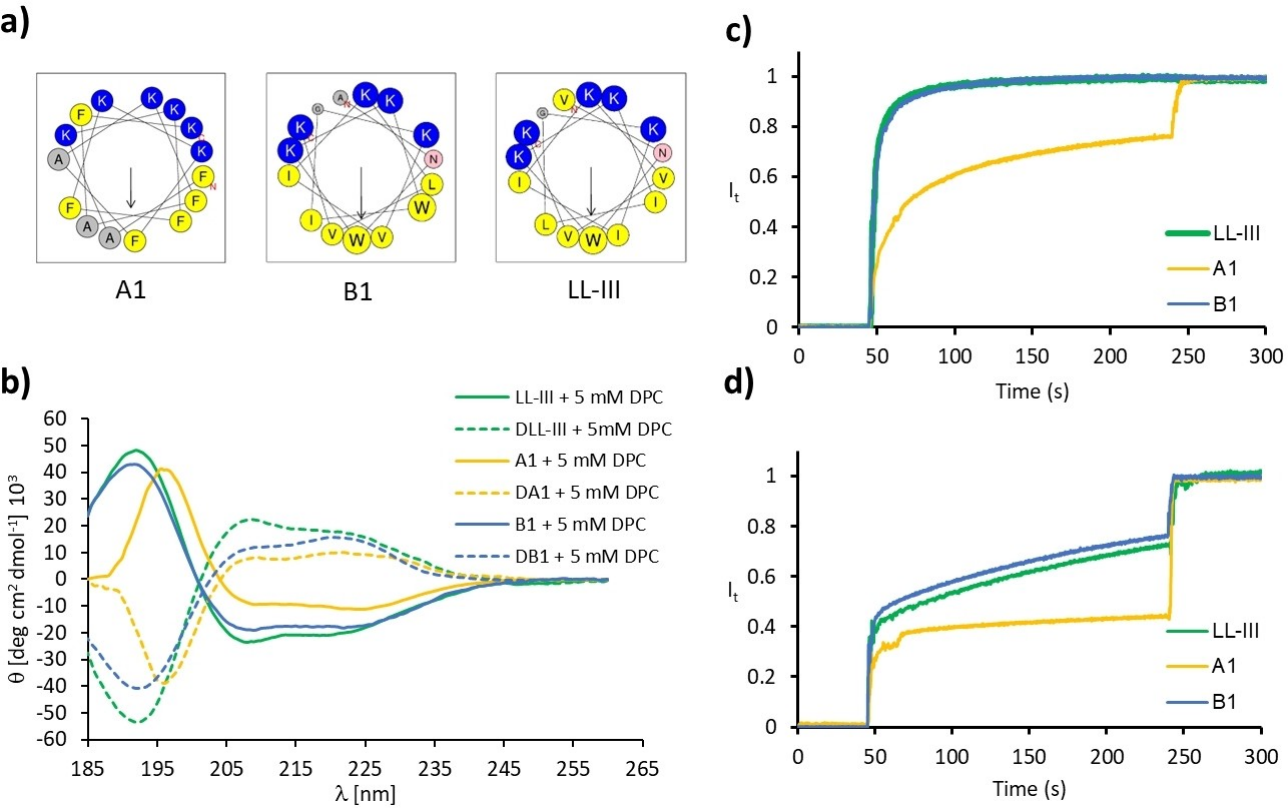
Helical properties of ACPs A1, B1 and LL‐III. (**a)** Helix properties predicted by HeliQuest.^[51]^ Circle size proportional to side‐chain size, blue indicates cationic residues, red indicates anionic residues, yellow indicates hydrophobic residues, grey indicates alanine and glycine, pink indicates asparagine. Arrows represent the helical hydrophobic moment. (**b**) CD spectra of hit peptides (100 μg/mL) in 10 mM phosphate buffer pH 7.4 in a presence of 5 mM DPC. (**c**) Vesicle leakage experiment using 5(6)‐carboxyfluorescein, induced by selected peptides at 10 μg/mL. Fluorescein leakage assay from egg yolk phosphatidyl choline (PC) lipid vesicles. (**d**) Fluorescein leakage assay from egg yolk phosphatidyl glycerol (PG) lipid vesicles. Vesicles were suspended in buffer (10 mM TRIS, 107 mM NaCl, pH 7.4) and compounds were added after 45 sec. After 240 seconds 1.2 % Triton X‐100 was added for full release of fluorescein.

**Mechanistic studies**. In view of the selection procedure applied, we anticipated that our peptides would behave as membrane disruptive α‐helices similar to most known ACPs.[^5^, ^49^, ^50^] Although the activities of the synthesized peptides varied strongly, inspection of helix‐wheel models showed that all the sequences synthesized could indeed be expected to form amphiphilic and potentially membrane disruptive α‐helices, in line with our selection for high predicted hydrophobic moment (Figure 2a and S6). To investigate whether this was the case, we measured circular dichroism (CD) spectra in neutral phosphate buffer, with optional addition of 5 mM dodecyl phosphocholine (DPC), which mimics the membrane surface and induces folding of α‐helical amphiphilic peptides. The peptides indeed behaved as typical α‐helical amphiphiles by showing an unordered conformation in neutral phosphate buffer but a substantial α‐helical fraction in the presence of 5 mM DPC (Figure 2b, S7–S8, Table 1). However, contrary to the predicted α‐helicity, which was equally high for all synthesized peptides, experimental helicity varied strongly between the different peptides and was correlated with ACP and AMP activity. For instance, helicity was higher in sequences with anticancer activity (66–79 % α‐helix) compared to those showing only antibacterial effects (35–70 % α‐helix) and those lacking any activity (26–60 % α‐helix).

To measure membrane disruptive activities directly, we performed fluorescein leakage assays with vesicles made from either phosphatidyl glycerol (PG) as an anionic lipid mimicking bacterial and cancer cell membranes, or phosphatidyl choline (PC) as a zwitterionic lipid mimicking the neutral membrane of healthy eukaryotic cells (Figure 2c/d, S9–S10, Table 1). In the RNN series, all active ACPs showed strong leakage activity at 10 μg/mL on PC vesicles. Furthermore, all peptides from this series except the least active A12 and A13 were also active on PG vesicles. In the PDGA series, only the most active ACPs (LL‐III, B1 and B2) showed strong activity against PC vesicles, while activity on PG vesicles was visible in the most active AMPs (LL‐III, B1–B9) as well as in two inactive peptides (B10 and B11). Overall, these data showed that the experimental percentage of α‐helix also varied with membrane disruptive effects on vesicle model systems. The fact that the most active ACPs discovered, peptides A1 and B1, were among the most α‐helical and showed very strong vesicle leakage activity, was consistent with a membrane disruptive mechanism of action.

**Figure 3:**
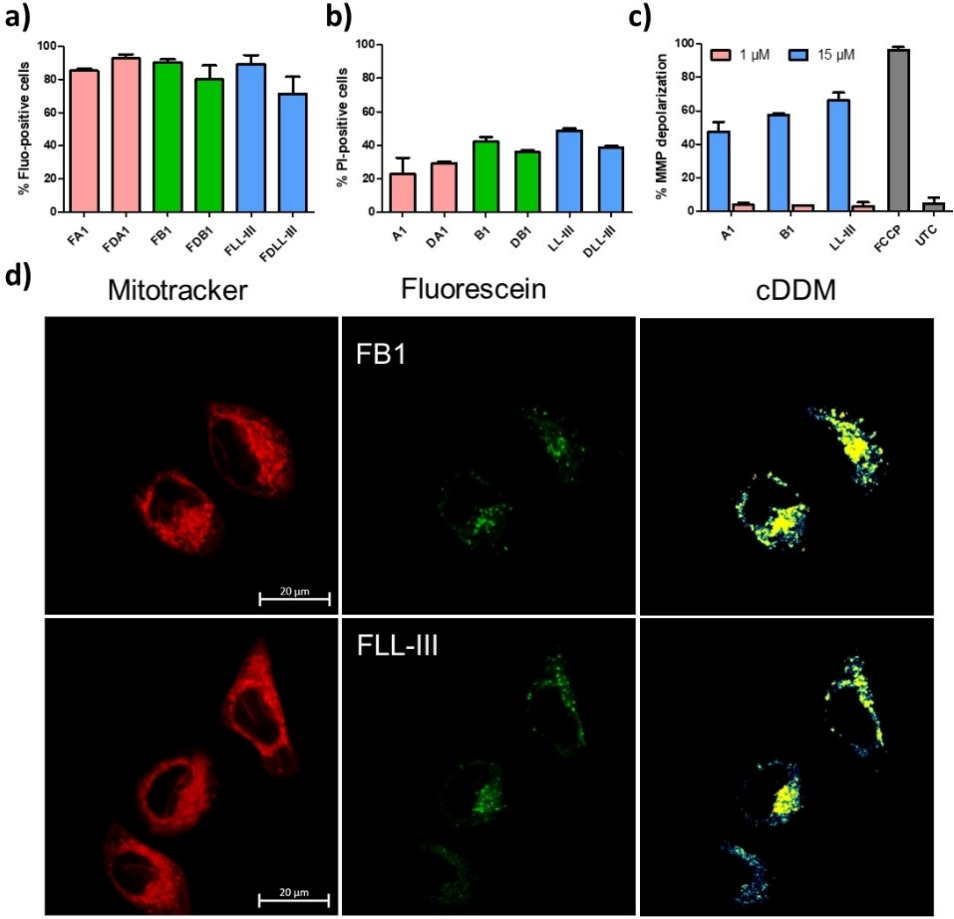
Interaction of ACPs with HeLa cells. (**a**) Cellular internalization of fluorescein‐labelled peptides. HeLa cells were treated with 10 μM of fluorescein‐labelled peptides, incubated for 3 h and analyzed by flow cytometry. (**b**) Propidium Iodine (PI) entrance to HeLa cells, treated by 10 μM of peptides and incubated for 15 min, was detected by flow cytometry. (**c**) Mitochondrial Membrane Potential (MMP) depolarization as detected by flow cytometry. HeLa cells were treated by 1 μM (pink),15 μM (blue) of peptides and incubated for 120 min and 15 min respectively. UTC – untreated cells, Carbonyl cyanide‐p‐trifluoromethoxyphenylhydrazone (FCCP) 50 μM was used as a positive control. (**d**) Colocalization analysis of fluorescein‐labelled peptides with mitochondria (live cells). HeLa cells were treated with 10 μM of peptide‐fluorescein and incubated for 1 h. Images were taken on a Zeiss LSM 880 confocal microscope with Oil compatible lens x63/1.3. cDDM: Co‐Density Distribution Map, built by coDDMaker software.^[52]^

The fact that our ACPs were non‐hemolytic and antibacterial raised the possibility that they might act by penetrating the cell and inducing apoptosis by disruption of the mitochondrial membrane,^[46]^ which resembles the bacterial membrane, rather than by direct disruption of the cell membrane. To investigate this point, we focused on HeLa cells and the most active peptides A1 and B1. Similar to LL‐III, the anticancer as well as the antibacterial activity of A1 and B1 was preserved in their D‐enantiomers DA1 and DB1, and hemolytic, helical properties were comparable to L‐enantiomers (Figure 2b, Table 1). Flow cytometry experiments of fluorescent analogs FA1, FB1, FDA1 and FDB1, FLL‐III, FDLL‐III obtained by acylation of N‐termini with 5/6‐carboxyfluorescein showed that the peptides bound to HeLa cells (Figure 3a) and permeabilized the cell membrane to propidium iodide (Figure 3b, S12), leading to cell death in the range 5–10 μM corresponding to their IC_50_ values. The comparable cell permeabilizing activity of both enantiomers was consistent with membrane disruption.

On the other hand, confocal imaging with FB1 and analogs showed that the peptides penetrated into cells and colocalized with mitochondria and partly the nucleus, similar to previous reports with LL‐III[^42^, ^53^, ^54^] (Figure 3d, Figure S11). However, we could not detect apoptosis by Annexin V staining reliably due to a faster cell death induced by disruption of the cell membrane. Furthermore, we detected mitochondrial membrane potential (MMP) depolarization by flow cytometry but only at a concentration (15 μM) higher than their IC_50_ values (Figure 3c, S13). Taken together, these experiments suggested that the newly discovered ACPs A1 and B1 killed HeLa cells primarily by disruption of the cytoplasmic membrane.

**Figure 4:**
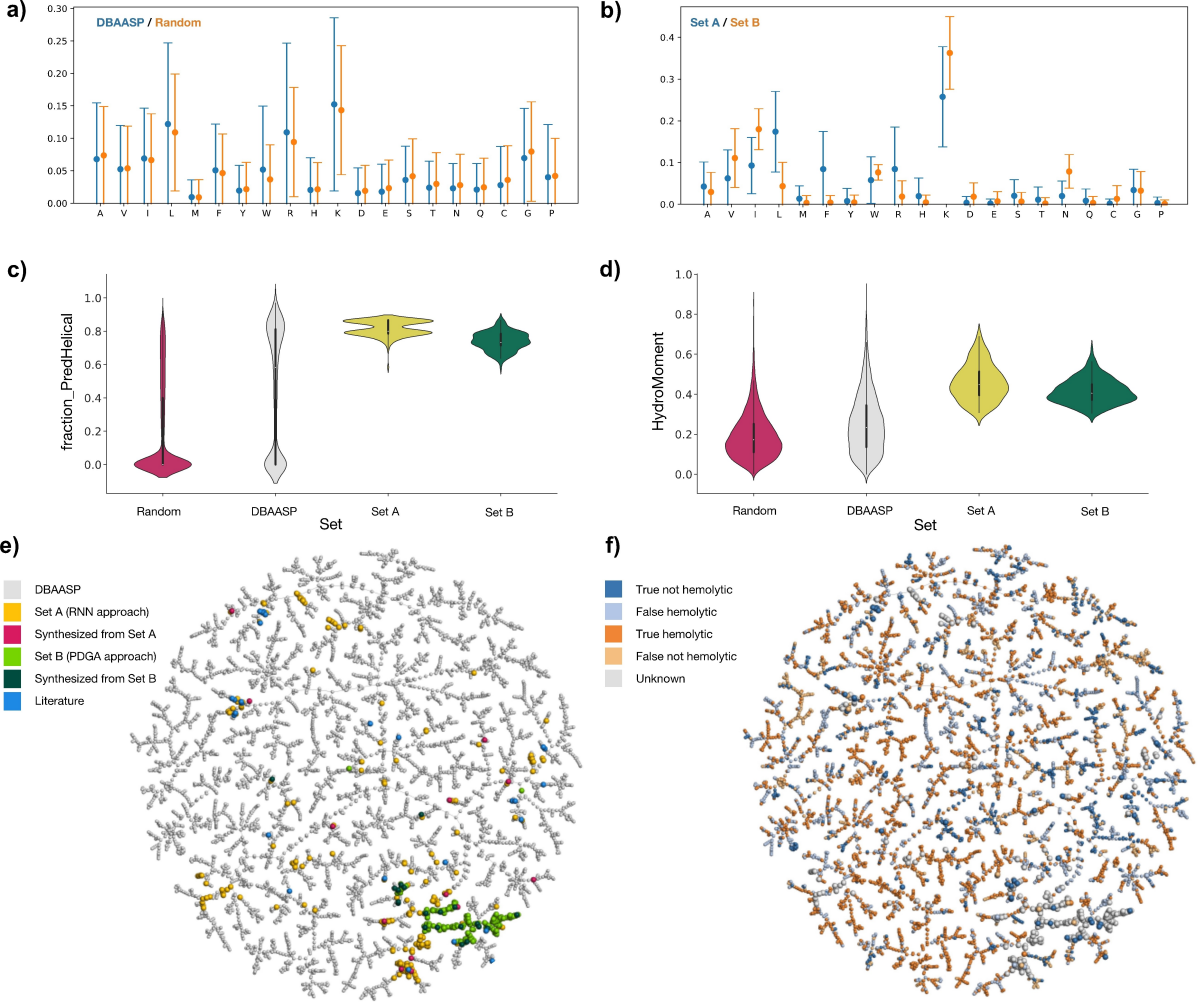
Chemical space analysis of DBAASP and the selected ACPs. **(a)** Mean amino acid fractions and standard deviations of all sequences contained in the DBAASP and random sets. **(b)** Mean amino acid fractions and standard deviations of all sequences contained in set A (from RNN generative model, fist approach) and set B (from PDGA, second approach). **(c)** Distributions of the fraction of helical residues predicted with SPIDER3^[40]^ in sequences from DBAASP, random set, set A and set B. **(d)** Distributions of hydrophobic moment calculated according to Eisenberg et al.^[41]^ in sequences from DBAASP, random set, set A and set B. **(e–f)** TMAP calculated on the MAP4 fingerprint for a combined set containing DBAASP sequences, set A and set B from this work, and a previously reported set of AMPs discovered by ML (label “Literature”).^[15]^ Color‐coding indicates either compound category (**e**) or hemolysis classification (**f**). An interactive version of the TMAP featuring additional color‐codes is accessible at https://tm.gdb.tools/map4/anticancer_peptides_tmap/

**Chemical space analysis**. To better understand the selection procedure applied to identify our ACPs, we analyzed the amino acid composition of relevant peptide sets and subsets and mapped the chemical space covered by the source database DBAASP and the selected peptides (Figure 4, Table S1, Figure S1–S2). DBAASP peptides contained predominantly cationic amphiphilic sequences, as evidenced by the amino acid composition showing an unusually high percentage of cationic (lysine and arginines) and hydrophobic residues (leucine, alanine, isoleucine, valine and phenylalanine, Figure 4a). A similar prevalence of lysine and leucine was also apparent in the peptides selected in the first (RNN, Set A, Figure 4b) and second approach (PDGA, Set B, Figure 4b). Compared to random sequences with the same composition, a much higher fraction DBAASP peptides were predicted to form α‐helices with a slightly higher amphiphilicity (Figure 4c/d). The selected peptides from both approaches were skewed towards high values in both predictions as a consequence of the applied filters, however it should be noted that the experimental helicities of the synthesized peptides spanned a broad range of values similar to the spread in DBAASP (56±13 % α‐helix, Table 1).

The above analysis showed that the amino acid composition as well as predicted helicity and amphiphilicity of the selected peptides reflected the source DBAASP. However, there was no significant difference in terms of these three parameters between active and inactive peptides as well as between hemolytic and non‐hemolytic peptides from DBAASP, showing that these parameters were not sufficient to define ACP activity (Figure S3–S5). On the other hand, comparing active versus inactive as well as hemolytic versus non‐hemolytic sequences in DBAASP in a Tree‐map (TMAP)^[55]^ calculated using the MAP4 fingerprint showed that these different categories formed small clusters, indicating common features, which were most likely the features learned by the corresponding RNN classifiers (Figure 4f). The TMAP also showed that the peptides originating from the RNN generative model trained on the entire DBAASP (first approach) were scattered across the entire map, as were our previously reported AMPs selected by a similar approach, in line with the fact that the RNN generative model learned features across the entire database (Figure 4e). By contrast, the LL‐III analogs generated using PDGA (second approach) were grouped around the source LL‐III sequence, reflecting the more focused generation of the genetic algorithm optimizing similarity to a given target.

## Conclusion

Despite of the many previous reports showing that ML methods can classify bioactive peptides including membrane disruptive ACPs, experimental ML based searches reported to date yielded hemolytic ACPs, requiring additional optimization to reduce their hemolysis.[^32^, ^33^] Here we showed that combining an RNN generative model or a genetic algorithm with activity and hemolysis classifiers allows to identify new non‐hemolytic ACPs directly. Detailed investigations showed that these new ACPs formed amphiphilic α‐helices and displayed membrane disruptive activities on model vesicles, significant antibacterial activities, and killed cancer cells by disruption of the outer membrane, thereby reproducing the properties of LL‐III, a known and typical natural ACP.

The experimental evaluation was essential to identify true actives, and revealed that predictions of activity, hemolysis, and α‐helicity, which were all based on neural networks, had only limited predictive power for the sequences tested, probably because these sequences reside at the interface between two incompatible regions of chemical space, namely non‐hemolytic sequences, which are mostly non‐amphiphilic, non‐membrane disruptive, and ACP active sequences, which are mostly amphiphilic and membrane disruptive (our hemolysis classifier was 73 % correct on the 33 peptides tested here, and a very recently reported hemolysis prediction tool achieved 79 % correct predictions on the same set).^[56]^ Importantly, our experiments delivered new sets of actives and inactives that can now be used to refine ML models in this critical region of sequence space. We therefore envision that iterative rounds of ML‐guided design and experiments might allow to expand the currently very limited set of non‐hemolytic ACPs and eventually lead to a better understanding of activity and selectivity in this class of compounds.

## Experimental Section

**Recurrent Neural Network (RNN) models**. The RNN activity and hemolysis classifier and the prior generative model used in this study were previously developed for the recently published work [11]using activity and hemolysis data of APCs and AMPs extracted from the manually curated DBAASP (Database of Antimicrobial Activity and Structure of Peptides).[^30^, ^31^] Sequences with a registered activity measure below 10 μM, or 10 000 nM, or 32 μg/mL towards at least one reported target were labeled as active. When present, activity against human erythrocytes was used to label the sequences as hemolytic or non‐hemolytic. The concentration was normalized to μM and sequences causing less than 20 % of hemolysis with a concentration equal or above 50 μM were flagged as non‐hemolytic. In the present study, the RNN prior generative model was fine‐tuned using transfer learning (TL) and 53 linear and natural peptides active against HeLa cancer cells extracted from the DBAASP.TL consisted in a second training of the prior model using the 37 out of the 53 peptides against HeLa which were present in the training set previously used for the training of the prior model. The remaining 16 sequences were used as test set. Negative log‐likelihood loss (NLLL) and Stochastic gradient descent with a momentum of 0.9 and a learning rate of 0.00001 were used and the training was stopped when the NLLL of the validation set reached its minimum. 50,000 peptide sequences were sampled from the fine‐tuned generative model.

**Peptide Design Genetic Algorithm (PDGA)**. The peptide design genetic algorithm (PDGA)^[38]^ was adapted to use the MinHashed atom pair fingerprint of diameter 4 (MAP4) in its string version.^[45]^ This version of the MAP4 fingerprint can be calculated using the parameter “return_strings=True” and it returns the shingles of the encoded molecules. In its fitness function, the MAP4 PDGA evaluates each generated peptide structure based on the Jaccard distance (JD) between the shingles of its MAP4 fingerprint and the shingles of the MAP4 fingerprint of the given query. The MAP4 PDGA was run 10 times in parallel for 12 hours using the anticancer peptides Lasioglossin III as query, an initial population of 100 peptides with a randomly generated sequence, a mutation rate and a generation gap of 0.5, linear topology, and excluding non‐natural building blocks. The runs resulted in the generation of 715,658 unique sequences.

**Properties calculation**. The Levenshtein distance (LD) from the nearest neighbor (NN) present in the training and the test used to implement the RNN activity and hemolysis classifier^[15]^ was calculated using the Levenshtein Python package.[^57^, ^58^] Hemolysis and activity were predicted by the respective classifiers converting the probabilistic prediction values into binary classification using the threshold that kept the prediction of false positive below 6 % (0.99205756 for the activity classifier and 0.99981695 for the hemolysis classifier).The helicity prediction was performed using SPIDER3,^[40]^ and the helicity fraction was calculated as the number of residues predicted helical in a peptide sequence divided by the length of the sequence itself. The hydrophobic moment was calculated as described by Eisenberg et al.^[41]^

**Peptide sequences selection**. The generated sequences sampled from the fine‐tuned generative model in the first approach and generated with the PDGA in the second approach were filtered based on multiple criteria. First, to ensure novelty, we have chosen sequences with LD >5 from the classifiers training sets and LD >4 from the classifiers test set. Second, we removed sequences that were outside the applicability domain of the classifiers. To do so, the minimum LD of every test set compound to the training set was calculated, and the applicability domain of the classifiers was set to be the 90 % quantile. This led to the exclusion of all generated sequences with a LD distance of 8 or more to the training set of the classifiers. Only sequences up to 15 residues were selected to facilitate the synthesis process and due to the low percentage of D amino acids in the training set, sequences containing D‐residues were excluded. Since helicity and amphiphilicity often correlate with antimicrobial activity, we selected sequences with a predicted helicity fraction above 0.8 and an Eisenberg hydrophobic moment above 0.3. The thresholds for the predicted helicity fraction and hydrophobic moment were chosen based on the median values of the active sequences in the training and test, respectively 0.83 and 0.31. The filtered sequences were clustered using the RDKit Butina module with a threshold of 10 and the Levenshtein distance as distance function, and the center of each cluster was picked. The workflow resulted in 13 sequences for the first approach and in 20 sequences for the second approach.

**Data retrieval for TMAP visualization**. The entire DBAASP was downloaded from the provided website (https://dbaasp.org) and sequences containing unnatural or D‐enantiomeric amino acids removed to obtain a total of 12,497 monomeric sequences. Only the lowest value in the “TARGET ACTIVITY – ACTIVITY (μg/ml) (Calculated By DBAASP)” column and the highest value in the “HEMOLYTIC CYTOTOXIC ACTIVITY – ACTIVITY (μg/ml) (Calculated By DBAASP)” column was kept for each sequence. A threshold of <4 μg/ml for activity and <200 μg/ml for hemolysis were applied to determine whether a sequence is active or hemolytic respectively. A random set of 20’000 sequences was generated to match the relative count of each amino acid in the DBAASP set. The RNN (202 sequences) and PDGA (153 sequences) sets were composed of all sequences generated by the RNN and PDGA approach that passed every selection filter. The literature set contained all tested bioactive sequences from our recently published work.^[15]^ Size and composition of the datasets are listed in the SI (Table S1).

**TMAP visualization**. The default version of the MAP4 fingerprint was used to encode all sequences contained in the DBAASP, RNN, PDGA and literature^[15]^ sets. The indices generated by the MinHash procedure of the MAP4 calculation were used to create a locality‐sensitive hashing (LSH) forest^[59]^ of 64 trees. Then, for each structure, the 15 approximate nearest neighbors (NNs) in the MAP4 feature space were extracted from the LSH forest, and the tree layout was calculated. The LSH forest and the minimum spanning tree layout were calculated using the TMAP open‐source code. Finally, Faerun^[60]^ was used to display the obtained layout interactively.

**Evaluation metrics**. ROC AUC is the area under the ROC curve, and the ROC curve is obtained by plotting the true positive rate (TPR) against the false positive rate (FPR):

$$TPR=\frac{TP}{TP+FP}$$

$$FPR=\frac{FP}{TP+FP}$$

where TP stands for true positives, TN for true negatives, FP for false positives, and FN for false negatives predicted by the classifier.

The F1 score is defined as the harmonic mean of precision and recall:

Precision=TPR

$$Recall=\frac{TP}{TP+FN}$$

$$F1\;score=2\times \frac{\left(Precision\times Recall\right)}{\left(Precision+Recall\right)}$$

The balanced accuracy is defined as:

$$Balanced\;accuracy=\frac{TPR+\frac{TN}{TN+FN}}{2}$$

The Matthews correlation coefficient (MCC) is a correlation between the observed and the predicted class and it is defined as:

$$MCC=\frac{TP\times TN-FP\times FN}{\sqrt{\left(TP+FP)(TP+FN)(TN+FP)(TN+FN\right)}}$$

**Solid‐phase peptide synthesis**. Linear peptides were synthesized manually using 150–300 mg of Rink Amide AM LL resin (0.29 mmol/g) by standard 9‐fuorenylmethoxycarbonyl (Fmoc) Solid Phase Peptide Synthesis at 60 °C under nitrogen bubbling. The resin was swollen in DMF for 10 min. Double deprotection of the Fmoc group was performed using a solution containing 5 % w/v piperazine, 2 % v/v 1,8 diazabicyclo(5.4.0)undec‐7‐ene (DBU), 10 % v/v of 2‐butanol in DMF during 1 min and 4 min respectively. The resin was washed with DMF (5×8 mL DMF) after deprotection. Coupling step (2×8 min) was performed with 3 mL of amino acid (0.2 M), 2 mL of DIC (0.8 M) and 1.5 mL of Oxyma (0.8 M) in DMF. Resin was washed with DMF between couplings (2×8 mL) and after second coupling (3×8 mL). For sequences containing aspartic or glutamic acid deprotection solution was exchanged to 20 % v/v piperidine+0.7 % v/v formic acid in DMF to avoid aspartimide, glutamide and side products formation. The cleavage from resin was carried out by treating the resins with 7 mL of a TFA/TIS/DODT/H_2_O (95/2/2/1, v/v/v/v) solution for 3 h. The peptide solutions were precipitated with 30 mL of cold *tert*‐butylmethyl ether (TBME), centrifuged for 10 min at 3500 rpm (twice), evaporated and dried in high vacuum for 60 min. The crude was then dissolved in a H_2_O/CH_3_CN (10/1, v/v) mixture, some drops of MeOH added when needed and purified by preparative RP‐HPLC.

**Cell Viability assay by AlamarBlue**. HeLa, HEK‐293, MCF‐7, MDA‐MB‐23, MCF‐10a cells were seeded into 96 well plates, in amount 4×10^3^, 2×10^4^, 6×10^3^, 5×10^3^, 9×10^3^ cells/well respectively, the day before the experiment. HEK‐293 were seeded into well plates, pretreated with solution of poly‐L‐Lysine. The medium was removed and the compounds at increasing concentration were added into the wells. The cells were incubated for 72 h in 200 μL/well at 37 °C in corresponding medium in the presence of 5 % CO_2_. After incubation time the medium was removed and replaced by 100 μL/well of medium containing 10 % AlamarBlue. The cells were incubated for 3–5 h at 37 °C with 5 % CO_2_ in a humidified atmosphere. The fluorescence was then measured on a Tecan Infinite M1000 Pro plate reader at λex 560 nm and λem 590 nm. The value was normalized according to the untreated cells.

**Hemolysis assay**. Compounds were subjected to a hemolysis assay to assess the hemolytic effect on human red blood cells (hRBCs). The blood was obtained from Interregionale Blutspende SRK AG, Bern, Switzerland. 1.5 mL of whole blood was centrifuged at 3000 rpm for 15 min at 4 °C. The plasma was discarded, and the hRBC pellet was re‐suspended in 5 mL of PBS (pH 7.4) then centrifuged at 3000 rpm for 5 min at 4 °C. The washing of hRBC was repeated three times and the remaining pellet was re‐suspended in 10 mL of PBS. The samples were prepared as the initial concentration of 2000 μg/mL in PBS, added to the first well of 96‐well microtiter plate and diluted serially by 1/2 , having 100 μL of sample in every well. Controls on each plate included a blank medium control (PBS 100 μL) and a hemolytic activity control (0.1 % Triton^TM^ X‐100). 100 μL of hRBC suspension was incubated with 100 μL of each sample in PBS in V‐shape 96‐well plate. After the plates were incubated for 4h at room temperature, 100 μL of supernatant was carefully pipetted to a flat bottom, clear 96‐wells plate. Hemolysis was measured by analyzing the absorbance of free hemoglobin leaked out of compromised in the supernatants at 540 nm with a plate reader (Tecan instrument Infinite M1000). The percentage of hemolysis at each concentration was detected and HC_50_ was determined.

**Minimal inhibitory concentration (MIC) determination**. The minimal inhibitory concentration (MIC) was determined by using broth microdilution method. Antimicrobial activity was assayed against P. aeruginosa PAO1 (WT), Acinetobacter baumannii (ATCC19606). A colony of bacteria was picked and grown in Luria‐Bertani (LB) medium overnight at 37 °C. Stock solutions of 1 mg/mL of the samples were prepared in sterilized milliQ water and diluted to the beginning concentration of 128 μg/mL in 300 μL Mueller Hinton (MH)‐medium. The diluted samples were added to first well of 96‐well microtiter plate and diluted serially by 1/2 . Bacteria concentrations were quantified by measuring the optical density at 600 nm and diluted to OD600 of 0.022 in MH‐medium. 4 μL of the diluted bacterial solution was used to inoculate into the sample solutions (150 μL) with a final inoculation of about 5×10^5^ CFU/mL. The plates were then incubated at 37 °C for 18 h. For each assay, sterility (broth only) and growth control (broth with bacterial inoculum, without antibiotics) were checked with two columns in the plate. The next day, 15 μL of MTT solution was added to each well of the plate, such a way that MIC was defined as the lowest concentration of the peptide that inhibited visible growth of the tested bacteria.

**Circular dichroism spectroscopy**. Circular dichroism (CD) spectra were recorded on a Jasco J‐715 Spectropolarimeter. All the experiments were performed using Hellma Suprasil 110‐QS 0.1 cm cuvettes. Stock solutions (1.00 mg/mL) of peptides were freshly prepared in 10 mM phosphate buffer (PB, pH 7.4). The PB buffer was degassed for 10 min under high vacuum before each set of experiments. For the measurement, the peptides were diluted to 100 μg/mL with PB buffer and dodecylphosphocholine (DPC) was added to final concentration 5 mM if needed. The range of measurement was 185–260 nm, the scan rate was 20 nm/min, pitch 0.5 nm, response 16 sec and bandwidth 1.0 nm. The nitrogen flow was kept >8.5 L/min. After each measurement, the cuvettes were washed successively with 1 M HCl, milli‐Q H_2_O and PB buffer. The baseline was recorded under the same conditions and subtracted manually. Percentage of different secondary structure types was calculated by DichroWeb.^[61]^

**Lipid vesicle leakage assays**. Egg Yolk Phosphatidylcholine (EYPC), Egg Yolk Phosphatidylglycerol (EYPG) thin lipid layers were prepared by evaporating a solution of 100 mg Egg PC or Egg PG in 4 mL MeOH/CHCl_3_ (1 : 1) on a rotary evaporator at room temperature and then dried in vacuo overnight. The resulting film was then hydrated with 4 mL buffer B (50 mM 5(6)‐carboxyfluorescein, 10 mM TRIS, 10 mM NaCl, pH 7.4) for 30 min, subjected to freeze‐thaw cycles (7x) and extrusion (15x) through a polycarbonate membrane (pore size 100 nm). Extra vesicular components were removed by gel filtration (Sephadex G‐50) with buffer A (10 mM TRIS, 107 mM NaCl, pH 7.4). Final conditions: ∼2.5 mM Egg PC or Egg PG; inside: 50 mM 5(6)‐carboxyfluorescein, 10 mM TRIS, 10 mM NaCl, pH 7.4; outside: 10 mM TRIS, 107 mM NaCl, pH 7.4. Egg PC or Egg PG stock solutions (37.5 μL) were diluted to 3000 μL with a buffer A (10 mM TRIS, 107 mM NaCl, pH 7.4) in a thermostated fluorescence cuvette (25 °C) and gently stirred (final lipid concentration ∼31  μM). 5(6)‐carboxyfluorescein efflux was monitored at λem 517 nm /λex 492 nm as a function of time after addition at t=45 sec of 30 μL of peptide stock solution (1 mg/mL stock in buffer A), having final concentration 10 μg/mL. Finally, 30 μL of 1.2 % Triton X‐100 was added to the cuvette (0.012 % final concentration) at t=240 sec to reach the maximum intensity. Fluorescence intensities were then normalized to the maximal emission intensity using I(_t_)=(I_t_–I_0_) / (I_∞_–I_0_) where I_0_=I_t_ at peptide addition, I_∞_=I_t_ at saturation of lysis.

**Confocal microscopy of cells treated with fluorescein‐labelled peptides**. 8‐well chambered cover glass plates were treated with poly‐L‐Lysine for 1 h, dried at RT and the day prior treatment HeLa cells were plated at 2×10^4^ cells per well. The medium was removed, and cells were treated with fluorescein‐labelled peptides (10 μM in complete DMEM, 250 μL/well), incubated for 3 h at 37 °C in a humidified atmosphere in 5 % CO_2_ following the removal of the complete growth medium. Then, cells were washed with prewarmed PBS twice and the cell membrane was labelled with CellMask Deep Red plasma membrane stain in PBS (0.25 μL in 0.25 mL/well) and nucleus was stained with Hoechst 33258 in PBS (0.25 μL in 0.25 mL/well) for 30 min at 37 °C. After the incubation cells were washed with PBS twice and prewarmed Glycergel Mounting Medium was added. Images were taken on a Zeiss LSM 880 confocal microscope with Oil compatible lens x63/1.3.

**Colocalization studies with mitochondria**. To 8‐well chambered cover glass plates HeLa cells were seeded at 2×10^4^ cells per well and incubated overnight. Then the medium was removed, and cells were treated with fluorescein‐labelled peptides (10 μM in complete DMEM, 250 μL/well), incubated for 1 h at 37 °C in a humidified atmosphere in 5 % CO_2_ following the removal of the complete growth medium. Then cells were washed with prewarmed PBS twice and nucleus was stained with Hoechst 33258 in PBS (0.25 μL in 0.25 mL/well) for 30 min at 37 °C. Mitochondria were stained with Mitotracker Red (Thermo Fisher Scientific) according to manufacture protocol (100 nM) at 37 °C for 30 min. Staining solution was replaced with fresh prewarmed FluoroBrite DMEM Medium. Images were taken on a Zeiss LSM 880 confocal microscope with Oil compatible lens x63/1.3. Colocalization was processed with coDDM Maker Software.^[52]^

**Flow cytometry studies**. HeLa cells were plated into 96 well plate, 3×10^4^ cells/well, and allowed to adhere overnight. Medium was removed and cells were treated with fluorescein‐labelled peptides (200 μl of 10 μM solution in complete growth medium), following by incubation at 37 °C for 2 h. Untreated cells were used as a control. Then the cell medium was removed, cells were washed with PBS, trypsinized, and trypsinization was quenched with 100 μL of complete growth medium (DMEM, 10 % FBS). Cells were centrifuged at 500 g for 5 min at 15 °C and supernatant was decanted. The cells were resuspended in 100 μL 2 % FBS PBS solution and analyzed by Beckman Coulter CytoFLEX™. CytExpert 2.0 (Beckman Coulter, Miami, FL, USA) was used for acquisition and FlowJo™ Software (Ashland, OR: Becton, Dickinson and Company; 2021) for data processing.

**Propidium iodine (PI) internalization**. HeLa cells were plated into 96 well plate, 3×10^4^ cells/well, and allowed to adhere overnight. Medium was removed and cells were treated with selected peptides (10 μM in DMEM, 200 μL/well) and incubated for 10 min at 37 °C in a humidified atmosphere in 5 % CO_2_. Then the medium was removed, cells were trypsinized (30 μL/well, 0.025 %), trypsinization was quenched by 100 μL of complete growth medium (DMEM, (−) Phenol Red, 10 % FBS). Cells were directly stained with PI according to manufacture protocol for 10 min and analyzed by Beckman Coulter CytoFLEX™.

**Mitochondrial membrane depolarization assay**. HeLa cells were plated into 96 well plate, 3×10^4^ cells/well, and allowed to adhere overnight. Medium was removed and cells were treated with selected peptides (1 and 15 μM in DMEM, 200 μL/well) and incubated for 120 and 15 min at 37 °C in a humidified atmosphere in 5 % CO_2_. Untreated cells and cells treated with 50 μM FCCP (15 min) were used as negative and positive controls. The medium was removed, cells were washed with PBS, trypsinized, trypsinization was quenched by 100 μL of complete growth medium (DMEM, (−) Phenol Red, 10 % FBS) and stained by TMRE‐Mitochondrial Membrane Potential Assay Kit (Abcam, Cambridge, UK) according to manufacture protocol (100 nM) for 15 min. Right after cells were analyzed using Beckman Coulter CytoFLEX™.

## Author contributions

E.Z. designed and carried out all experiments and wrote the paper. A.C. and M.O. designed and carried out computations. J.L.R. co‐designed and supervised the study and wrote the paper. All authors read and approved the final manuscript.

## Conflict of interest

The authors declare no conflict of interest.

1

## Supporting information

As a service to our authors and readers, this journal provides supporting information supplied by the authors. Such materials are peer reviewed and may be re‐organized for online delivery, but are not copy‐edited or typeset. Technical support issues arising from supporting information (other than missing files) should be addressed to the authors.

Supporting Information

## Data Availability

The source code and datasets used for this study are available at https://github.com/reymond‐group/ML‐PDGA‐anticancer‐peptides.
