# Supplementary material for: Machine Learning Guided Discovery of Non‐Hemolytic Membrane Disruptive Anticancer Peptides
Source: ChemMedChem. 2022 Aug 5;17(17):e202200291. doi: 10.1002/cmdc.202200291 (PMC9541320; doi:10.1002/cmdc.202200291)
Supplement: Supplementary file 1 — Supporting Information [file CMDC-17-0-s001.pdf]

ChemMedChem

Supporting Information

Machine Learning Guided Discovery of Non-Hemolytic Membrane Disruptive Anticancer Peptides

Elena Zakharova, Markus Orsi, Alice Capecchi, and Jean-Louis Reymond*

Table of content

1. Supplementary figures and tables 3
    1.1. Datasets and subsets 3
    1.2. Experimental data 7
2. Materials and Reagents 14
3. Solid-phase peptide synthesis 15
    3.1 General Information 15
    3.2 Fluorescein-labelled peptides 15
4. Cell culture conditions 15
5. HPLC/MS and HRMS spectra 16
6. References 102

1. Supplementary figures and tables

1.1.Datasets and subsets

Table S1. Size and composition of peptide sequence datasets and subsets that were analyzed.

Set	Number of sequences	Description
**DBAASP**	12’497	All monomeric sequences contained in DBAASP that have at least one activity or hemolysis value annotated.
**Random**	20’000	Sequences with random length (between 5 and 25 amino acids) and amino acid distribution that matches the amino acid distribution of the DBAASP set.
**RNN**	202	Sequences sampled from the RNN approach that passed all the selection filters.
**PDGA**	153	Sequences sampled from the PDGA approach that passed all the selection filters.
**Actives**	4’046	DBAASP subset that contains sequences with at least one annotated activity value <4 µg/ml.
**Inactives**	8’538	DBAASP subset that contains sequences without any annotated activity value <4 µg/ml.
**Not hemolytic**	2’945	DBAASP subset that contains sequences with at least one annotated hemolysis value >200 µg/ml.
**Hemolytic**	2’810	DBAASP subset that contains sequences without any annotated hemolysis value >200 µg/ml.
**Active & hemolytic**	862	Inner join of actives and hemolytic subsets.
**Active & not hemolytic**	935	Inner join of active and not hemolytic subsets.
**Inactive & hemolytic**	2’048	Inner join of inactives and hemolytic subsets.
**Inactive & not hemolytic**	2’172	Inner join of inactives and not hemolytic subsets.

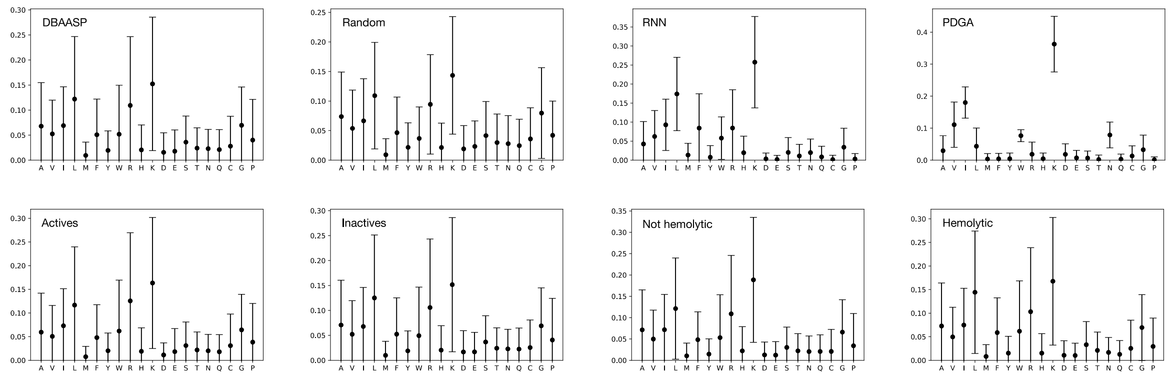

Fig. S1. Mean fractions and standard deviation of each amino acid in the DBAASP, random, RNN and PDGA sets and the actives, inactives, not hemolytic, and hemolytic subsets.

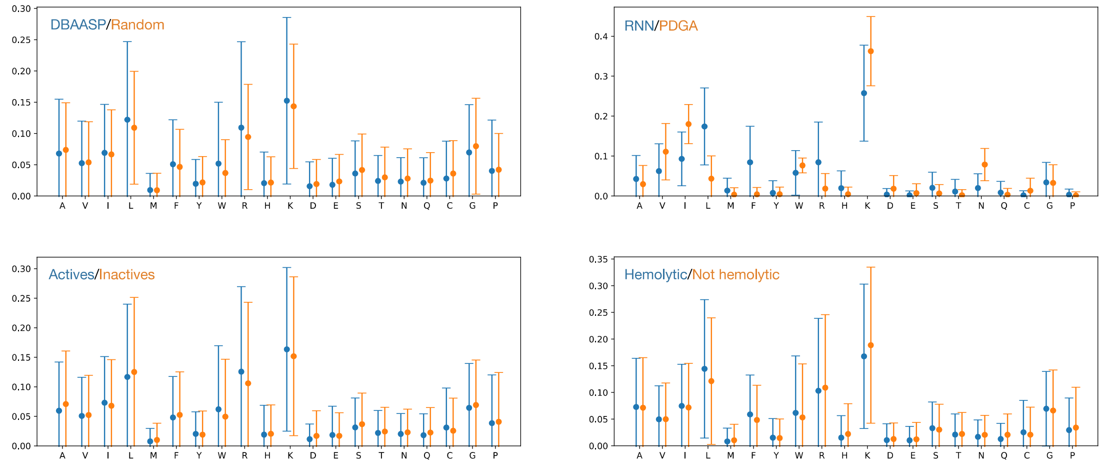

Fig. S2. Grouped mean fractions and standard deviations of each amino acid in the DBAASP/Random, RNN/PDGA sets and the actives/inactives and not hemolytic/hemolytic subsets.

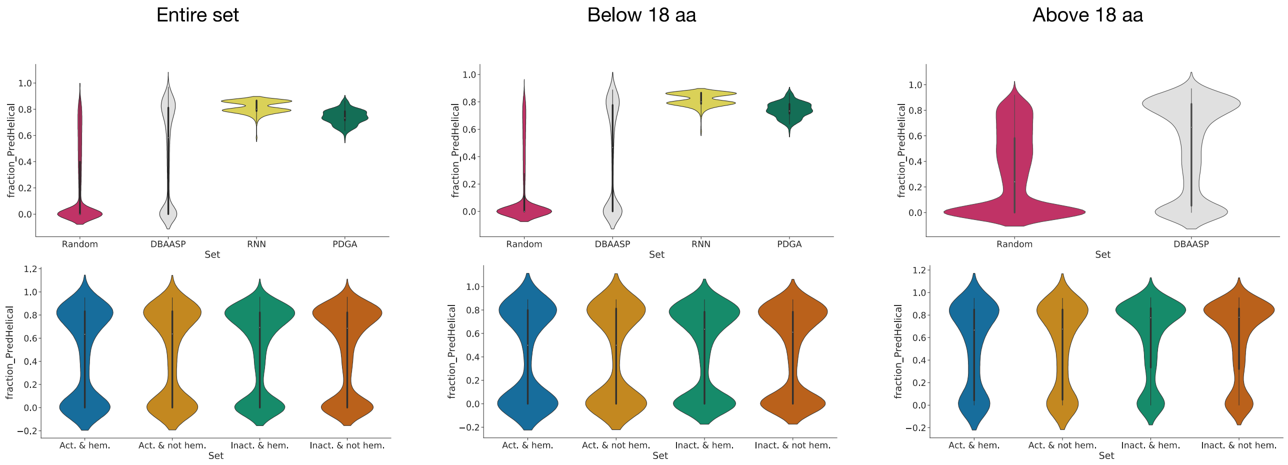

Fig. S3. Violin plots showing the distribution of helicity predicted with SPIDER3[1] in the random, DBAASP, RNN and PDGA sets and the active & hemolytic, active & not hemolytic, inactive & hemolytic, and inactive & not hemolytic subsets.

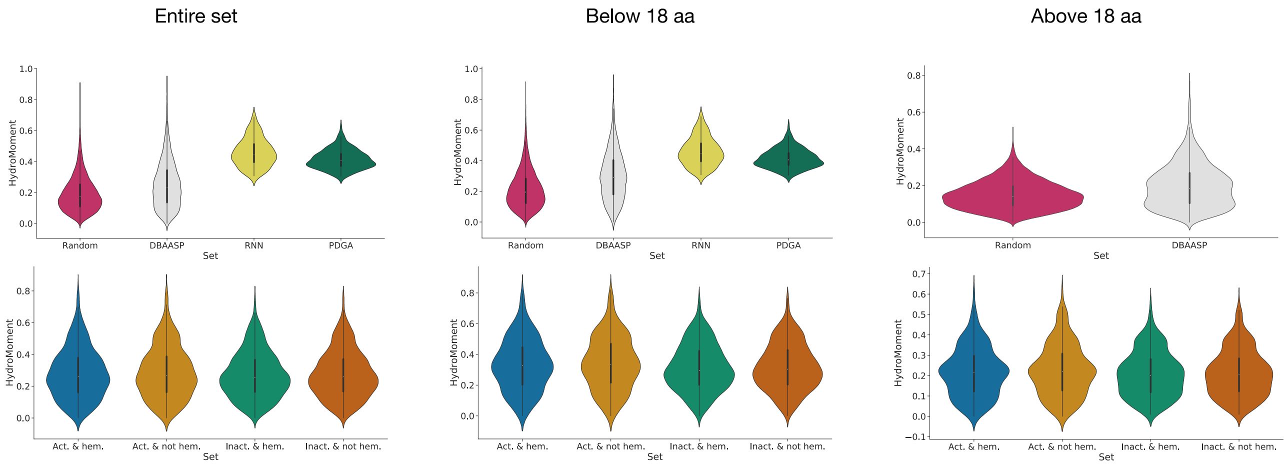

Fig. S4. Violin plots showing the distribution of the hydrophobic moment calculated according to Eisenberg *et al*.[2] in the random, DBAASP, RNN and PDGA sets and the active & hemolytic, active & not hemolytic, inactive & hemolytic, and inactive & not hemolytic subsets.

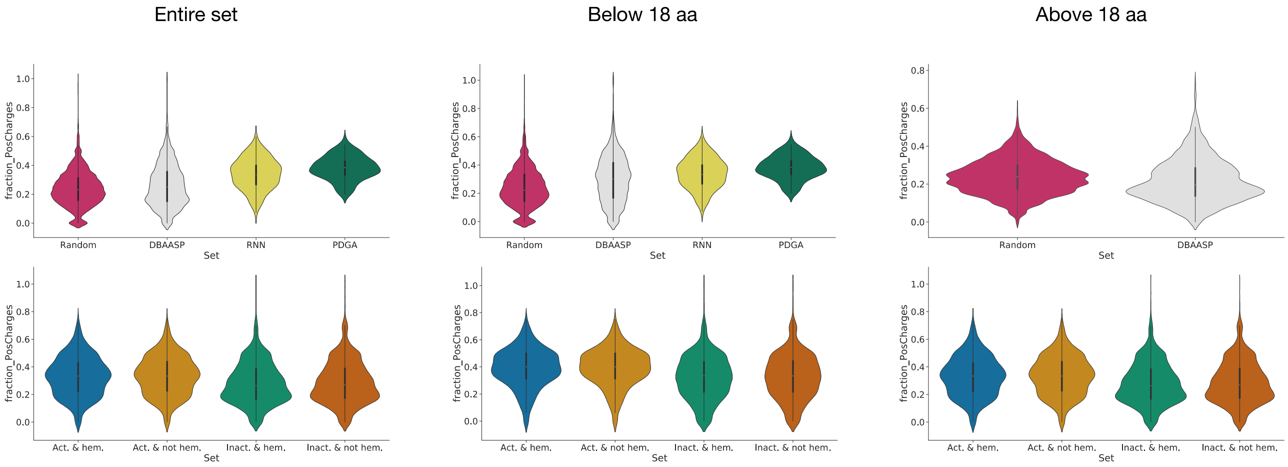

Fig. S5. Violin plots showing the distribution of the calculated fraction of positive charges in the random, DBAASP, RNN and PDGA sets and the active & hemolytic, active & not hemolytic, inactive & hemolytic, and inactive & not hemolytic subsets.

Random Lasioglossin mutants:

A random sequence mutator was built with following parameters:

Type of modification	Chances of occurring	Description
Mutation	0.50 (50% of the cases)	If a mutation occurs, a random number (between 1 and 7) of amino acids is exchanged for another amino acid
Insertion	0.25 (25% of the cases)	If an insertion occurs, a random number (between 1 and 5) of amino acids is inserted at a random position in the sequence
Deletion	0.25 (25% of the cases)	If a deletion occurs, a random number (between 1 and 5) of amino acids is removed from the sequence

100’000 sequences were sampled by applying the parameters described above to the sequence of Lasioglossin III. 89’621 (approx. 90%) sequences were unique. Out of the unique sequences, 807 (0.9% of the unique set) were predicted to be both, active and not hemolytic. Levenshtein distance towards Lasioglossin III, predicted fraction of helical residues and hydrophobic moment were calculated for each of the 807 remaining sequences.

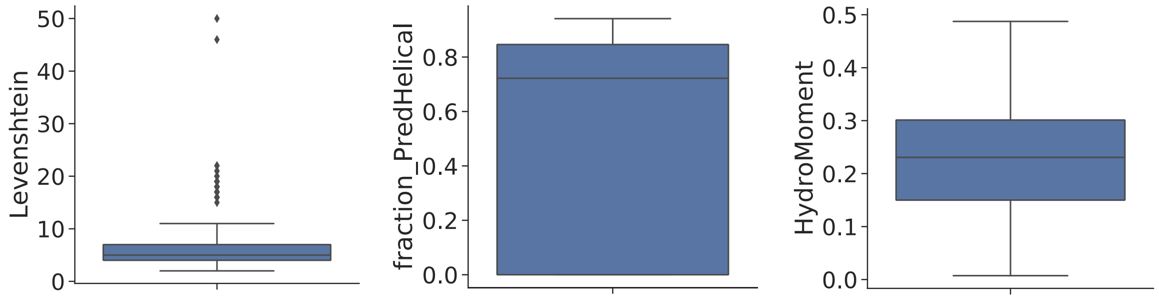

Fig. S6. Distributions of Levenshtein distance towards Lasioglossin III, predicted fraction of helical residues and calculated hydrophobic moment of a set of random mutants of Lasioglossin III. The set is composed of all sequences contained in a population of 100’000 randomly sampled mutants that were unique and passed activity and hemolysis classifier.

A total of 13 sequences (0.015%) remained after applying the additional selection filters:

- Levenshtein distance towards Lasioglossin III between 5 and 8 (excluding these values)
- Predicted fraction of helical residues >= 0.31
- Calculated hydrophobic moment >= 0.81

These results indicate that the performance of random mutation (0.015%) is comparable to the one from the proposed genetic algorithm (0.02%).

1.2.Experimental data

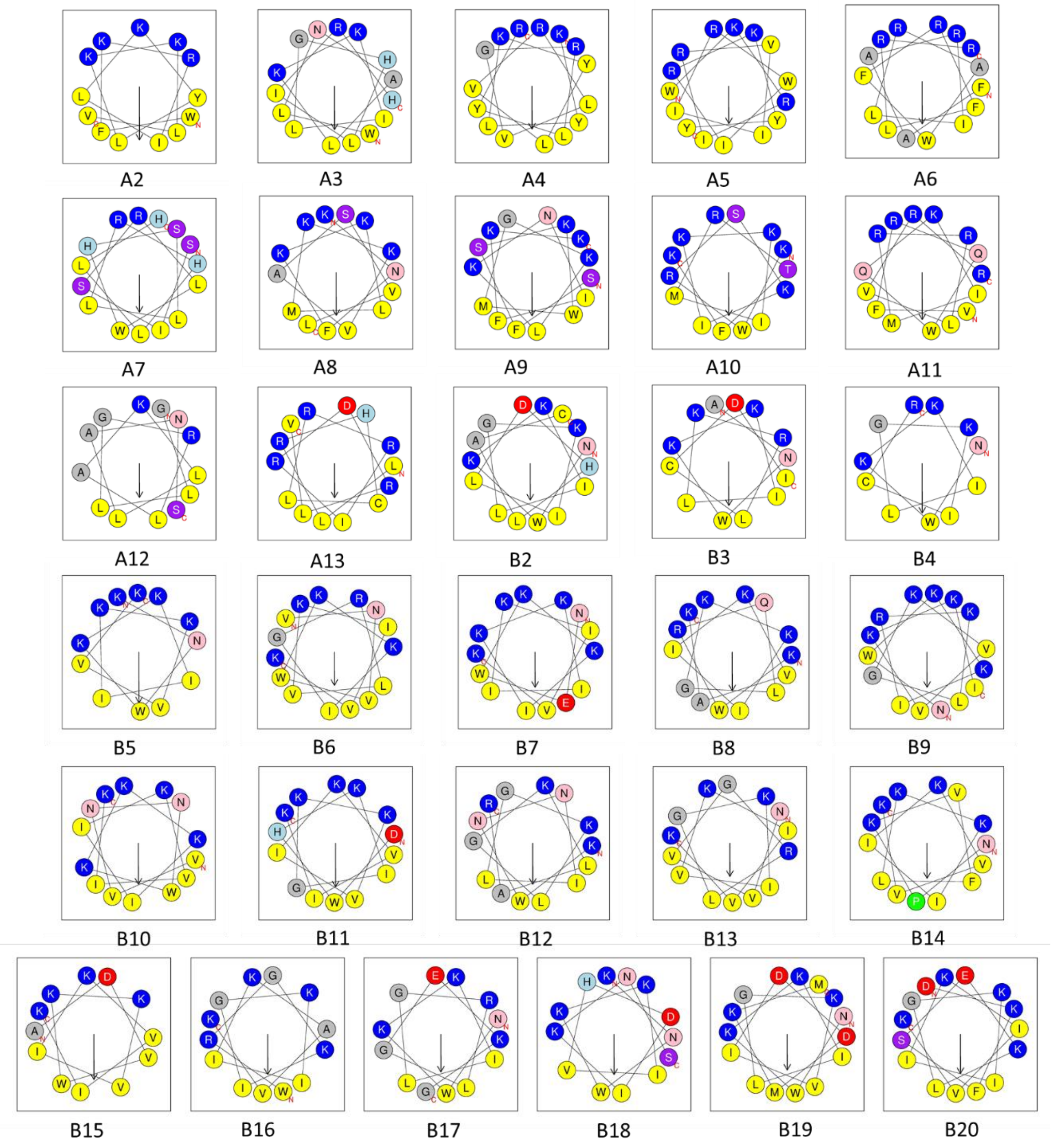

Fig. S7. Helix wheels predicted by HeliQuest.[3] Circle size proportional to side-chain size, blue indicates cationic residues, red indicates anionic residues, yellow indicates hydrophobic residues, grey indicates alanine and glycine, pink indicates asparagine, light blue indicates histidine. Arrows represent the helical hydrophobic moment.

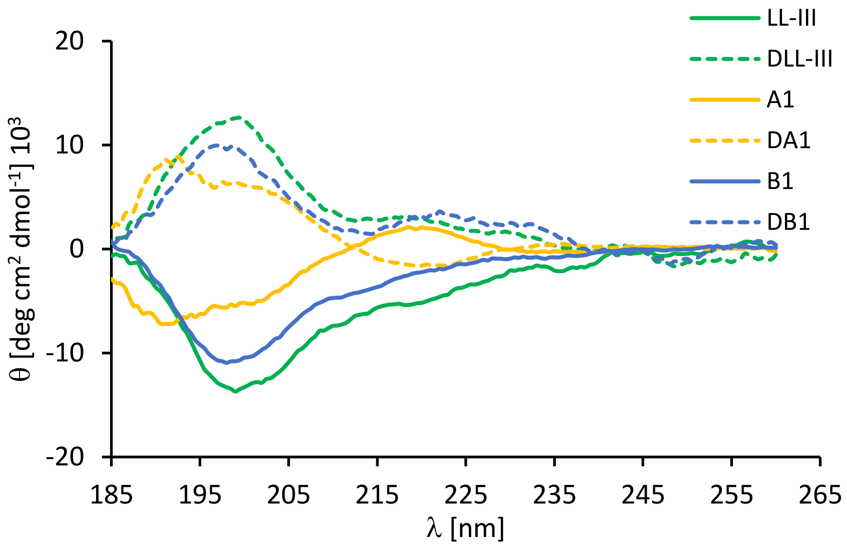

Fig. S8. CD spectra of hit peptides (100 μg/mL) in 10 mM phosphate buffer pH 7.4.

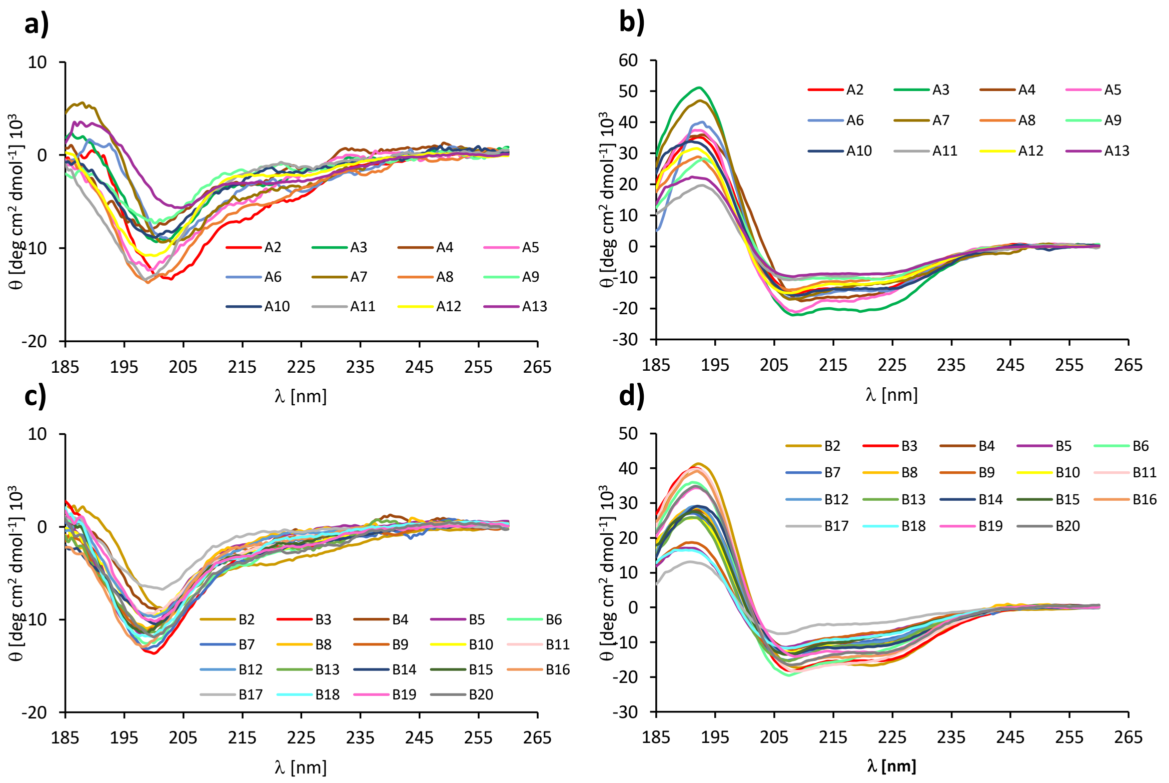

Fig. S9. CD spectra of A2-A13 (**a**), B2-B20 (**c**) at 100 μg/mL in 10 mM phosphate buffer pH 7.4 and (**b**), (**d**) in a presence of 5 mM DPC.

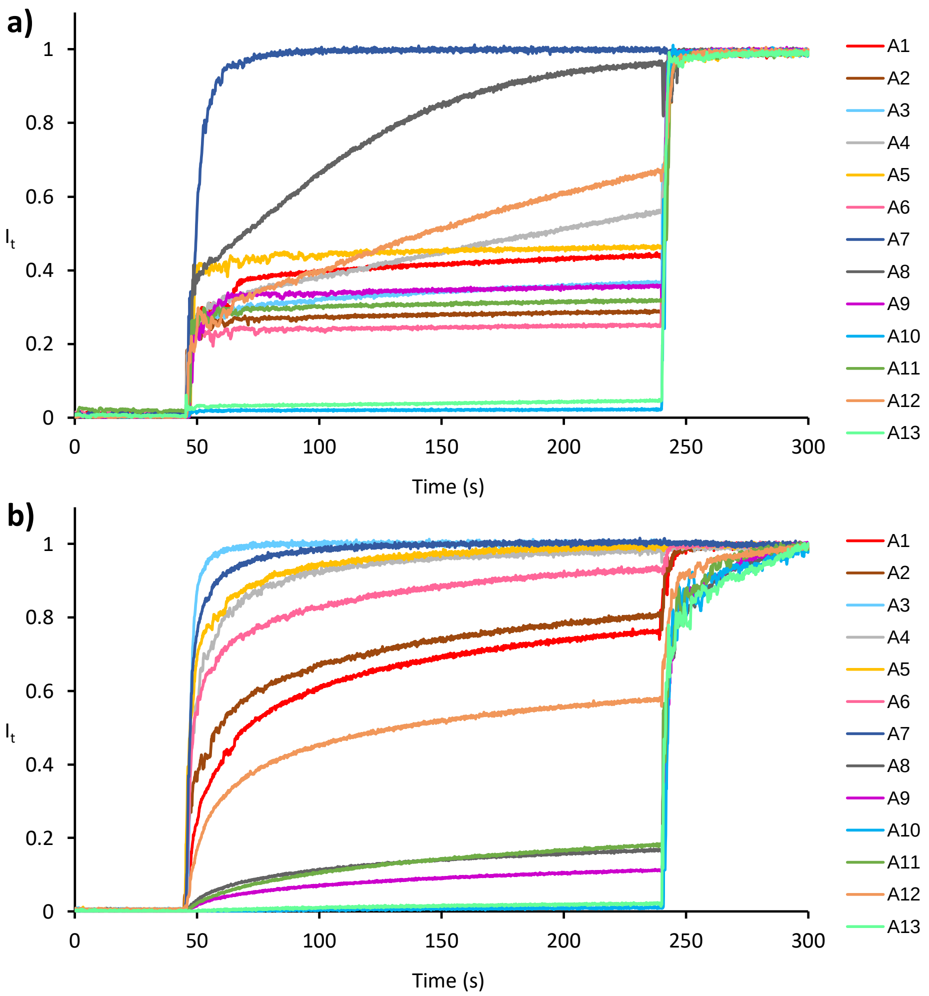

Fig. S9. (**a**) Vesicle leakage experiment of A1-A13 using 5(6)-carboxyfluorescein, at 10 µg/mL. Fluorescein leakage assay from egg yolk phosphatidyl glycerol (PG) lipid vesicles. (**b**) Fluorescein leakage assay from egg yolk phosphatidyl choline (PC) lipid vesicles. Vesicles were suspended in buffer (10 mM TRIS, 107 mM NaCl, pH 7.4) and compounds were added after 45 sec. After 240 seconds 1.2% Triton X-100 was added for full release of fluorescein.

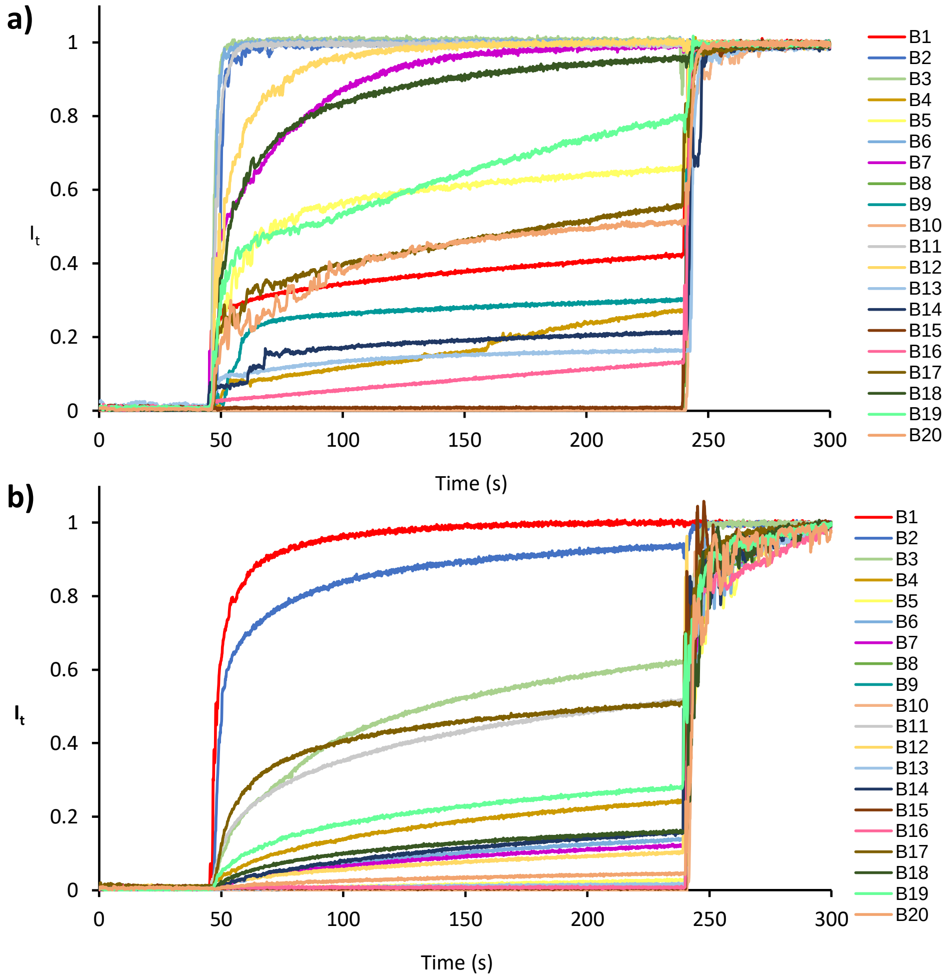

Fig. S10. (**a**) Vesicle leakage experiment of B1-B20 using 5(6)-carboxyfluorescein, at 10 µg/mL. Fluorescein leakage assay from egg yolk phosphatidyl glycerol (PG) lipid vesicles. (**b**) Fluorescein leakage assay from egg yolk phosphatidyl choline (PC) lipid vesicles. Vesicles were suspended in buffer (10 mM TRIS, 107 mM NaCl, pH 7.4) and compounds were added after 45 sec. After 240 seconds 1.2% Triton X-100 was added for full release of fluorescein.

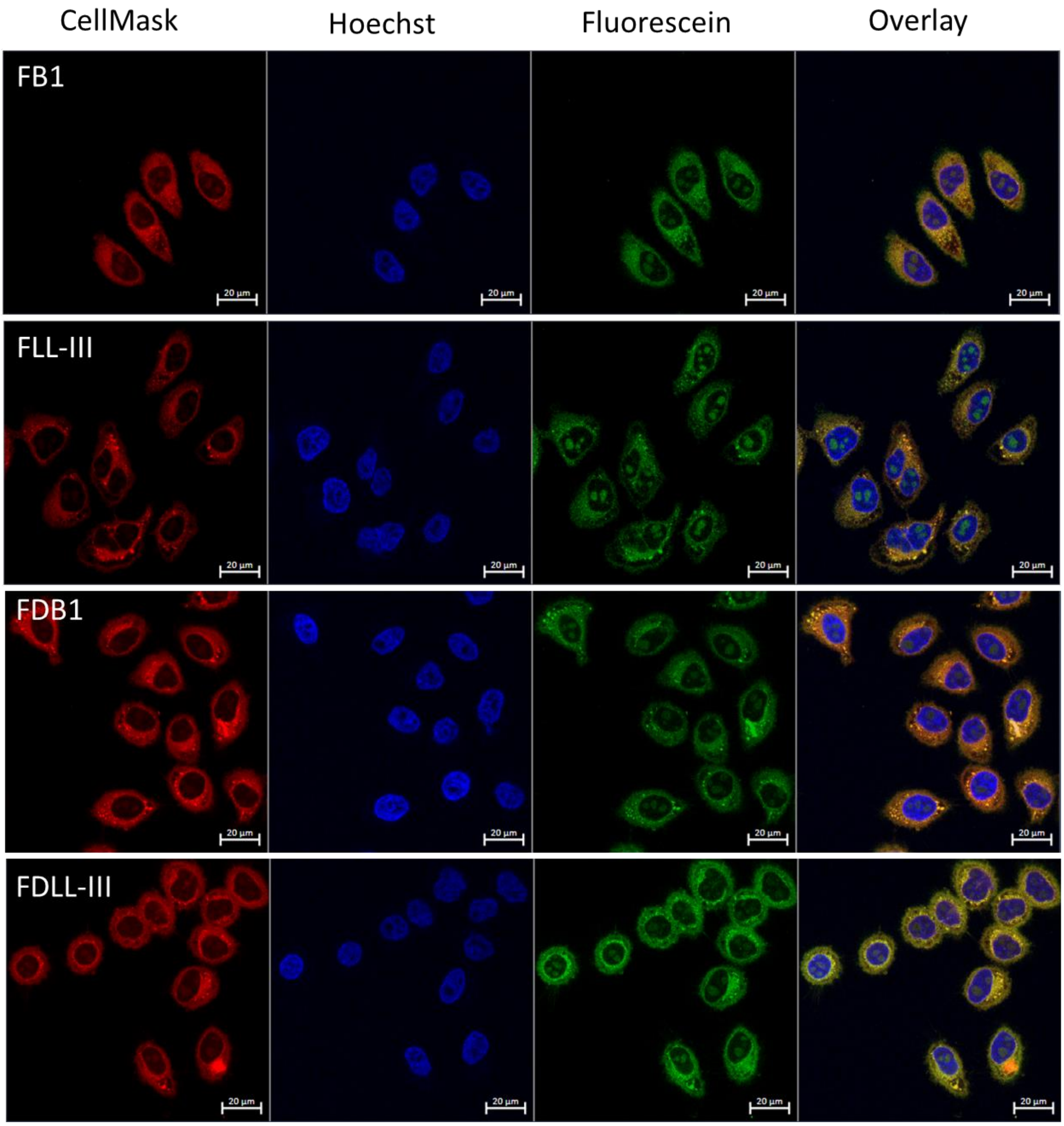

Fig. S11. Confocal microscopy of HeLa cells, incubated for 2 h with 10 µM fluorescein-labelled ACPs; Cell plasma membrane is in red (CellMask), Nucleus is in blue (Hoechst33258), compounds are in green (Fluorescein). Scale bar 20 µm.

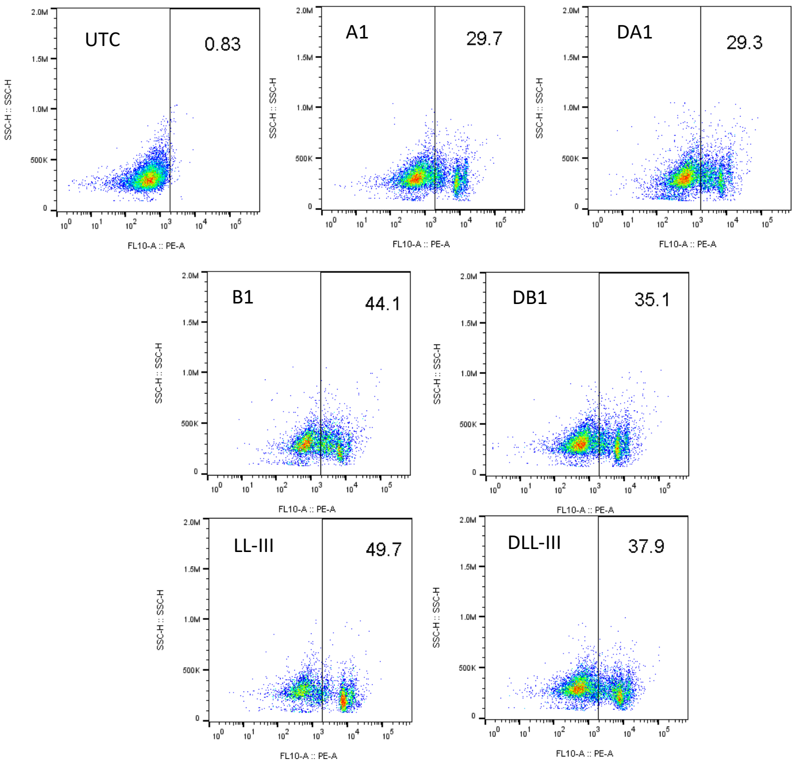

Fig. S12. Propidium Iodine (PI) entrance to HeLa cells, treated by 10 μM of peptides and incubated for 10 min, was detected by FACS.

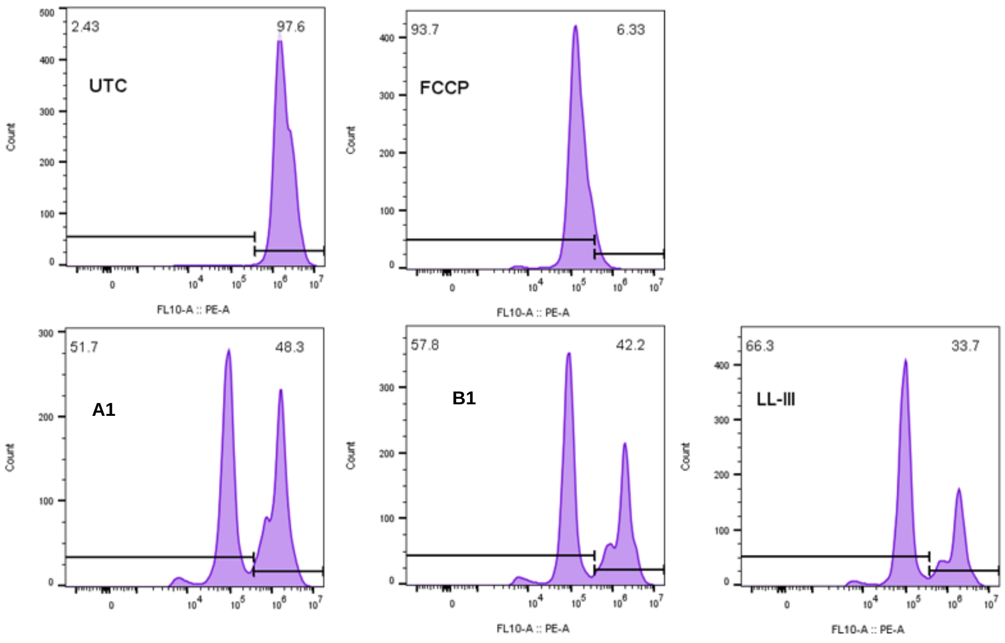

Fig. S13. Mitochondrial Membrane Potential (MMP) depolarization as detected by FACS. HeLa cells were treated by 15 μM of ACPs and incubated for 15 min. UTC – untreated cells, Carbonyl cyanide-p-trifluoromethoxyphenylhydrazone (FCCP) 50 μM was used as a positive control.

Table S2. Extended IC_{50} profiling

nr.	Sequence	HeLa[a]	MCF-7[a]	MB-MDA-231[a]	MCF-10a[b]	HEK293[a]
A1	FAKKFFKKFAKFAFK	8.2±0.5	6.0 ±0.5	6.4±0.5	17.3±3.5	15.2±0.7
B1	ANWKKWIGKVIKLVK	5.5±0.8	6.1±1.0	5.0±0.6	19.5±2.8	12.1±1.6
B2	NWKKILGKILDHLAC	7.0±1.4	5.4±0.3	5.4±1.2	11.7±0.4	6.7±0.5
LL-III[a]	VNWKKILGKIIKVVK	6.0±0.5	7.1±0.3	5.9±0.4	14.8±2.1	15.0±3.0

[a] IC_{50} was determined after 72 h incubation at 37°C in DMEM high glucose medium supplemented with 10 % FBS. [b] IC_{50} was determined after 72 h incubation at 37°C in serum-free HUMEC ready medium.

2. Materials and Reagents

All reagents, salts, and buffers were used as purchased from commercial suppliers: Sigma Aldrich, Fluorochem Ltd, Iris Biotech Gmbh, TCI (Tokyo Chemical Company), Space Peptides Pharmaceutical AG. Amino acids were used as the following derivatives: Fmoc-Ala-OH, Fmoc-Arg(Pbf)-OH, Fmoc-Asn(Trt)-OH, Fmoc-Asp(OtBu)-OH, Fmoc-Cys(Trt)-OH, Fmoc-Gln(Trt)-OH, Fmoc-Glu(OtBu)-OH, Fmoc-Gly-OH, Fmoc-His(Trt)-OH, Fmoc-Ile-OH, Fmoc-Leu-OH, Fmoc-Lys(Boc)-OH, Fmoc-Met-OH, Fmoc-Phe-OH, Fmoc-Ser(tBu)-OH, Fmoc-Thr(tBu)-OH, Fmoc-Trp(Boc)-OH, Fmoc-Tyr(tBu)-OH, Fmoc-Val-OH. Rink Amide AM LL resin was purchased from Novabiochem (loading: 0.29 mmol·g^{-1}).

Materials for biological assays: 0.05% Trypsin-EDTA, FluoroBrite DMEM were purchased from Gibso, Thermo Fisher Scientific (Reinach, CH). Hoechst 33258, Mitotracker Red, Propidium Iodine staining solution were purchased from Invitrogen, Thermo Fisher Scientific (Reinach, CH). Dulbecco's modified Eagle medium (DMEM) high glucose, Poly L-Lysine, Fetal Bovine Serum (FBS), AlamarBlue® were purchased from Sigma Aldrich (Buchs, CH). TMRE-Mitochondrial Membrane Potential Assay Kit was purchased from Abcam (Cambridge, UK).

Analytical RP-HPLC was performed with an Ultimate 3000 Rapid Separation LC-MS System (DAD-3000RS diode array detector) using an Acclaim RSLC 120 C18 column (2.2 µm, 120 Å, 3×50 mm, flow 1.2 mL/min) from Dionex. Data recording and processing were done with Dionex Chromeleon Management System Version 6.80 (analytical RP-HPLC). All RP-HPLC were using HPLC-grade acetonitrile and Milli-Q deionized water. The elution solutions were: A Milli-Q deionized water containing 0.05% TFA; D Milli-Q deionized water/acetonitrile (10:90, v/v) containing 0.05% TFA. Preparative RP-HPLC was performed with a Waters automatic Prep LC Controller System containing the four following modules: Waters2489 UV/Vis detector, Waters2545 pump, Waters Fraction Collector III, and Waters2707 Autosampler. A Dr. Maisch GmbH Reprospher column (C18-DE, 100×30 mm, particlesize 5 µm, pore size 100 Å, flow rate 40 mL/min) was used. Compounds were detected by UV absorption at 214 nm using a Waters 248 Tunable Absorbance Detector. Data recording and processing were performed with Waters ChromScope version 1.40 from Waters Corporation. All RP-HPLC were performed using HPLC-grade acetonitrile and Milli-Q deionized water. The elution solutions were: A: Milli-Q deionized water containing 0.1% TFA; D: Milli-Q deionized water/acetonitrile (10/90, v/v) containing 0.1% TFA. MS spectra were recorded on a Thermo Scientific LTQ OrbitrapXL. HRMS spectra were provided by the MS analytical service of the Department of Chemistry and Biochemistry at the University of Bern (group PD Dr. Stefan Schürch).

3. Solid-phase peptide synthesis

3.1 General Information

Linear peptides were synthesized manually using 150-300 mg of Rink Amide AM LL resin (0.29 mmol/g) by standard 9-fuorenylmethoxycarbonyl (Fmoc) Solid Phase Peptide Synthesis at 60°C under nitrogen bubbling. The resin was swollen in DMF for 10 min. Double deprotection of the Fmoc group was performed using a solution containing 5% w/v piperazine, 2% v/v 1,8-diazabicyclo(5.4.0)undec-7-ene DBU, 10% v/v of 2-butanol in DMF during 1 min and 4 min respectively. The resin was washed with DMF-(5×8 mL DMF) after deprotection. Coupling step (2×8 min) was performed with 3 mL of amino acid (0.2 M), 2 mL of DIC (0.8 M) and 1.5 mL of Oxyma (0.8 M) in DMF. Resin was washed with DMF between couplings (2×8 mL) and after second coupling (3×8 mL).

For sequences containing aspartic or glutamic acid deprotection solution was exchanged to 20% v/v piperidine + 0.7% v/v formic acid in DMF to avoid aspartimide, glutamide and side products formation.

The cleavage from resin was carried out by treating the resins with 7 mL of a TFA/TIS/DODT/H_2O (95/2/2/1, v/v/v/v) solution for 3 h. The peptide solutions were precipitated with 30 mL of cold terbutylmethyl ether (TBME), centrifuged for 10 min at 3500 rpm (twice), evaporated and dried in high vacuum for 60 min. The crude was then dissolved in a H_2O/CH_3CN (10/1, v/v) mixture, some drops of MeOH added when needed and purified by preparative RP-HPLC. The fractions of the crudes were then lyophilized. Yields are given as SPPS total yields. In all cases, yields are calculated for the corresponding TFA salts.

3.2 Fluorescein-labelled peptides

The synthesis was carried out manually in a polypropylene syringe, fitted with a polypropylene frit, a teflon stopcock and a stopper. Last amino acid was deprotected to have free N-terminus. Coupling of 5/6-carboxyfluorescein (5/6-CF) was performed by using 5/6-CF (7 eq., relative to resin loading), HOBt (7 eq, relative to resin loading) and DIC (7 eq., relative to resin loading) in DMF (5 ml). The resin was stirred overnight and protected from light by covering the syringe with aluminium foil. The next day, the resin was washed with DMF, MeOH and DCM (3×4 mL each). A solution of 20% piperidine in DMF (8×5 mL, until the supernatant was colorless) was added to the resin to remove the excess of free 5/6-CF before the resin was finally washed with DCM (5×4 mL).

4. Cell culture conditions

HeLa, HEK-293, MCF-7, MDA-MB-231 cells (ATCC, Manassas, USA) were cultured and maintained in DMEM high glucose (Dulbecco`s modified Eagle medium, Sigma Aldrich) medium, supplemented with 10% FBS (Sigma Aldrich) and 1% penicillin/streptomycin. MCF-10a cells (ATCC, Manassas, USA) were cultured and maintained in HuMEC ready medium (Thermo Fisher Scientific), contained epidermal growth factor, hydrocortisone, isoproterenol, transferrin, insulin, and bovine pituitary extract. The cells were handled and subcultured according to the manufacturer instructions. Cells were incubated in a humidified incubator at 37°C in the presence of 5% CO_2.

5. HPLC/MS and HRMS spectra

A1 (FAKKFFKKFAKFAFK-NH_2) was obtained after manual synthesis from Rink Amide AM resin LL (150 mg, 0.29 mmol/g), the peptide was obtained as a white foamy solid after preparative RP-HPLC purification (20 mg, 18.0%). Analytical RP-HPLC: t_R = 1.40 min (100% A to 100% D in 3.5 min, λ= 214 nm). MS (ESI+): $C_{99}H_{144}N_{22}O_{15}$ calc./obs. 1881.12/1881.12 $[M]^+$

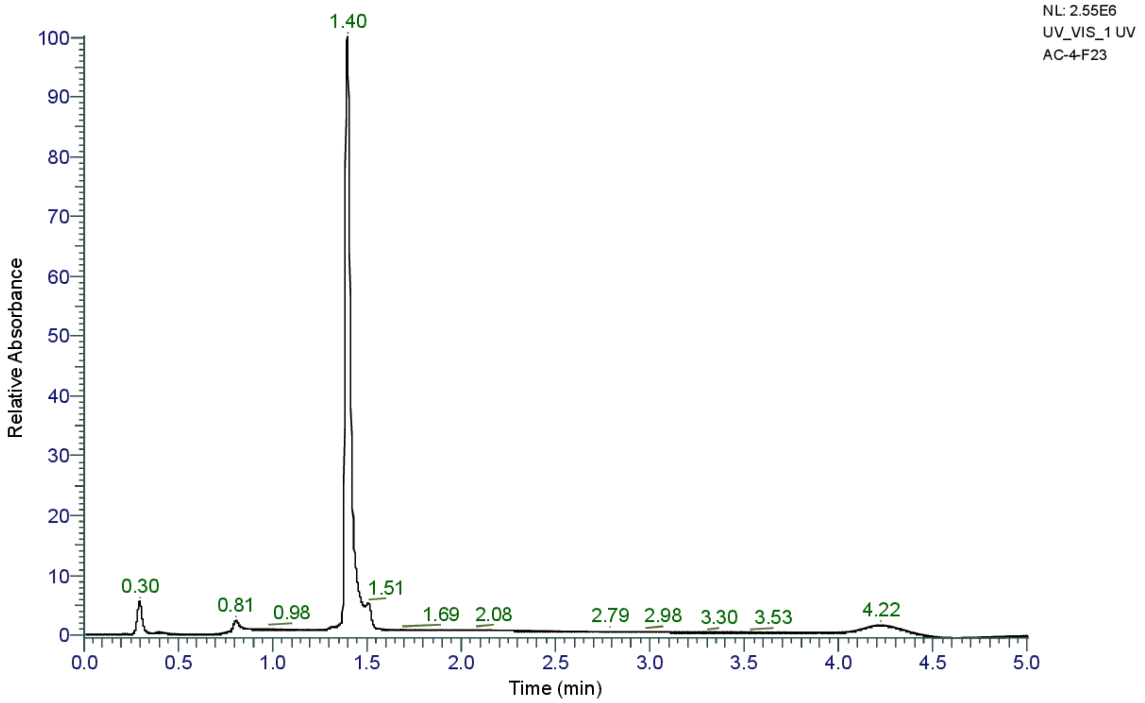

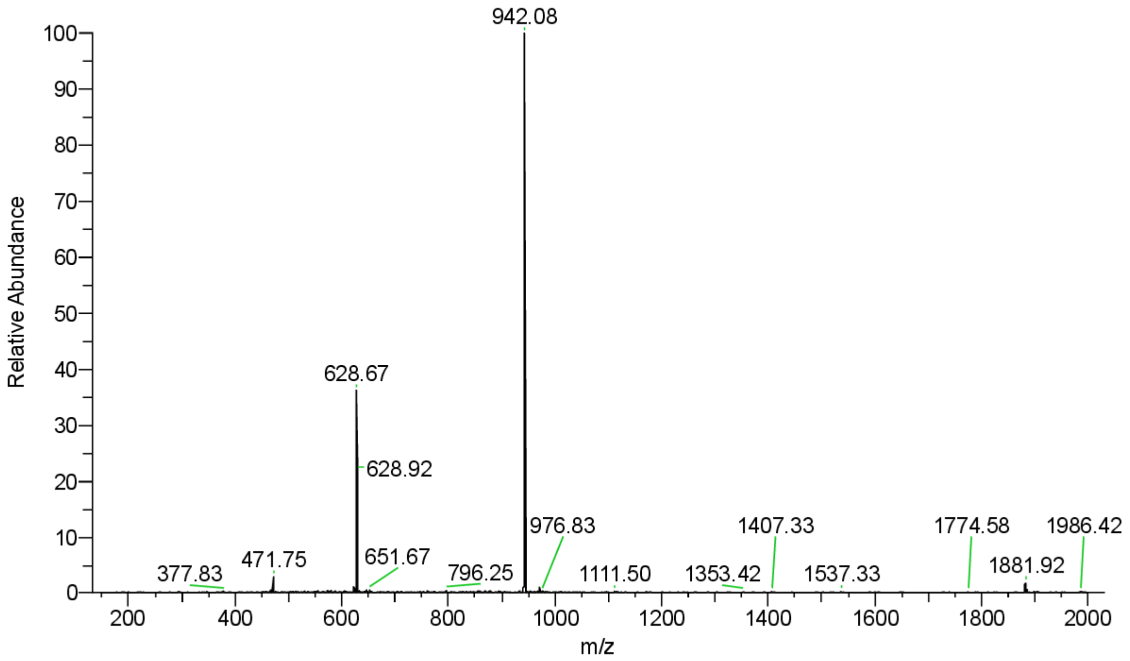

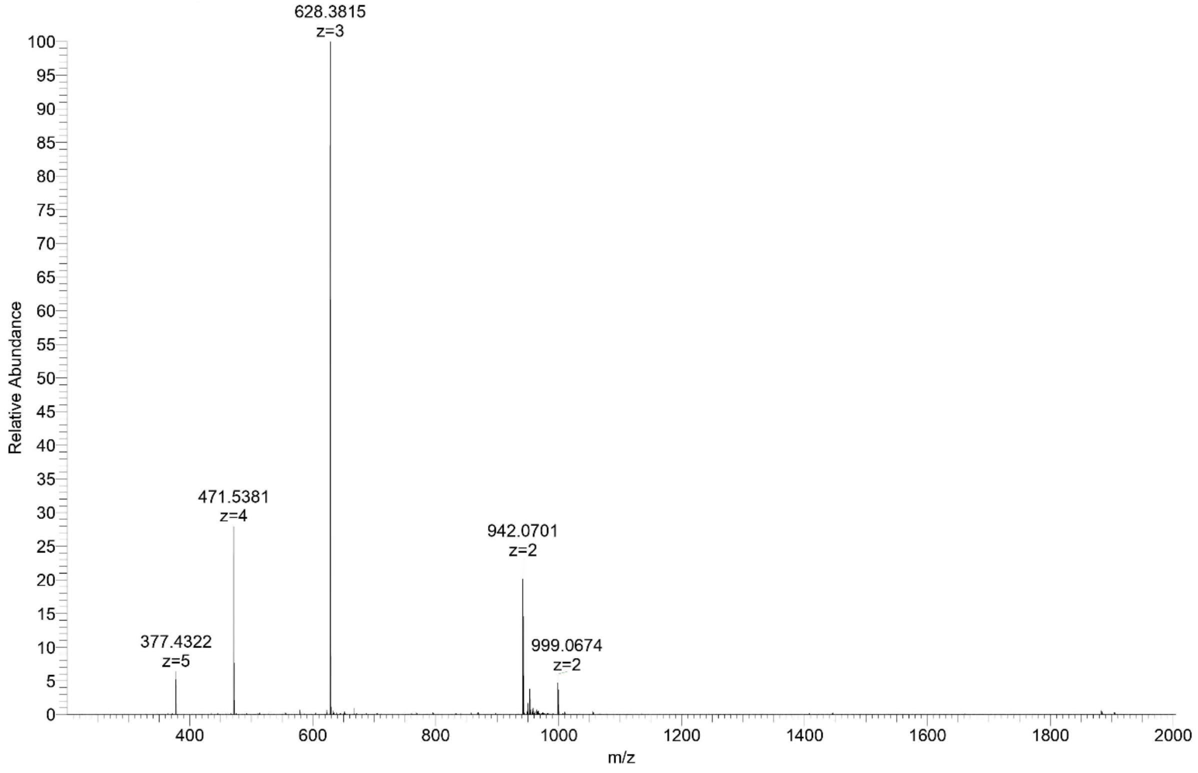
628.3815
z=3
471.5381
z=4
377.4322
z=5
942.0701
z=2
999.0674
z=2
Relative Abundance
m/z

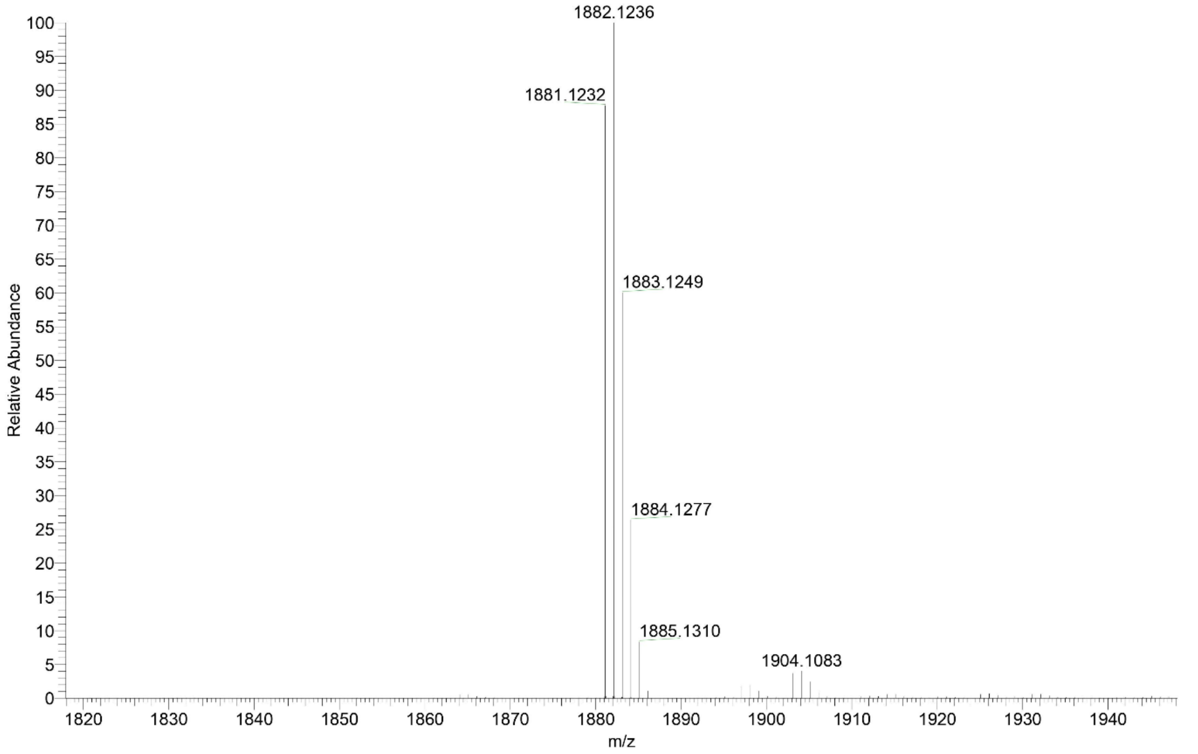
1882.1236
1881.1232
1883.1249
1884.1277
1885.1310
1904.1083
Relative Abundance
m/z

A2 (WFKRILKYLKKLV-NH_2) was obtained after manual synthesis from Rink Amide AM resin LL (150 mg, 0.29 mmol/g), the peptide was obtained as a white foamy solid after preparative RP-HPLC purification (14 mg, 14.0%). Analytical RP-HPLC: t_R = 1.60 min (100% A to 100% D in 3.5 min, λ= 214 nm). MS (ESI+): $C_{88}H_{144}N_{22}O_{14}$ calc./obs. 1733.12/1733.13 $[M]^+$

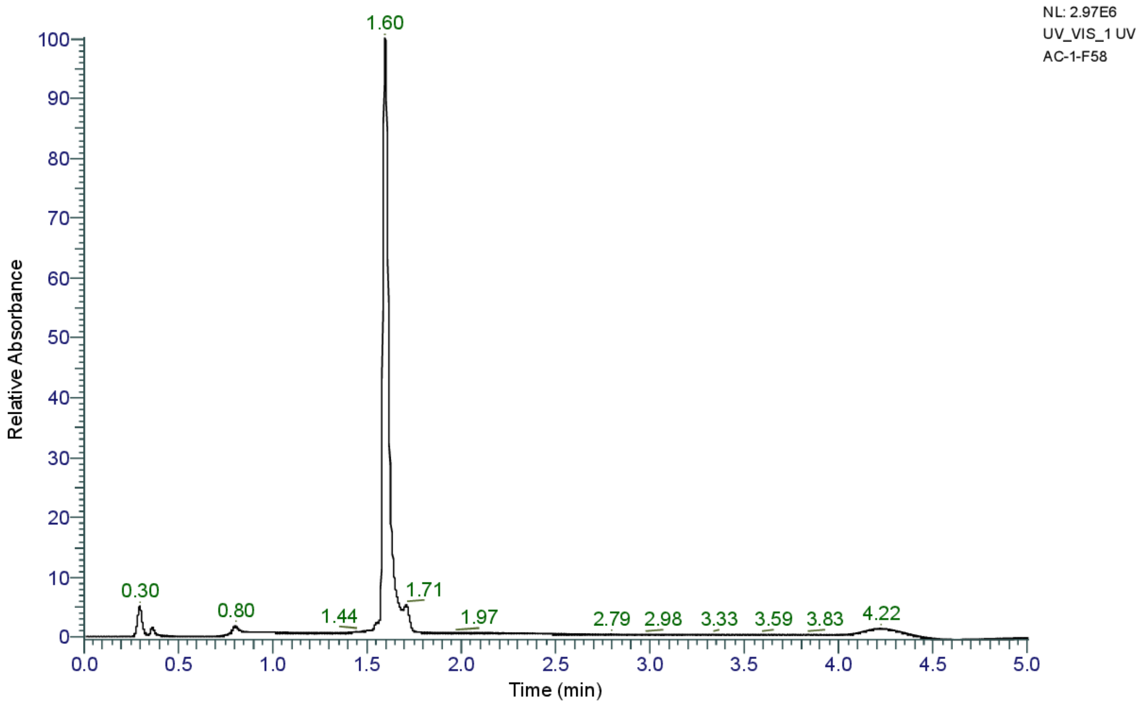

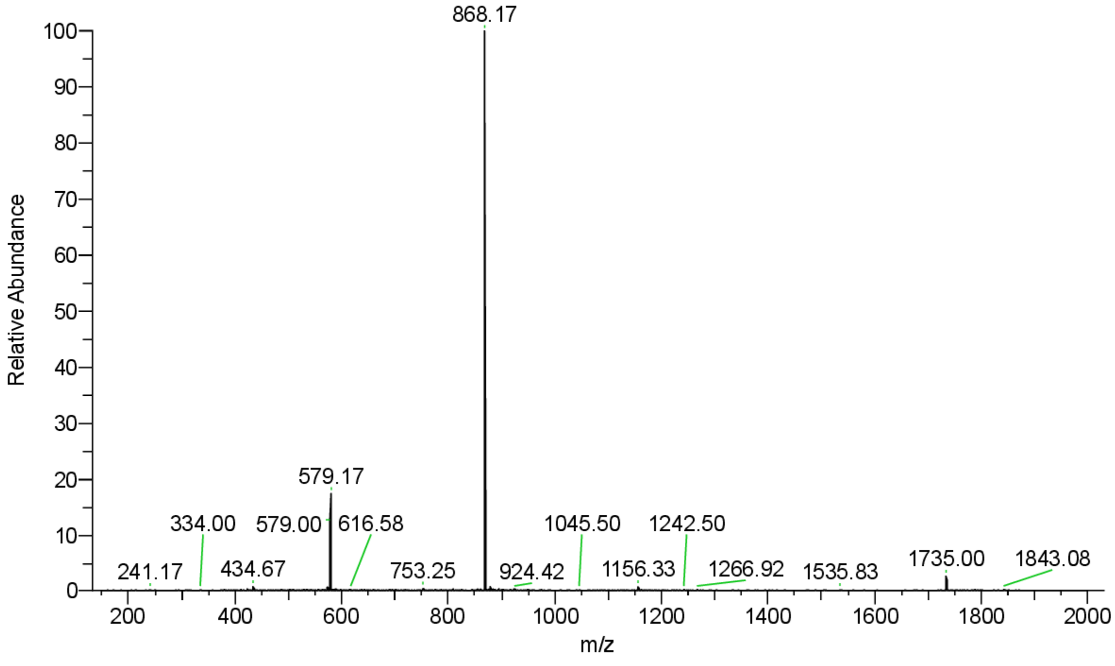

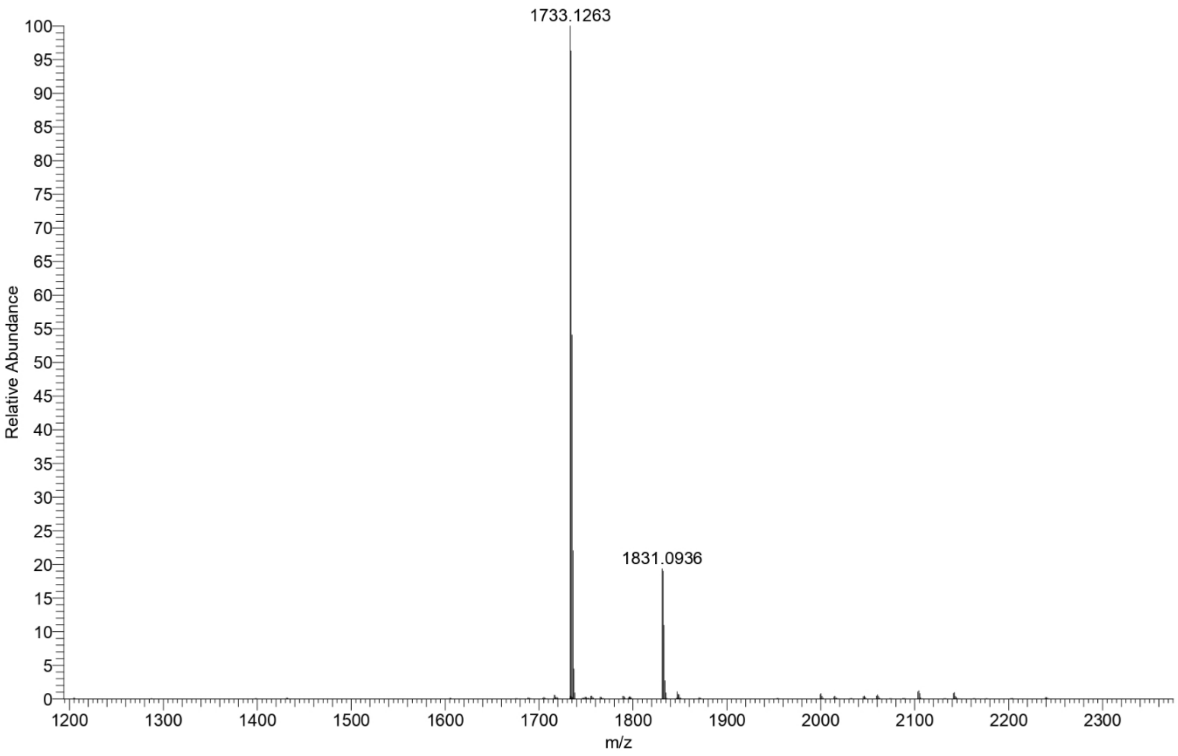
1733.1263
1831.0936
Relative Abundance
m/z

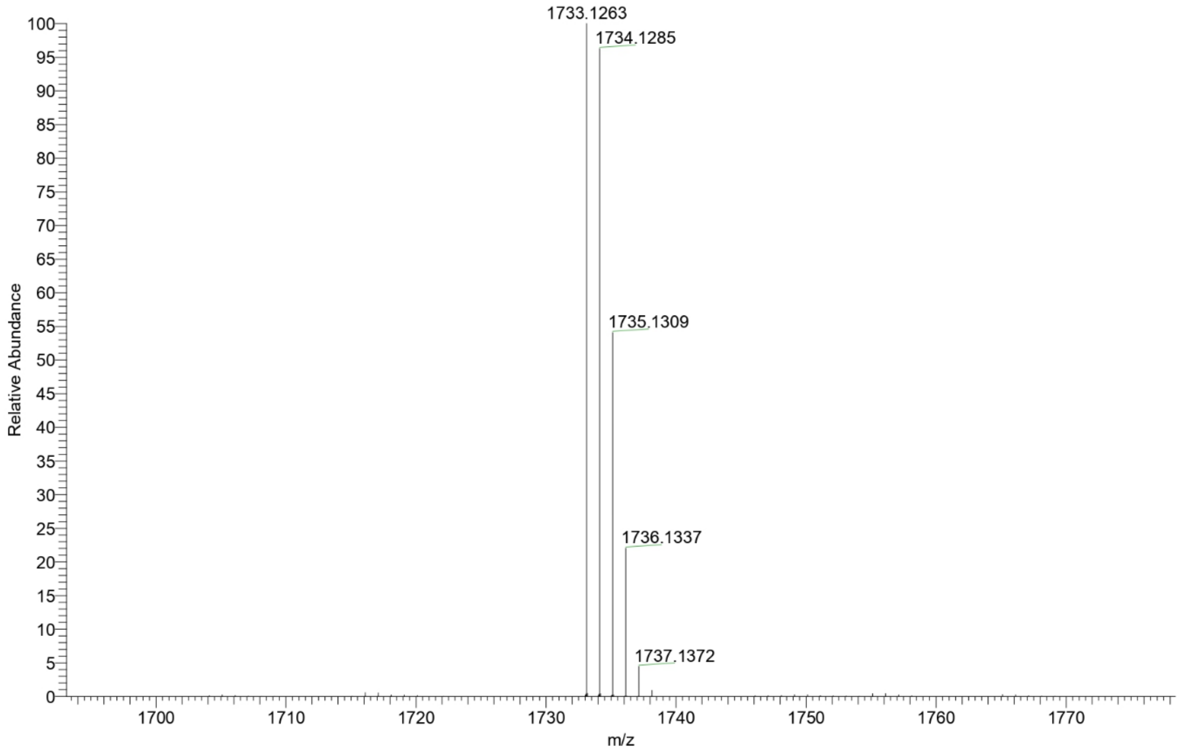
1733.1263
1734.1285
1735.1309
1736.1337
1737.1372
Relative Abundance
m/z

A3 (WLNALKKILGHLIRH-NH_2) was obtained after manual synthesis from Rink Amide AM resin LL (300 mg, 0.29 mmol/g), the peptide was obtained as a white foamy solid after preparative RP-HPLC purification (33 mg, 17.6%). Analytical RP-HPLC: t_R = 1.65 min (100% A to 100% D in 3.5 min, λ= 214 nm). MS (ESI+): $C_{86}H_{143}N_{27}O_{16}$ calc./obs. 1810.12/1810.12 $[M]^+$

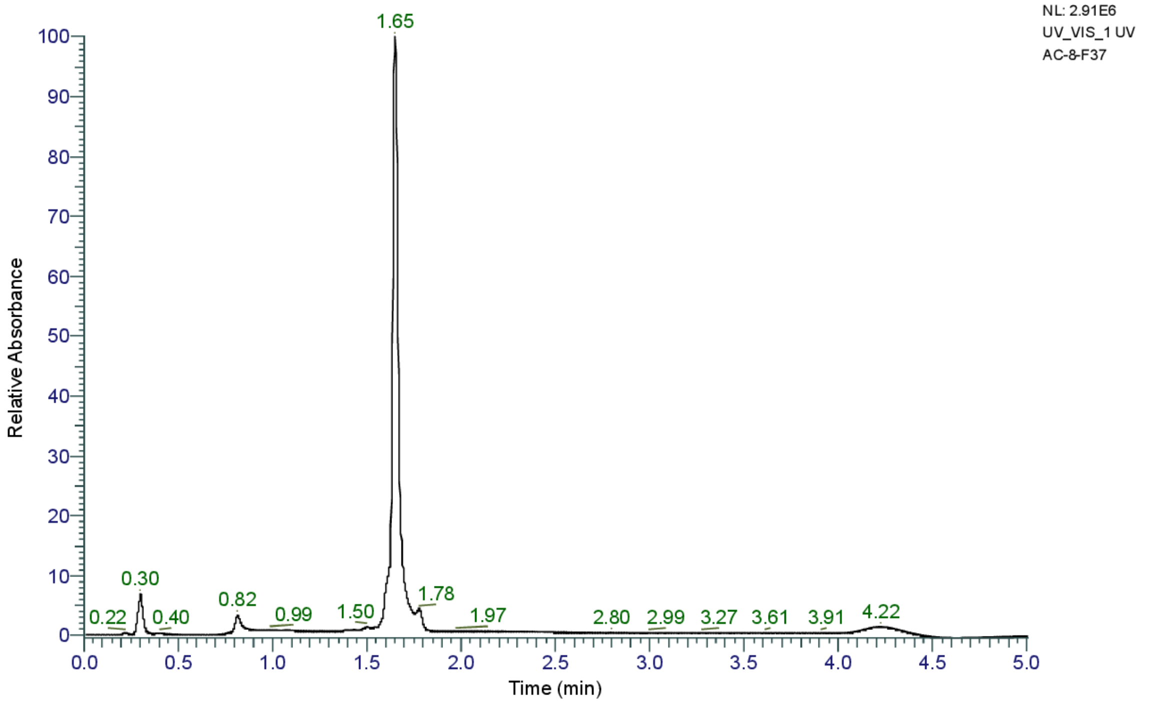

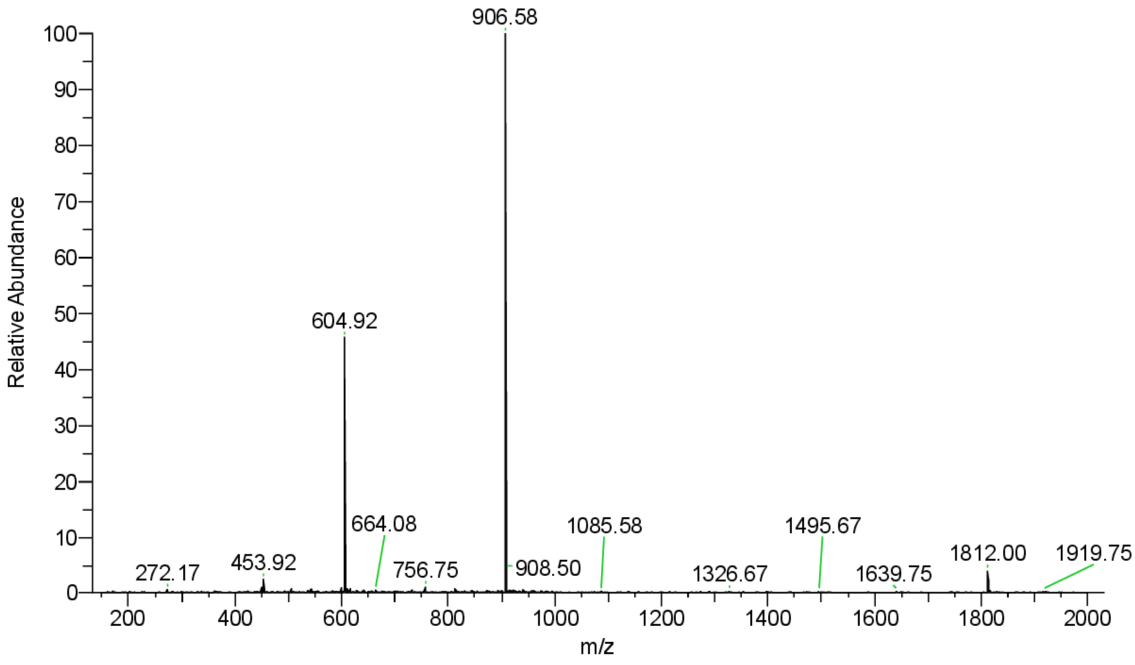

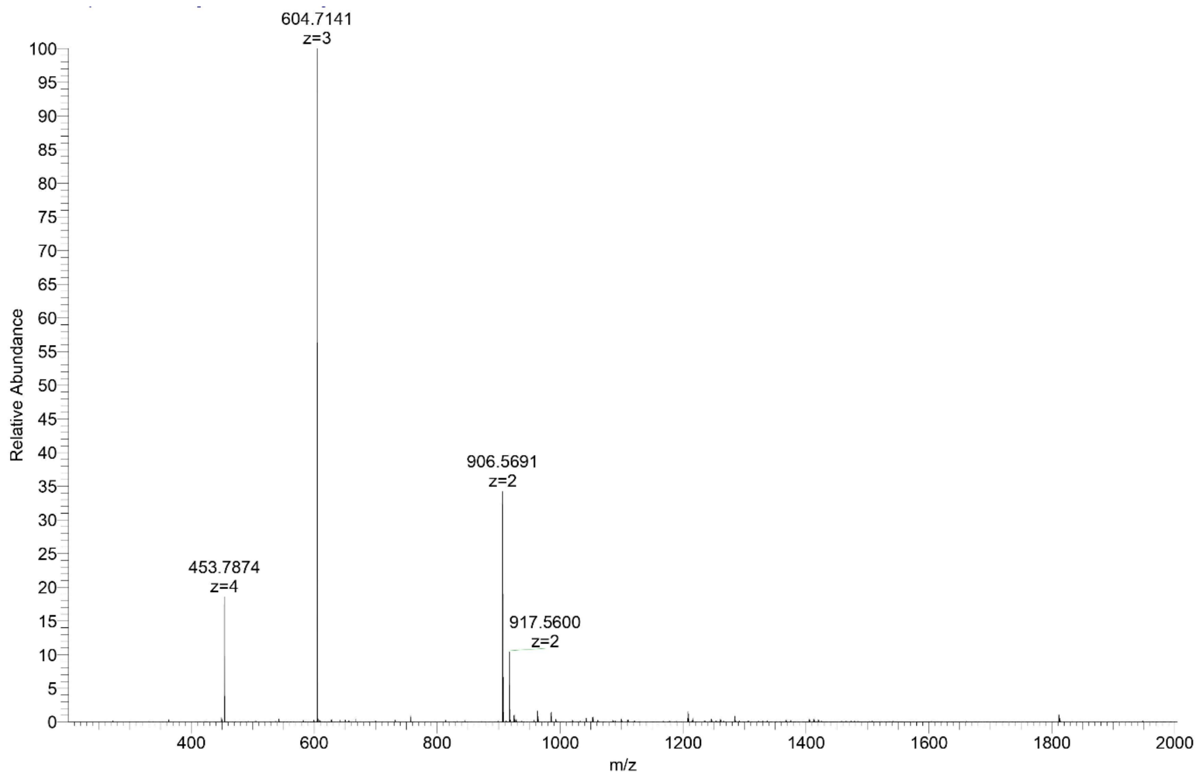
604.7141
z=3
906.5691
z=2
453.7874
z=4
917.5600
z=2
Relative Abundance
m/z

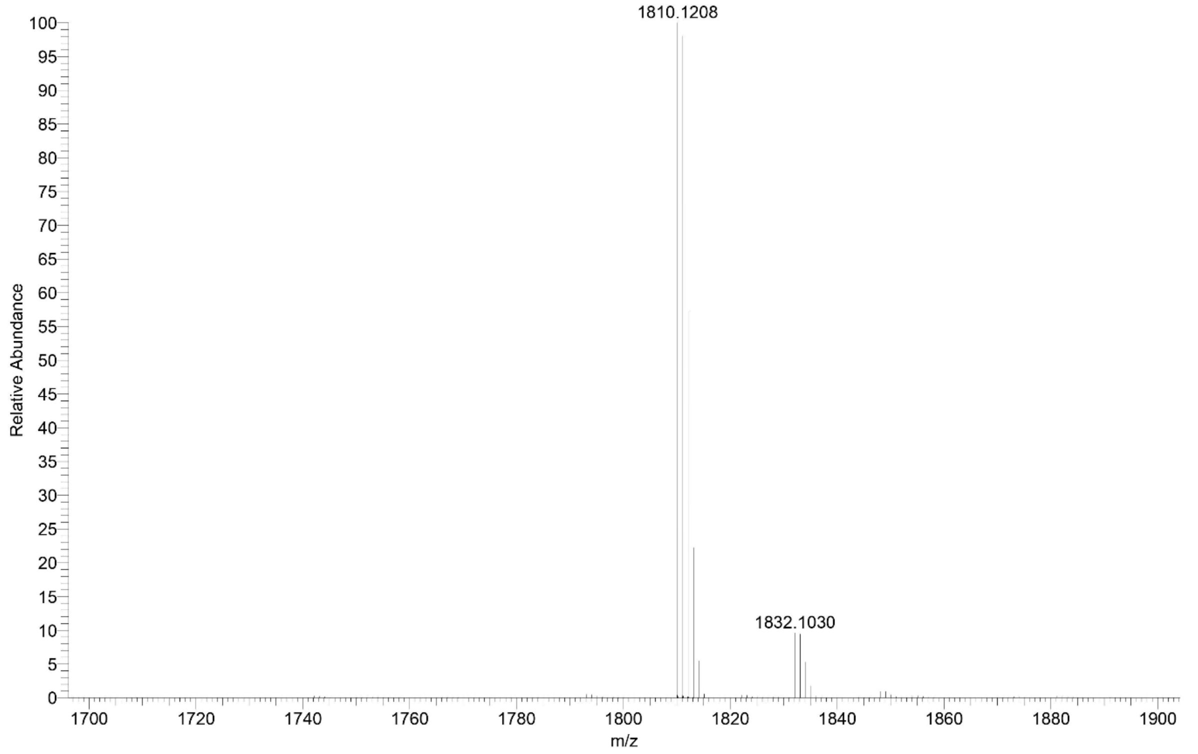
1810.1208
1832.1030
Relative Abundance
m/z

A4 (KYLKYLVRLVGRLYR-NH_2) was obtained after manual synthesis from Rink Amide AM resin LL (300 mg, 0.29 mmol/g), the peptide was obtained as a white foamy solid after preparative RP-HPLC purification (33 mg, 13.7%). Analytical RP-HPLC: t_R = 1.51 min (100% A to 100% D in 3.5 min, λ= 214 nm). MS (ESI+): $C_{93}H_{155}N_{27}O_{18}$ calc./obs. 1938.20/1938.20 $[M]^+$

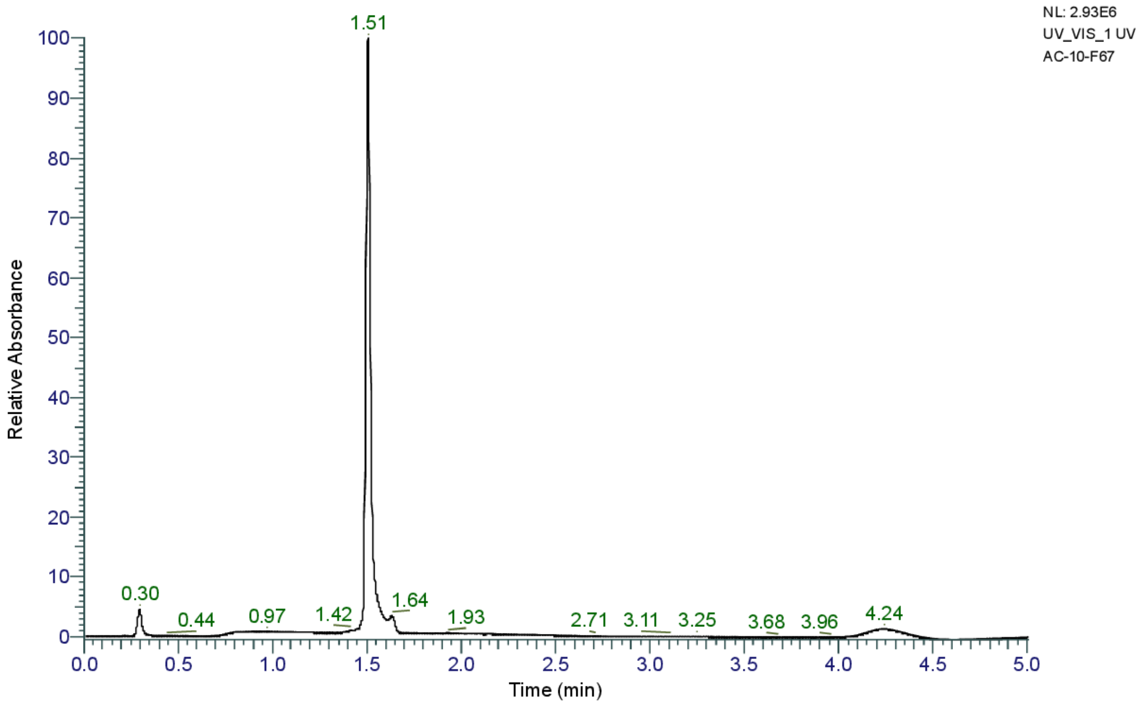

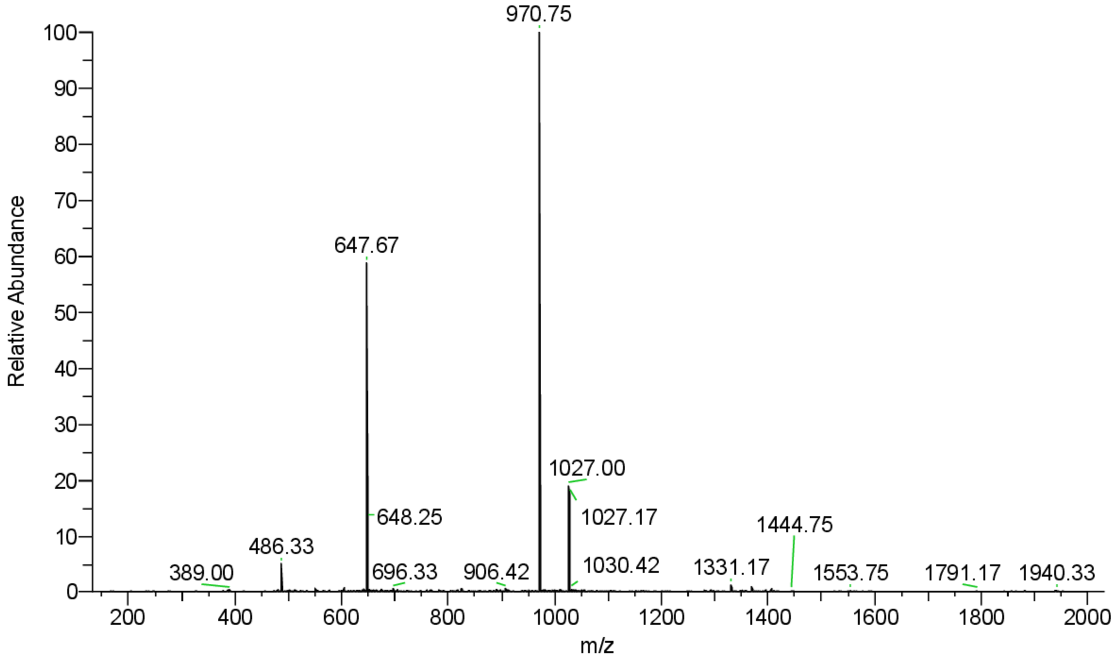

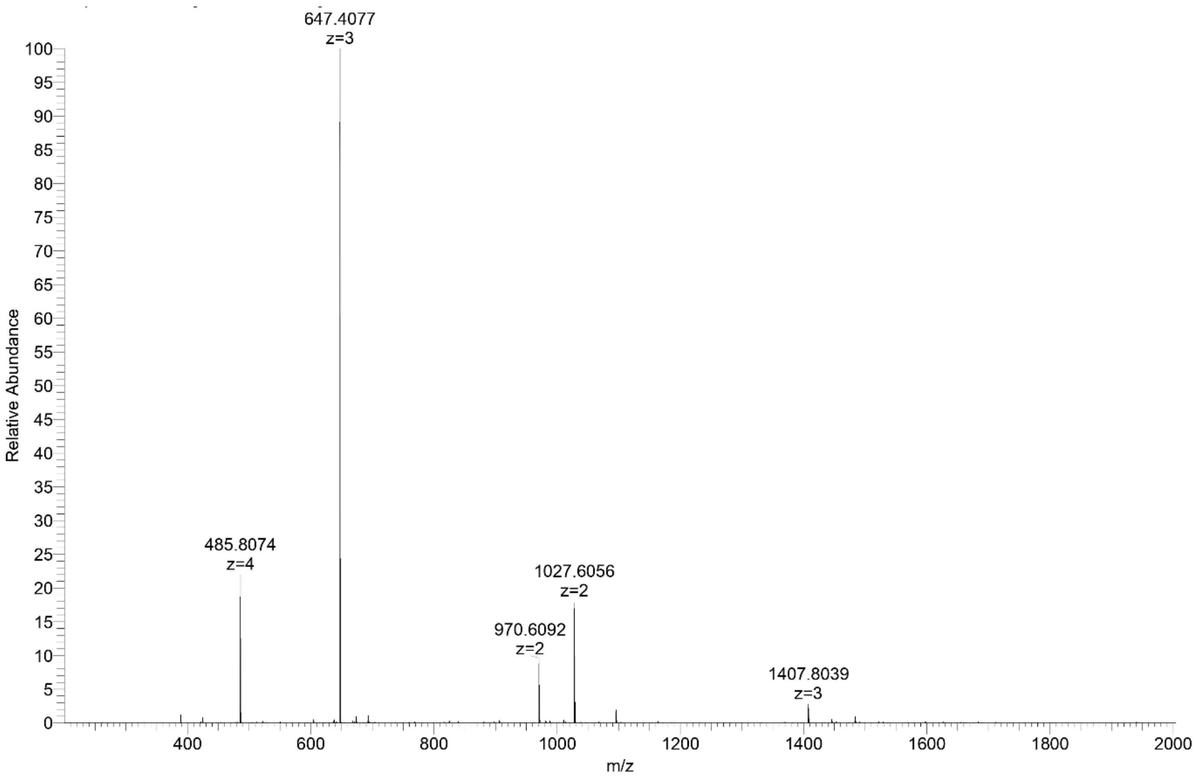
647.4077
z=3
485.8074
z=4
1027.6056
z=2
970.6092
z=2
1407.8039
z=3
Relative Abundance
m/z

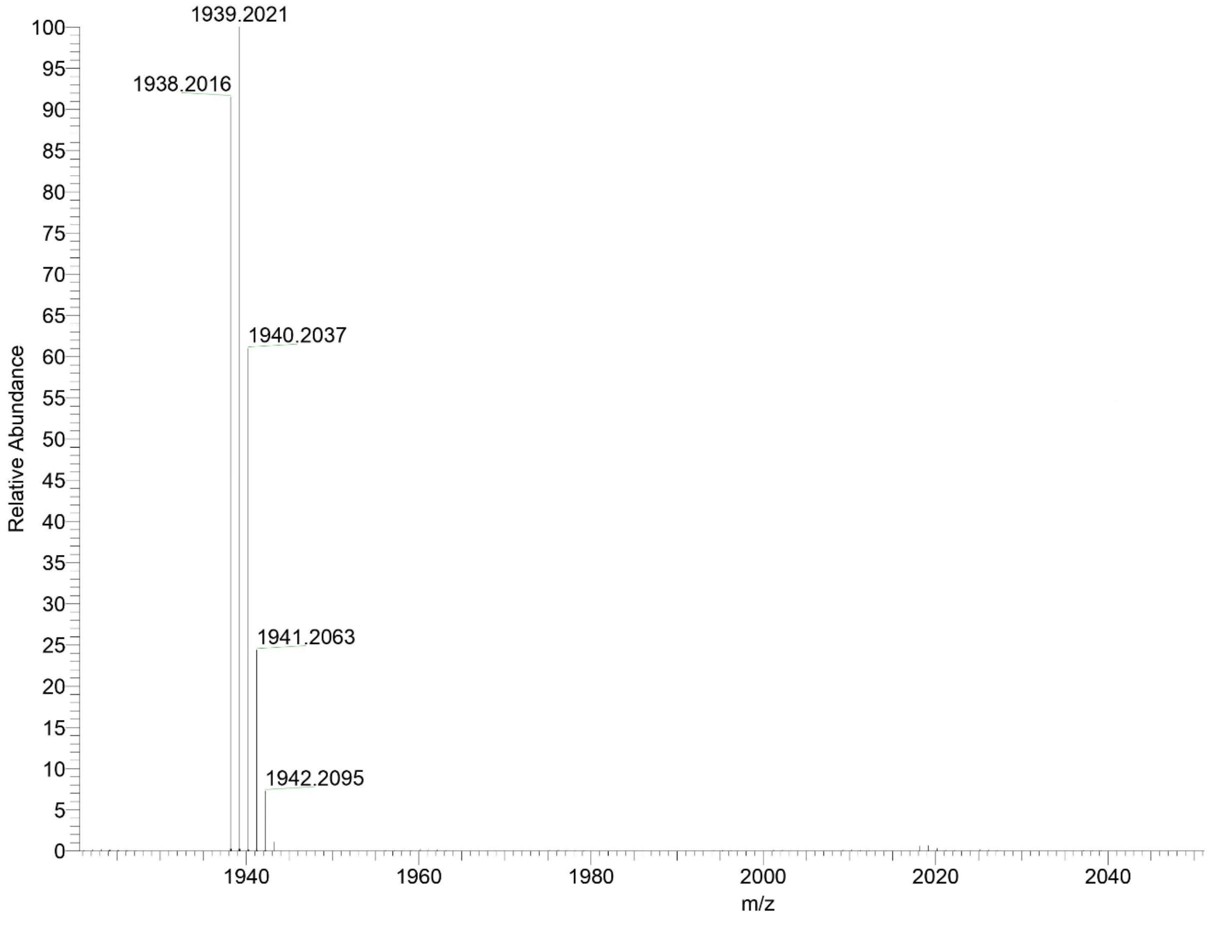
1939.2021
1938.2016
1940.2037
1941.2063
1942.2095
Relative Abundance
m/z

A5 (WKRIVRIIRWIRKYY-NH_2) was obtained after manual synthesis from Rink Amide AM resin LL (300 mg, 0.29 mmol/g), the peptide was obtained as a white foamy solid after preparative RP-HPLC purification (35 mg, 14.2%). Analytical RP-HPLC: t_R = 1.48 min (100% A to 100% D in 3.5 min, λ= 214 nm). MS (ESI+): $C_{105}H_{166}N_{32}O_{17}$ calc./obs. 2147.31/2147.31$[M]^+$

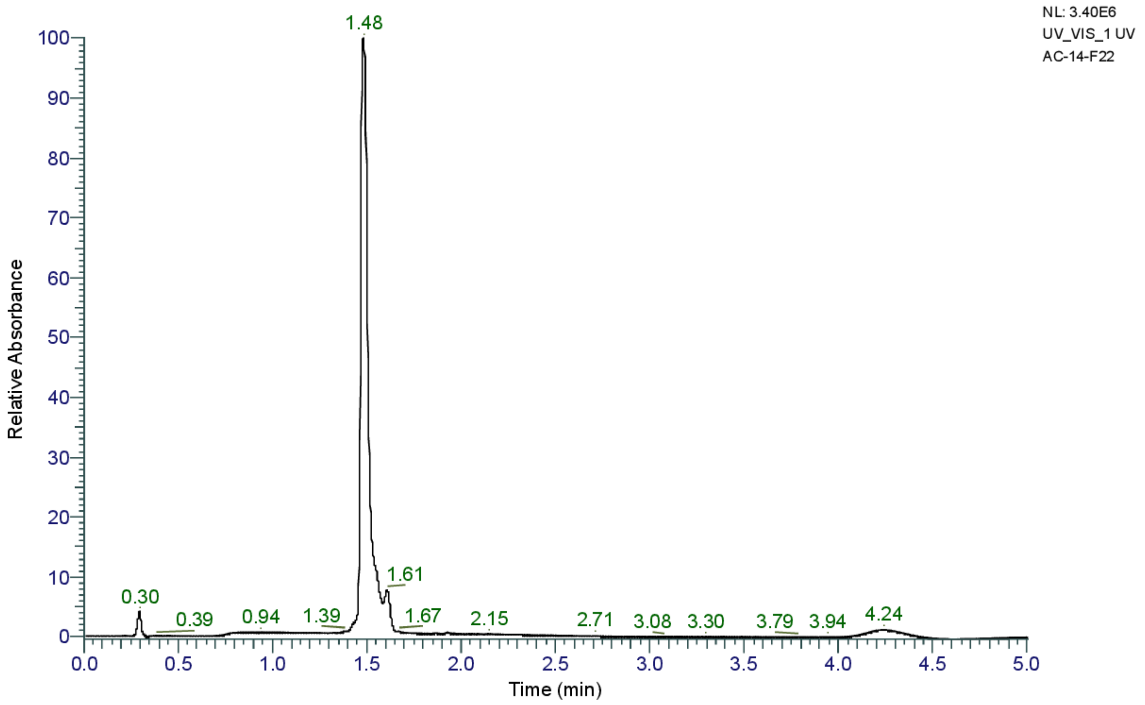

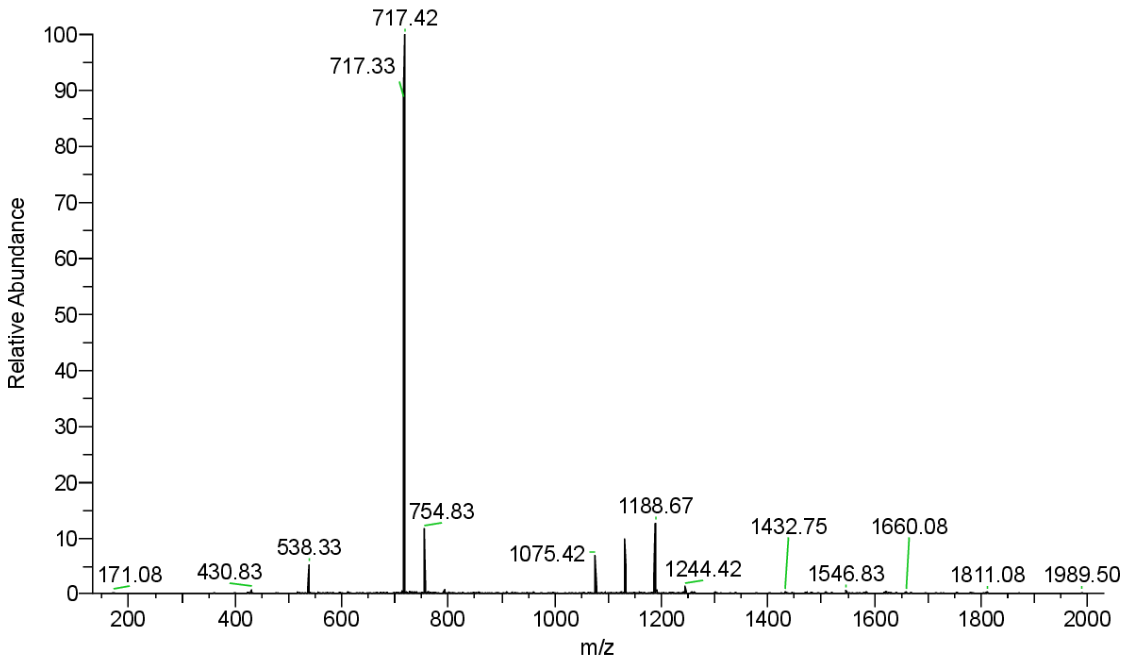

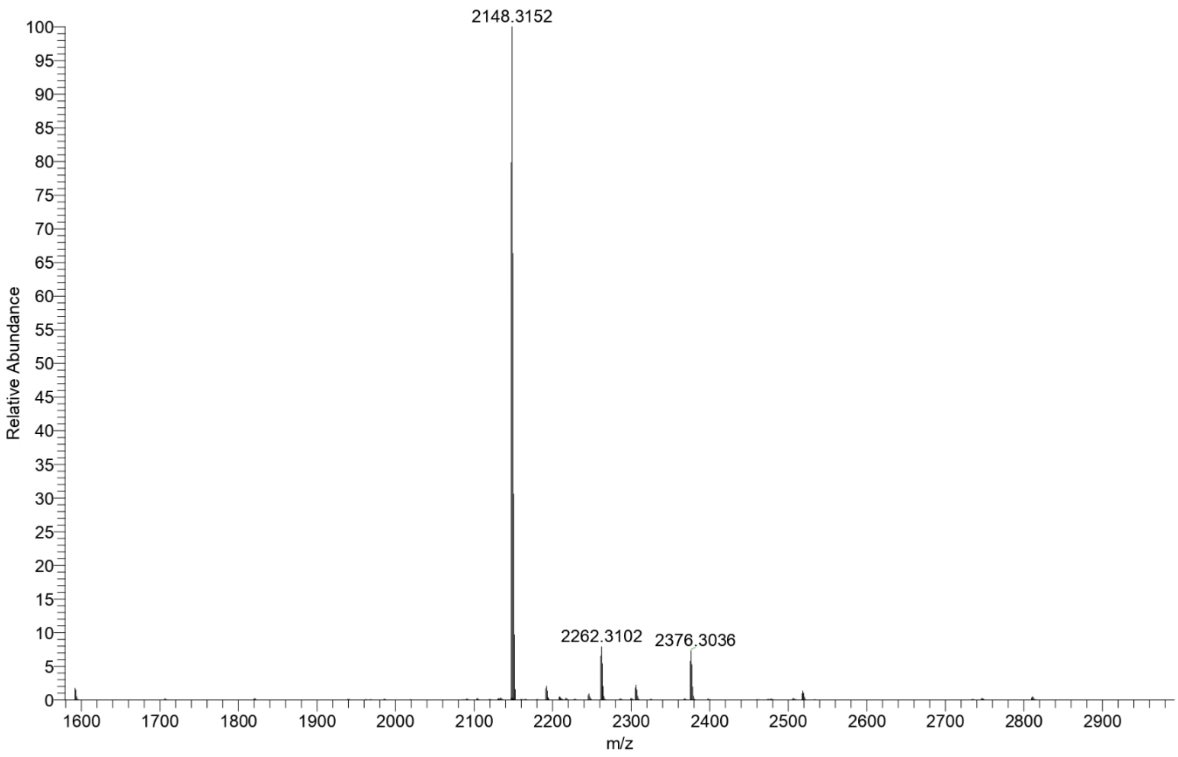
2148.3152
2262.3102
2376.3036
Relative Abundance
m/z

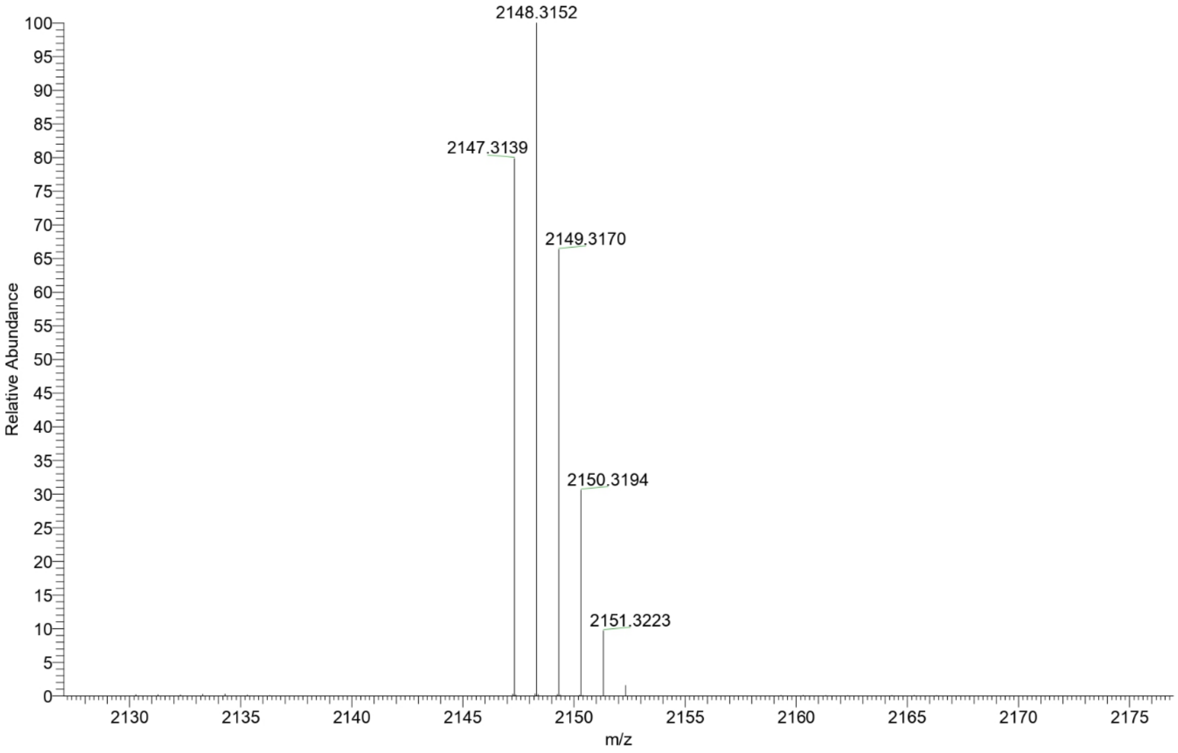
2148.3152
2147.3139
2149.3170
2150.3194
2151.3223
Relative Abundance
m/z

A6 (FAARILRAWFRFLRR-NH_2) was obtained after manual synthesis from Rink Amide AM resin LL (300 mg, 0.29 mmol/g), the peptide was obtained as a white foamy solid after preparative RP-HPLC purification (32 mg, 15.5%). Analytical RP-HPLC: t_R = 1.61 min (100% A to 100% D in 3.5 min, λ= 214 nm). MS (ESI+): $C_{95}H_{148}N_{32}O_{15}$ calc./obs. 1977.18/1977.18 $[M]^+$, $C_{99}H_{150}F_6N_{32}O_{19}$ calc./obs. 2205.16/2205.17 $[M+H+2CF_3COOH]^+$

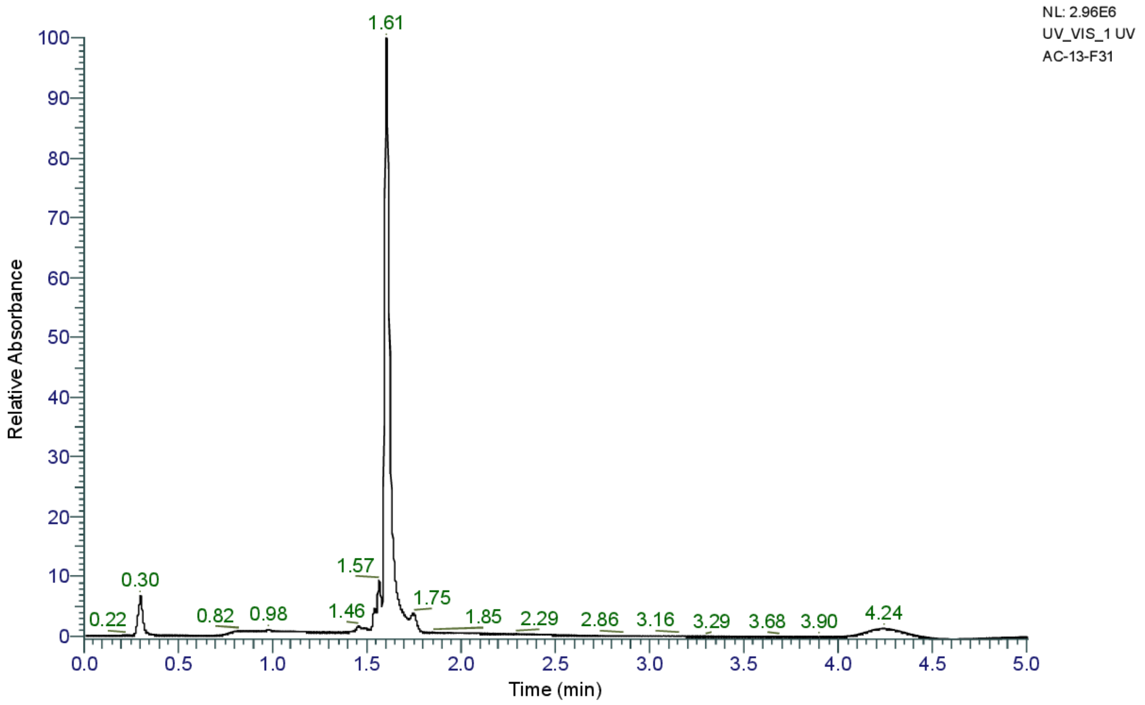

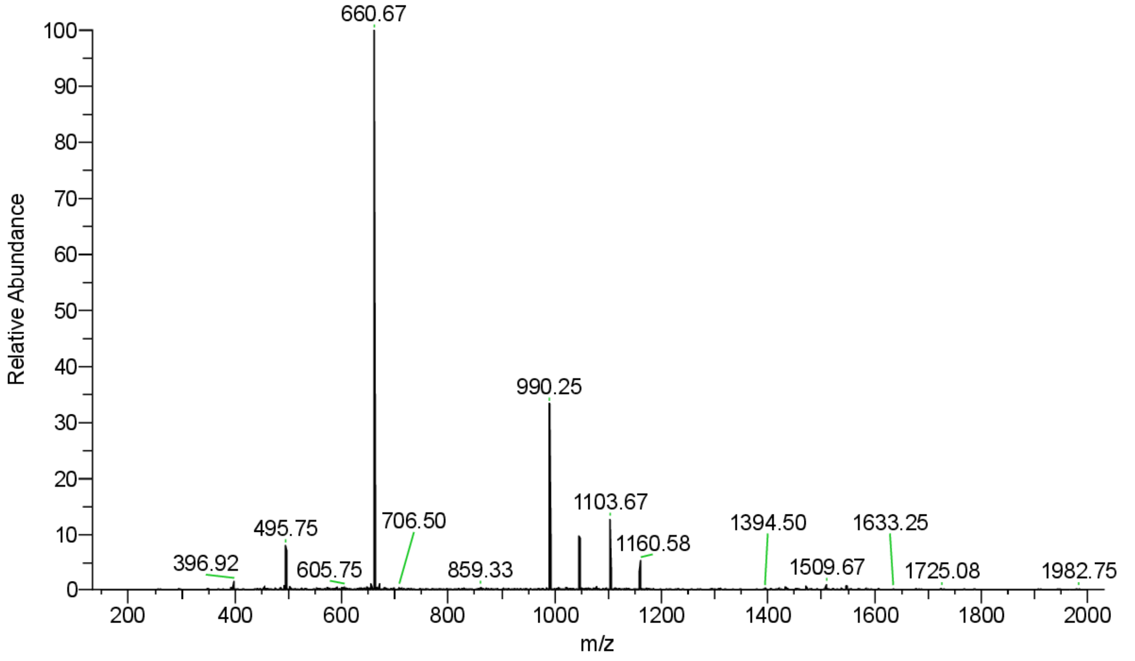

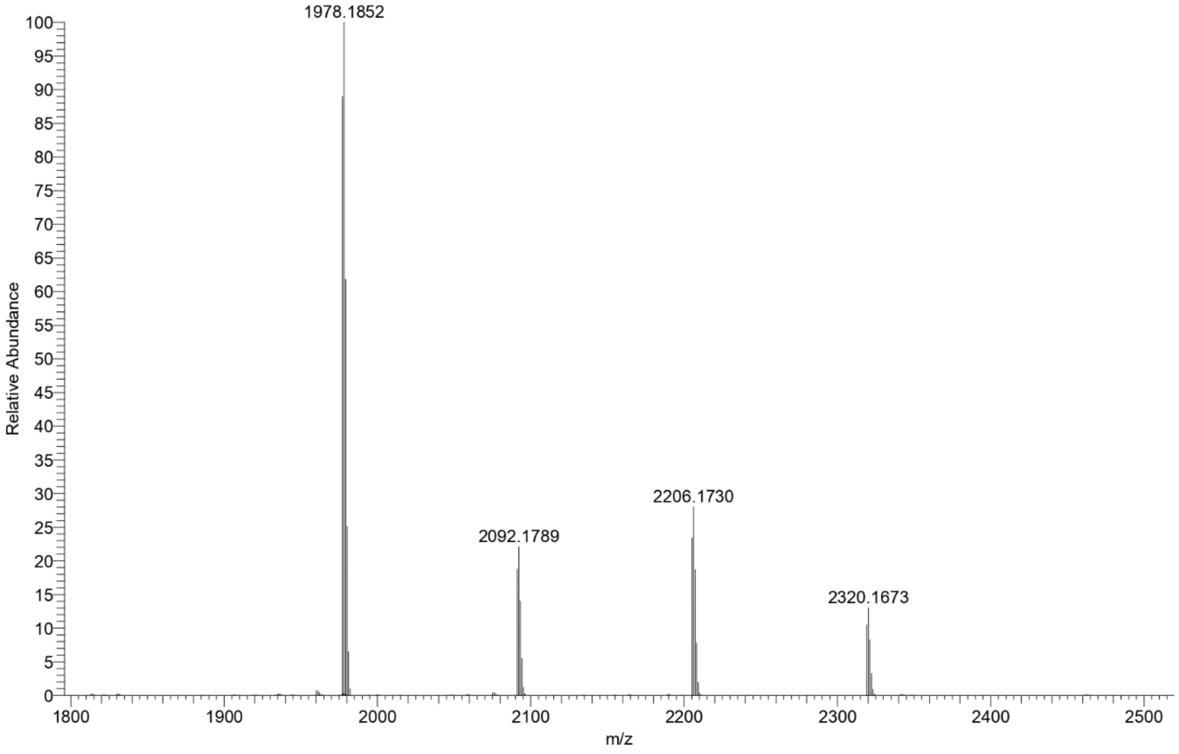
1978.1852
2092.1789
2206.1730
2320.1673
Relative Abundance
m/z

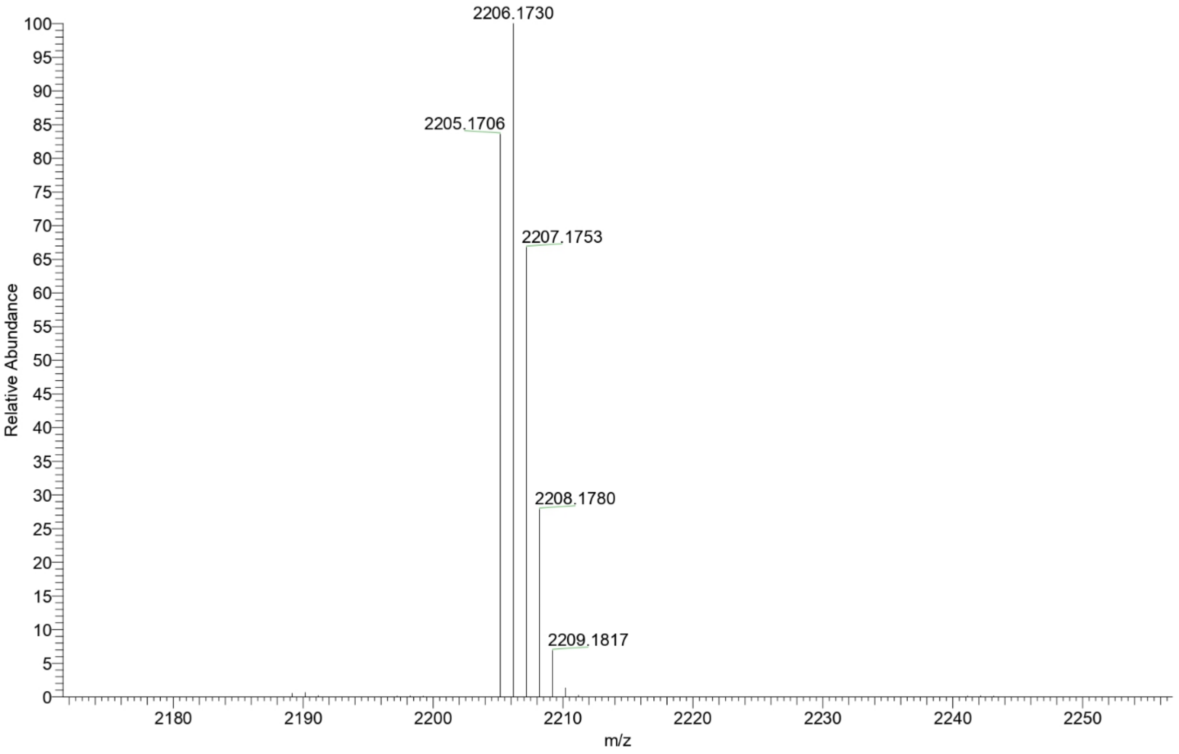
2206.1730
2205.1706
2207.1753
2208.1780
2209.1817
Relative Abundance
m/z

A7 (SISRLWHSLLRHLLH-NH_2) was obtained after manual synthesis from Rink Amide AM resin LL (150 mg, 0.29 mmol/g), the peptide was obtained as a white foamy solid after preparative RP-HPLC purification (18 mg, 19.9%). Analytical RP-HPLC: t_R = 1.64 min (100% A to 100% D in 3.5 min, λ= 214 nm). MS (ESI+): $C_{86}H_{139}N_{29}O_{18}$ calc./obs. 1866.09/1866.08 $[M]^+$

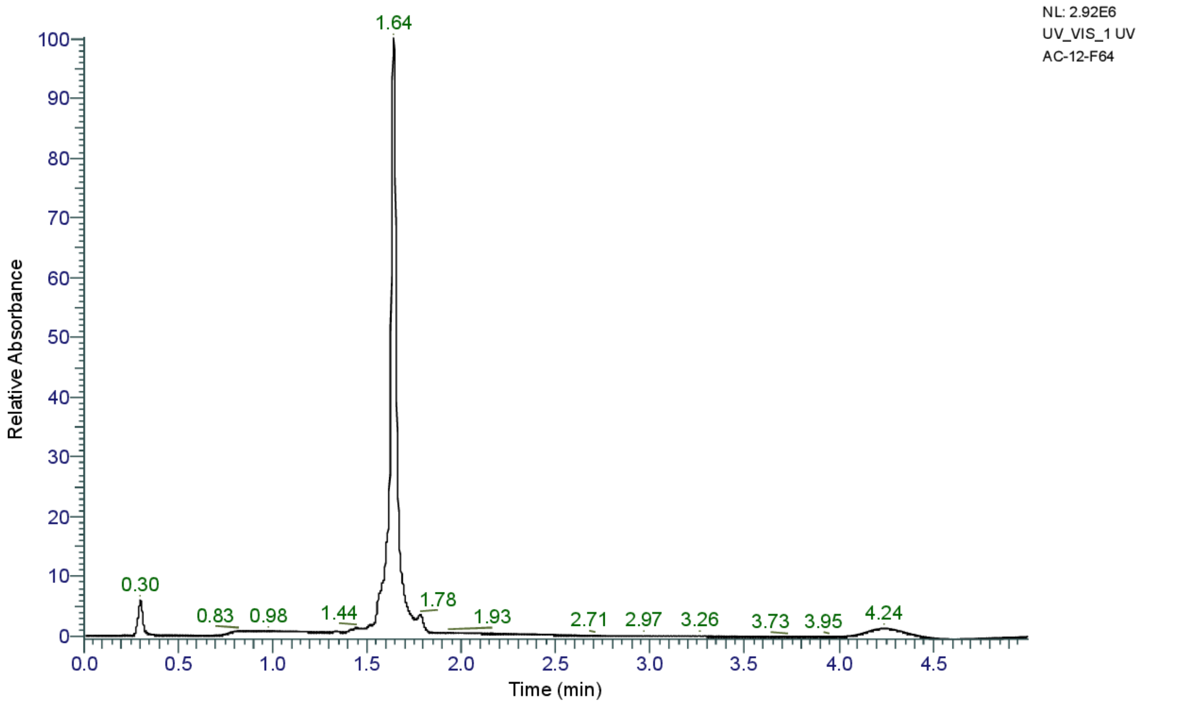

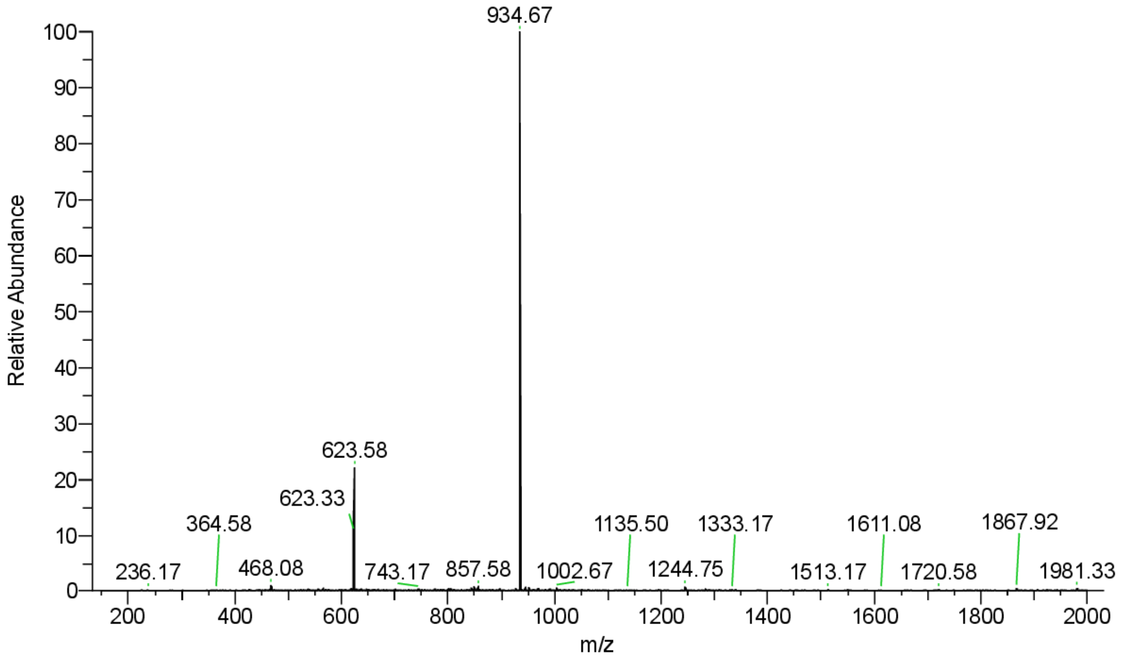

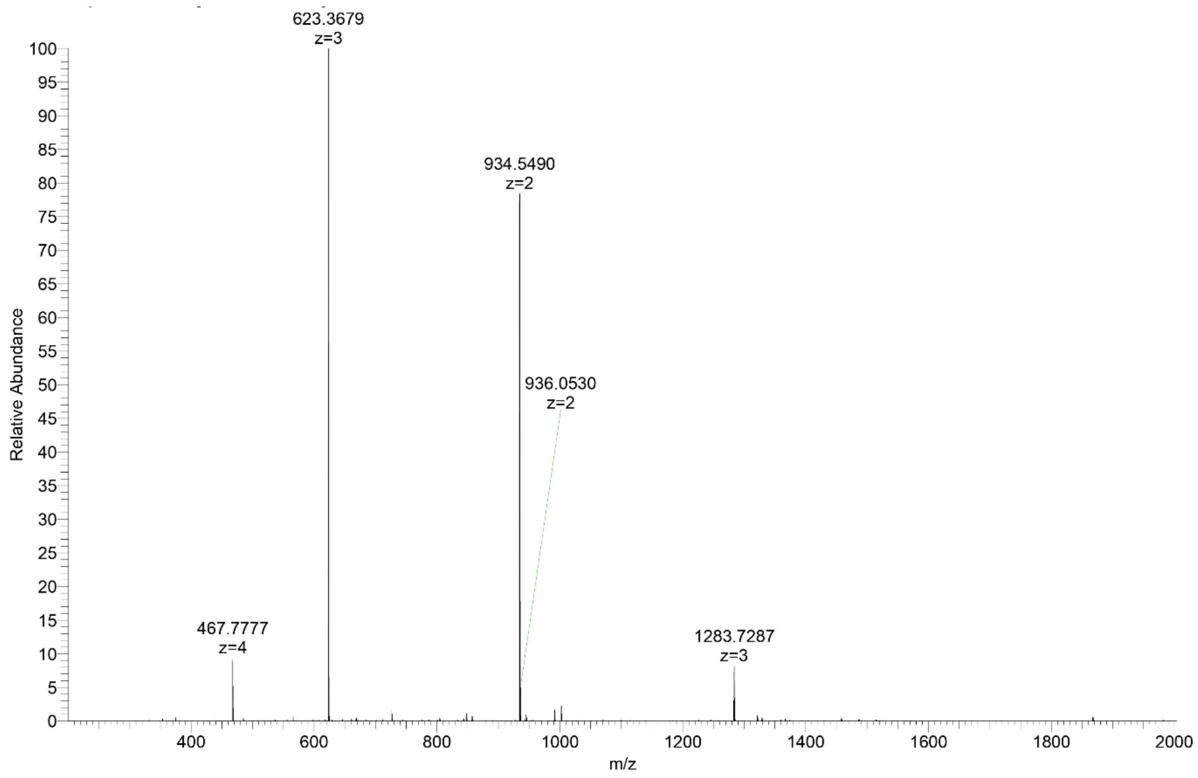
623.3679
z=3
934.5490
z=2
936.0530
z=2
467.7777
z=4
1283.7287
z=3
Relative Abundance
m/z

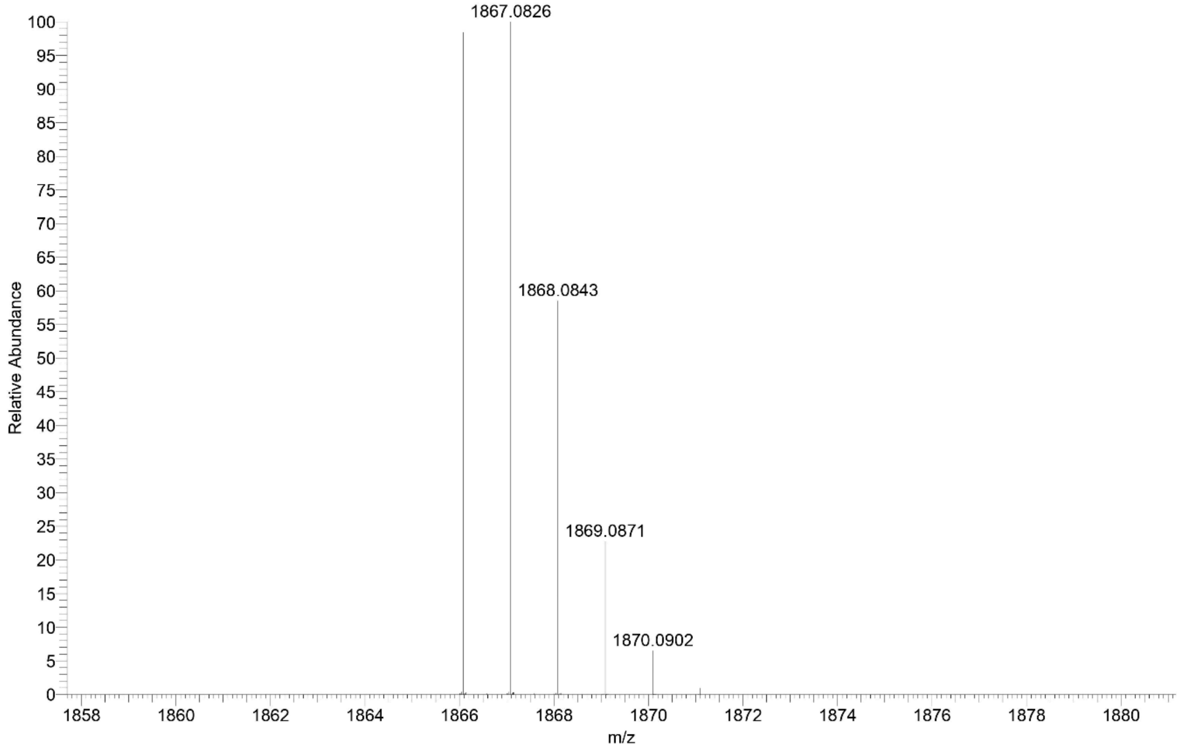
1867.0826
1868.0843
1869.0871
1870.0902
Relative Abundance
m/z

A8 (KNFKKLMKKVASVL-NH$_2$) was obtained after manual synthesis from Rink Amide AM resin LL (150 mg, 0.29 mmol/g), the peptide was obtained as a white foamy solid after preparative RP-HPLC purification (16 mg, 16.8%). Analytical RP-HPLC: t_R = 1.42 min (100% A to 100% D in 3.5 min, λ= 214 nm). MS (ESI+): $C_{76}H_{137}N_{21}O_{16}S$ calc./obs. 1632.03/1632.03 $[M]^+$

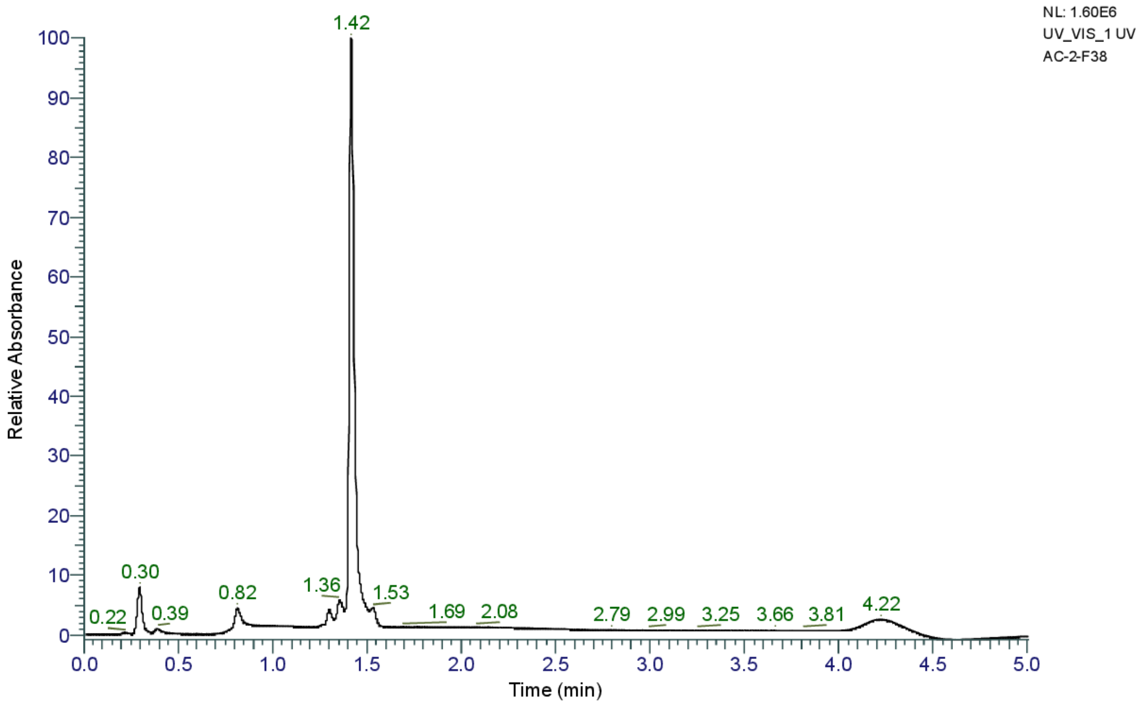

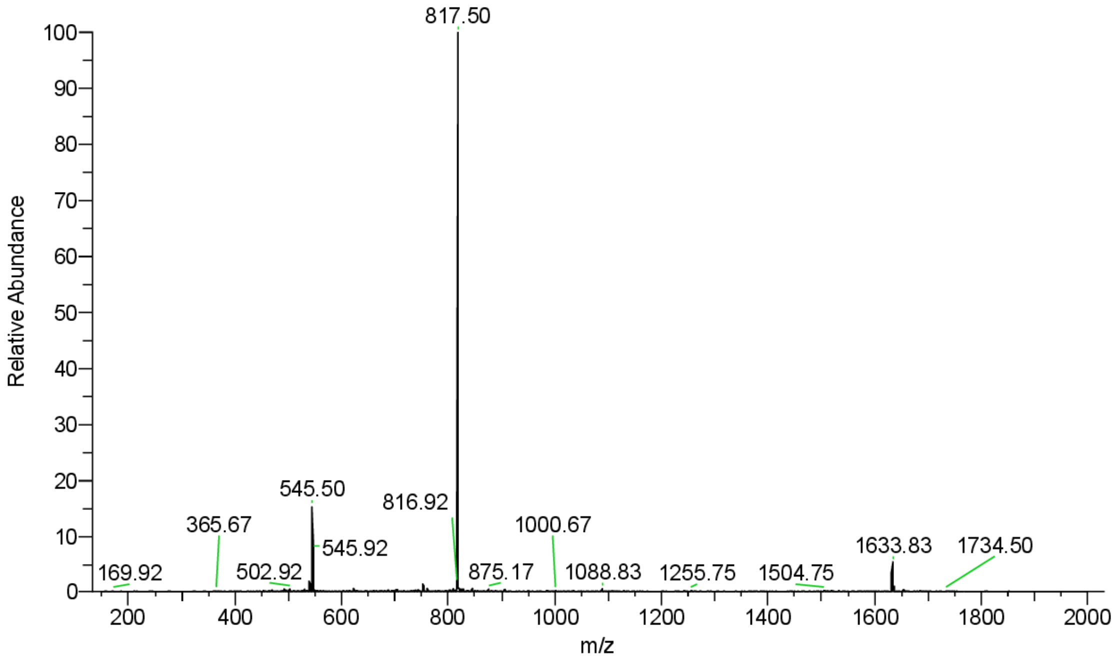

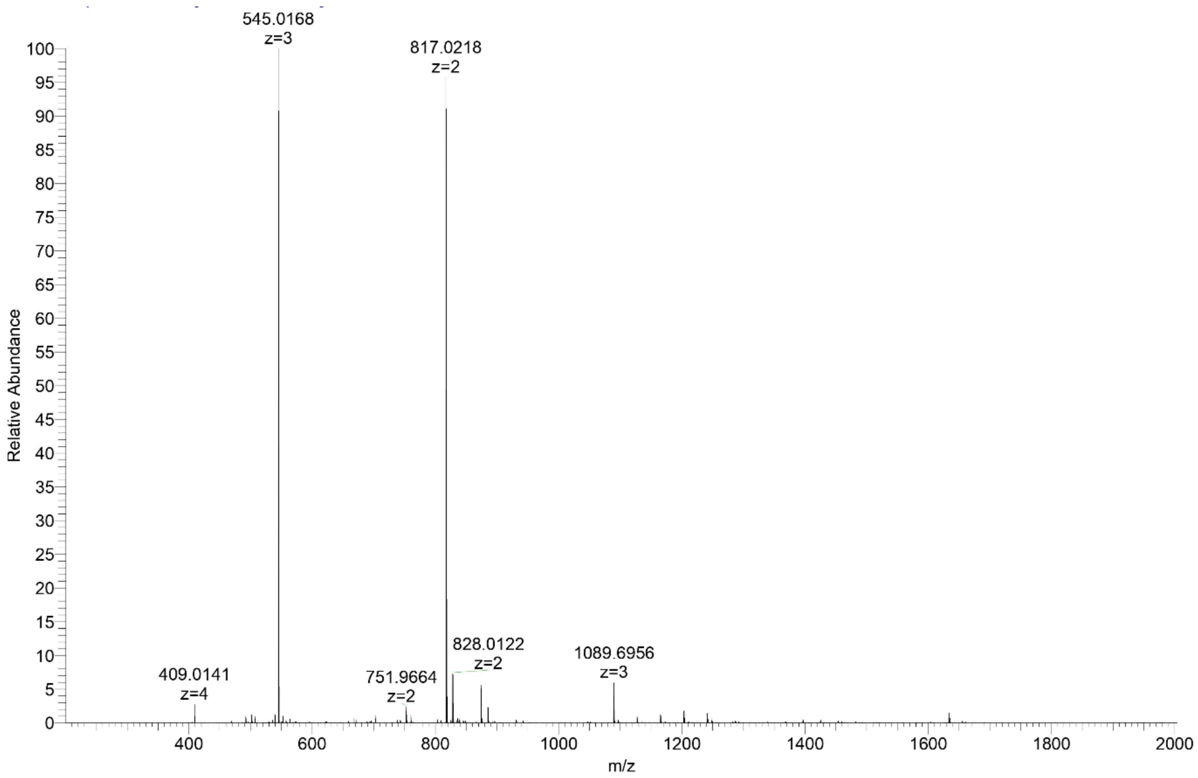
545.0168
z=3
817.0218
z=2
409.0141
z=4
751.9664
z=2
828.0122
z=2
1089.6956
z=3
Relative Abundance
m/z

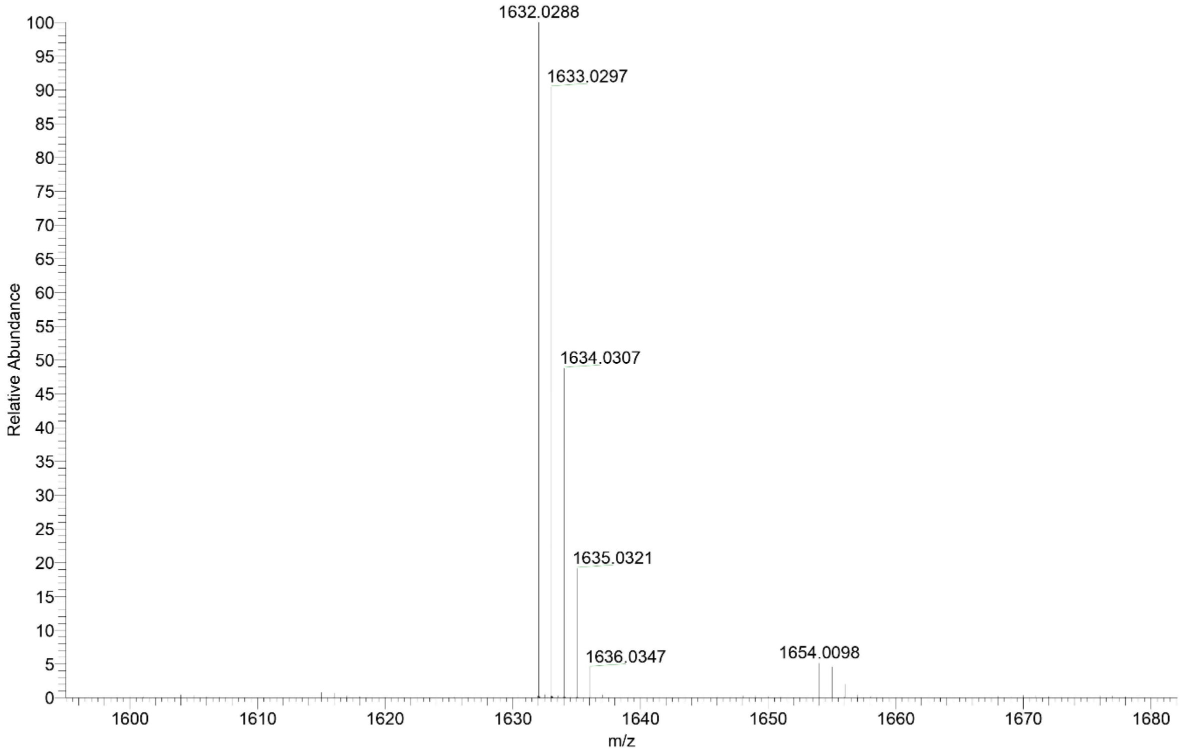
1632.0288
1633.0297
1634.0307
1635.0321
1636.0347
1654.0098
Relative Abundance
m/z

A9 (SFSKWMGKLKNIFKK-NH$_2$) was obtained after manual synthesis from Rink Amide AM resin LL (150 mg, 0.29 mmol/g), the peptide was obtained as a white foamy solid after preparative RP-HPLC purification (25 mg, 23.9%). Analytical RP-HPLC: t_R = 1.48 min (100% A to 100% D in 3.5 min, λ= 214 nm). MS (ESI+): $C_{88}H_{141}N_{23}O_{18}S$ calc./obs. 1840.05/1840.06[M]$^+$

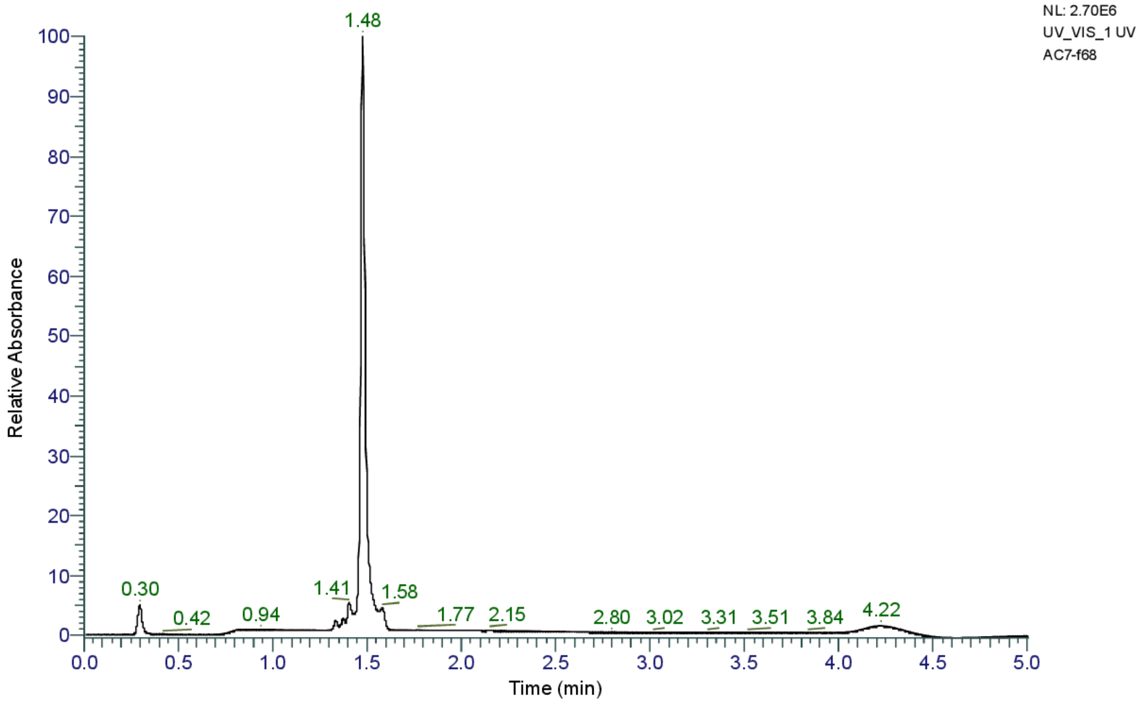

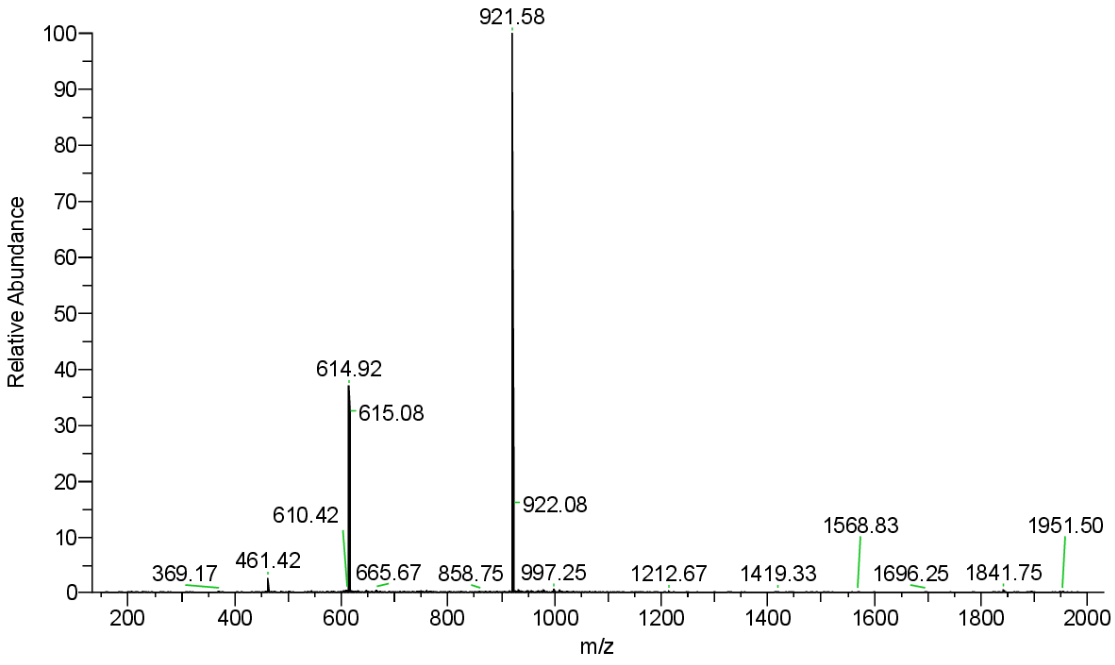

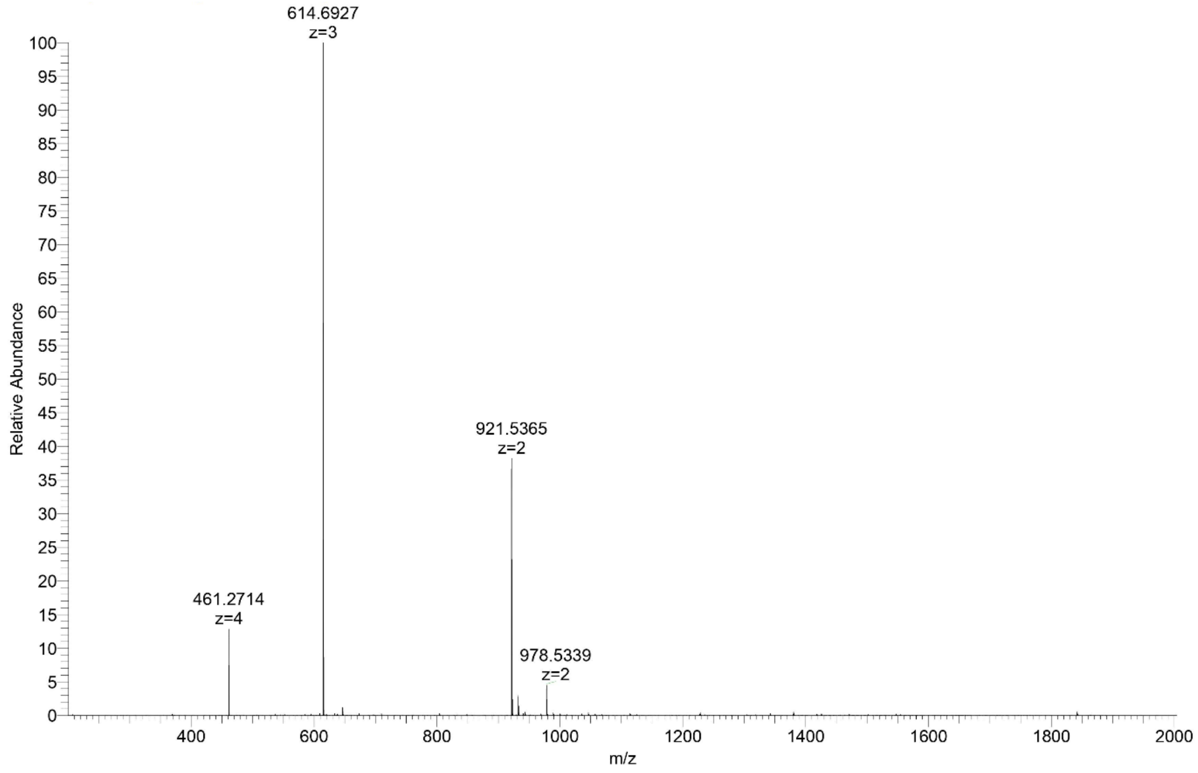
614.6927
z=3
921.5365
z=2
461.2714
z=4
978.5339
z=2
Relative Abundance
m/z

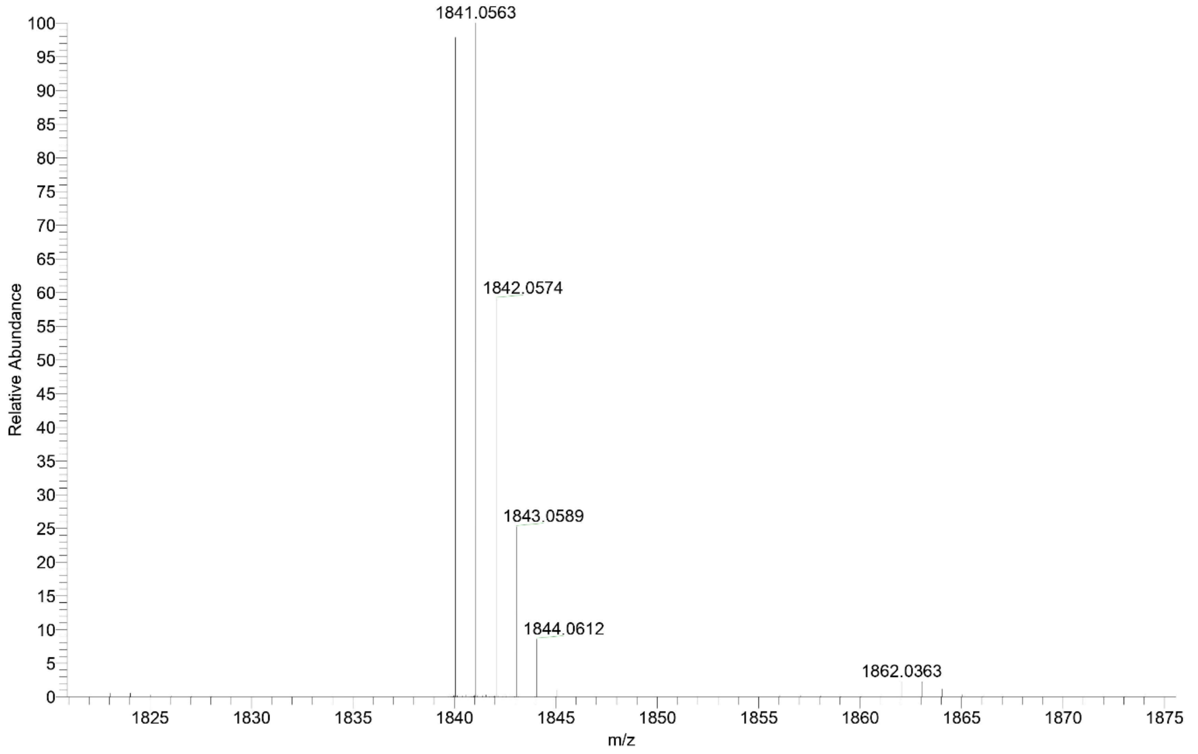
1841.0563
1842.0574
1843.0589
1844.0612
1862.0363
Relative Abundance
m/z

A10 (LLRHCLRRIRDRLV-NH_2) was obtained after manual synthesis from Rink Amide AM resin LL (300 mg, 0.29 mmol/g), the peptide was obtained as a white foamy solid after preparative RP-HPLC purification (36 mg, 18.2%). Analytical RP-HPLC: t_R = 1.32 min (100% A to 100% D in 3.5 min, λ= 214 nm). MS (ESI+): $C_{78}H_{144}N_{32}O_{16}S$ calc./obs. 1817.12/1817.11[M]$^+$

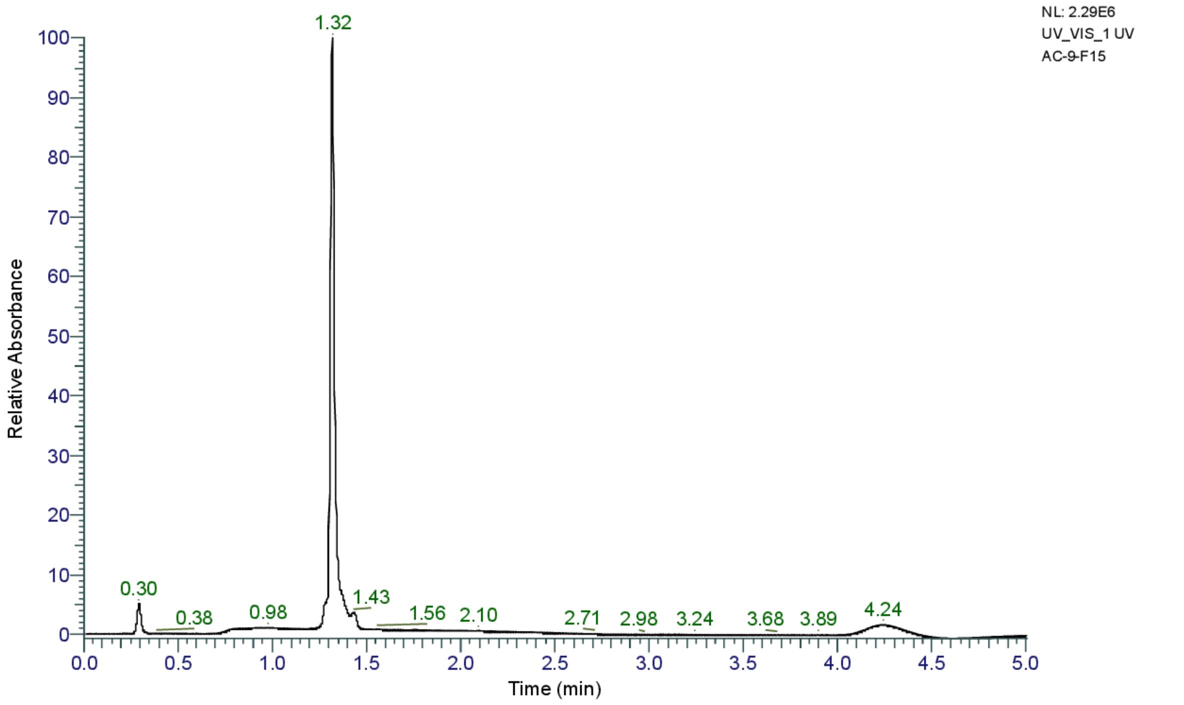

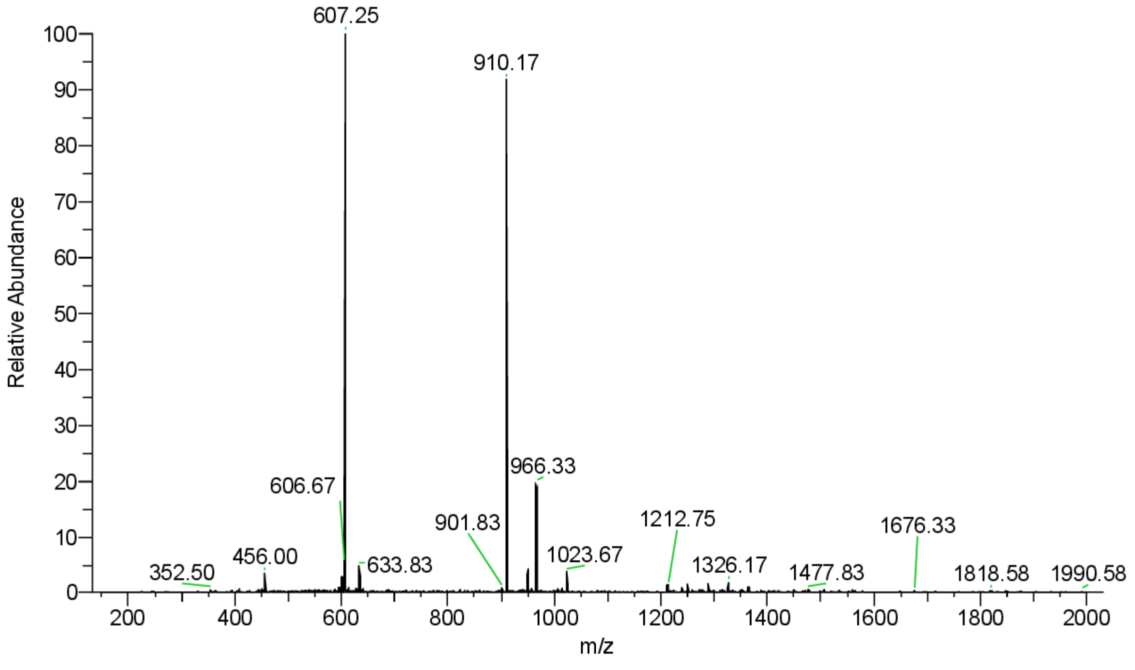

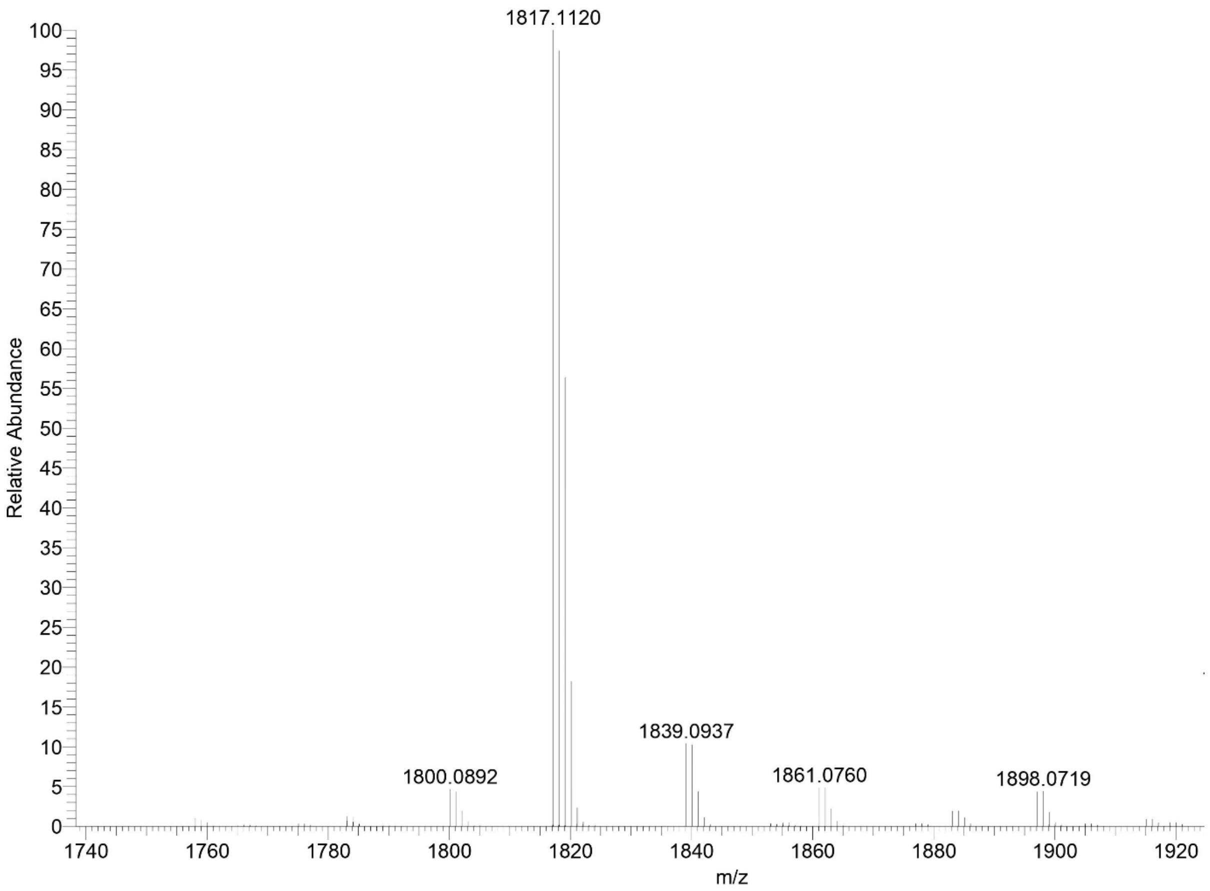
1817.1120
1800.0892
1839.0937
1861.0760
1898.0719
Relative Abundance
m/z

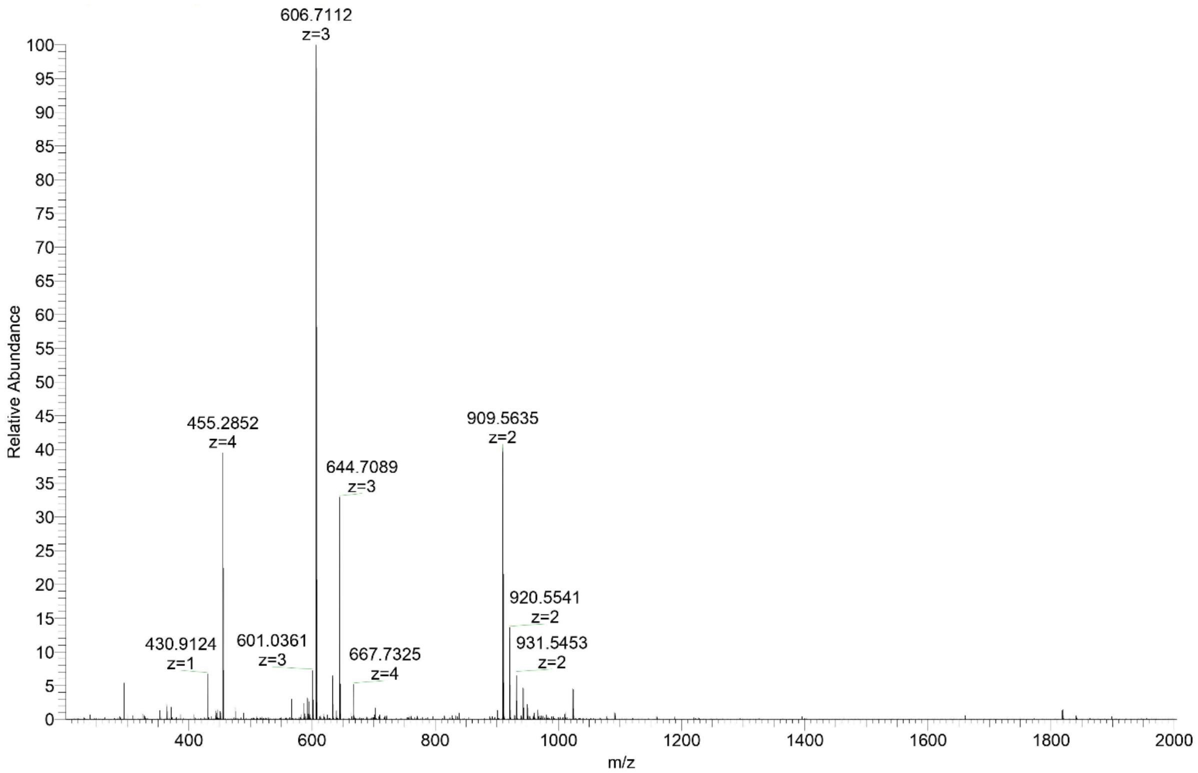
606.7112
z=3
455.2852
z=4
909.5635
z=2
644.7089
z=3
920.5541
z=2
931.5453
z=2
430.9124
z=1
601.0361
z=3
667.7325
z=4
Relative Abundance
m/z

A11(KWRSKIKKIMRTFK-NH$_2$) was obtained after manual synthesis from Rink Amide AM resin LL (150 mg, 0.29 mmol/g), the peptide was obtained as a white foamy solid after preparative RP-HPLC purification (23 mg, 20.0%). Analytical RP-HPLC: t_R = 1.29 min (100% A to 100% D in 3.5 min, λ= 214 nm). MS (ESI+): $C_{86}H_{149}N_{27}O_{16}S$ calc./obs. 1848.14/1848.14 $[M]^+$

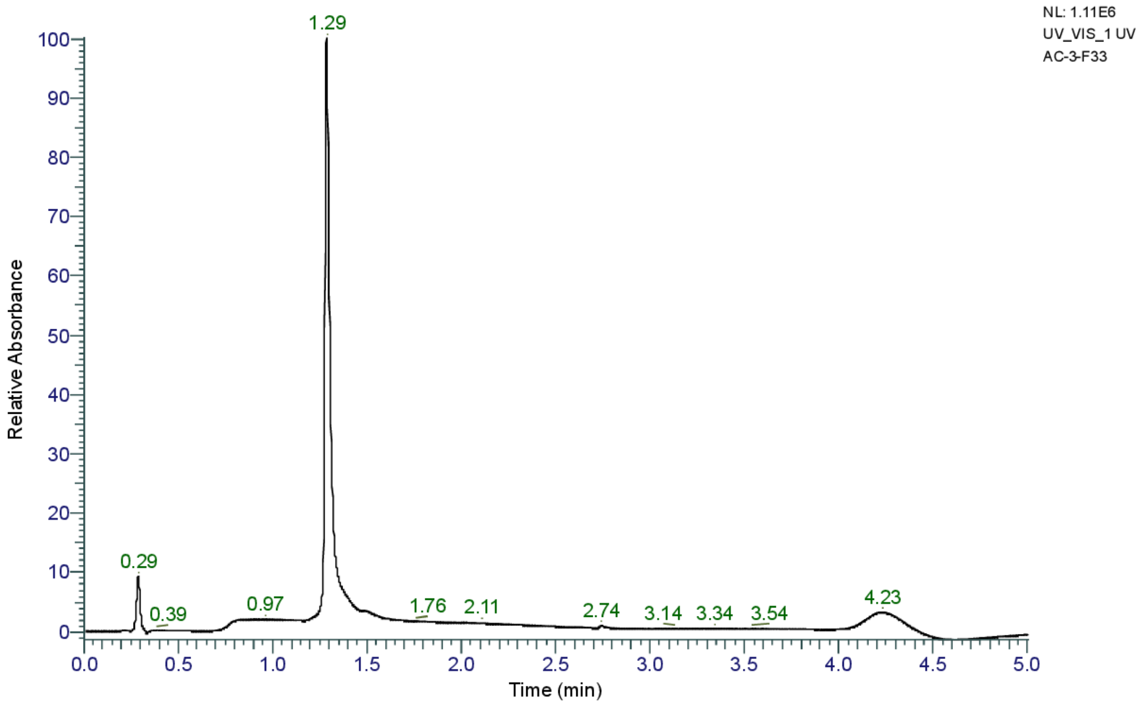

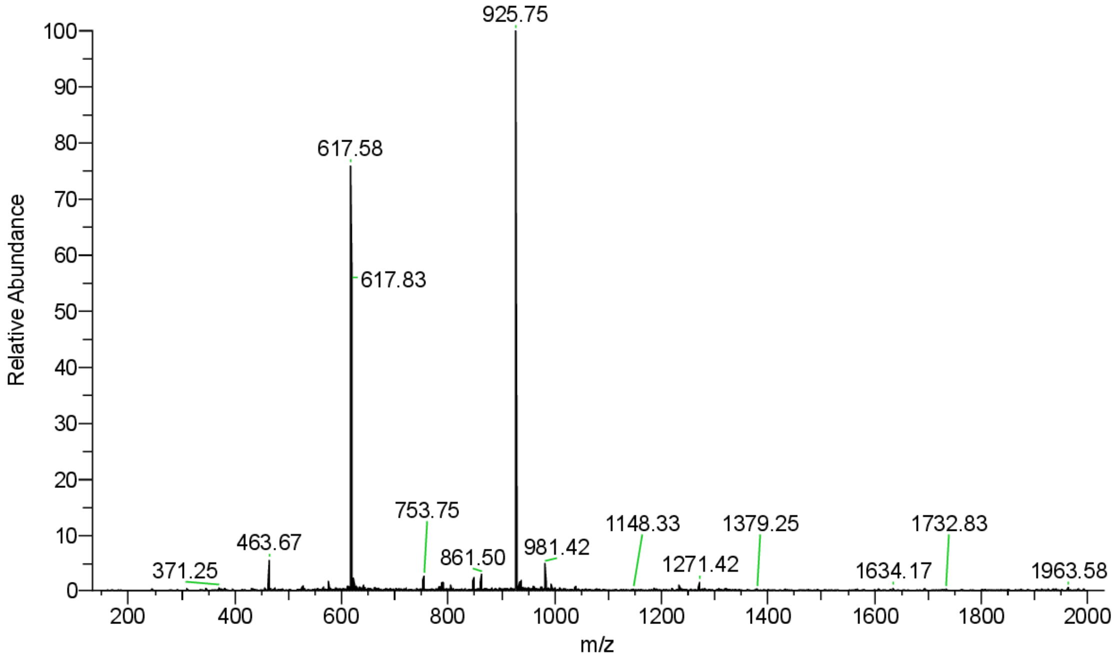

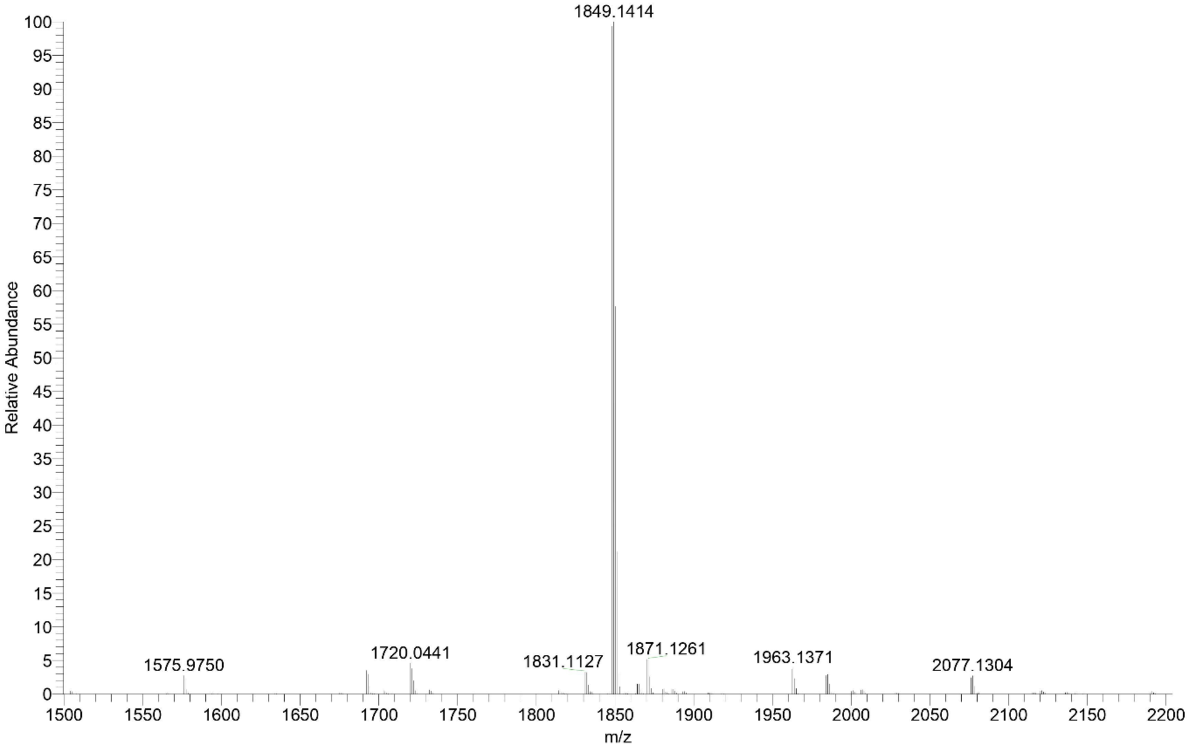
1849.1414
1575.9750
1720.0441
1831.1127
1871.1261
1963.1371
2077.1304
Relative Abundance
m/z

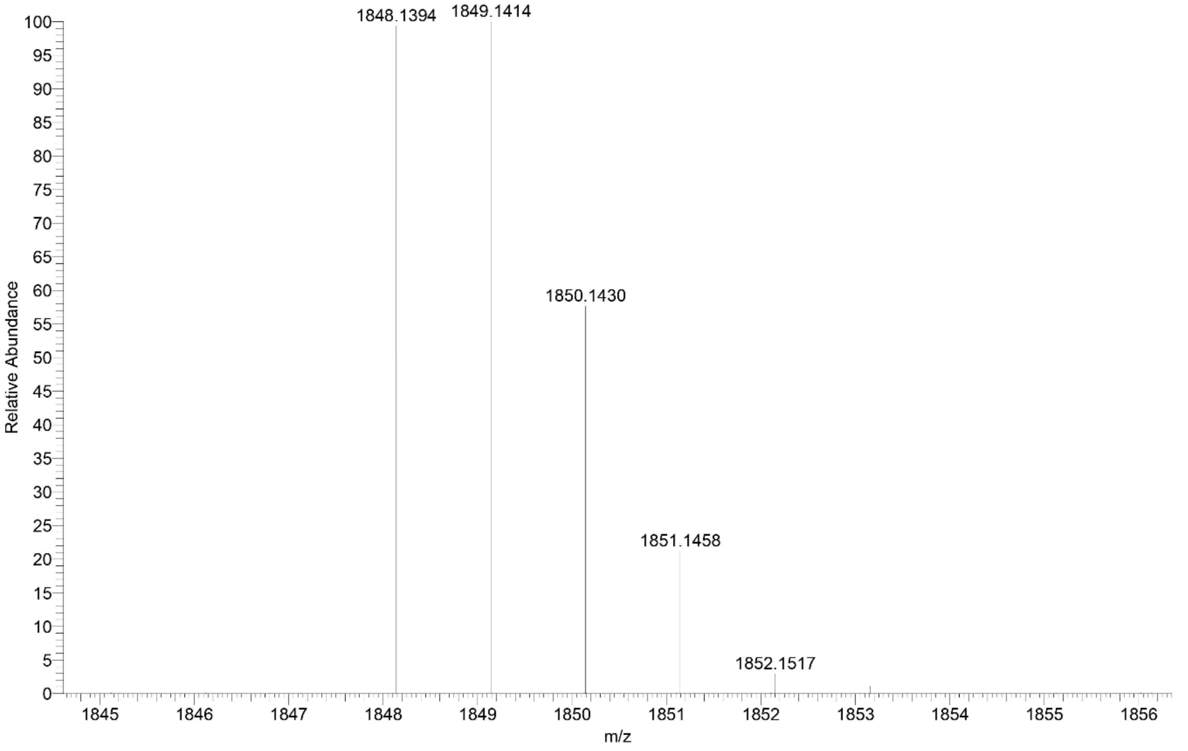
1848.1394
1849.1414
1850.1430
1851.1458
1852.1517
Relative Abundance
m/z

A12 (GLLGRLAKLLANS-NH_2) was obtained after manual synthesis from Rink Amide AM resin LL (300 mg, 0.29 mmol/g), the peptide was obtained as a white foamy solid after preparative RP-HPLC purification (31 mg, 23.0%). Analytical RP-HPLC: t_R = 1.66 min (100% A to 100% D in 3.5 min, λ= 214 nm). MS (ESI+): $C_{59}H_{109}N_{19}O_{15}$ calc./obs. 1323.84/1323.83[M]$^+$

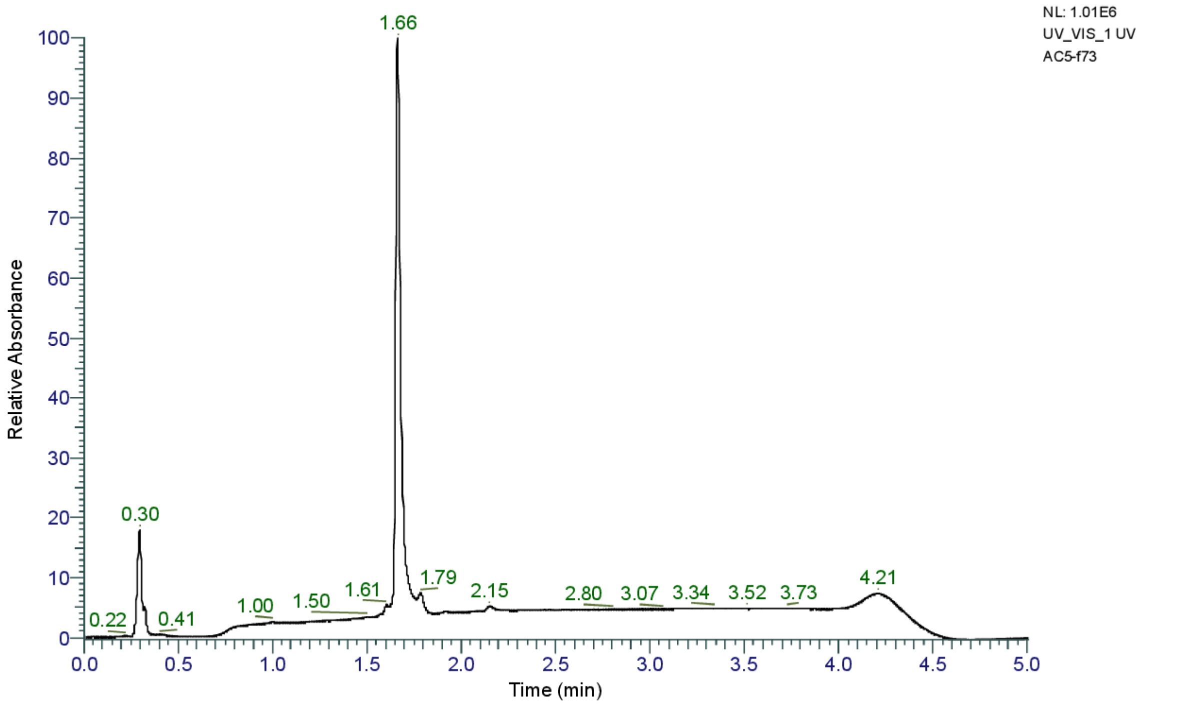

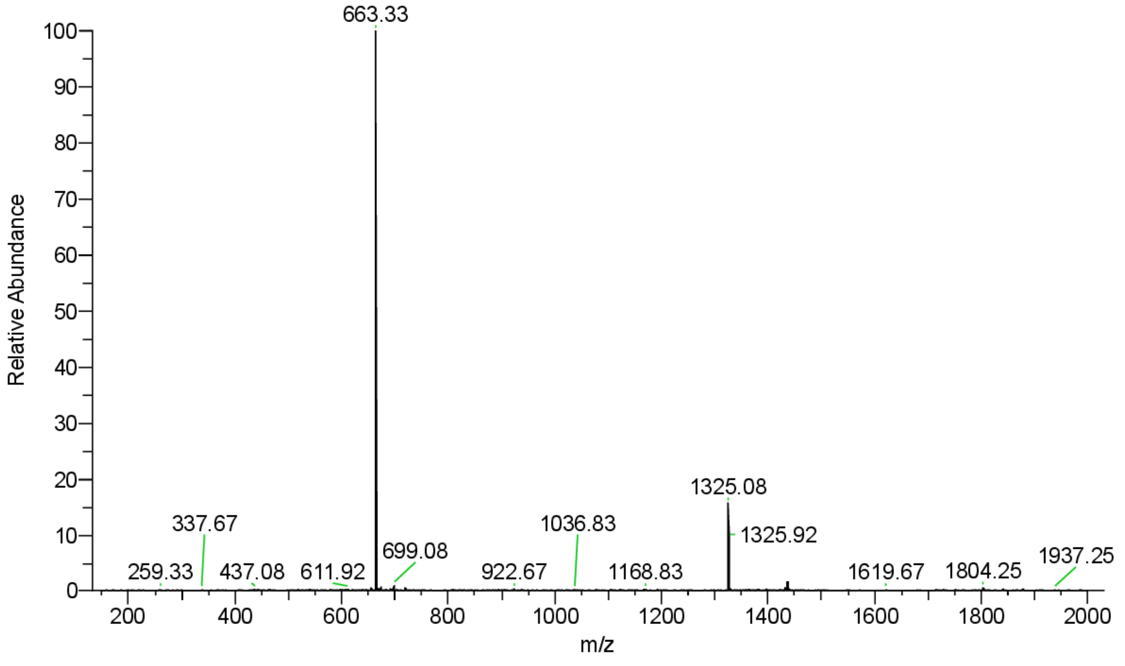

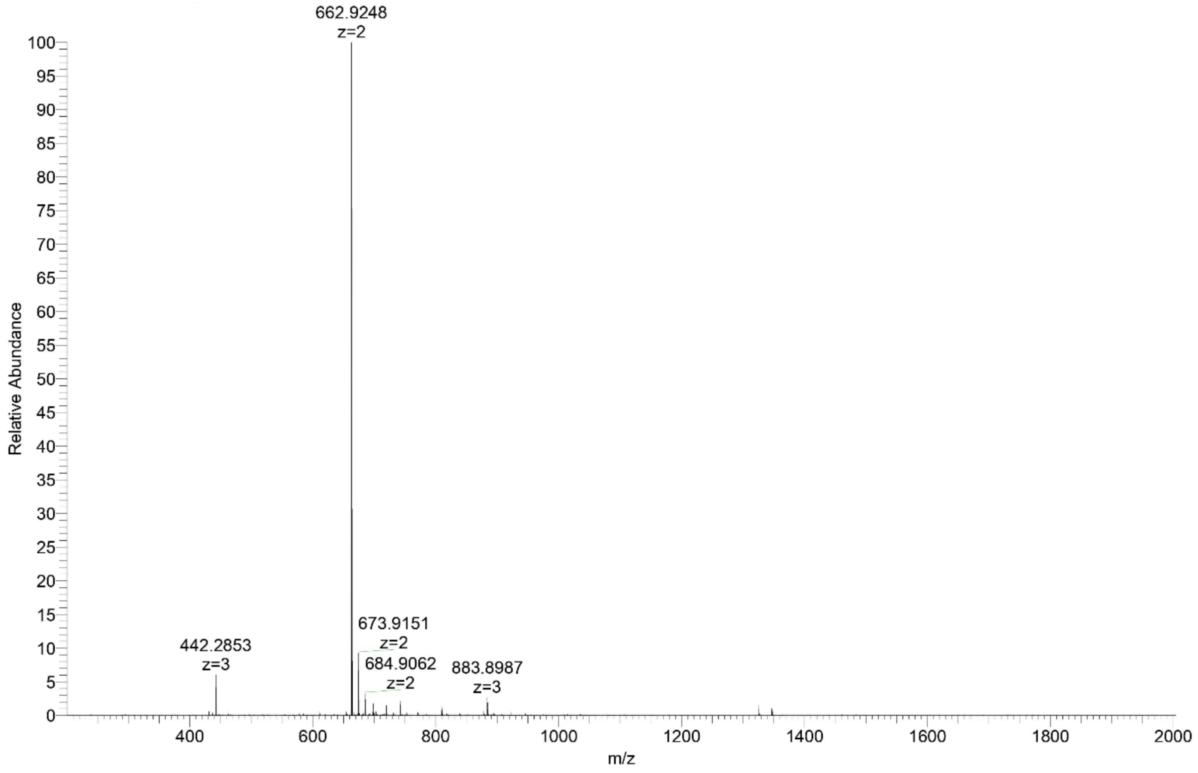
662.9248
z=2
442.2853
z=3
673.9151
z=2
684.9062
z=2
883.8987
z=3
Relative Abundance
m/z

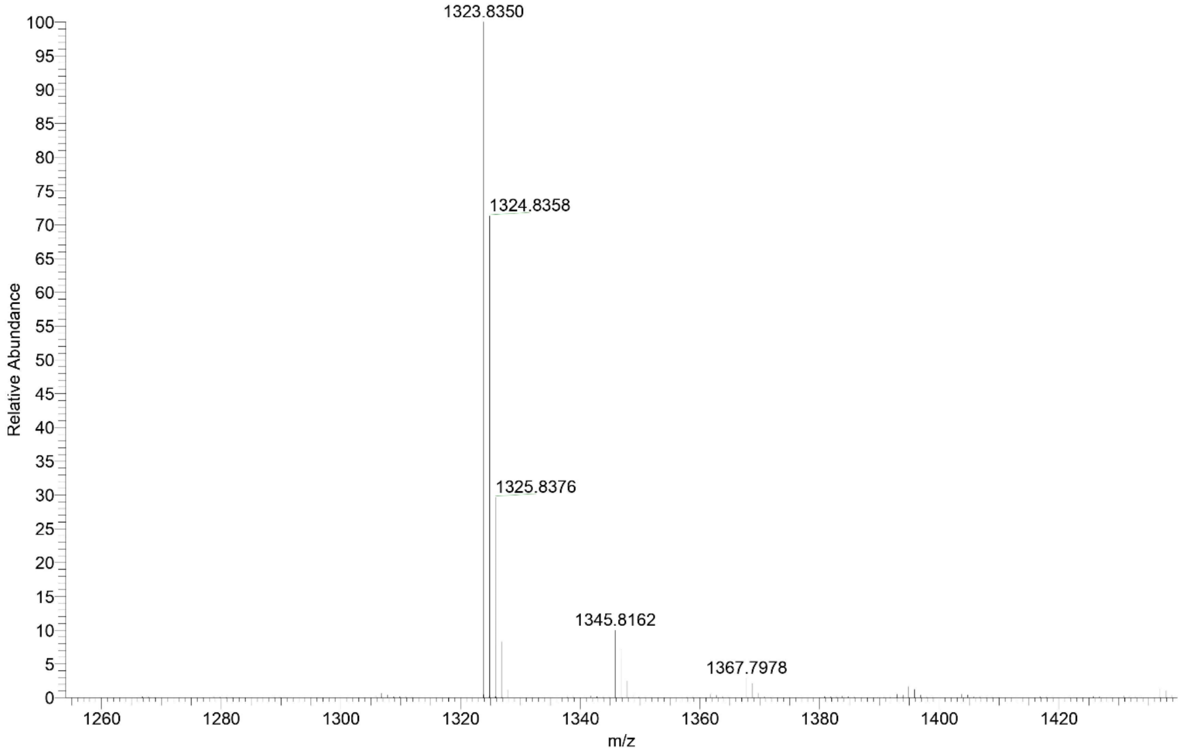
1323.8350
1324.8358
1325.8376
1345.8162
1367.7978
Relative Abundance
m/z

A13 (VFRQWQKIMRRLVRR-NH_2) was obtained after manual synthesis from Rink Amide AM resin LL (150 mg, 0.29 mmol/g), the peptide was obtained as a white foamy solid after preparative RP-HPLC purification (29 mg, 24.2%). Analytical RP-HPLC: t_R = 1.45 min (100% A to 100% D in 3.5 min, λ= 214 nm). MS (ESI+): $C_{93}H_{159}N_{35}O_{17}S$ calc./obs. 2070.24/2070.24 $[M]^+$

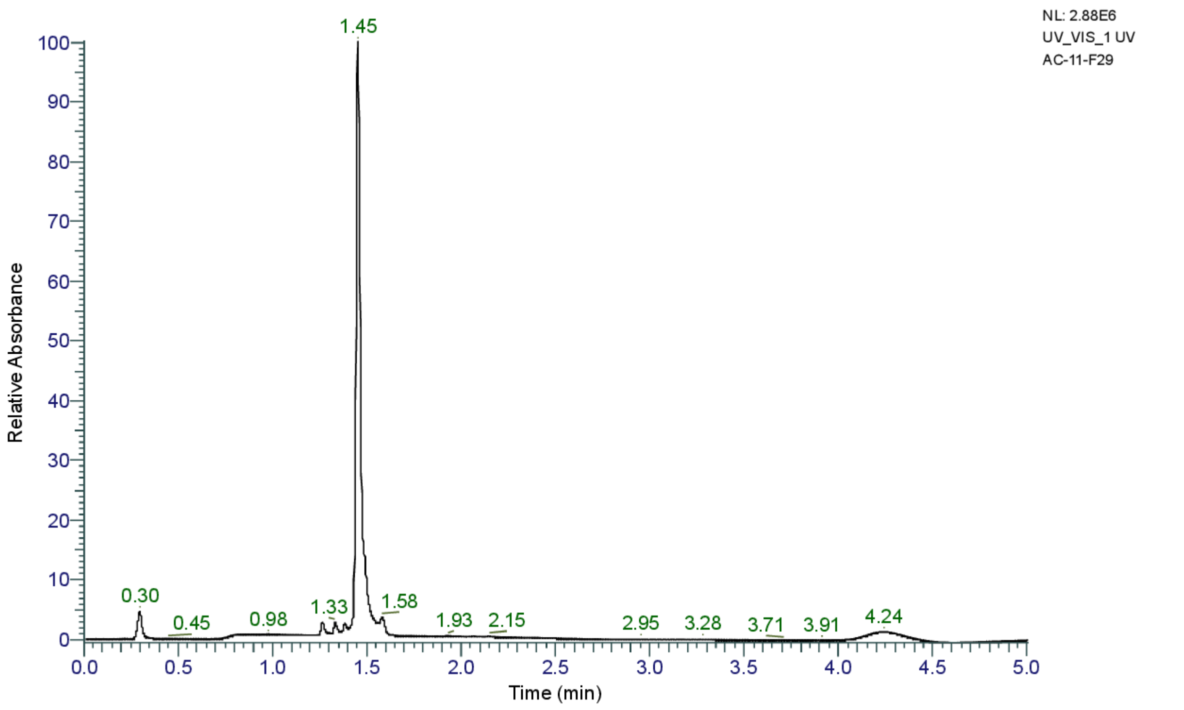

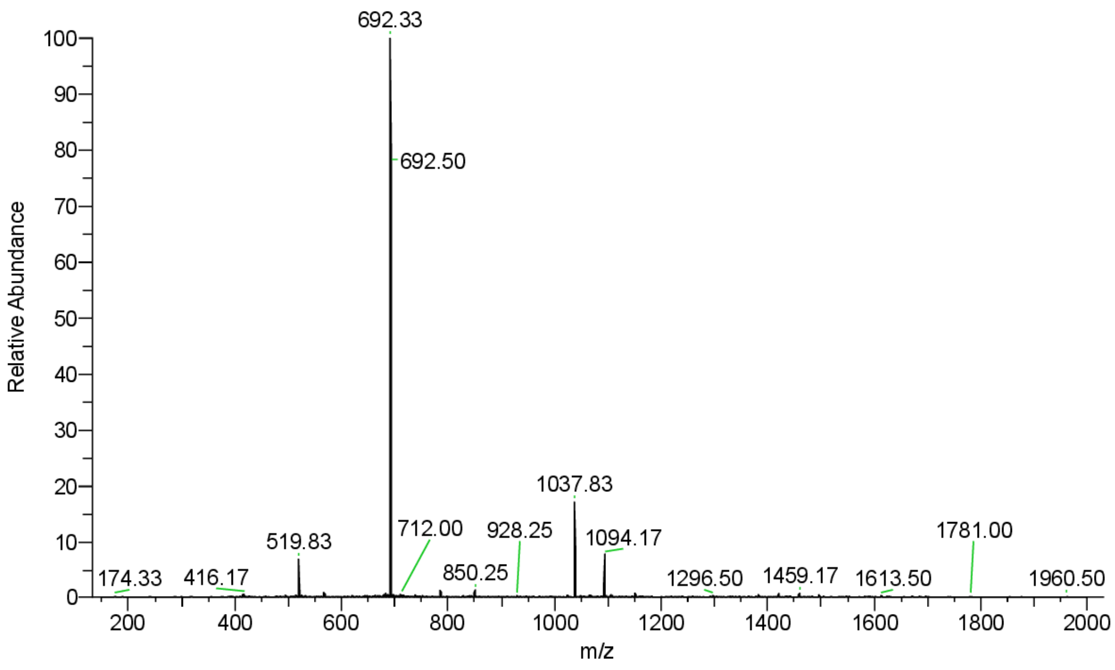

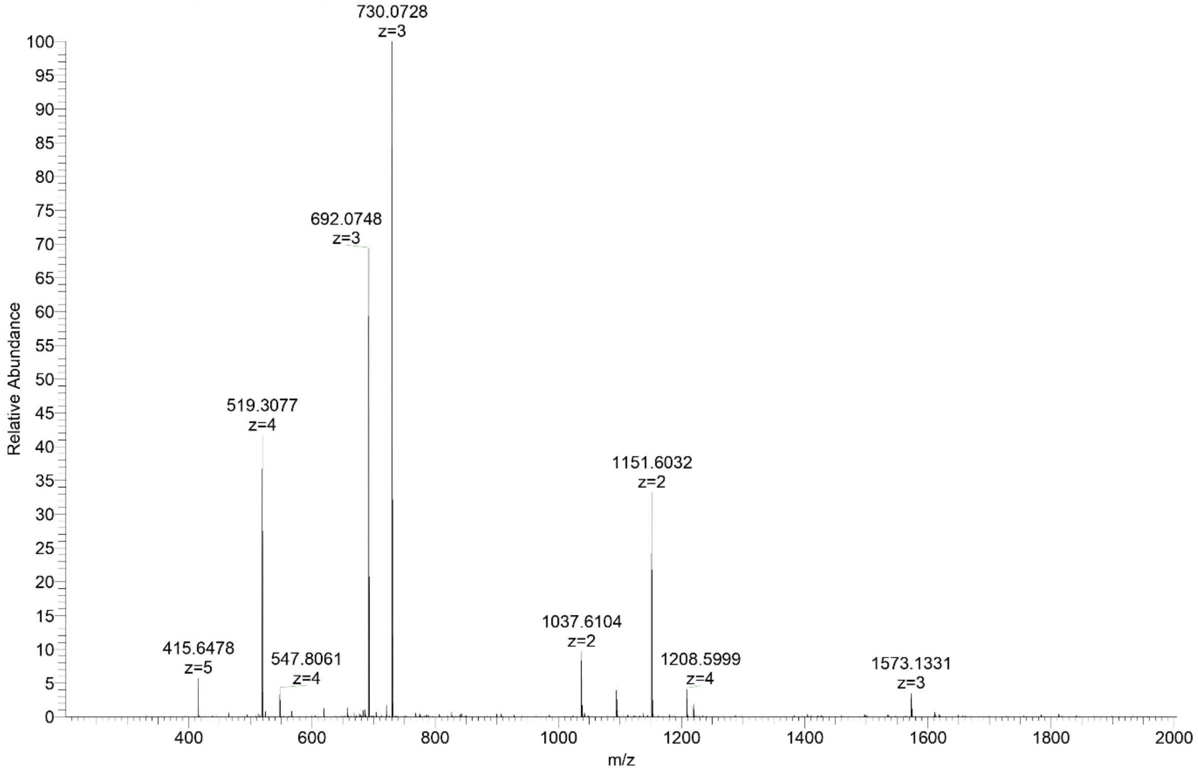
730.0728
z=3
692.0748
z=3
519.3077
z=4
1151.6032
z=2
1037.6104
z=2
415.6478
z=5
547.8061
z=4
1208.5999
z=4
1573.1331
z=3
Relative Abundance
m/z

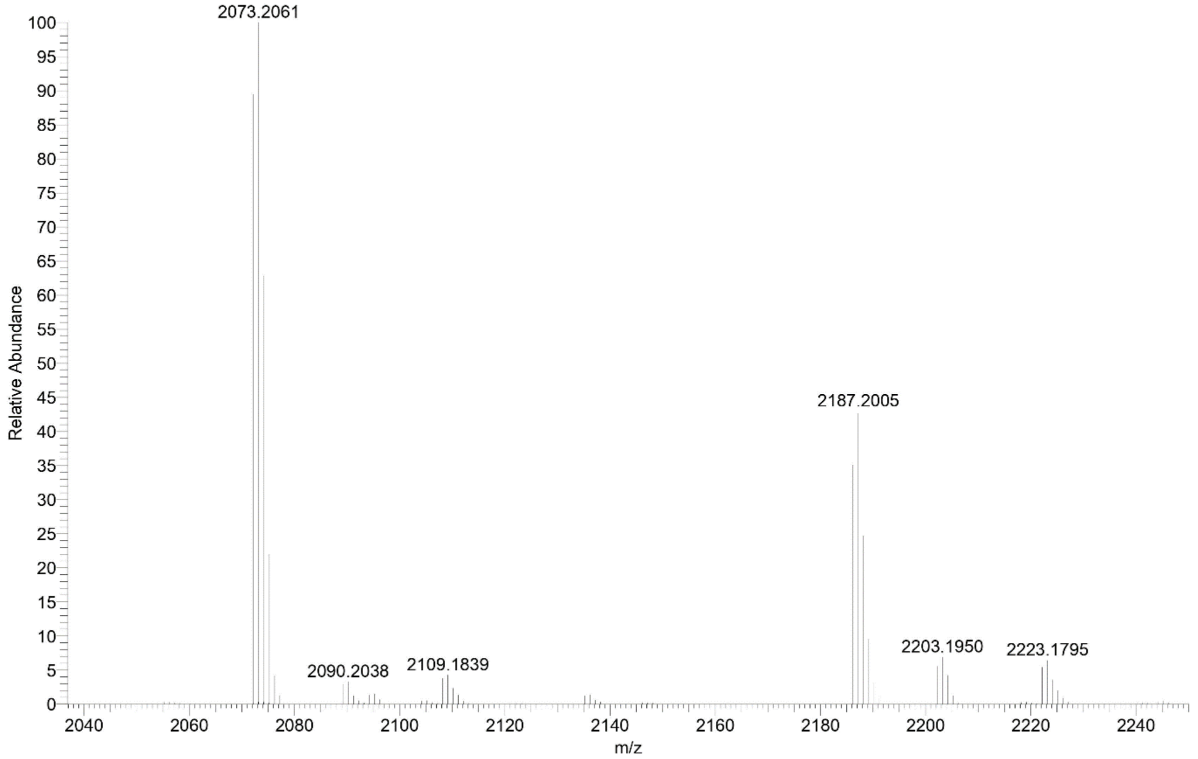
2073.2061
2187.2005
2090.2038
2109.1839
2203.1950
2223.1795
Relative Abundance
m/z

LL-III (VNWKKILGKIIKVVK-NH_2) was obtained after manual synthesis from Rink Amide AM resin LL (300 mg, 0.29 mmol/g), the peptide was obtained as a white foamy solid after preparative RP-HPLC purification (41 mg, 20.2%). Analytical RP-HPLC: t_R = 1.63 min (100% A to 100% D in 3.5 min, λ= 214 nm). MS (ESI+): $C_{86}H_{153}N_{23}O_{16}$ calc./obs. 1764.19/1764.20 $[M]^+$

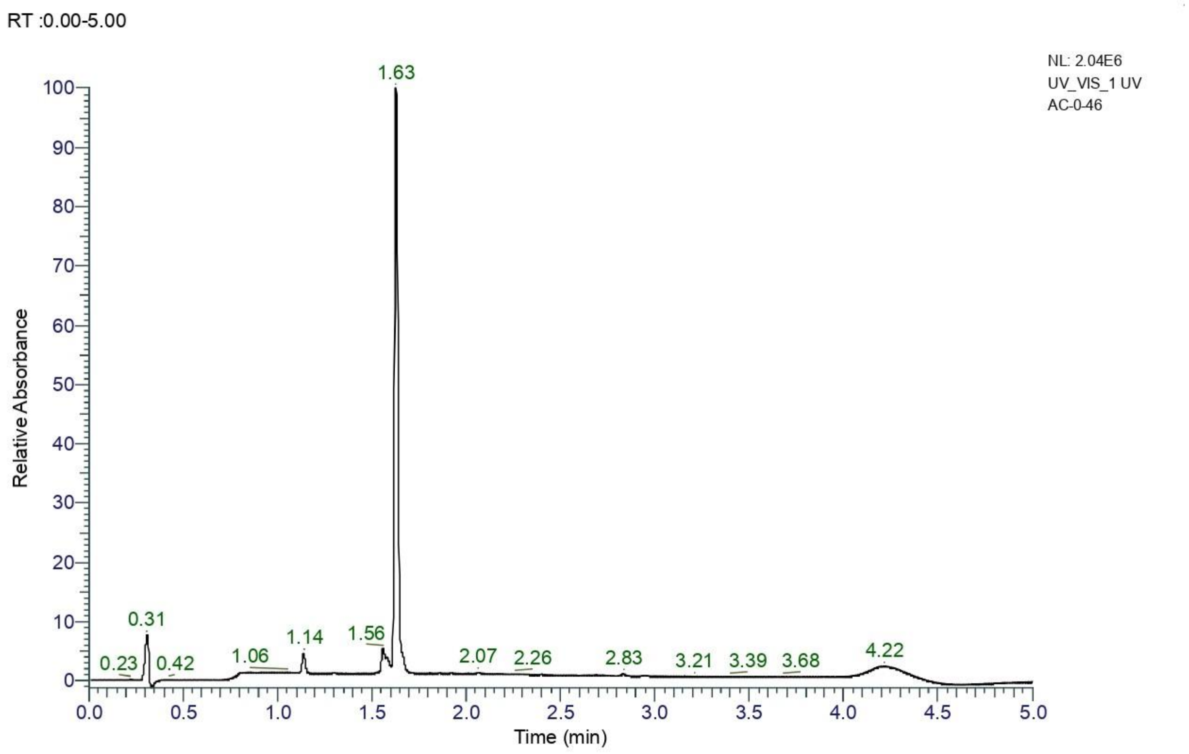

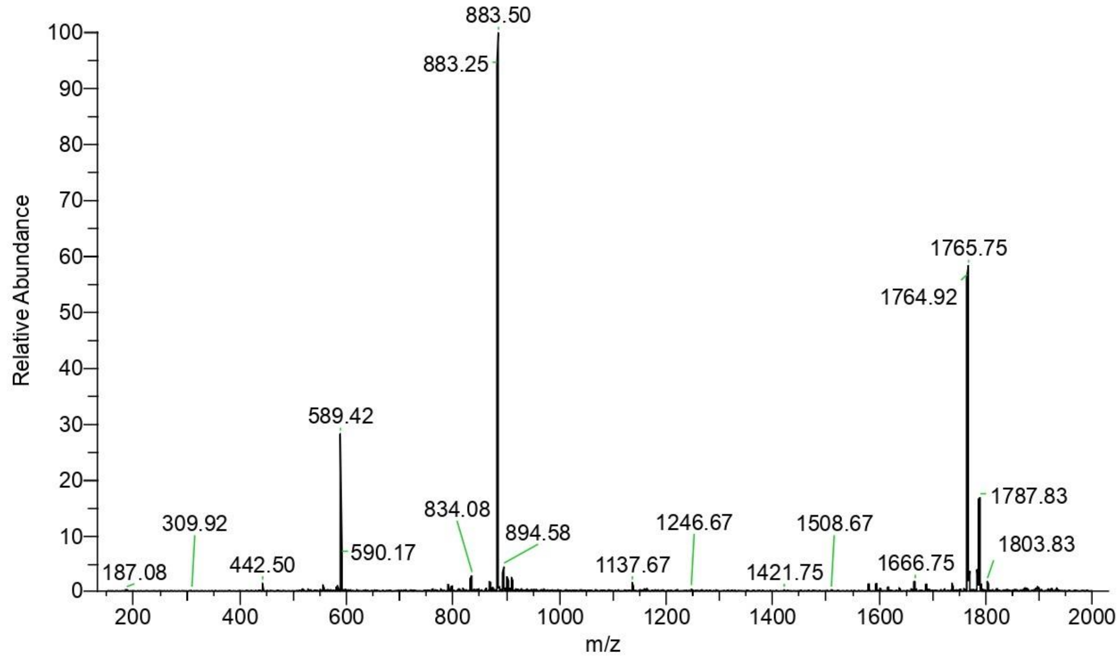

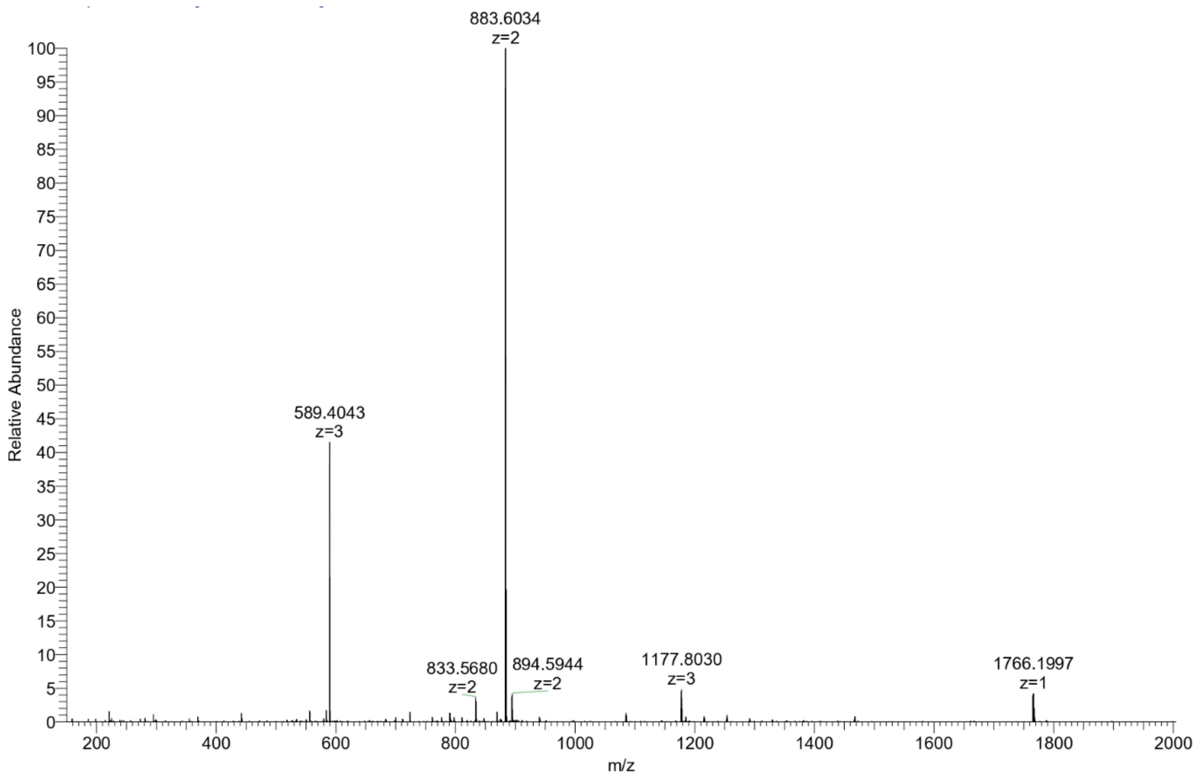
883.6034
z=2
589.4043
z=3
833.5680
z=2
894.5944
z=2
1177.8030
z=3
1766.1997
z=1
Relative Abundance
m/z

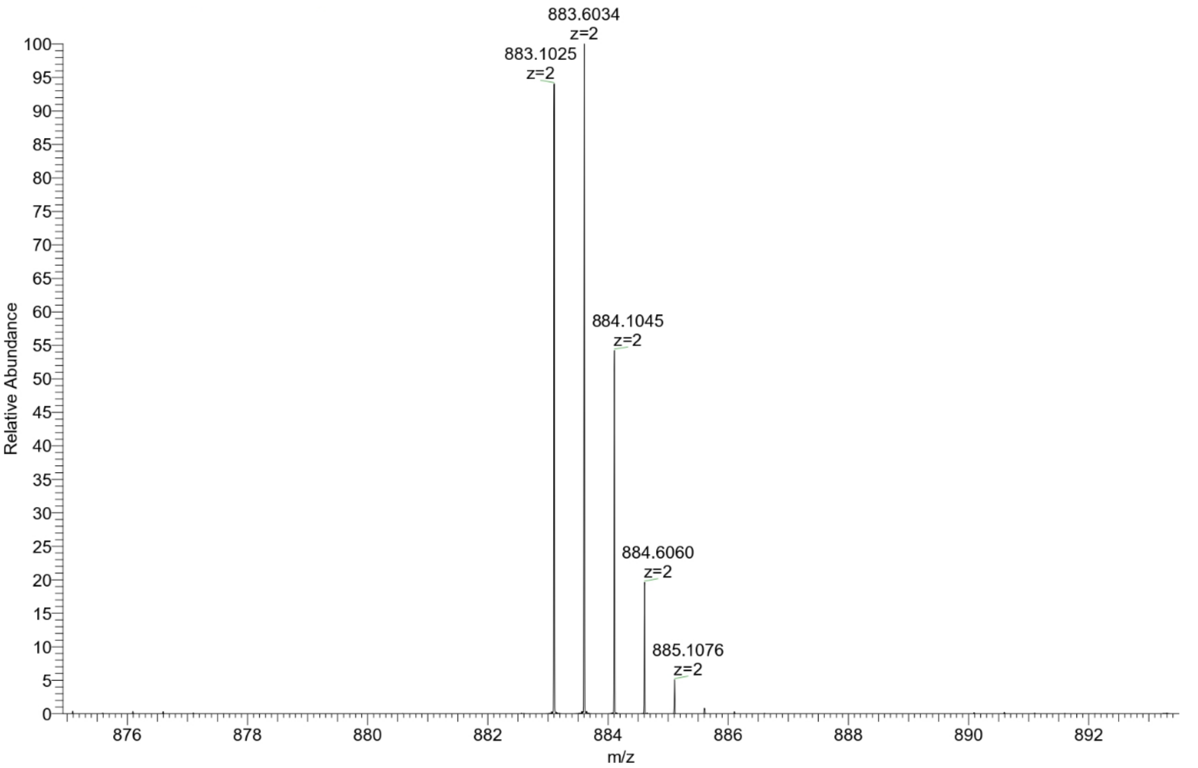
883.6034
z=2
883.1025
z=2
884.1045
z=2
884.6060
z=2
885.1076
z=2
Relative Abundance
m/z

B1 (ANWKKWIGKVIKLVK-NH_2) was obtained after manual synthesis from Rink Amide AM resin LL (300 mg, 0.29 mmol/g), the peptide was obtained as a white foamy solid after preparative RP-HPLC purification (30 mg, 14.5%). Analytical RP-HPLC: t_R = 1.56 min (100% A to 100% D in 3.5 min, λ= 214 nm). MS (ESI+): $C_{89}H_{148}N_{24}O_{16}$ calc./obs. 1809.15/1809.15 $[M]^+$

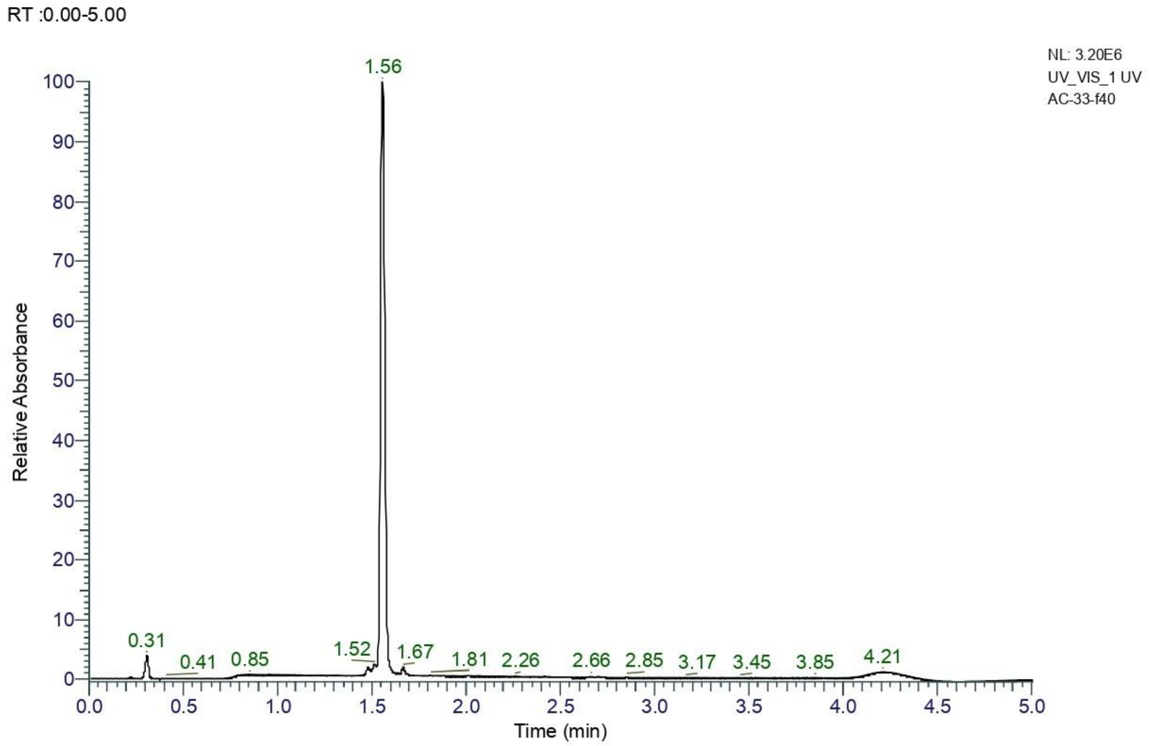

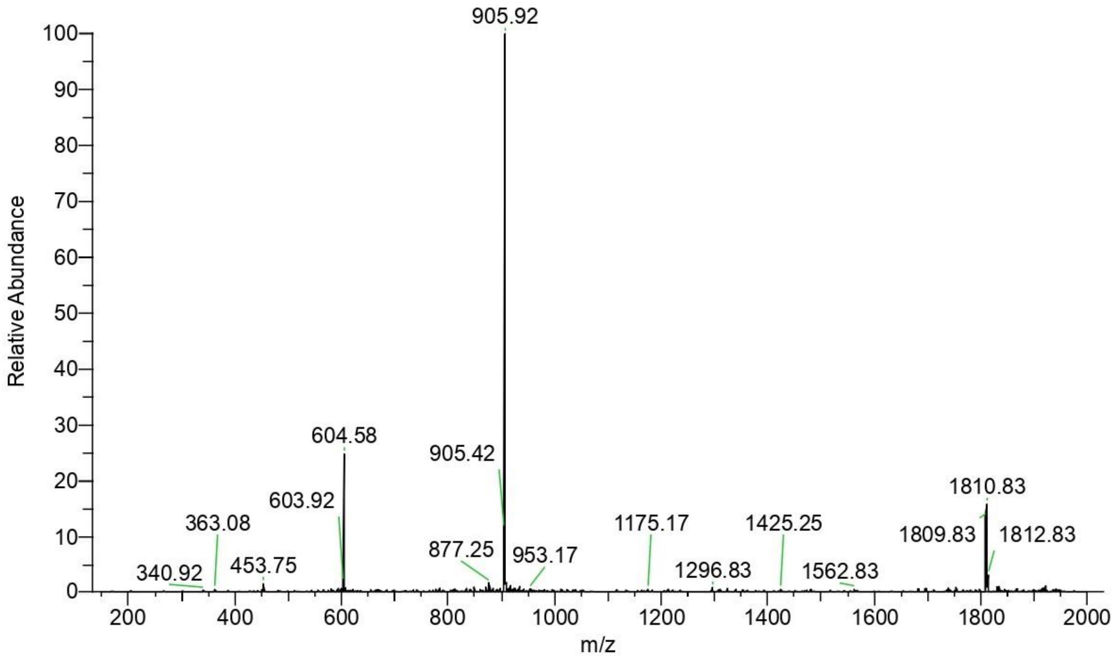

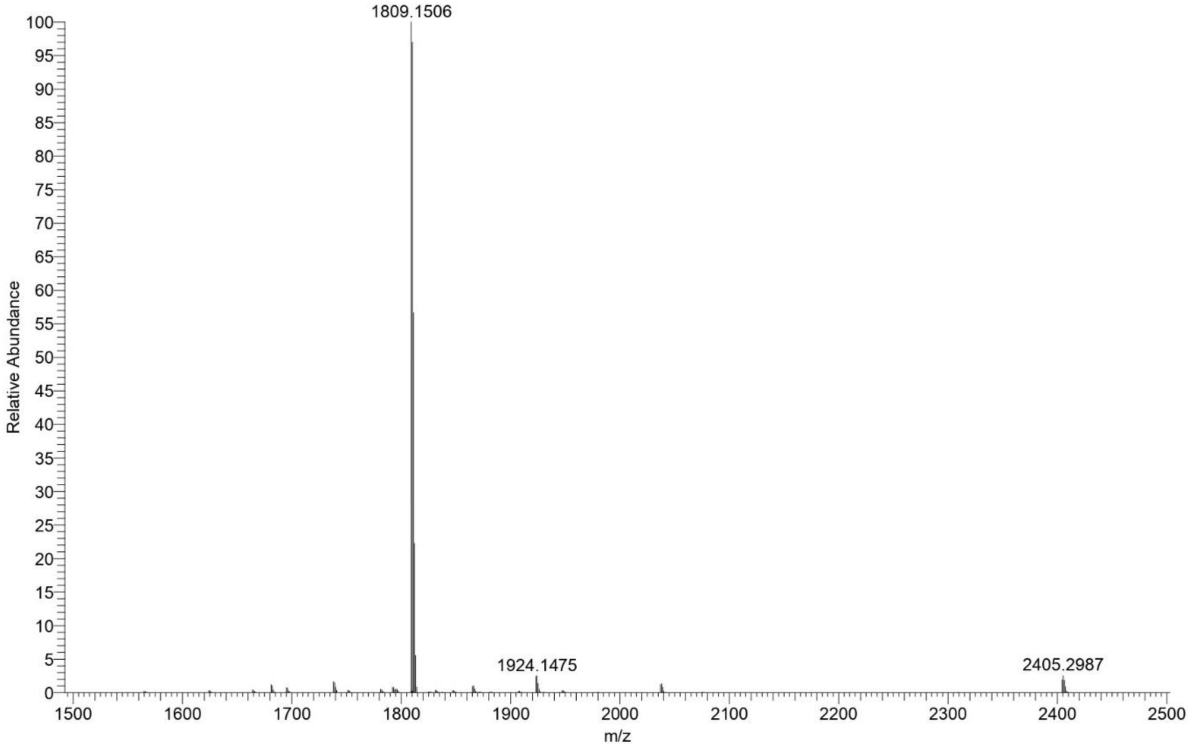
1809.1506
1924.1475
2405.2987
Relative Abundance
m/z

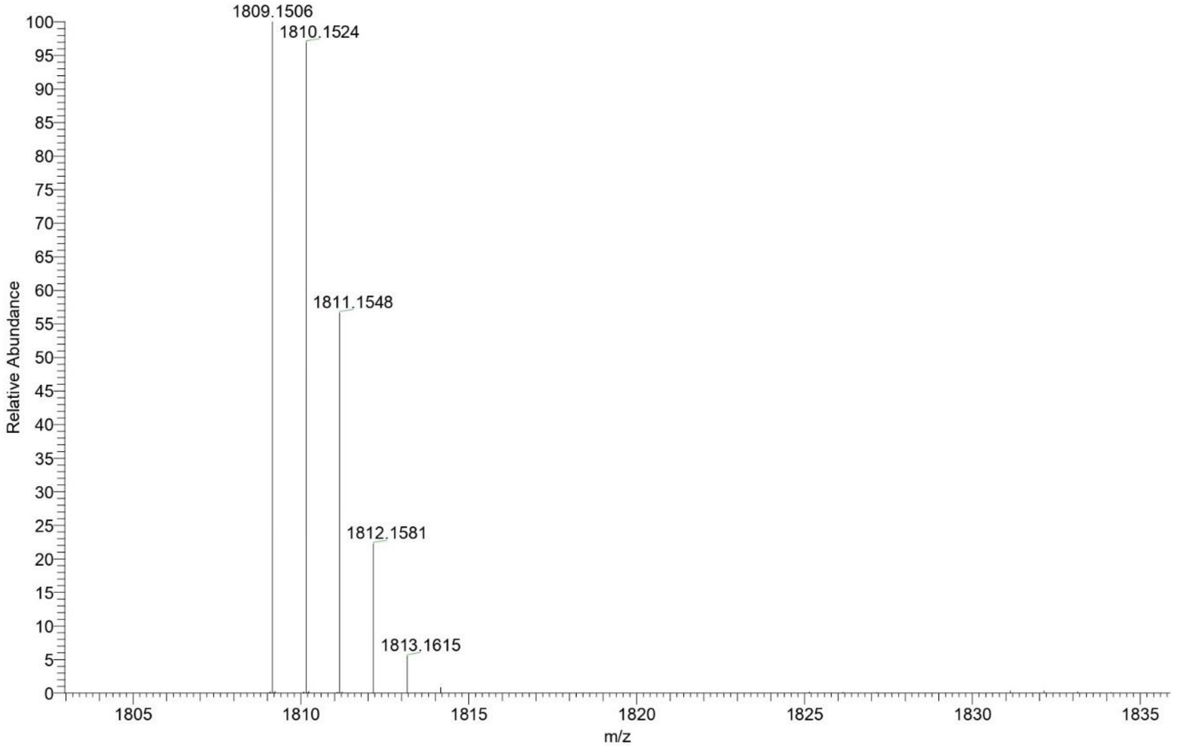
1809.1506
1810.1524
1811.1548
1812.1581
1813.1615
Relative Abundance
m/z

B2 (NWKKILGKILDHLAC-NH_2) was obtained after manual synthesis from Rink Amide AM resin LL (300 mg, 0.29 mmol/g), the peptide was obtained as a white foamy solid after preparative RP-HPLC purification (25 mg, 14.5%). Analytical RP-HPLC: t_R = 1.65 min (100% A to 100% D in 3.5 min, λ= 214 nm). MS (ESI+): $C_{81}H_{135}N_{23}O_{18}S$ calc./obs. 1750.01/1750.00 $[M]^+$

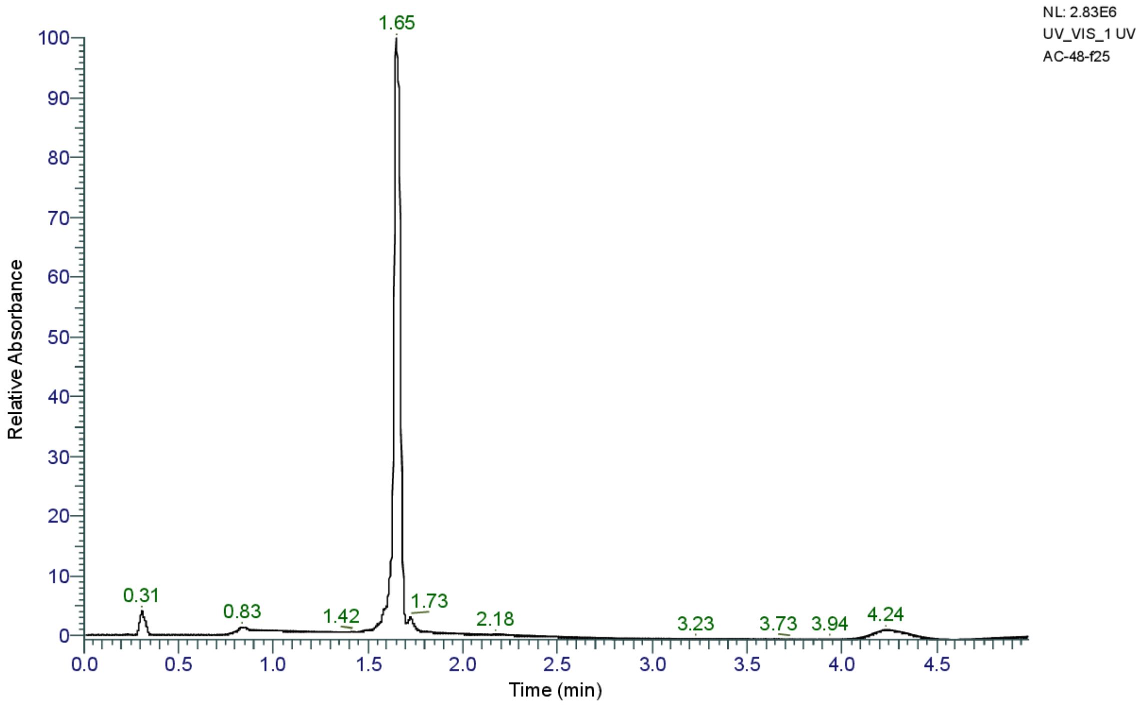

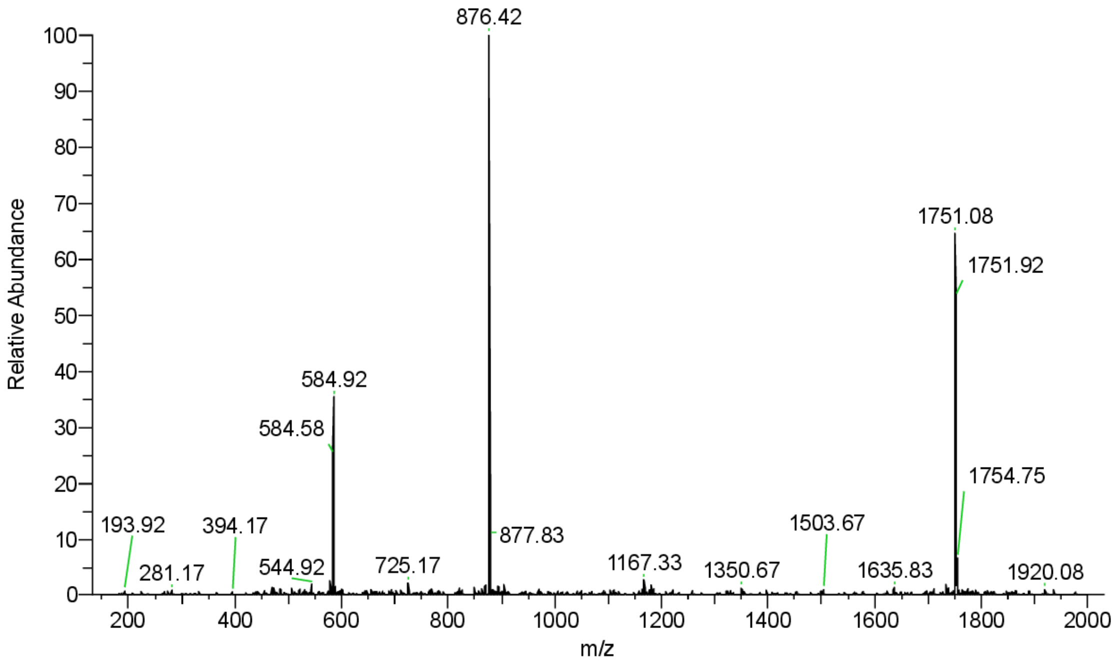

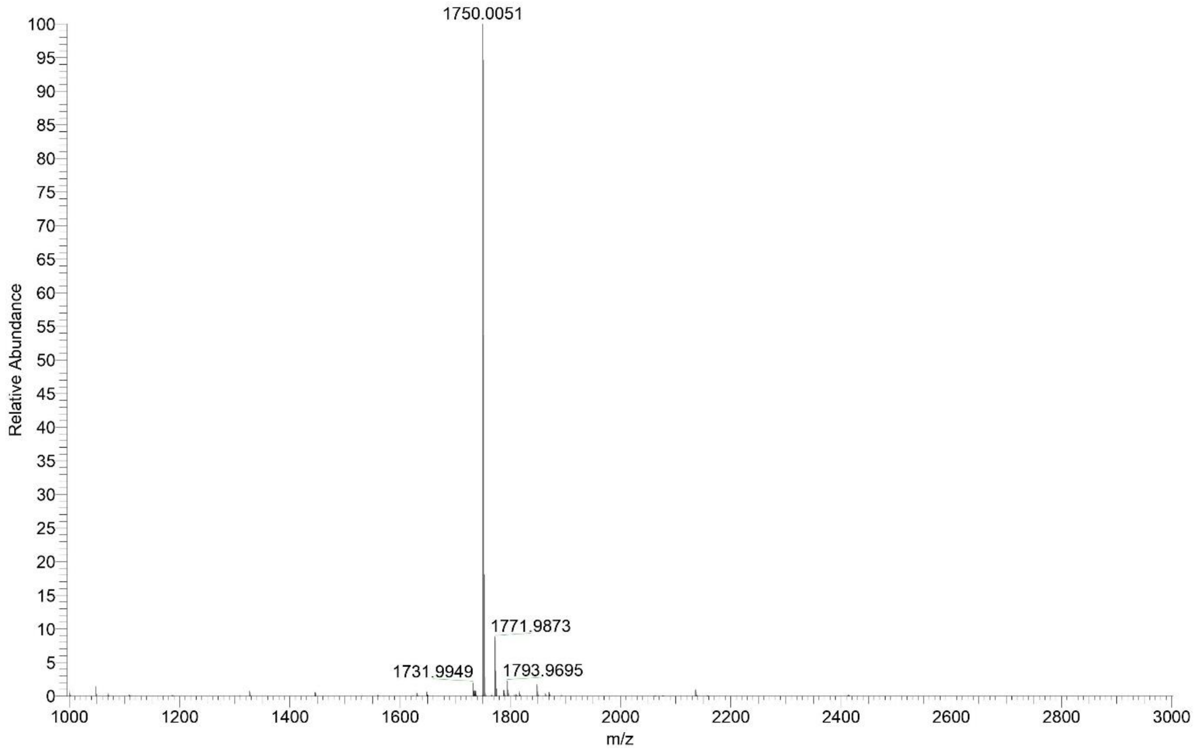
1750.0051
1771.9873
1731.9949
1793.9695
Relative Abundance
m/z

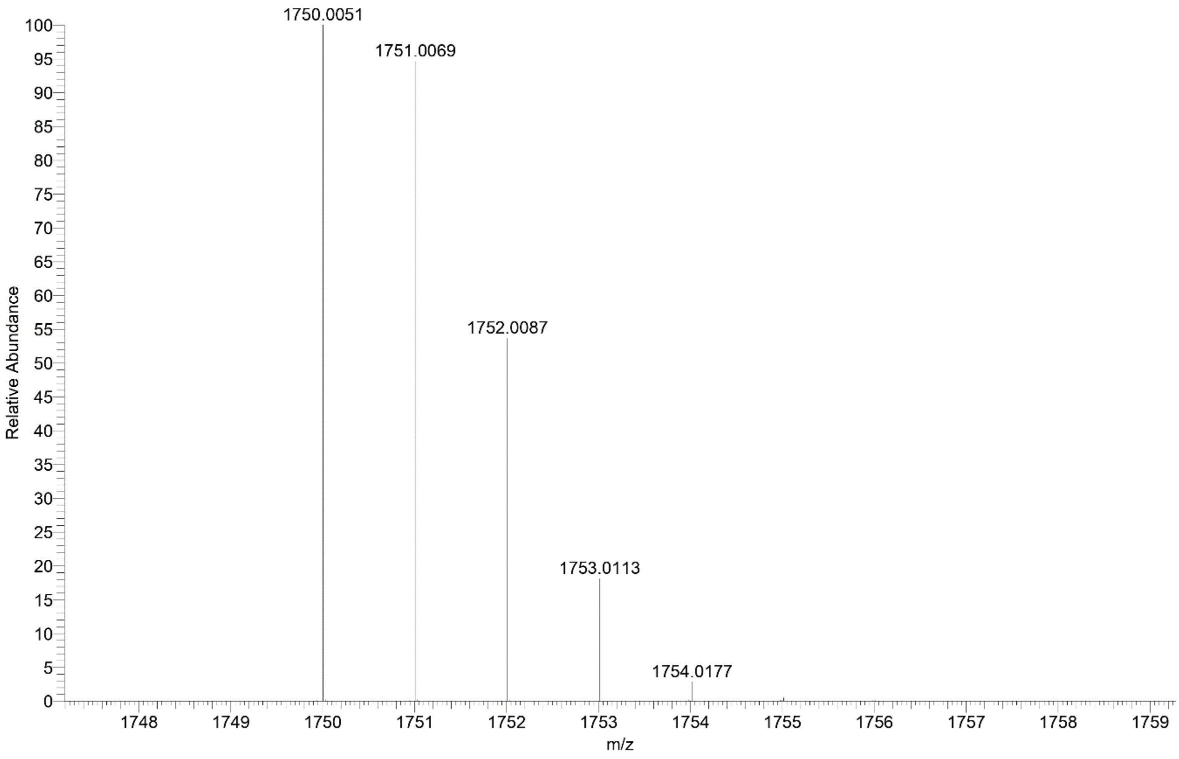
1750.0051
1751.0069
1752.0087
1753.0113
1754.0177
Relative Abundance
m/z

B3 (ANWKKILKRLCDI-NH_2) was obtained after manual synthesis from Rink Amide AM resin LL (300 mg, 0.29 mmol/g), the peptide was obtained as a white foamy solid after preparative RP-HPLC purification (51 mg, 30.2%). Analytical RP-HPLC: t_R = 1.57 min (100% A to 100% D in 3.5 min, λ= 214 nm). MS (ESI+): $C_{73}H_{126}N_{22}O_{16}S$ calc./obs. 1598.94/1598.94 $[M]^+$

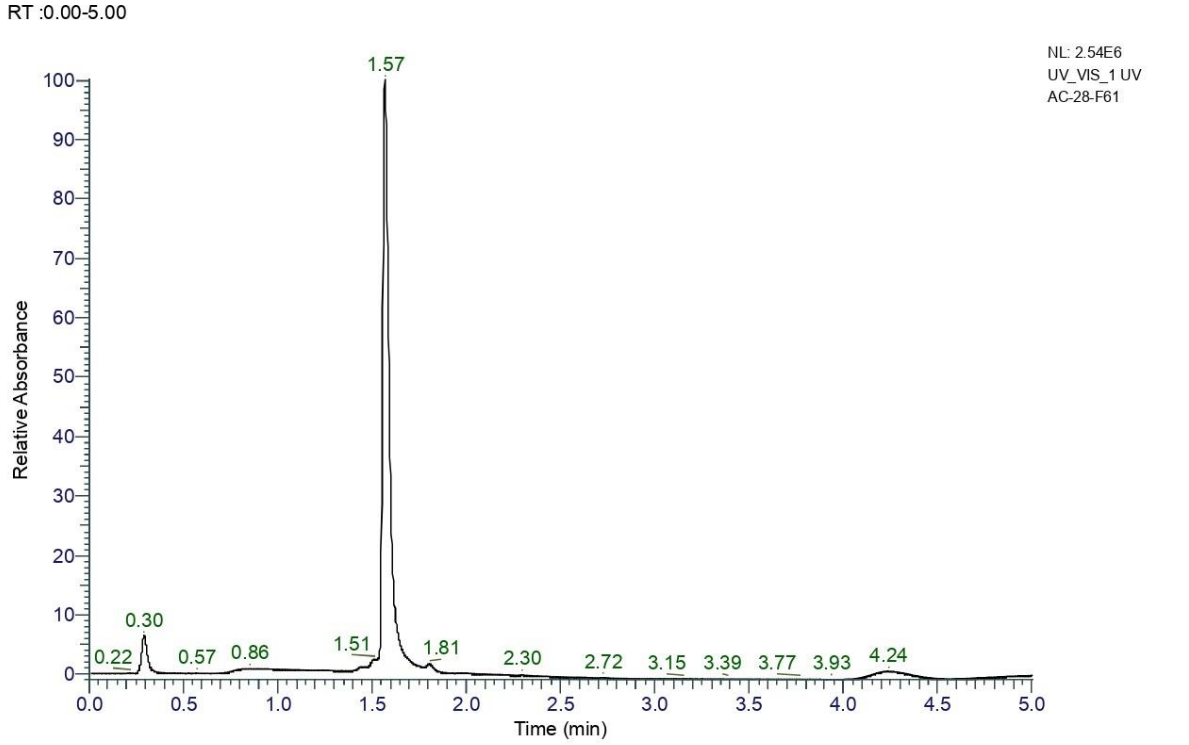

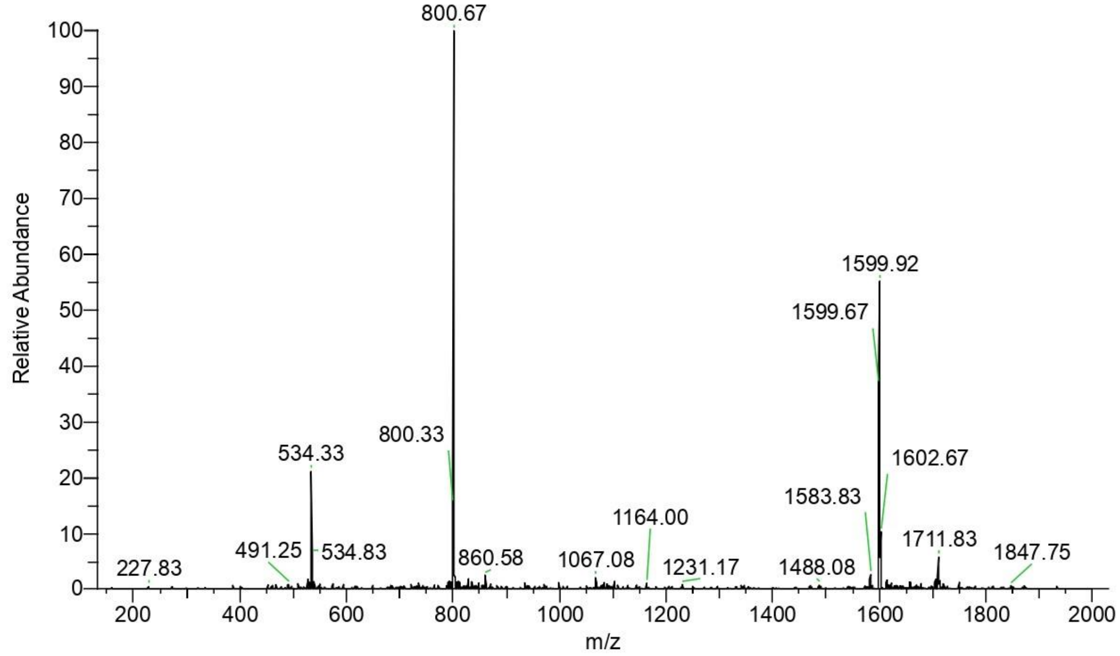

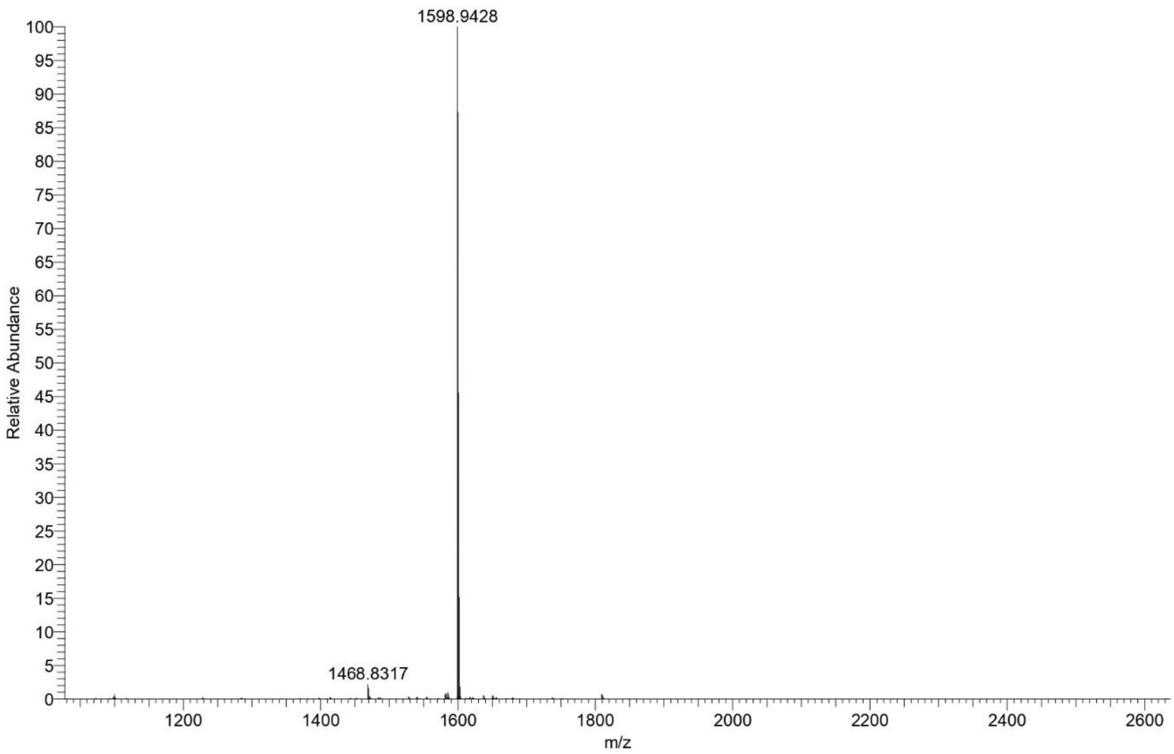
1598.9428
1468.8317
Relative Abundance
m/z

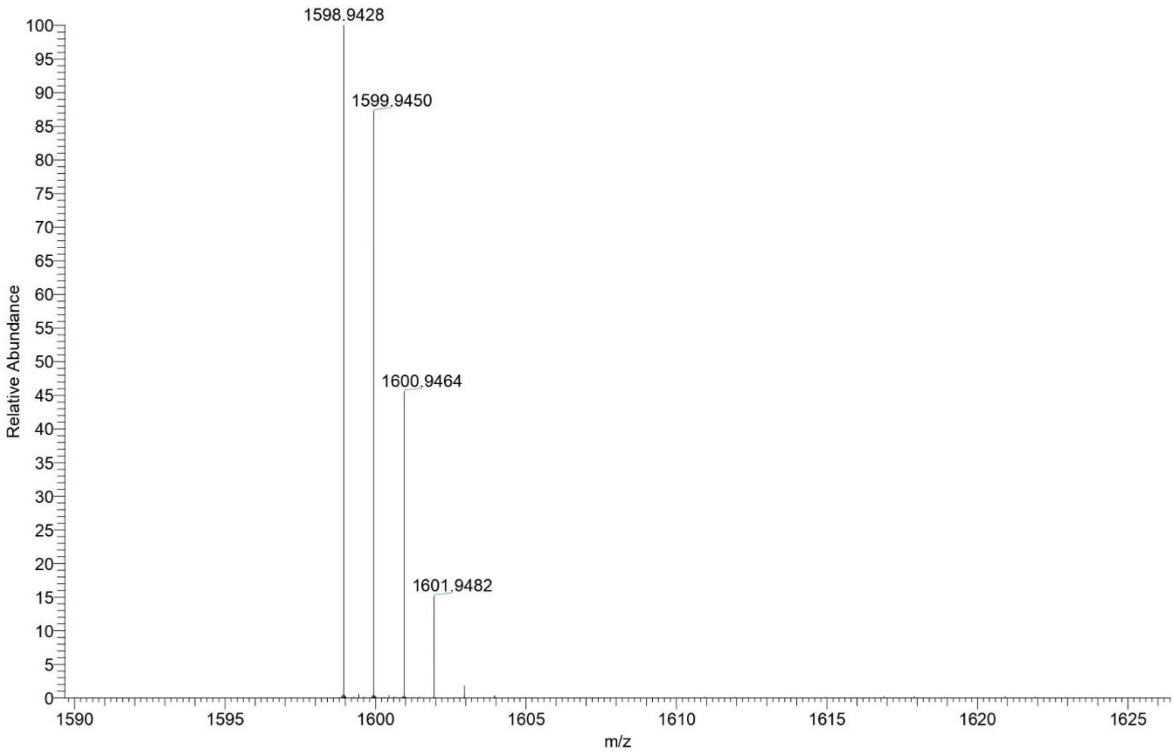
1598.9428
1599.9450
1600.9464
1601.9482
Relative Abundance
m/z

B4 (NWKKILGKICR-NH_2) was obtained after manual synthesis from Rink Amide AM resin LL (300 mg, 0.29 mmol/g), the peptide was obtained as a white foamy solid after preparative RP-HPLC purification (22 mg, 14.0%). Analytical RP-HPLC: t_R = 1.42 min (100% A to 100% D in 3.5 min, λ= 214 nm). MS (ESI+): $C_{62}H_{108}N_{20}O_{12}S$ calc./obs. 1356.82/1356.82 $[M]^+$

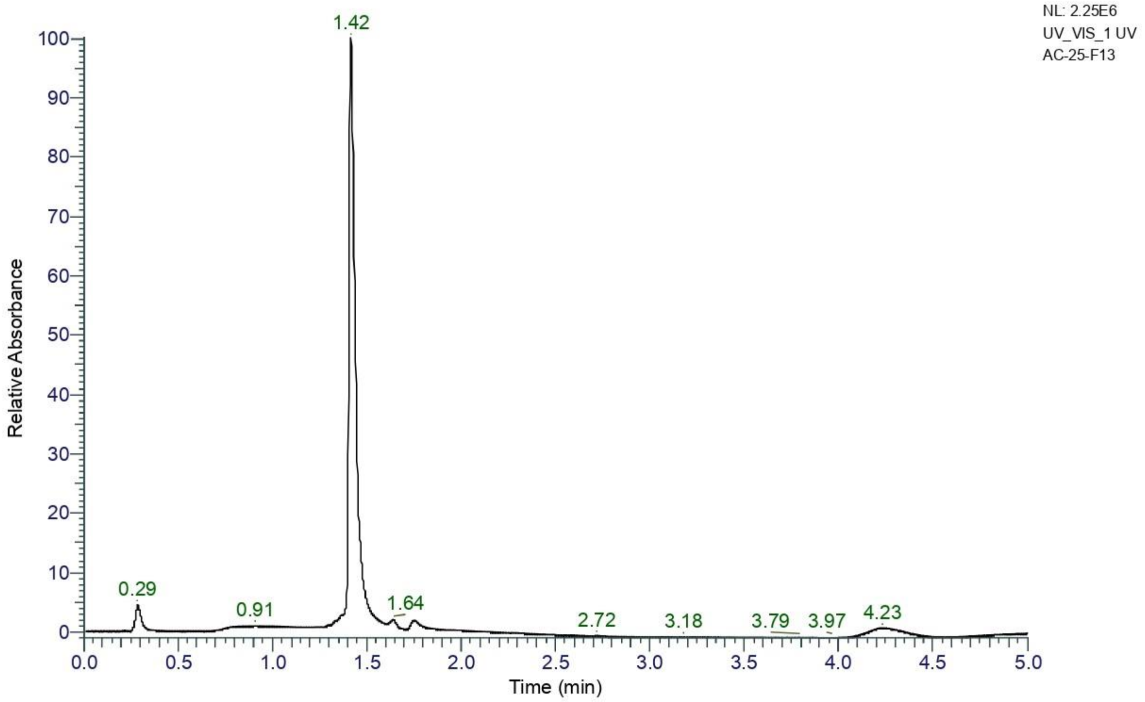

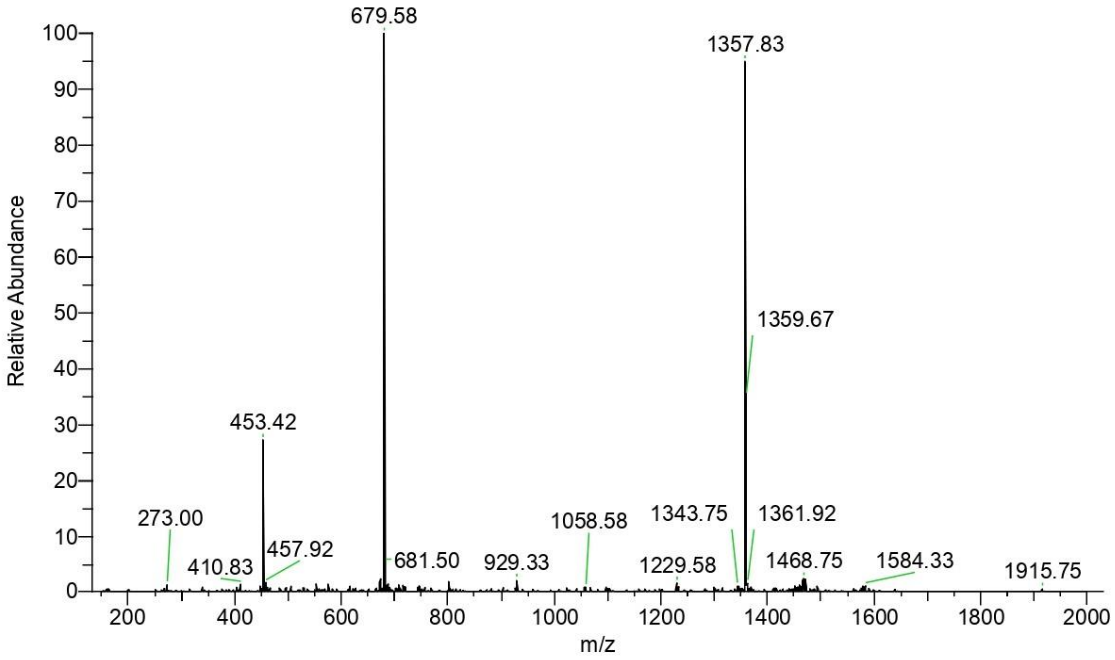

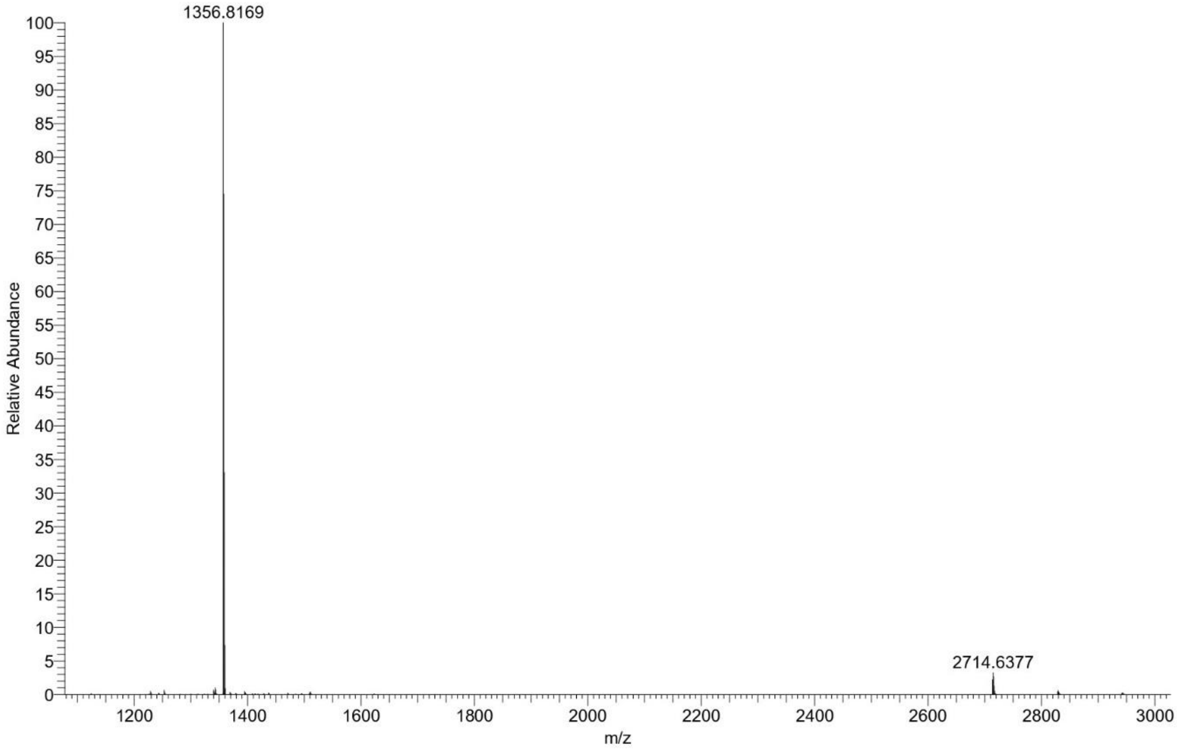
1356.8169
2714.6377
Relative Abundance
m/z
100
95
90
85
80
75
70
65
60
55
50
45
40
35
30
25
20
15
10
5
0
1200
1400
1600
1800
2000
2200
2400
2600
2800
3000

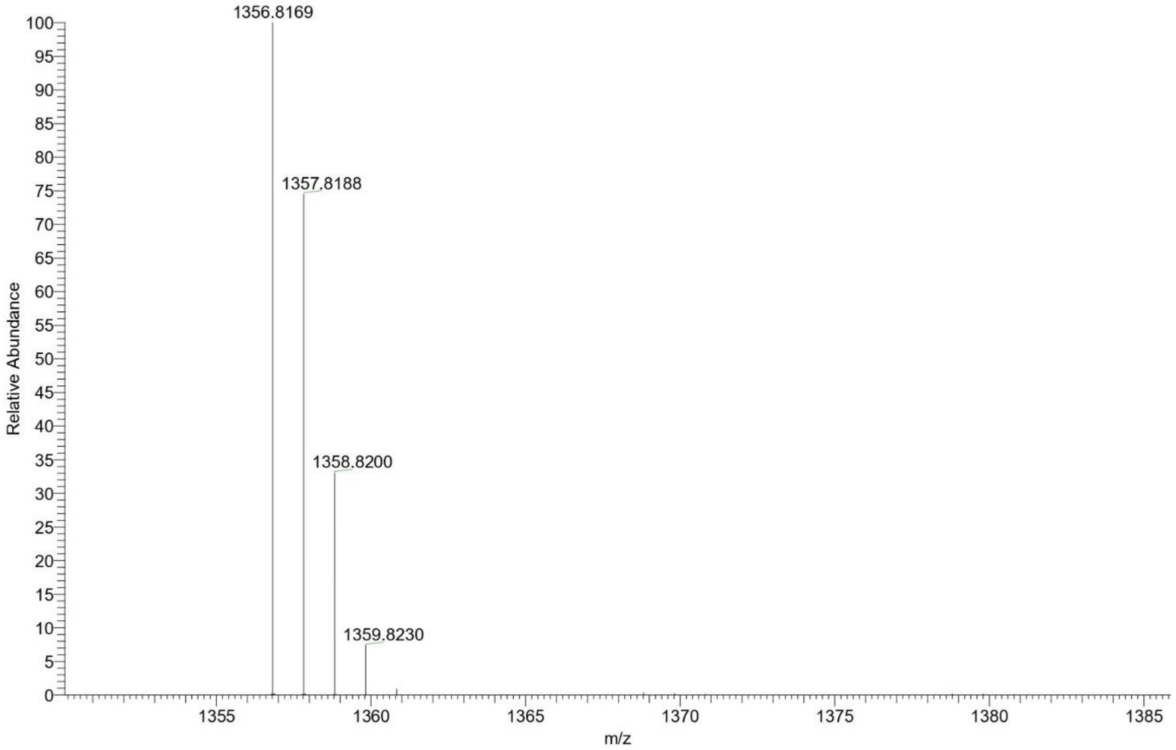
1356.8169
1357.8188
1358.8200
1359.8230
Relative Abundance
m/z
1355
1360
1365
1370
1375
1380
1385

B5 (KNWKKIIKKVVK-NH_2) was obtained after manual synthesis from Rink Amide AM resin LL (300 mg, 0.29 mmol/g), the peptide was obtained as a white foamy solid after preparative RP-HPLC purification (37 mg, 19.4%). Analytical RP-HPLC: t_R = 1.26 min (100% A to 100% D in 3.5 min, λ= 214 nm). MS (ESI+): $C_{73}H_{131}N_{21}O_{13}$ calc./obs. 1510.02/1510.02 $[M]^+$

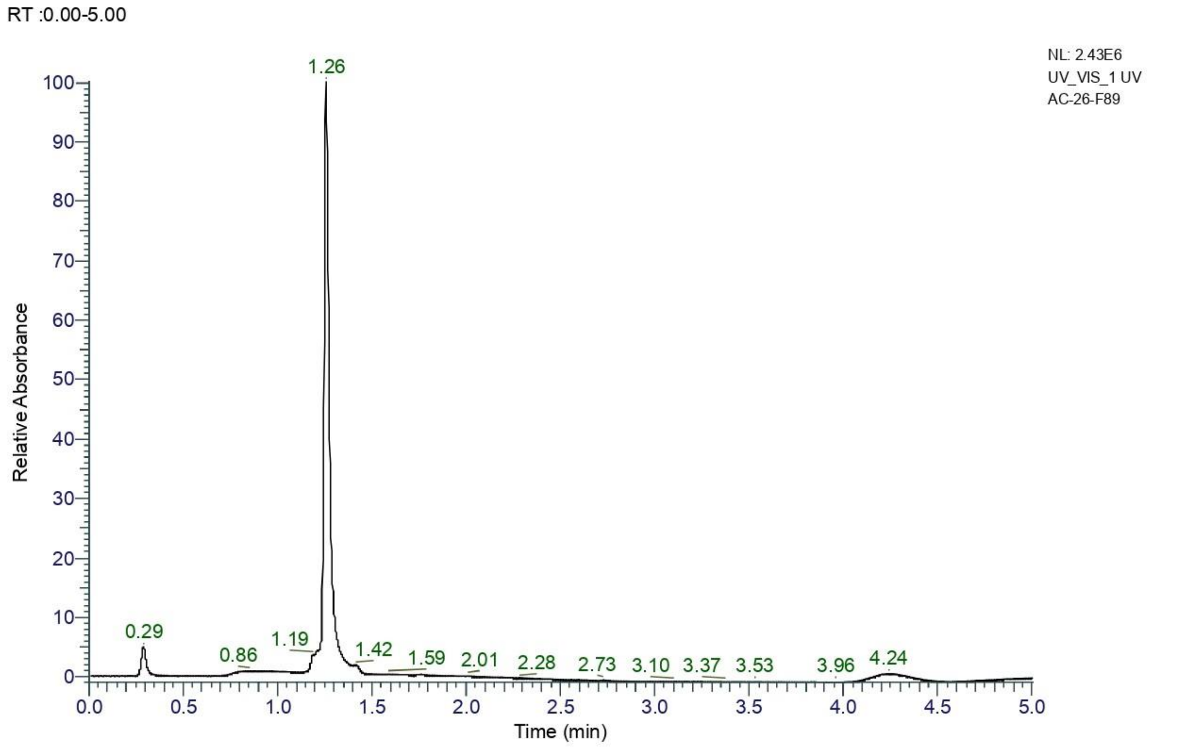

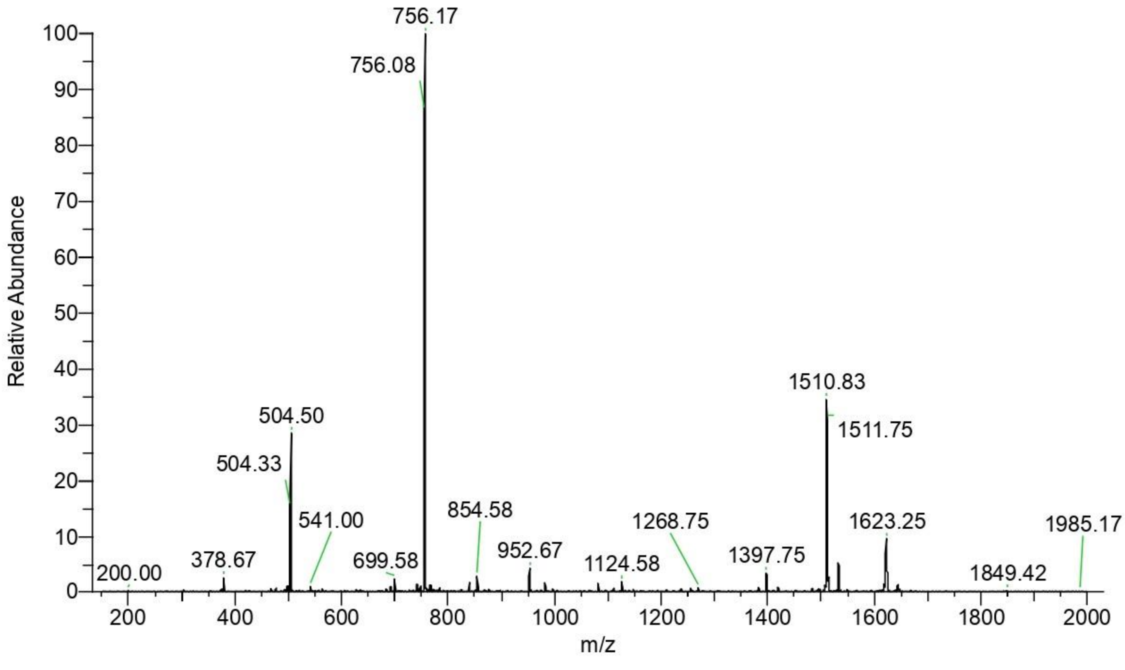

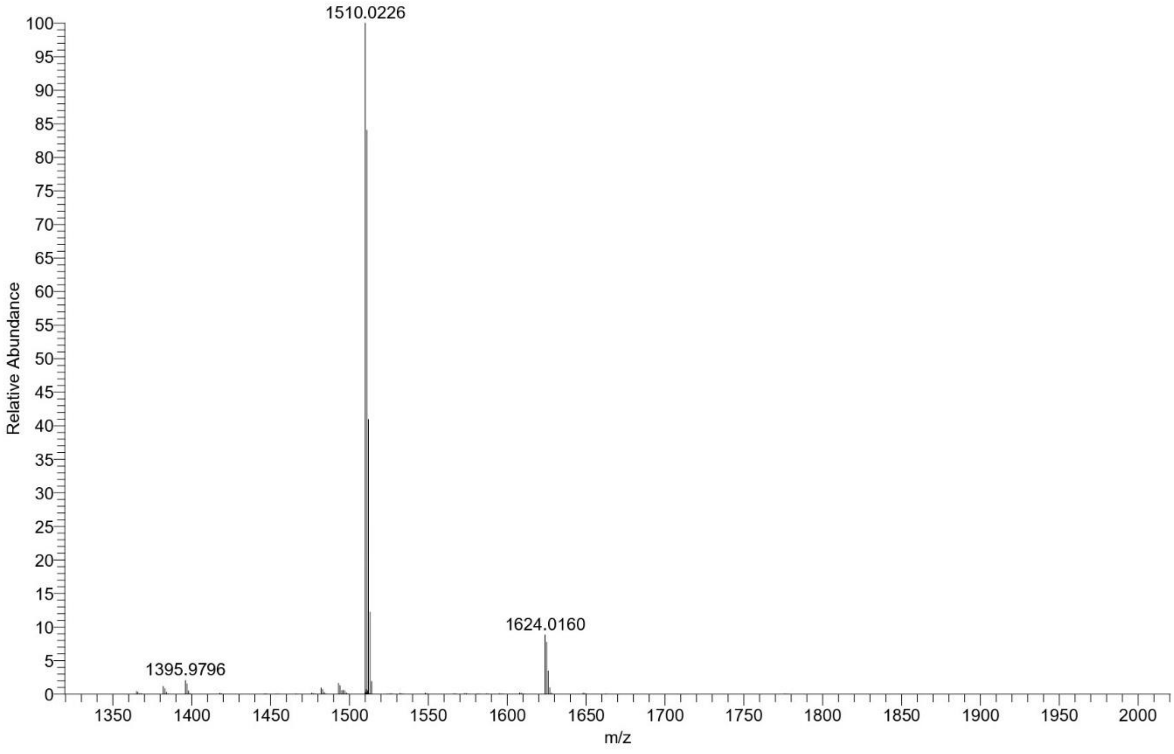
1510.0226
1624.0160
1395.9796
Relative Abundance
m/z

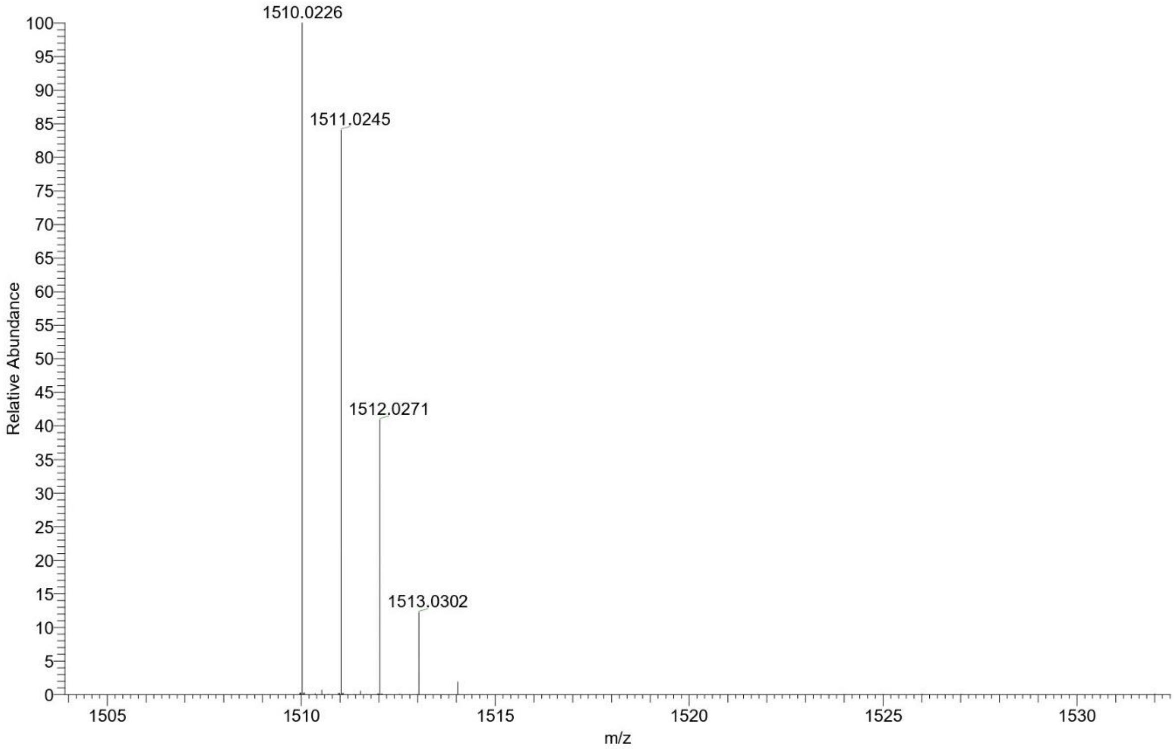
1510.0226
1511.0245
1512.0271
1513.0302
Relative Abundance
m/z

B6 (VNVWKKIGRLVKIVK-NH_2) was obtained after manual synthesis from Rink Amide AM resin LL (300 mg, 0.29 mmol/g), the peptide was obtained as a white foamy solid after preparative RP-HPLC purification (36 mg, 17.6%). Analytical RP-HPLC: t_R = 1.47 min (100% A to 100% D in 3.5 min, λ= 214 nm). MS (ESI+): $C_{85}H_{151}N_{25}O_{16}$ calc./obs. 1778.18/1778.17 $[M]^+$

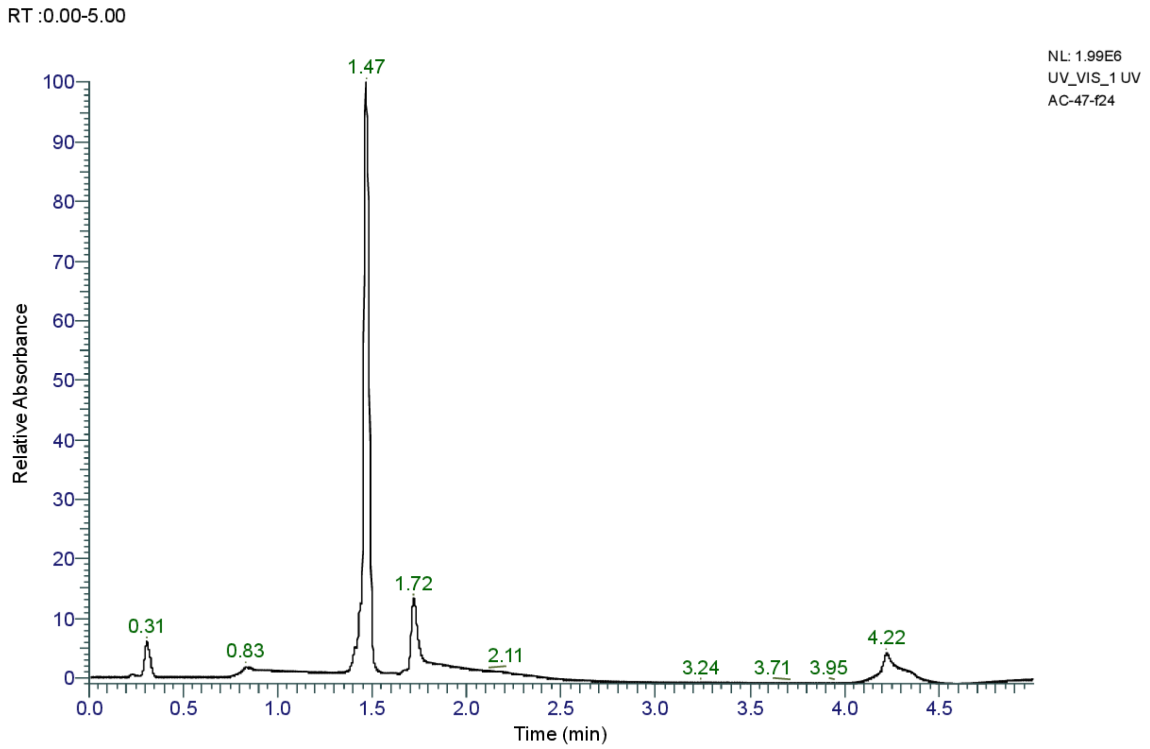

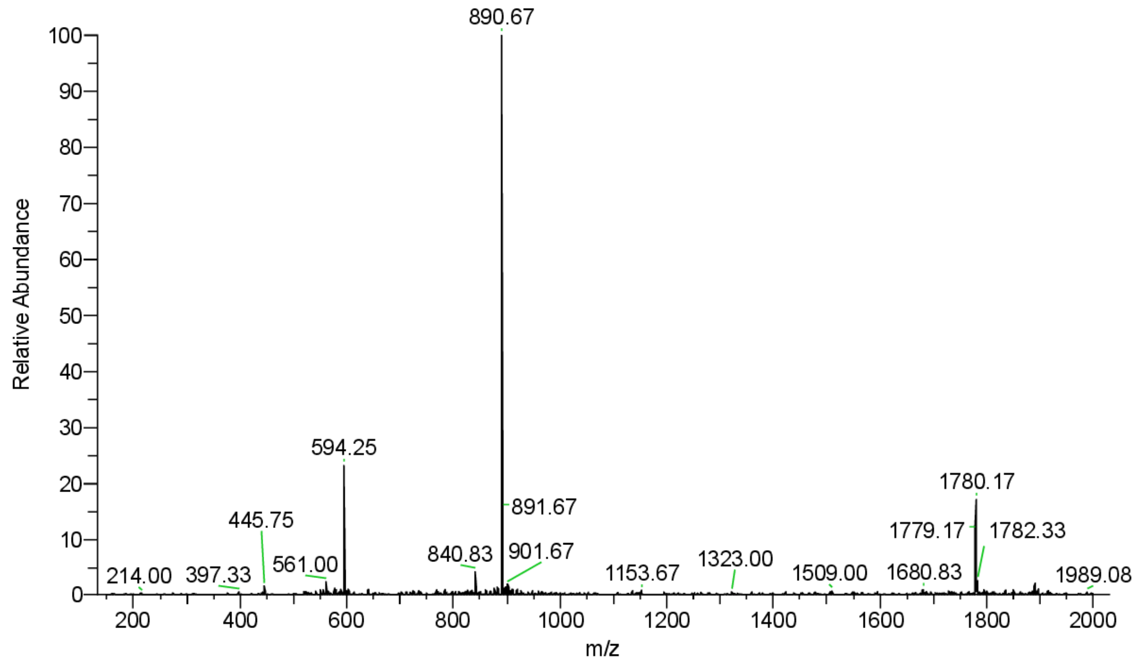

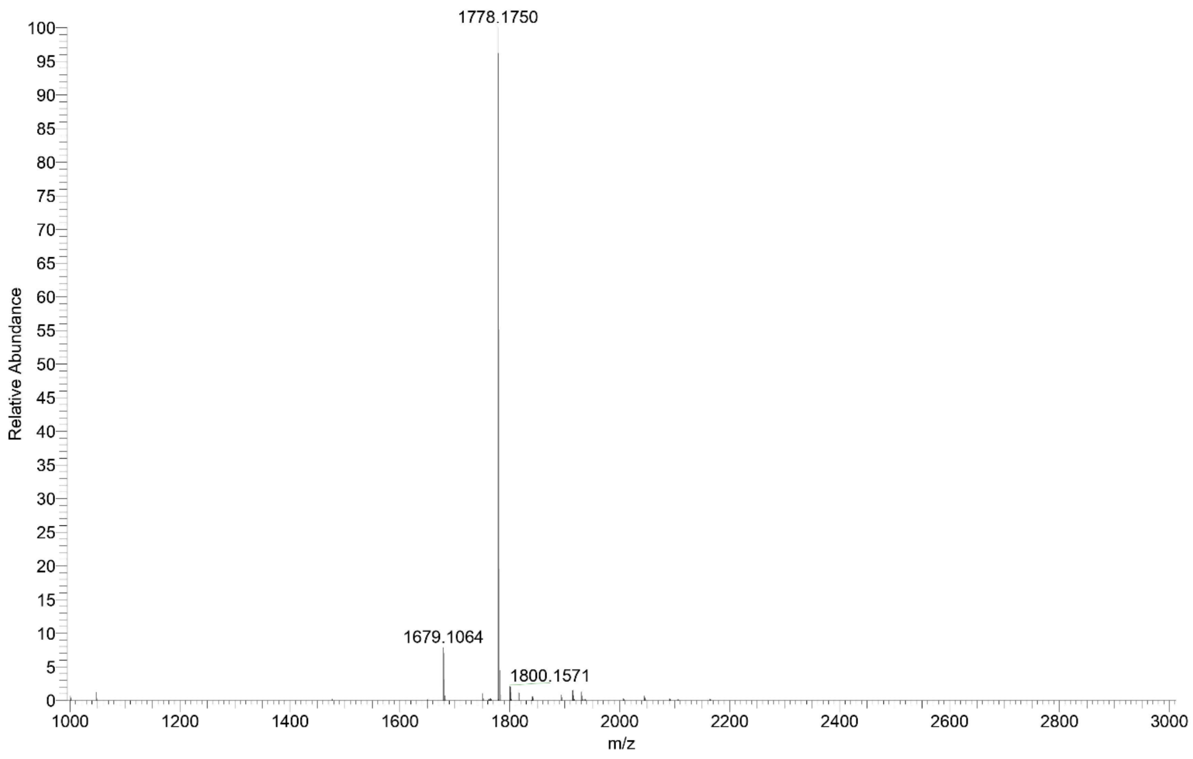
1778.1750
1679.1064
1800.1571
Relative Abundance
m/z

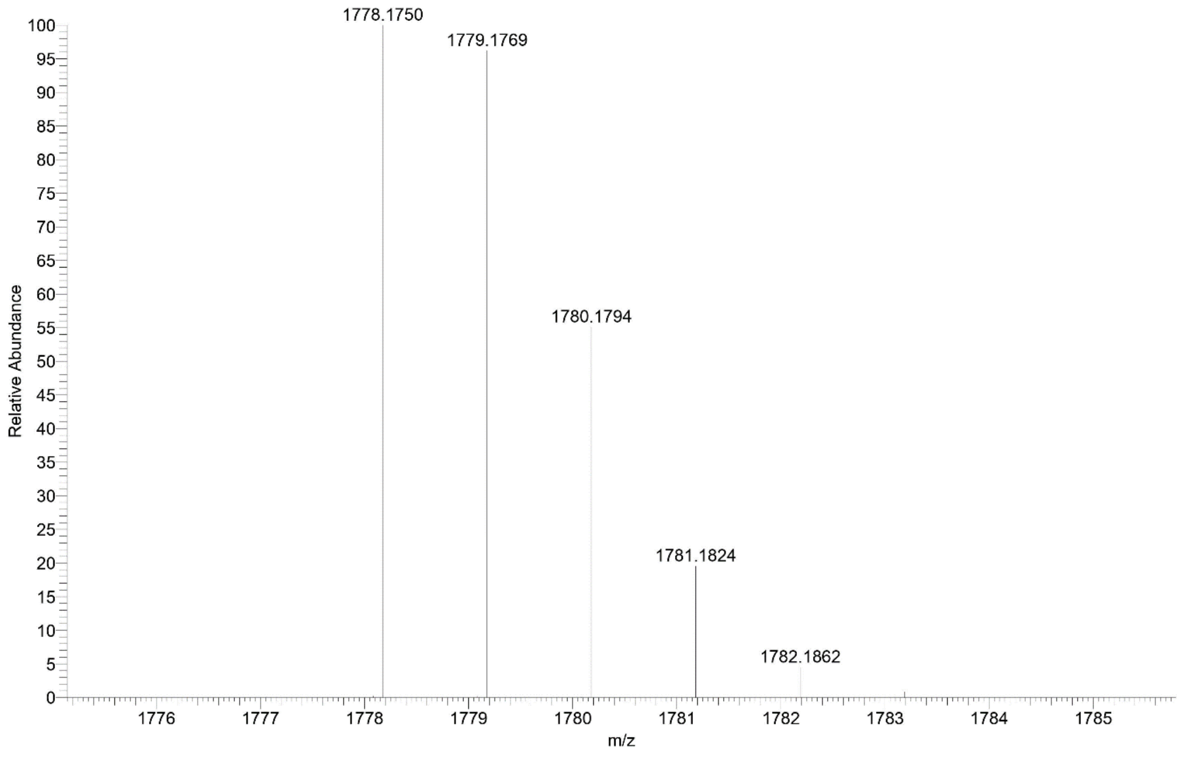
1778.1750
1779.1769
1780.1794
1781.1824
1782.1862
Relative Abundance
m/z

B7 (NEWKKIKKIIKIVK-NH$_2$) was obtained after manual synthesis from Rink Amide AM resin LL (300 mg, 0.29 mmol/g), the peptide was obtained as a white foamy solid after preparative RP-HPLC purification (54 mg, 26.6%). Analytical RP-HPLC: t_R = 1.37 min (100% A to 100% D in 3.5 min, λ= 214 nm). MS (ESI+): $C_{85}H_{151}N_{23}O_{17}$ calc./obs. 1766.17/1766.17 $[M]^+$

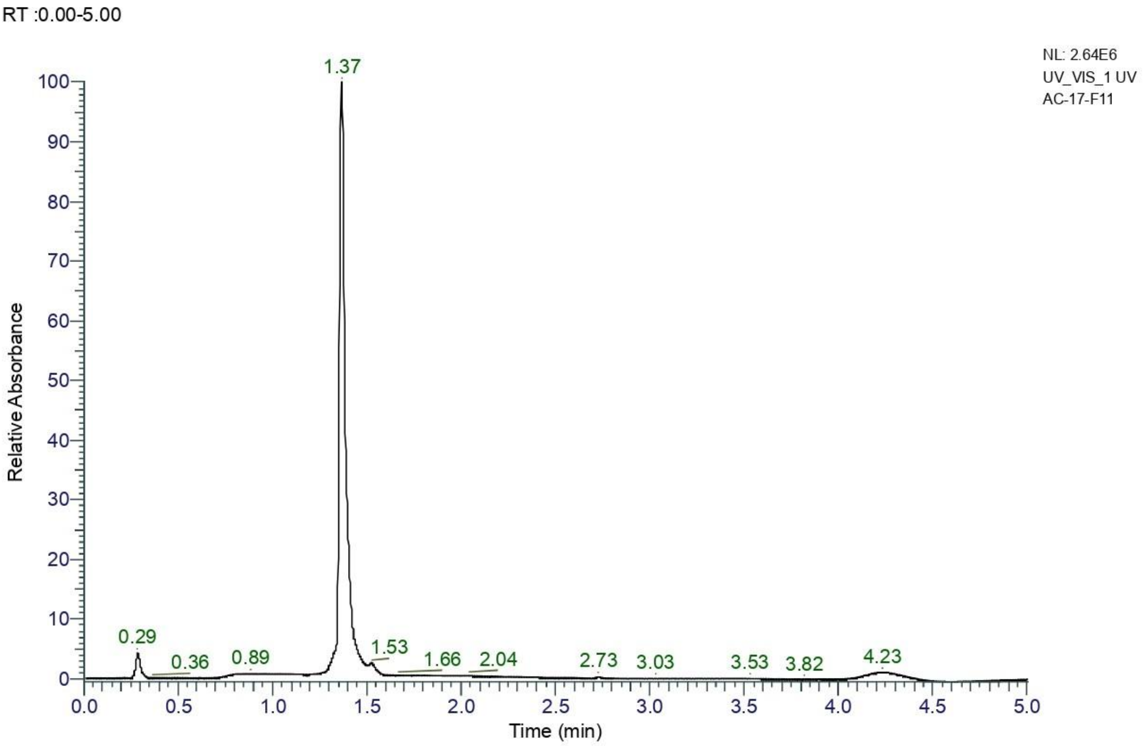

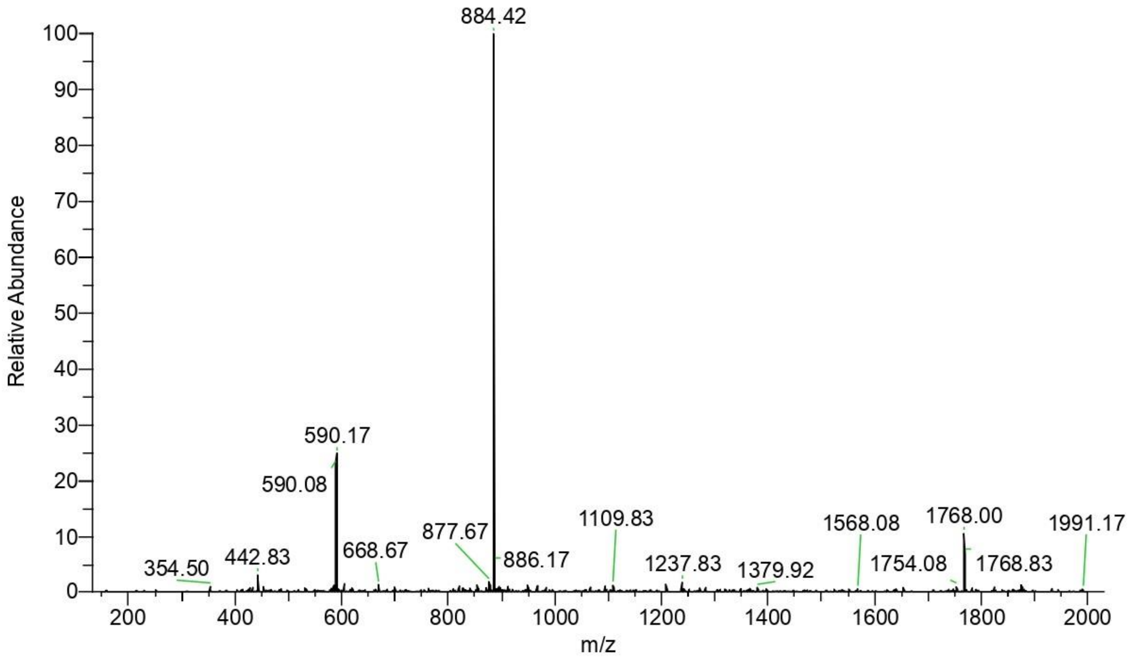

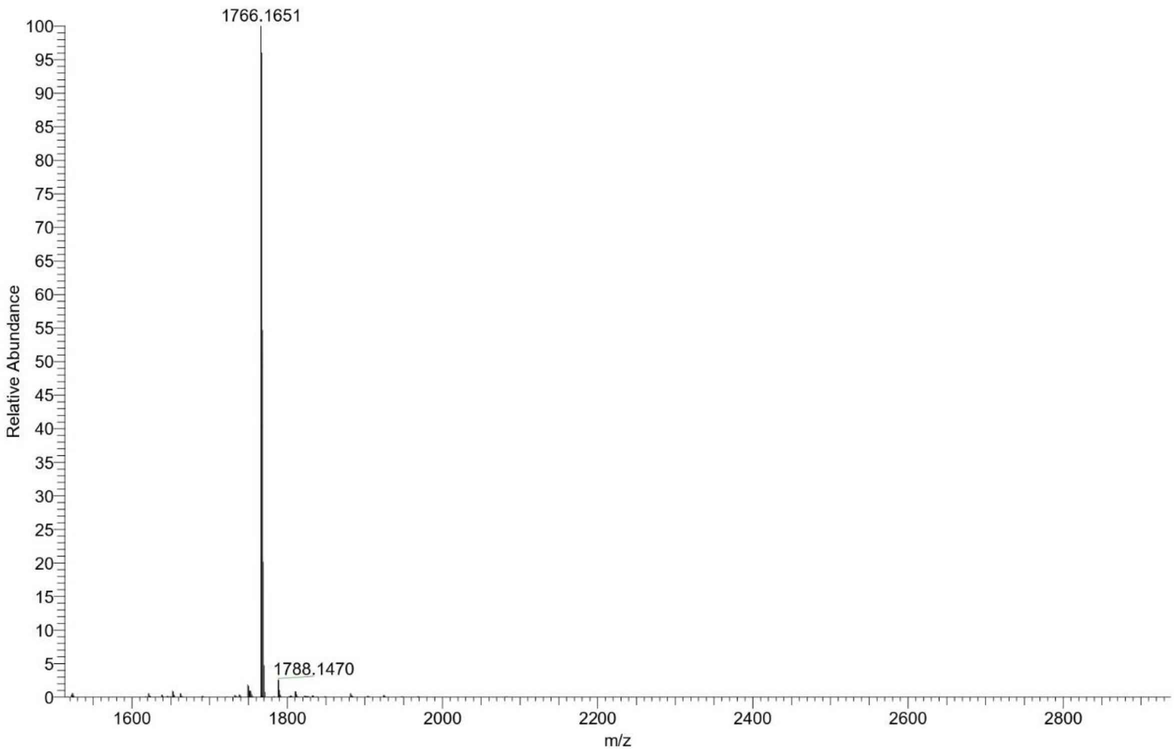
1766.1651
1788.1470
Relative Abundance
m/z

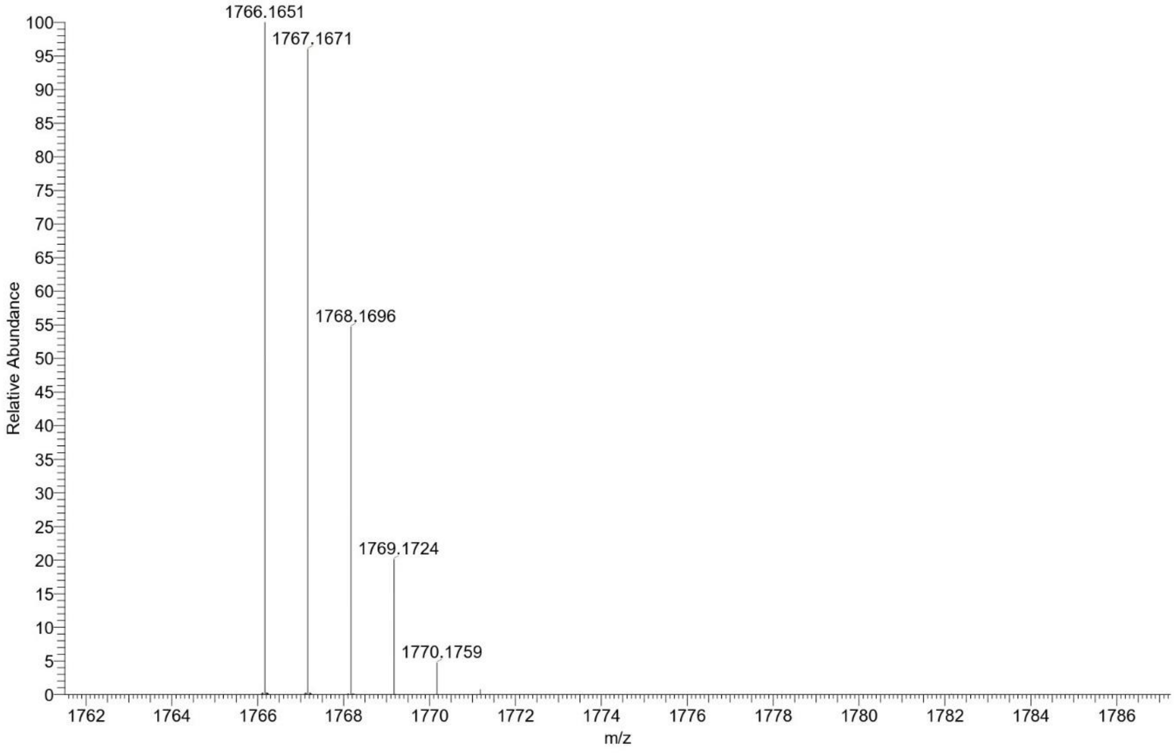
1766.1651
1767.1671
1768.1696
1769.1724
1770.1759
Relative Abundance
m/z

B8 (KWRQLGKKIIKVAK-NH_2) was obtained after manual synthesis from Rink Amide AM resin LL (300 mg, 0.29 mmol/g), the peptide was obtained as a white foamy solid after preparative RP-HPLC purification (41 mg, 23.2%). Analytical RP-HPLC: t_R = 1.24 min (100% A to 100% D in 3.5 min, λ= 214 nm). MS (ESI+): $C_{80}H_{143}N_{25}O_{15}$ calc./obs. 1694.12/1694.12 $[M]^+$

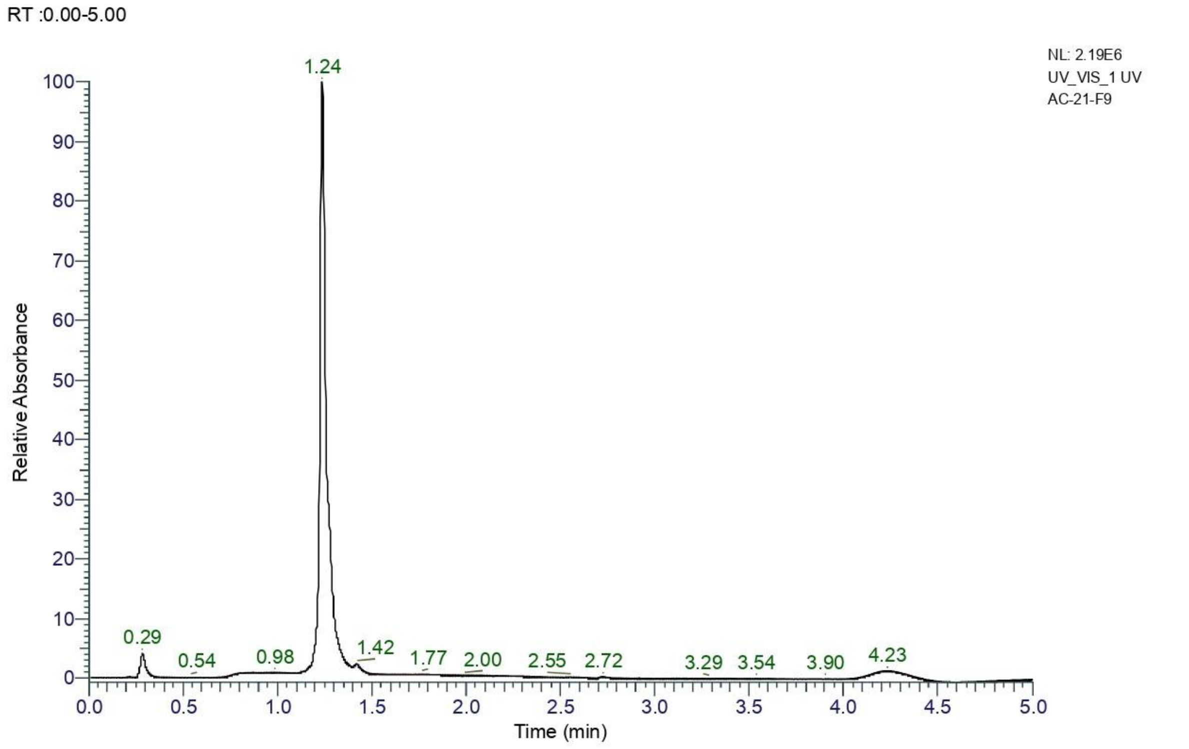

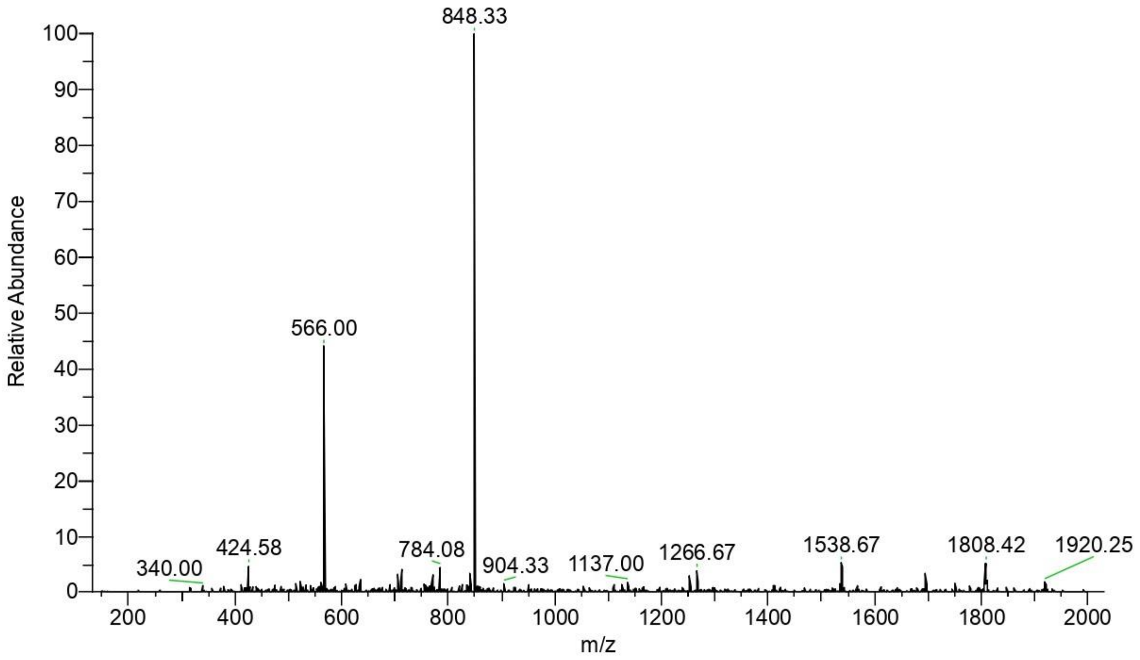

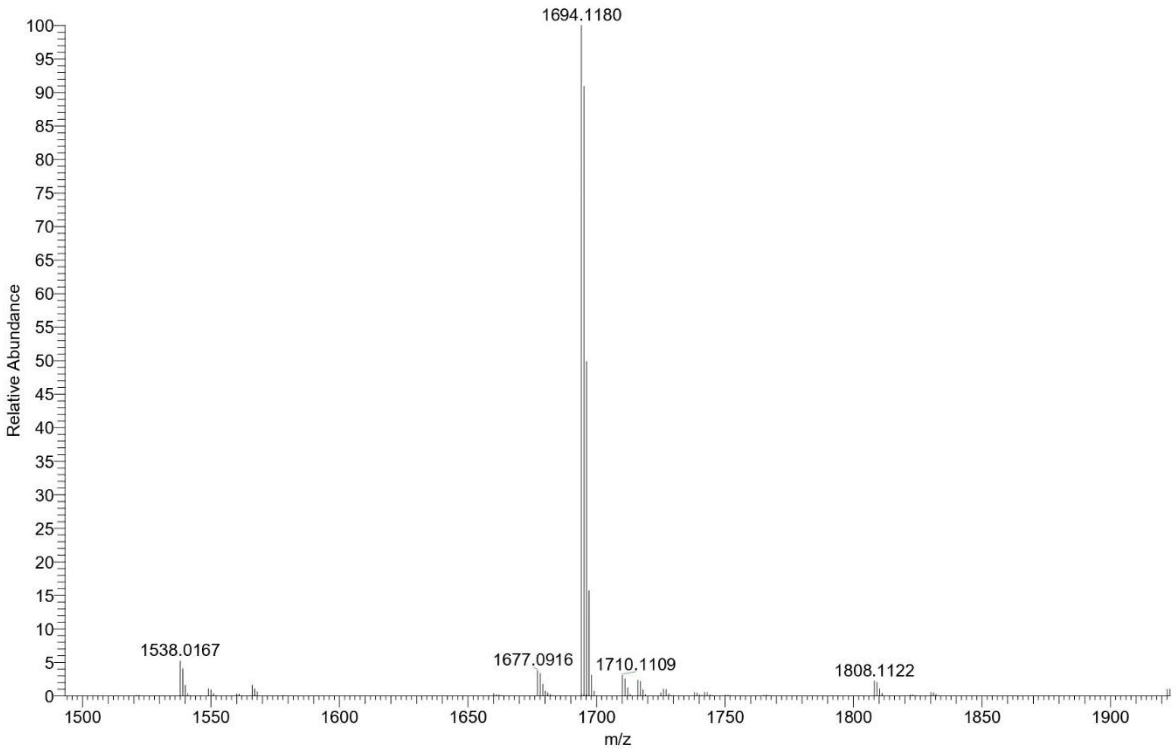
1694.1180
1538.0167
1677.0916
1710.1109
1808.1122
Relative Abundance
m/z

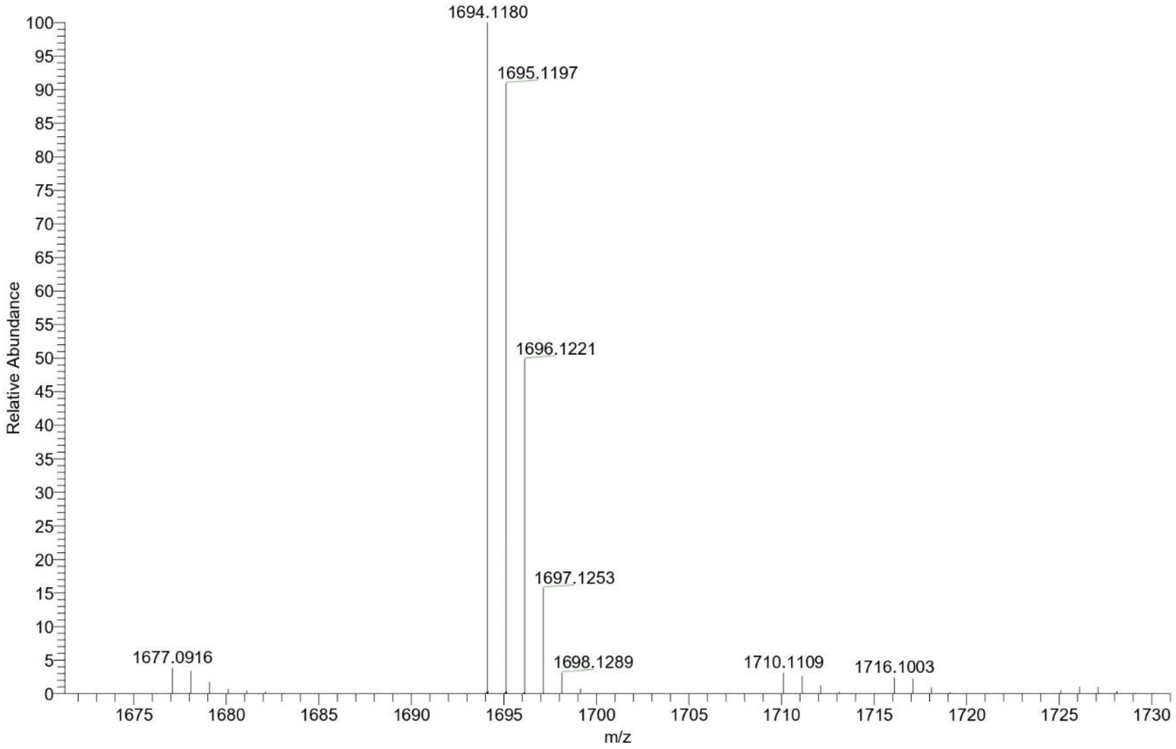
1694.1180
1695.1197
1696.1221
1697.1253
1698.1289
1677.0916
1710.1109
1716.1003
Relative Abundance
m/z

B9 (NWKKIRKLGKVVKKI-NH_2) was obtained after manual synthesis from Rink Amide AM resin LL (300 mg, 0.29 mmol/g), the peptide was obtained as a white foamy solid after preparative RP-HPLC purification (28 mg, 12.2%). Analytical RP-HPLC: t_R = 1.26 min (100% A to 100% D in 3.5 min, λ= 214 nm). MS (ESI+): $C_{87}H_{157}N_{27}O_{16}$ calc./obs. 1836.23/1836.23 $[M]^+$

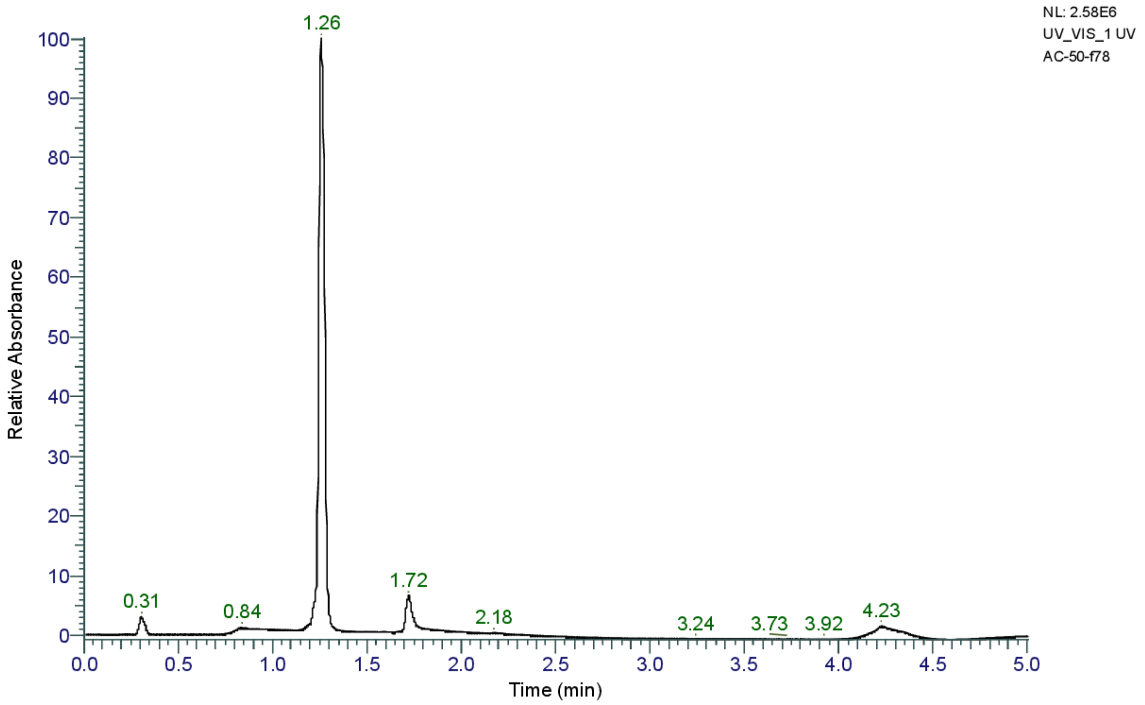

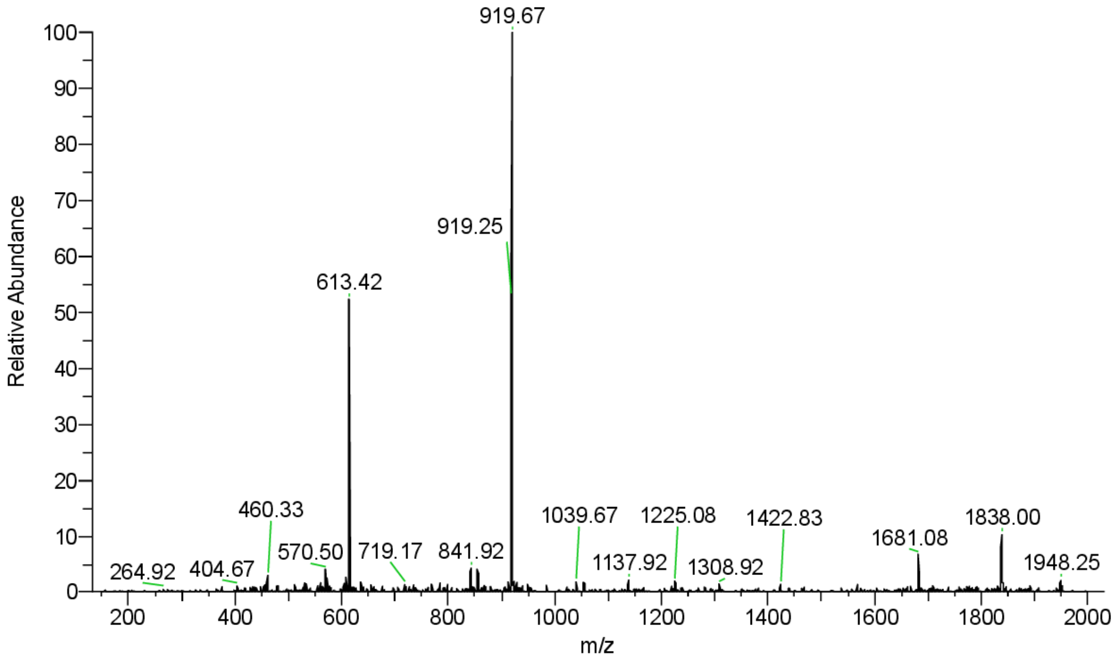

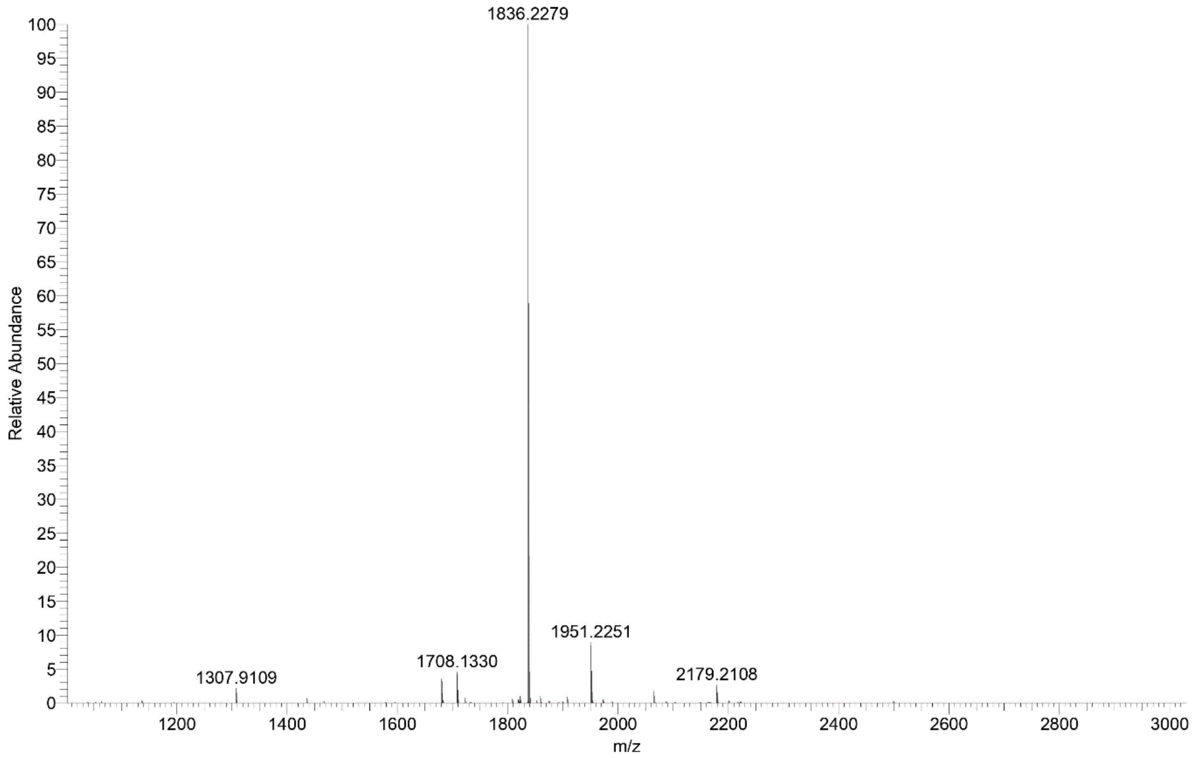
1836.2279
1307.9109
1708.1330
1951.2251
2179.2108
Relative Abundance
m/z

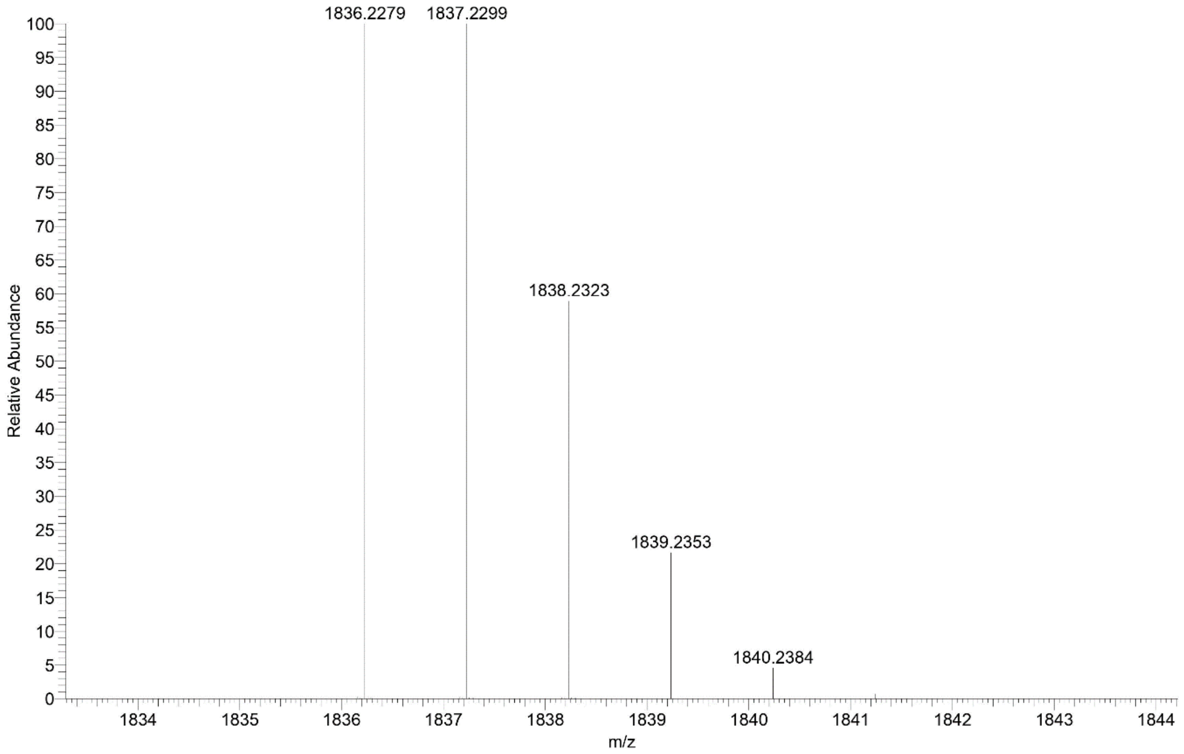
1836.2279
1837.2299
1838.2323
1839.2353
1840.2384
Relative Abundance
m/z

B10 (VVNNWKKKKIIKVIK-NH_2) was obtained after manual synthesis from Rink Amide AM resin LL (300 mg, 0.29 mmol/g), the peptide was obtained as a white foamy solid after preparative RP-HPLC purification (33 mg, 16.6%). Analytical RP-HPLC: t_R = 1.33 min (100% A to 100% D in 3.5 min, λ= 214 nm). MS (ESI+): $C_{82}H_{145}N_{23}O_{16}$ calc./obs. 1708.12/1708.12 $[M]^+$

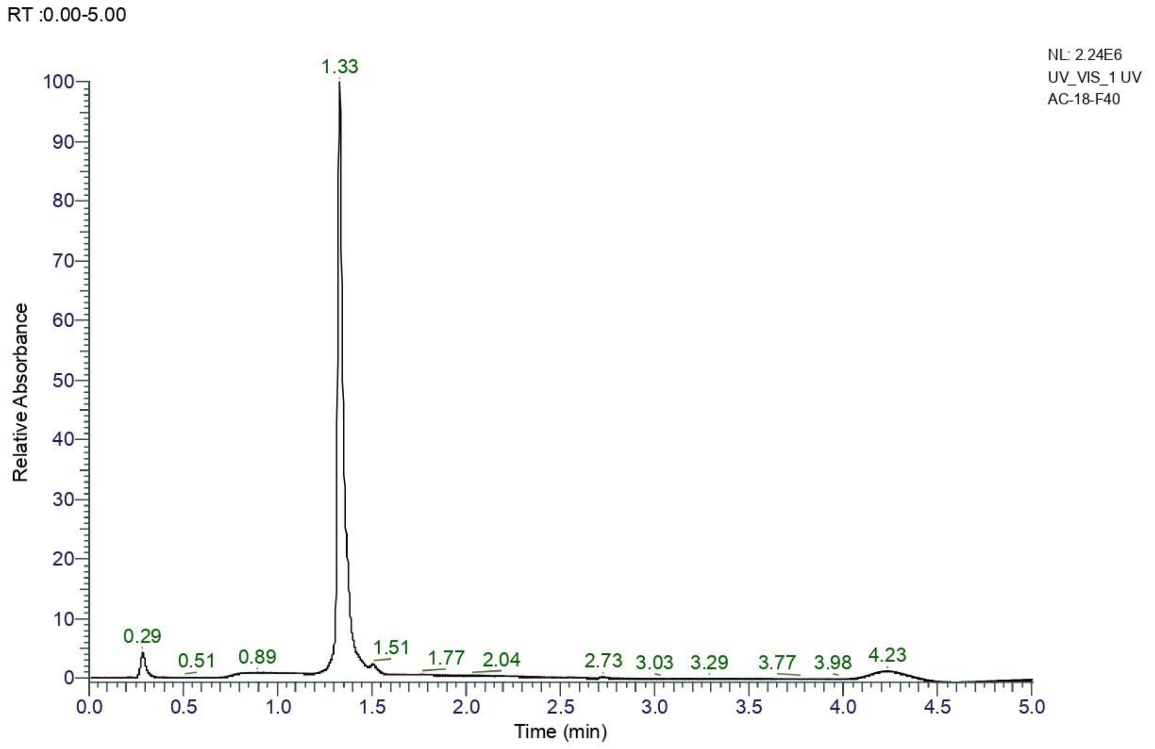

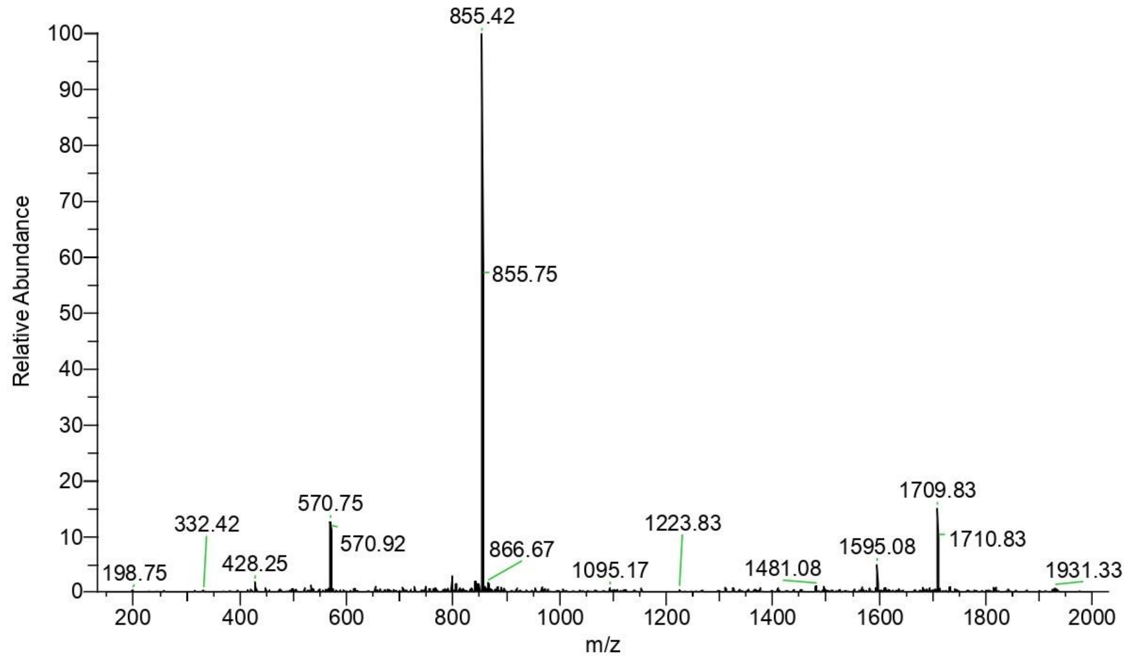

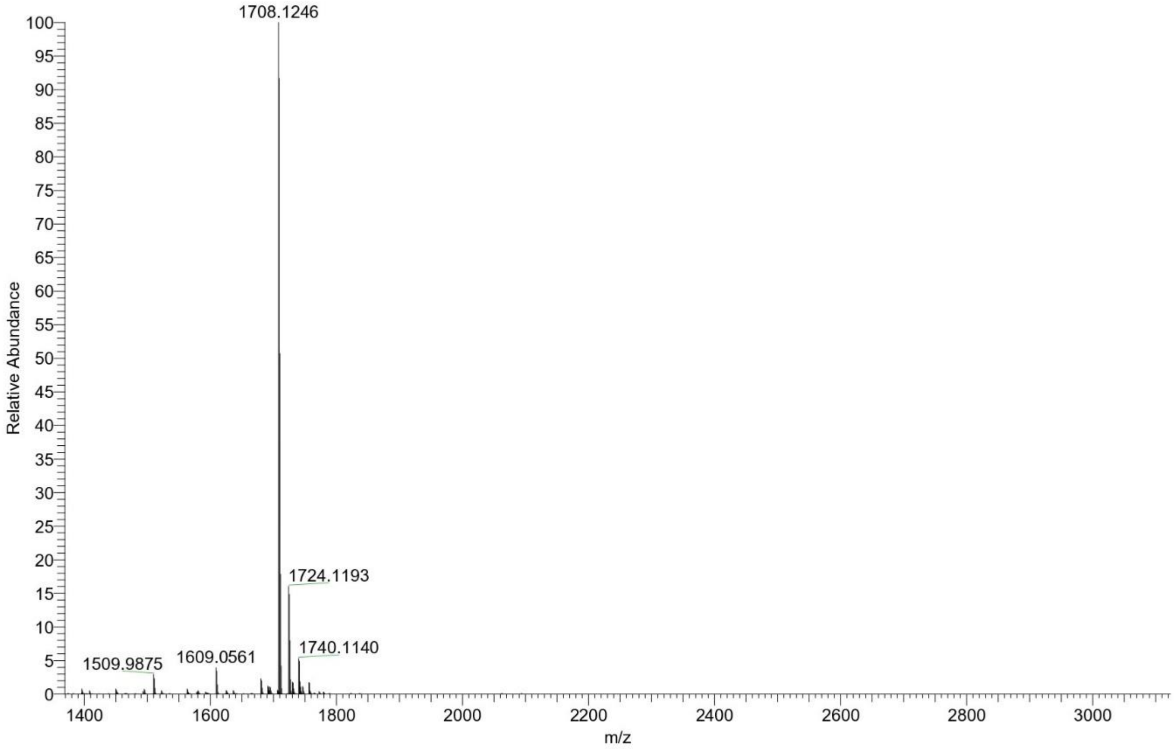
1708.1246
1724.1193
1740.1140
1509.9875
1609.0561
Relative Abundance
m/z

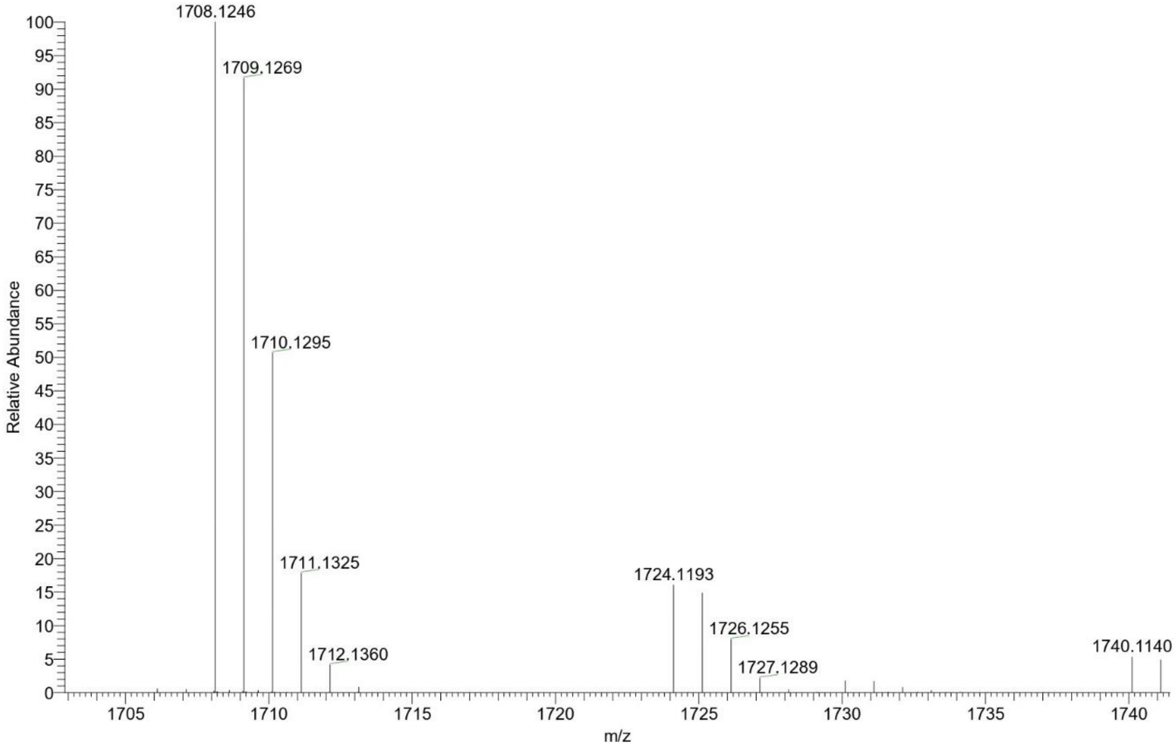
1708.1246
1709.1269
1710.1295
1711.1325
1712.1360
1724.1193
1726.1255
1727.1289
1740.1140
Relative Abundance
m/z

B11 (DWHKIGKKVIKVIK-NH_2) was obtained after manual synthesis from Rink Amide AM resin LL (300 mg, 0.29 mmol/g), the peptide was obtained as a white foamy solid after preparative RP-HPLC purification (29 mg, 14.7%). Analytical RP-HPLC: t_R = 1.31 min (100% A to 100% D in 3.5 min, λ= 214 nm). MS (ESI+): $C_{81}H_{139}N_{23}O_{16}$ calc./obs. 1690.08/1690.08 $[M]^+$

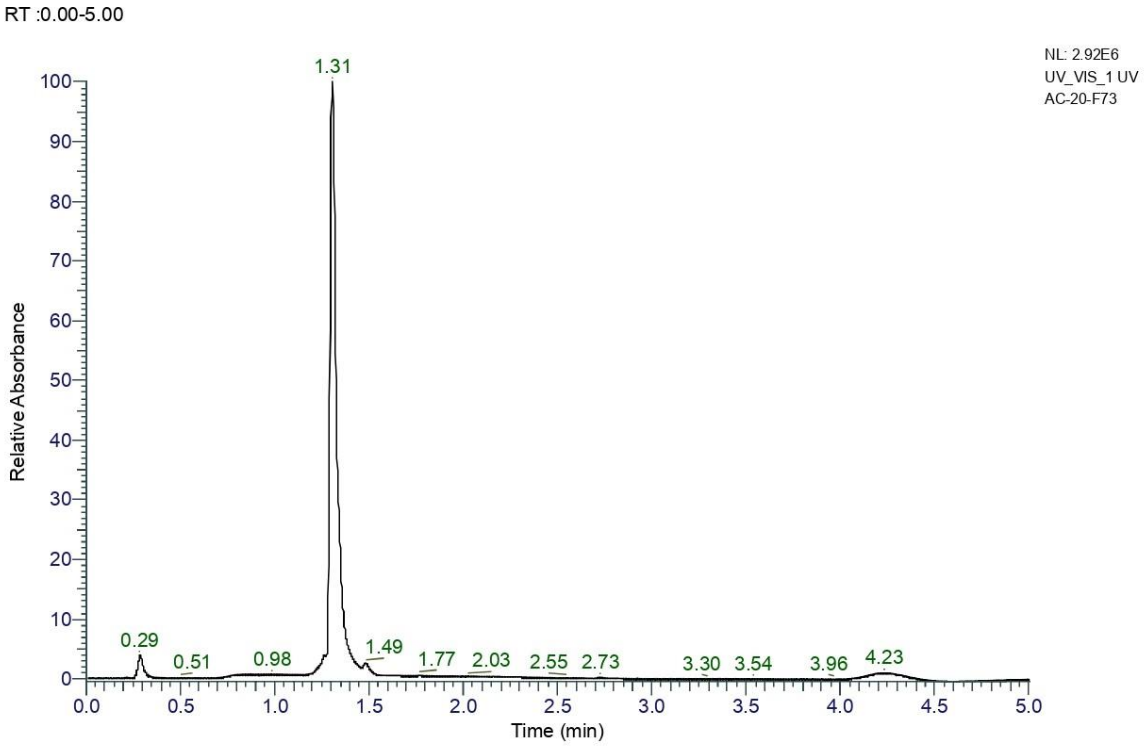

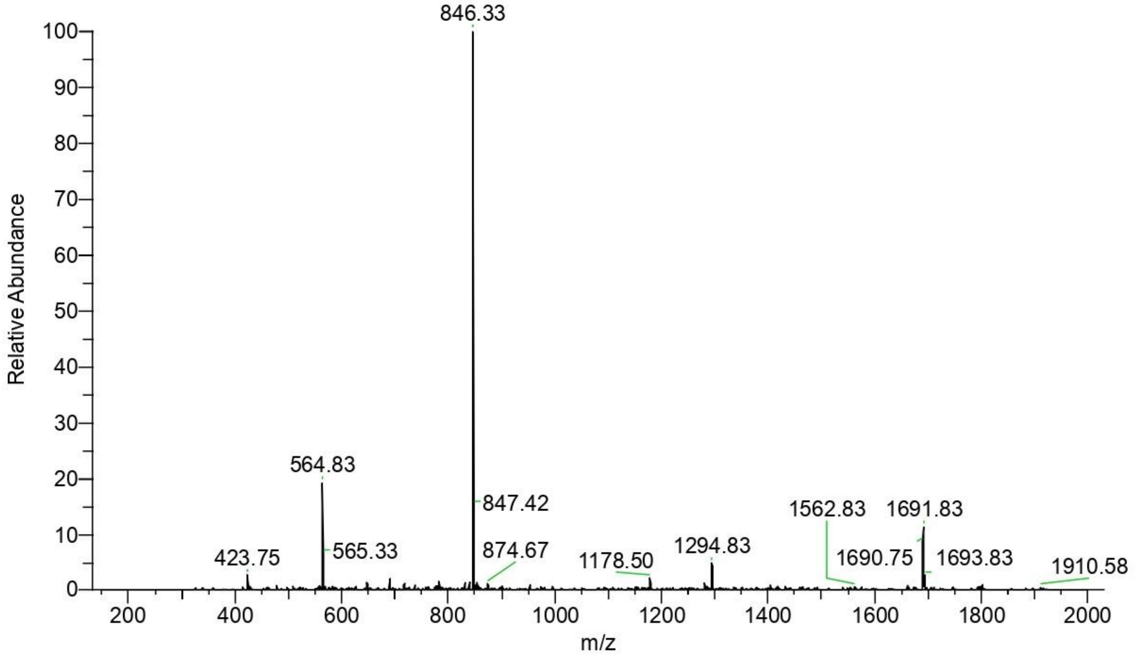

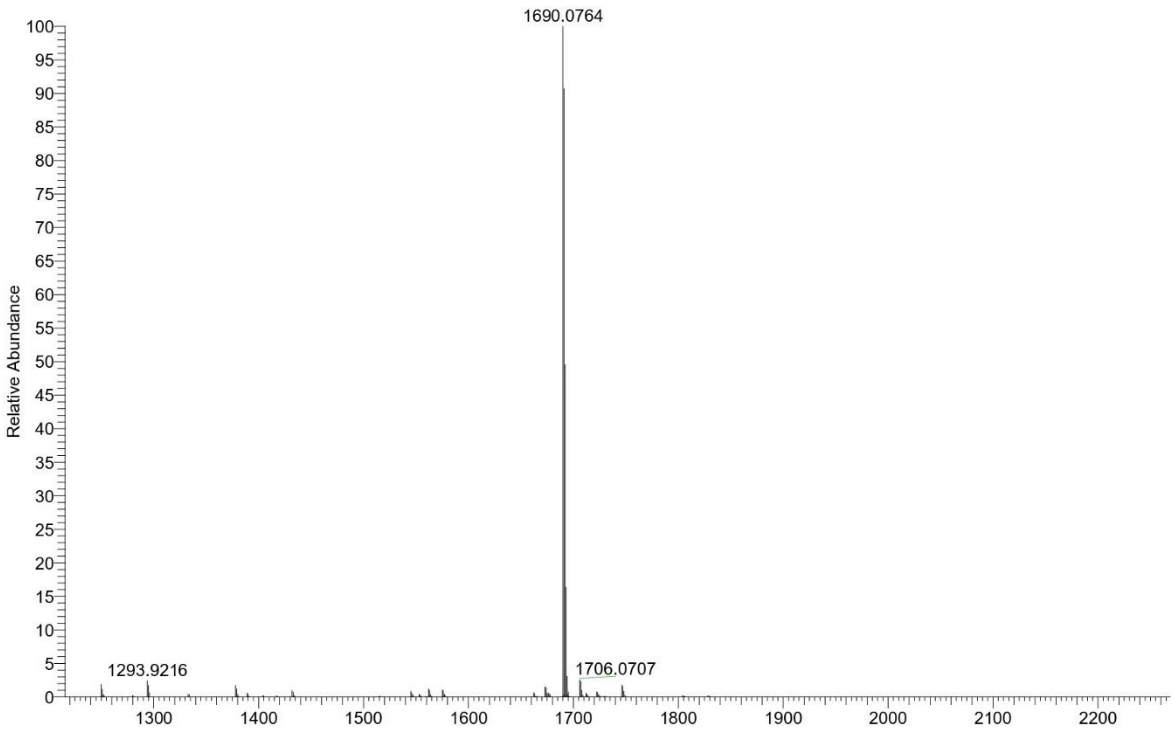
1690.0764
1293.9216
1706.0707
Relative Abundance
m/z
0
5
10
15
20
25
30
35
40
45
50
55
60
65
70
75
80
85
90
95
100
1300
1400
1500
1600
1700
1800
1900
2000
2100
2200

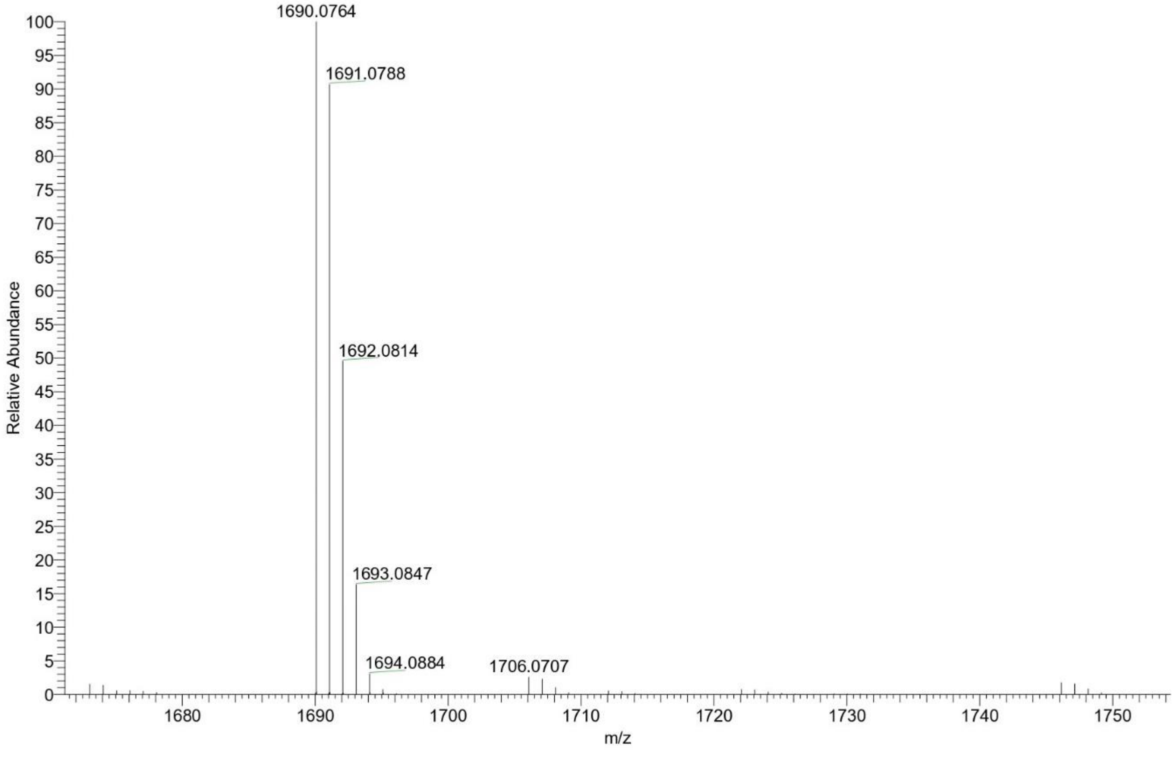
1690.0764
1691.0788
1692.0814
1693.0847
1694.0884
1706.0707
Relative Abundance
m/z
0
5
10
15
20
25
30
35
40
45
50
55
60
65
70
75
80
85
90
95
100
1680
1690
1700
1710
1720
1730
1740
1750

B12 (KWNNILGKLGKLAR-NH_2) was obtained after manual synthesis from Rink Amide AM resin LL (300 mg, 0.29 mmol/g), the peptide was obtained as a white foamy solid after preparative RP-HPLC purification (23 mg, 12.8%). Analytical RP-HPLC: t_R = 1.45 min (100% A to 100% D in 3.5 min, λ= 214 nm). MS (ESI+): $C_{74}H_{128}N_{24}O_{16}$ calc./obs. 1609.00/1609.00 $[M]^+$

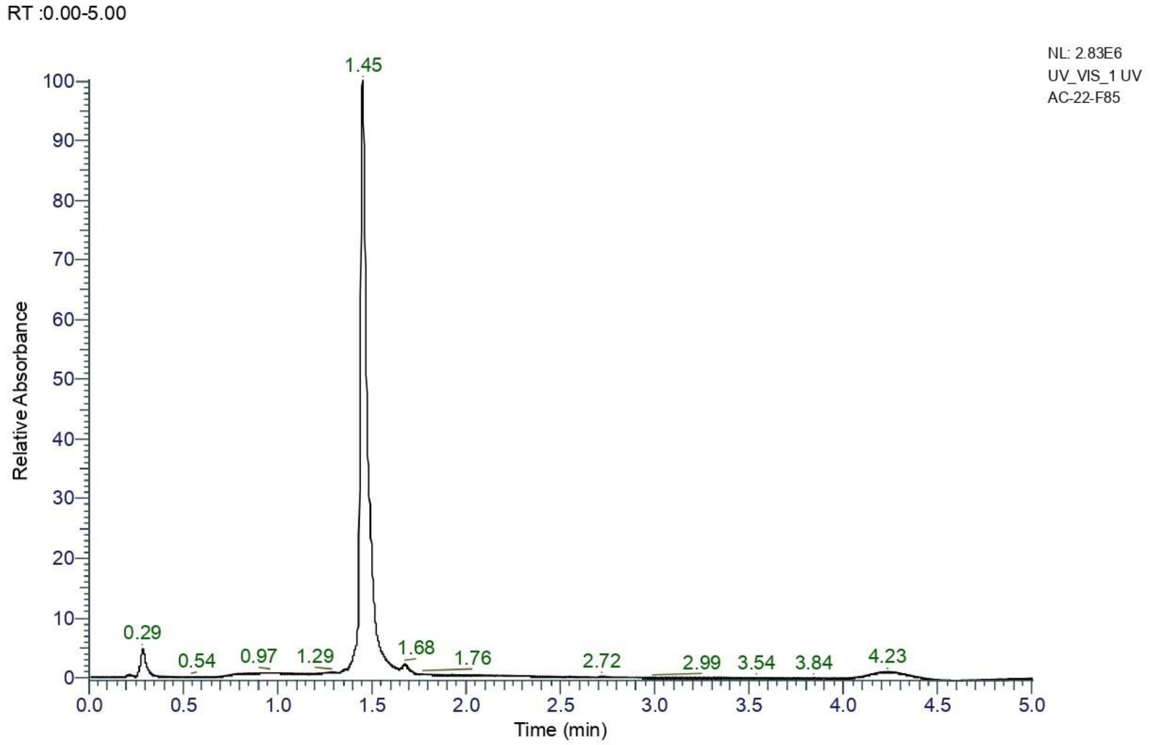

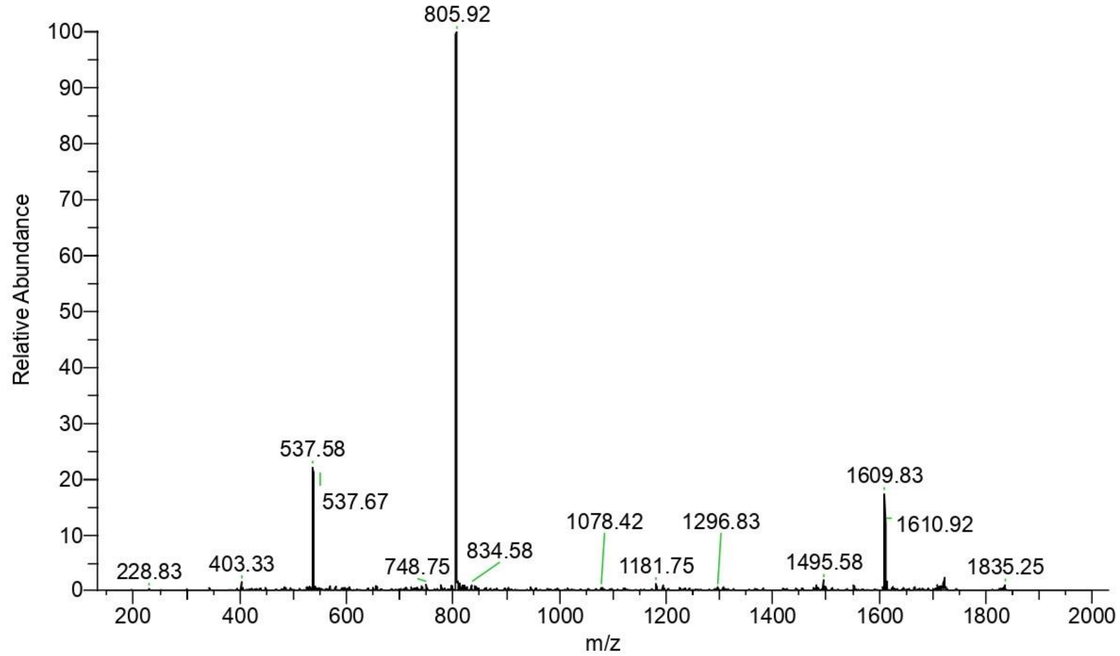

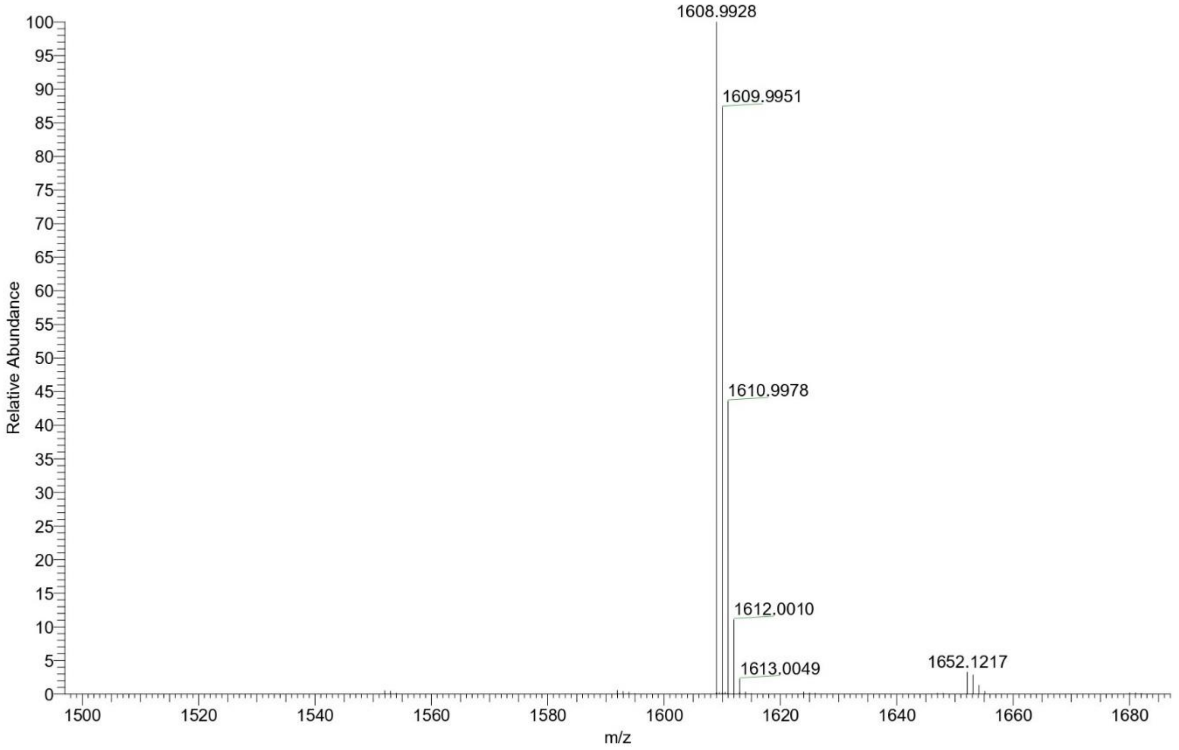
1608.9928
1609.9951
1610.9978
1612.0010
1613.0049
1652.1217
Relative Abundance
m/z

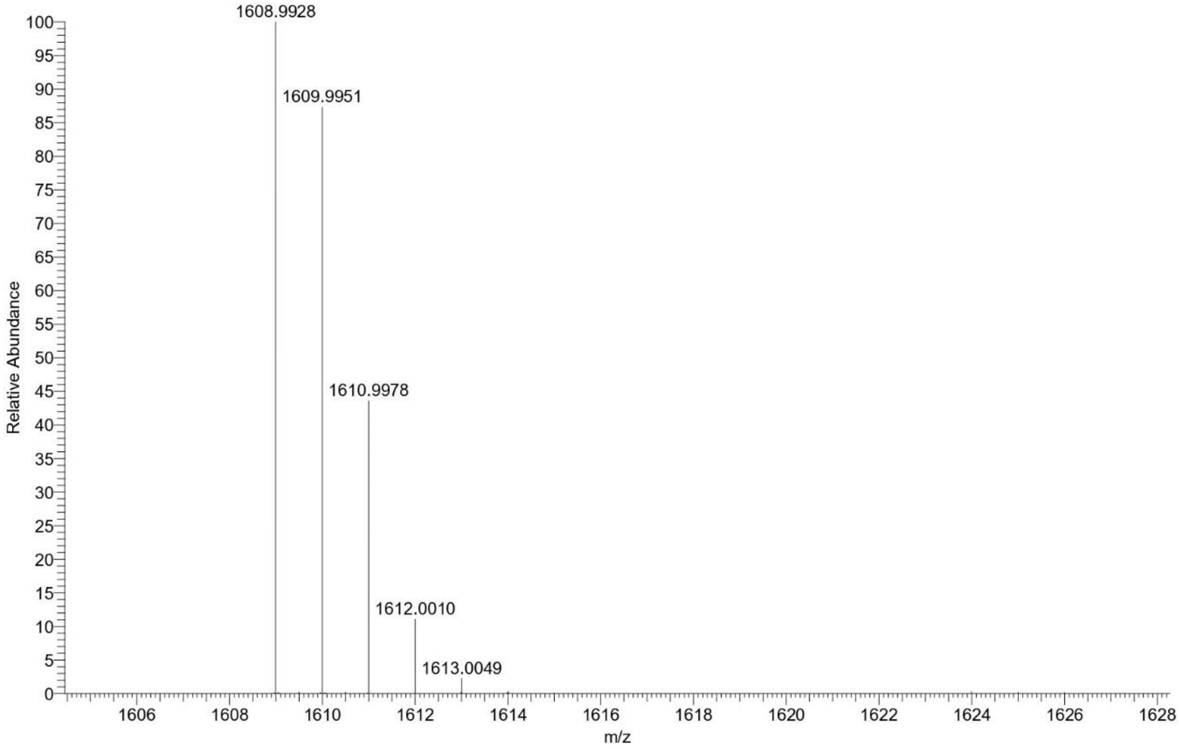
1608.9928
1609.9951
1610.9978
1612.0010
1613.0049
Relative Abundance
m/z

B13 (NVVGRLGKIVKIVK-NH_2) was obtained after manual synthesis from Rink Amide AM resin LL (300 mg, 0.29 mmol/g), the peptide was obtained as a white foamy solid after preparative RP-HPLC purification (60 mg, 34.9%). Analytical RP-HPLC: t_R = 1.40 min (100% A to 100% D in 3.5 min, λ= 214 nm). MS (ESI+): $C_{70}H_{132}N_{22}O_{15}$ calc./obs. 1521.02/1521.02 $[M]^+$

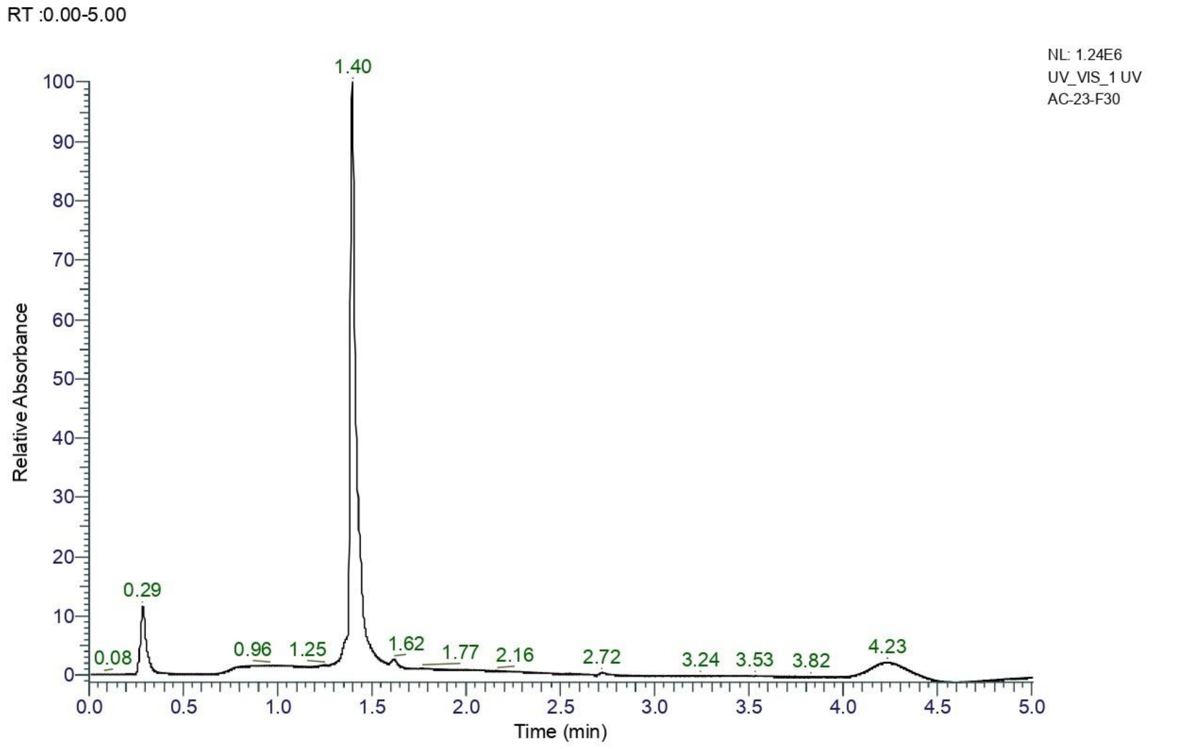

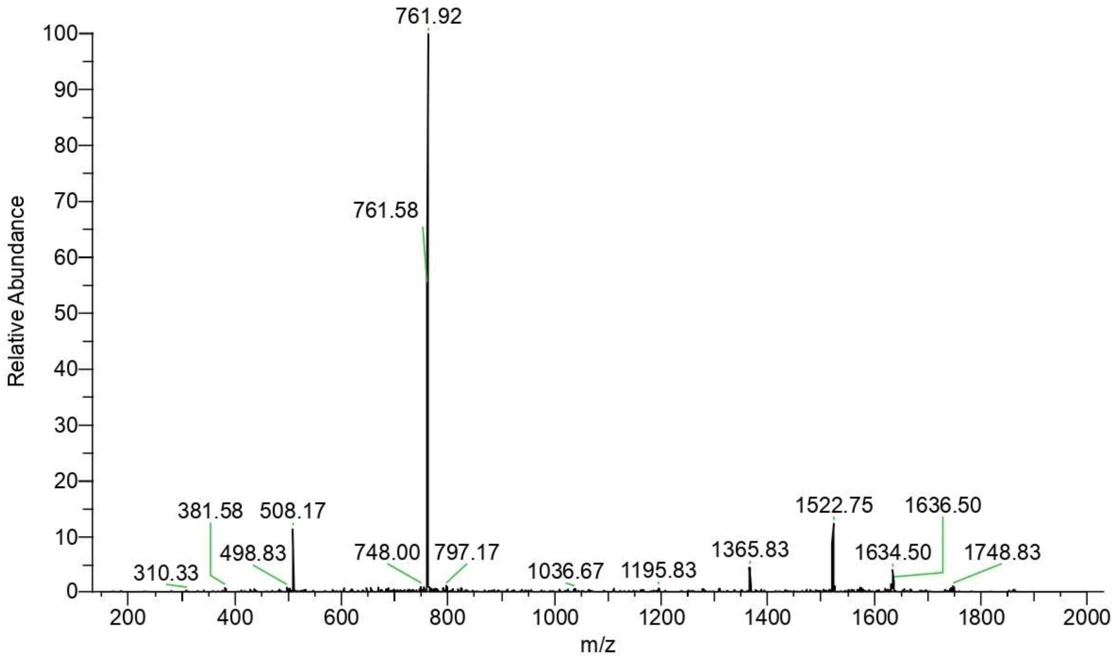

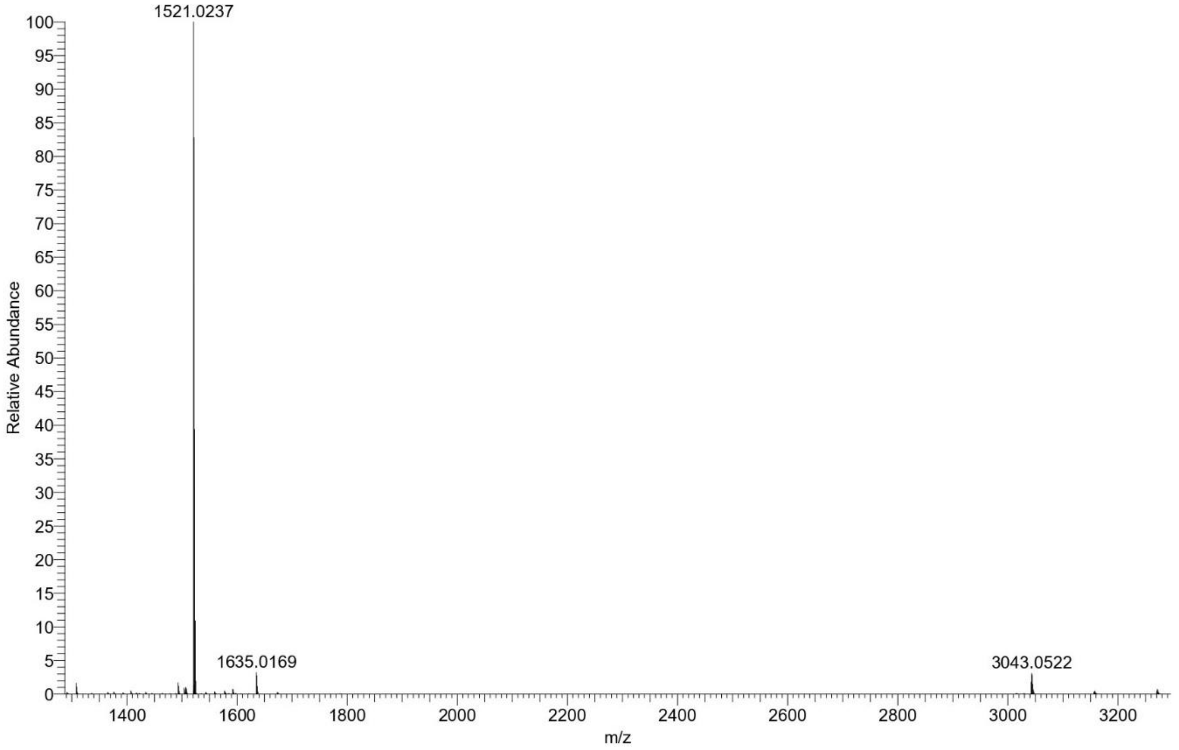
1521.0237
1635.0169
3043.0522
Relative Abundance
m/z

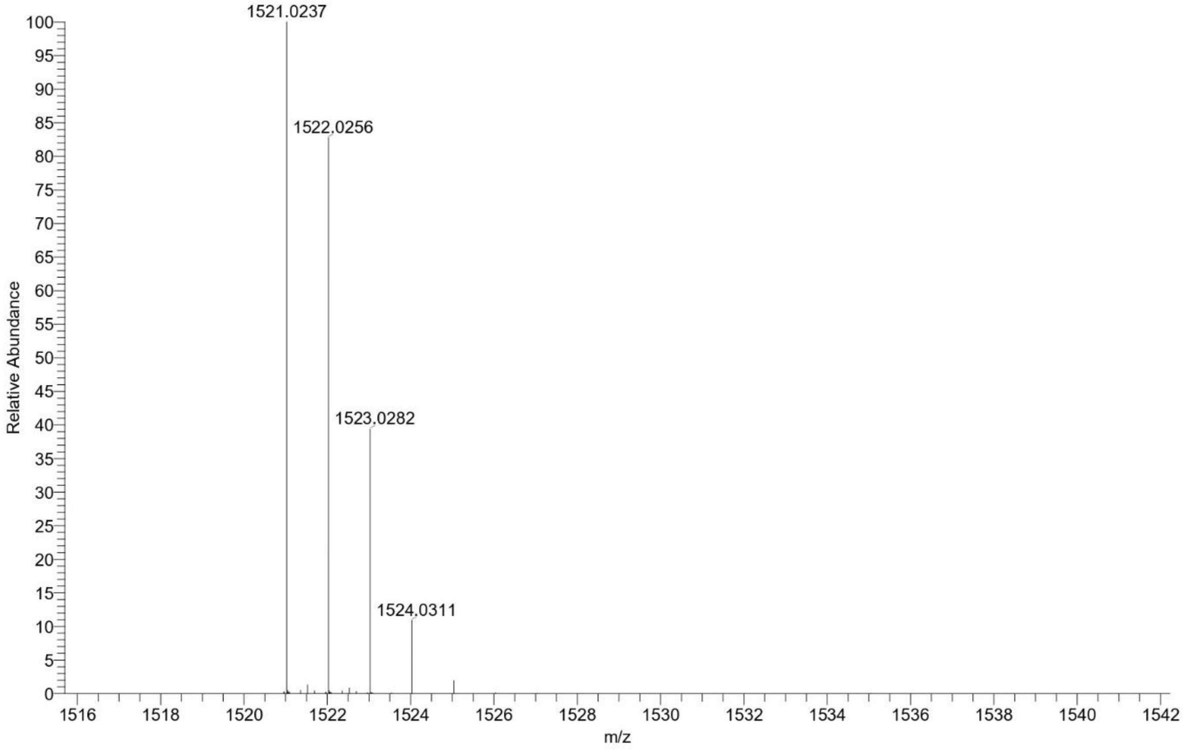
1521.0237
1522.0256
1523.0282
1524.0311
Relative Abundance
m/z

B14 (NPKVFLKKIIKVVK-NH_2) was obtained after manual synthesis from Rink Amide AM resin LL (300 mg, 0.29 mmol/g), the peptide was obtained as a white foamy solid after preparative RP-HPLC purification (36 mg, 18.6%). Analytical RP-HPLC: t_R = 1.42 min (100% A to 100% D in 3.5 min, λ= 214 nm). MS (ESI+): $C_{81}H_{145}N_{21}O_{15}$ calc./obs. 1652.12/1652.12 $[M]^+$

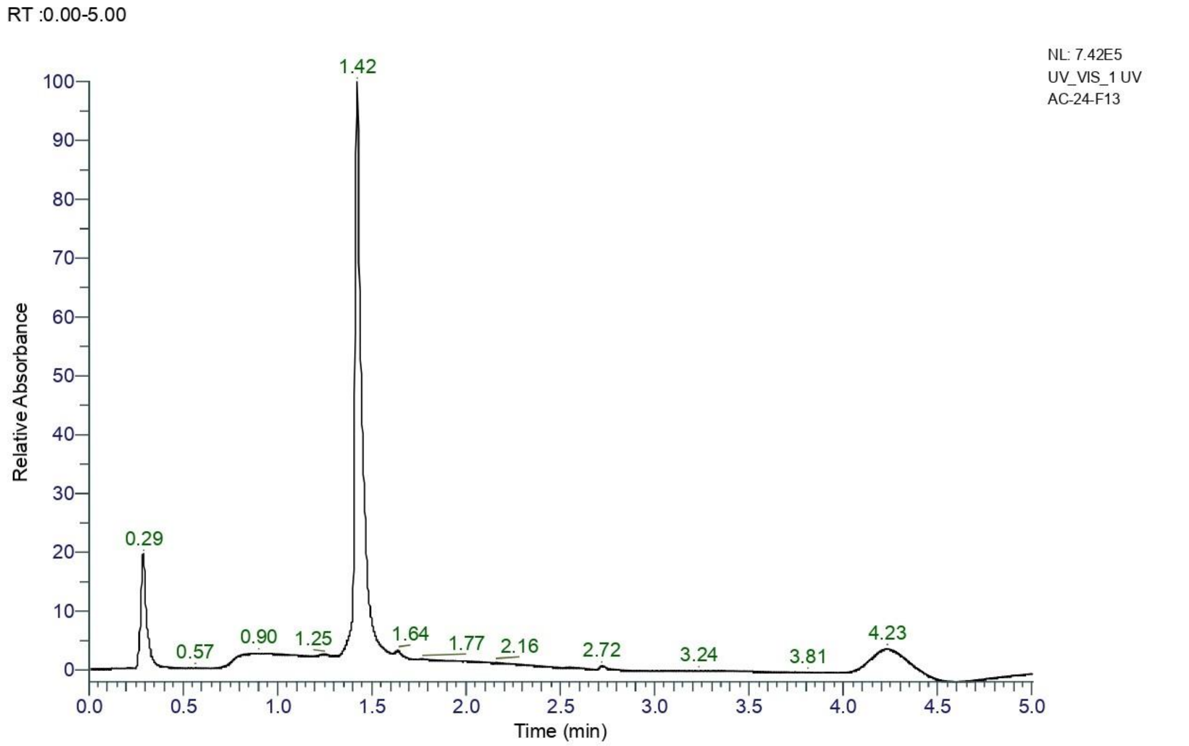

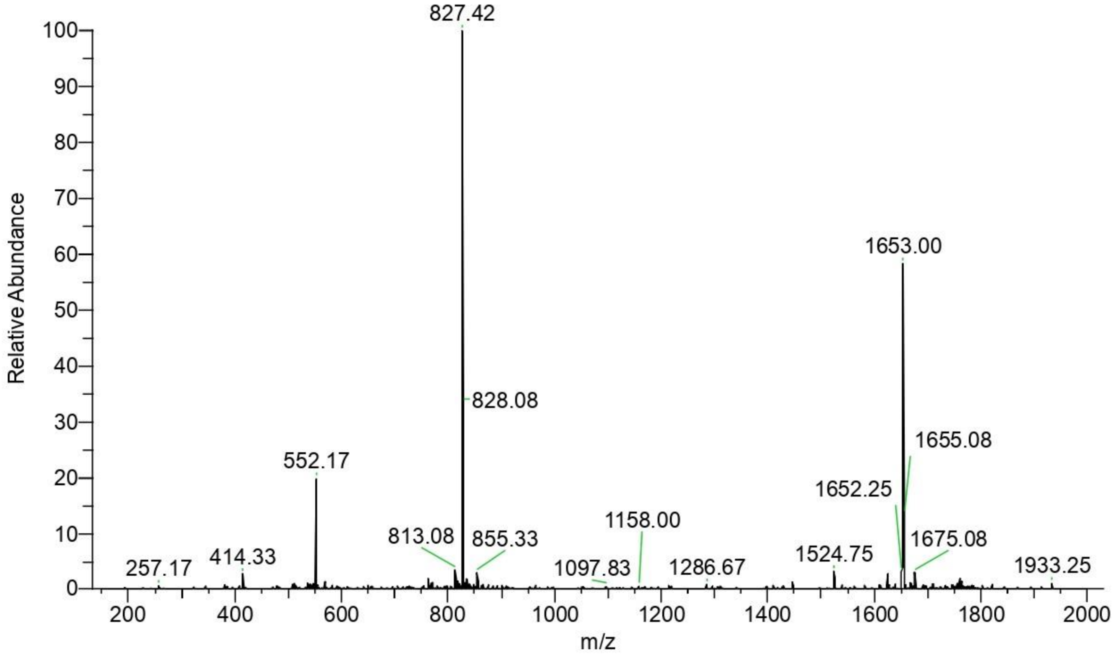

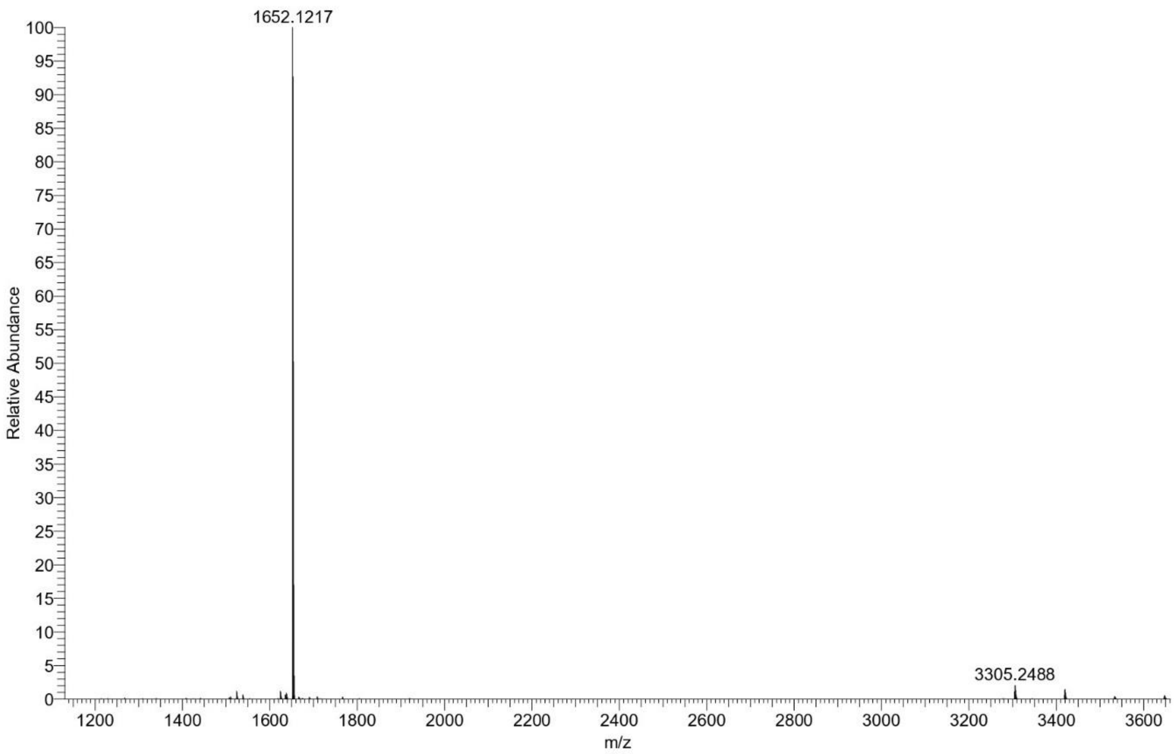
1652.1217
3305.2488
Relative Abundance
m/z

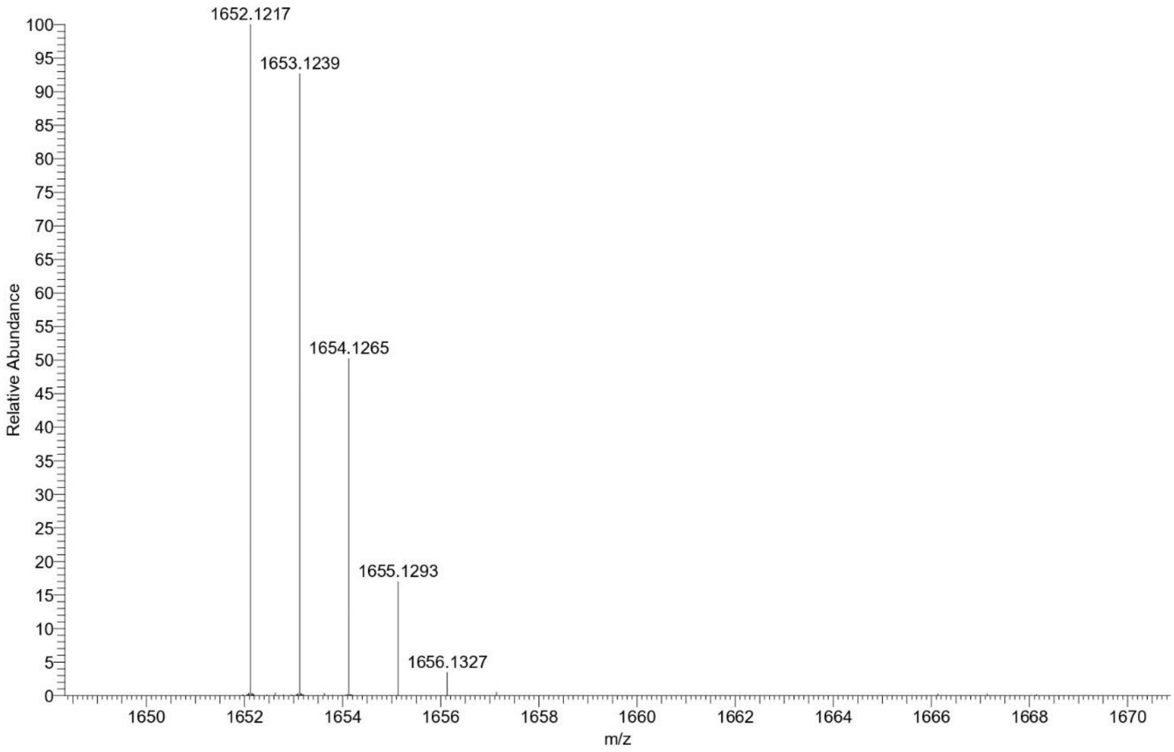
1652.1217
1653.1239
1654.1265
1655.1293
1656.1327
Relative Abundance
m/z

B15 (ADVWKKVIKVIK-NH_2) was obtained after manual synthesis from Rink Amide AM resin LL (300 mg, 0.29 mmol/g), the peptide was obtained as a white foamy solid after preparative RP-HPLC purification (55 mg, 35.8%). Analytical RP-HPLC: t_R = 1.39 min (100% A to 100% D in 3.5 min, λ= 214 nm). MS (ESI+): $C_{69}H_{120}N_{18}O_{14}$ calc./obs. 1424.92/1424.92 $[M]^+$

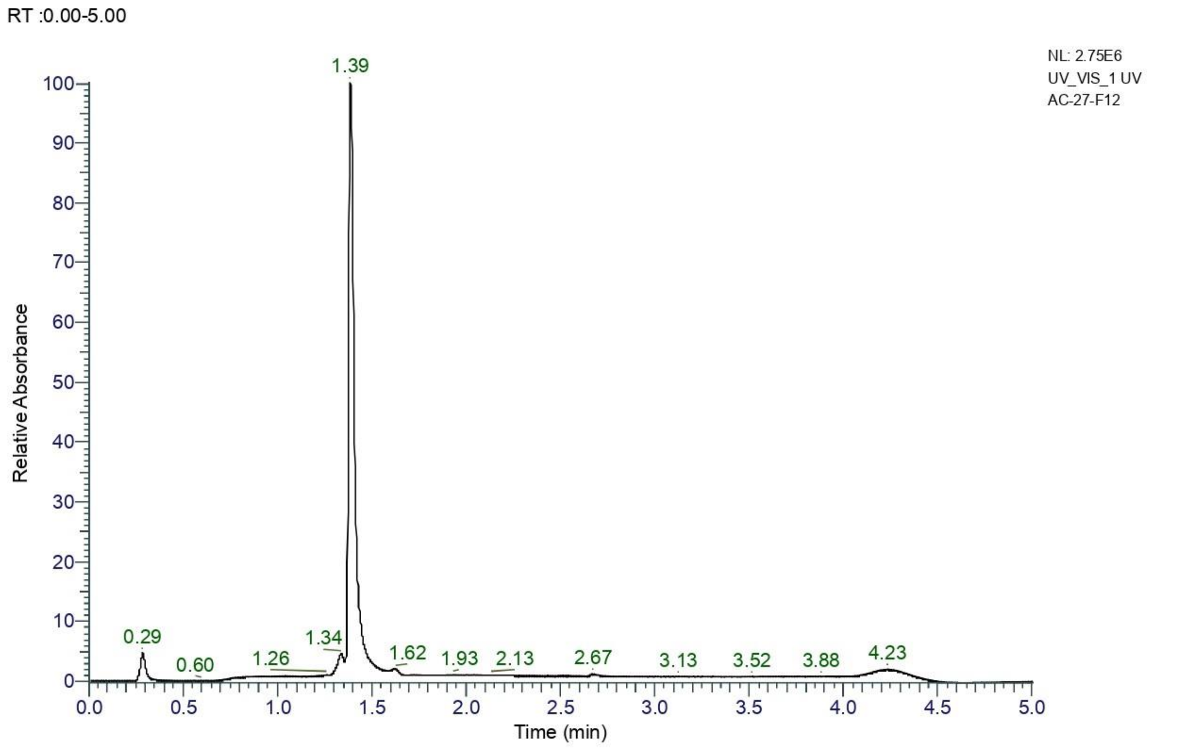

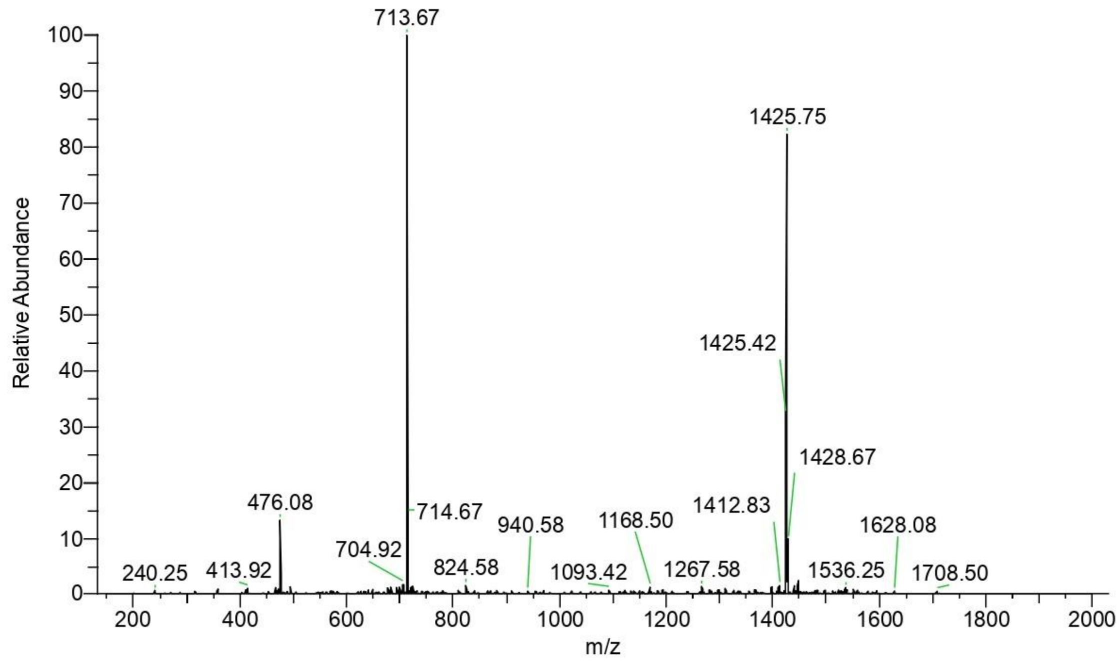

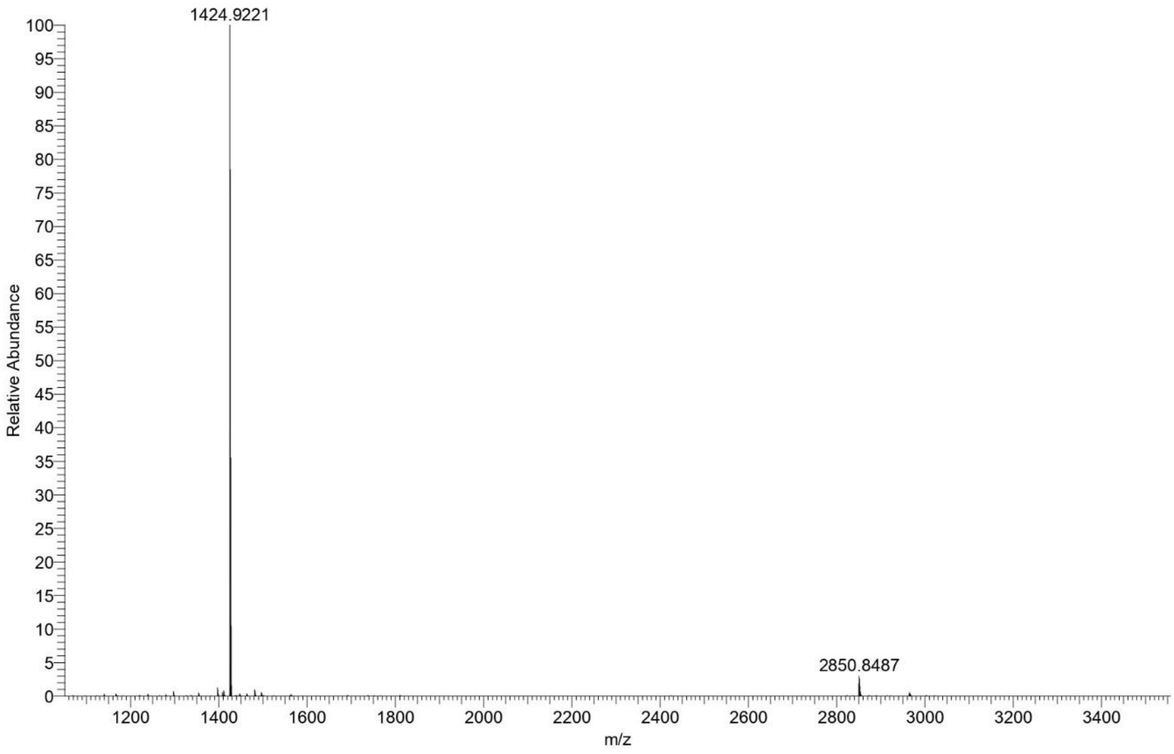
1424.9221
2850.8487
Relative Abundance
m/z

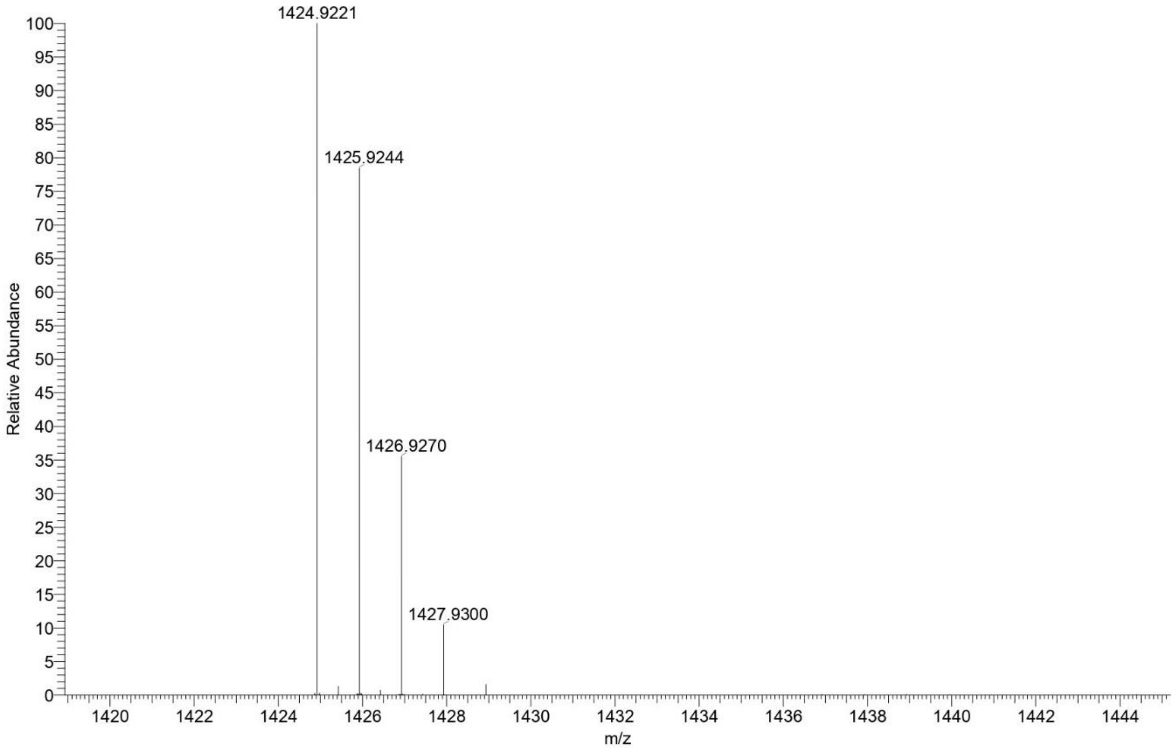
1424.9221
1425.9244
1426.9270
1427.9300
Relative Abundance
m/z

B16 (WRGKIGKIIKAVK-NH$_2$) was obtained after manual synthesis from Rink Amide AM resin LL (300 mg, 0.29 mmol/g), the peptide was obtained as a white foamy solid after preparative RP-HPLC purification (25 mg, 13.9%). Analytical RP-HPLC: t_R = 1.28 min (100% A to 100% D in 3.5 min, λ= 214 nm). MS (ESI+): $C_{71}H_{126}N_{22}O_{13}$ calc./obs. 1494.99/1494.99 $[M]^+$

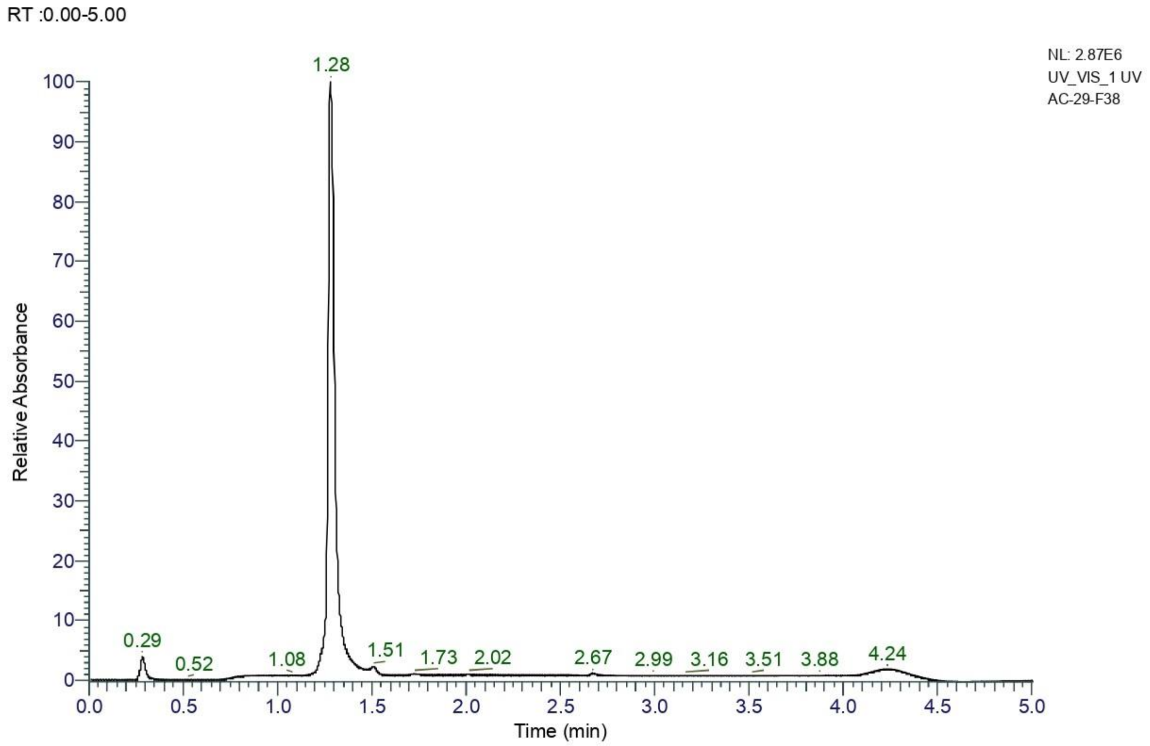

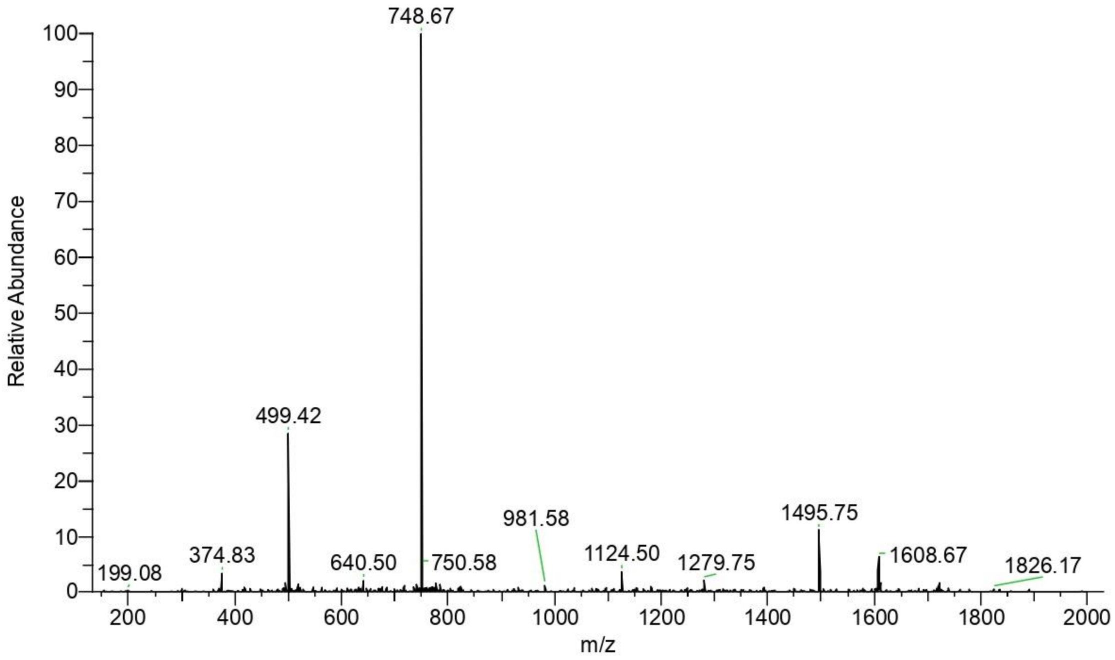

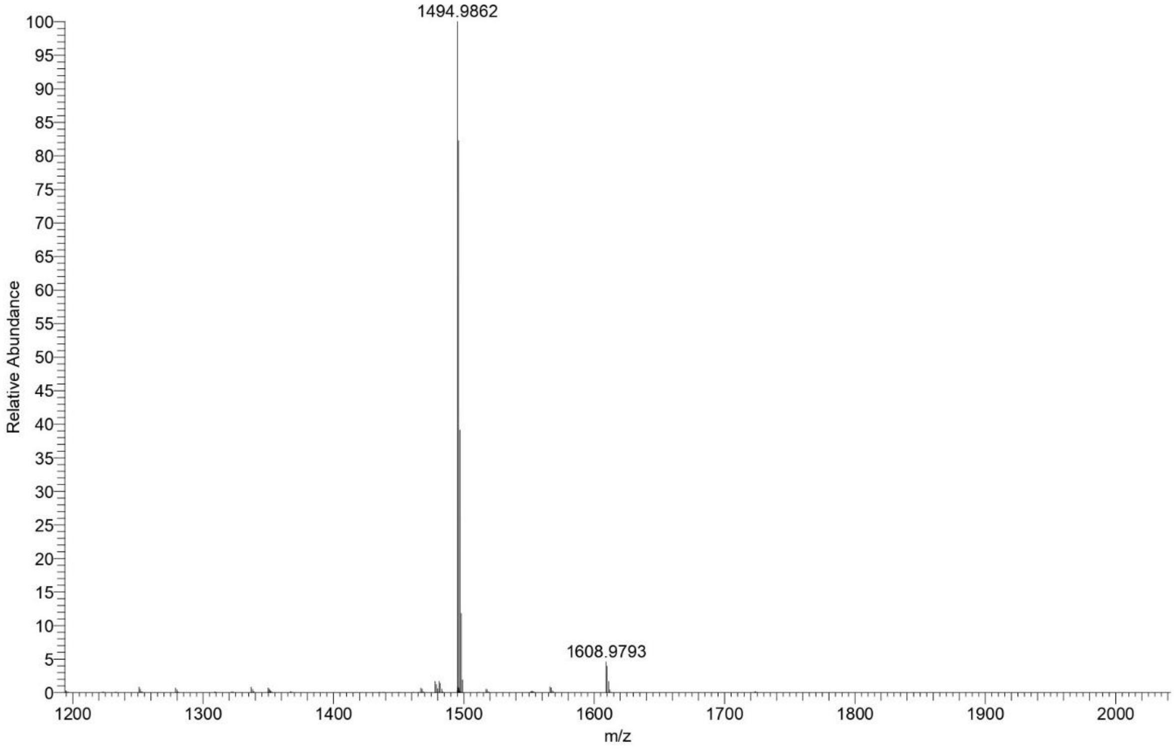
1494.9862
1608.9793
Relative Abundance
m/z

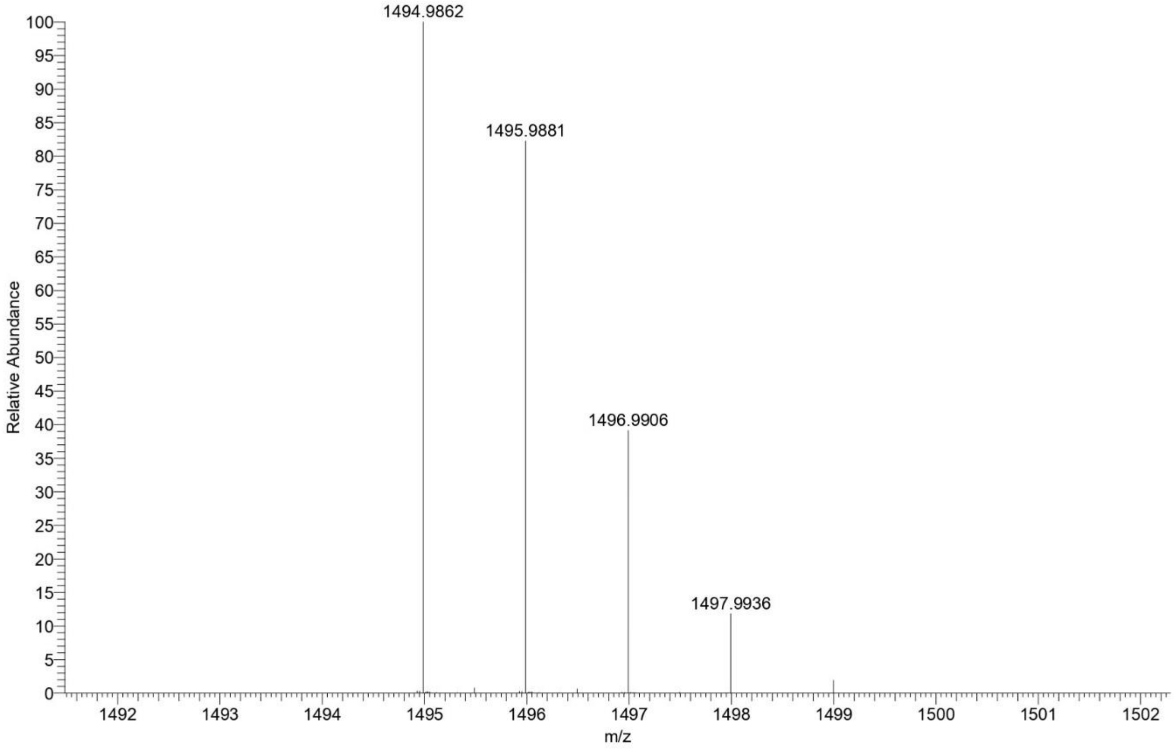
1494.9862
1495.9881
1496.9906
1497.9936
Relative Abundance
m/z

B17 (NWKKILGRLGEKG-NH_2) was obtained after manual synthesis from Rink Amide AM resin LL (300 mg, 0.29 mmol/g), the peptide was obtained as a white foamy solid after preparative RP-HPLC purification (26 mg, 16.3%). Analytical RP-HPLC: t_R = 1.34 min (100% A to 100% D in 3.5 min, λ= 214 nm). MS (ESI+): $C_{68}H_{116}N_{22}O_{16}$ calc./obs. 1496.89/1496.89 $[M]^+$

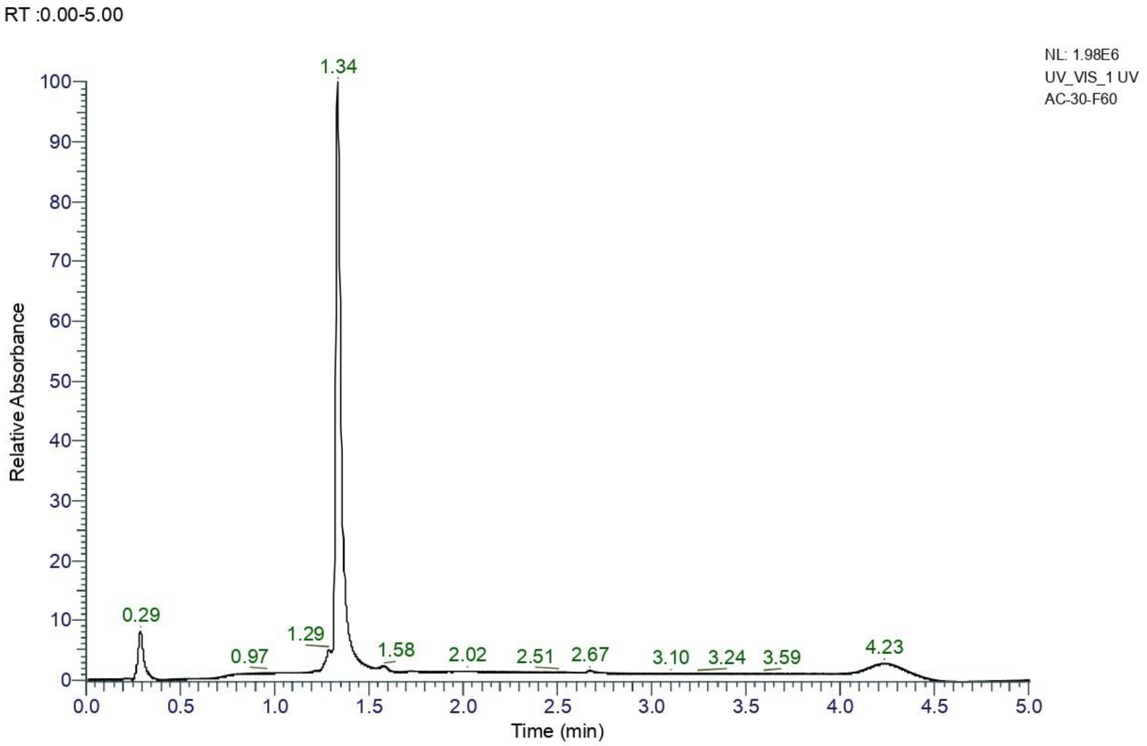

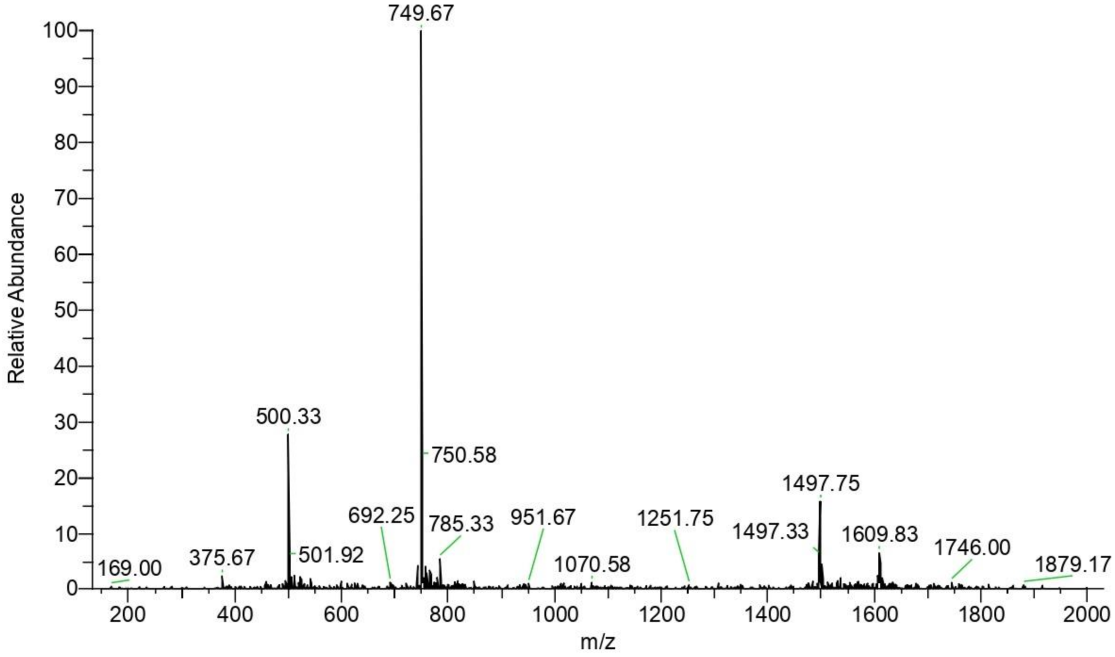

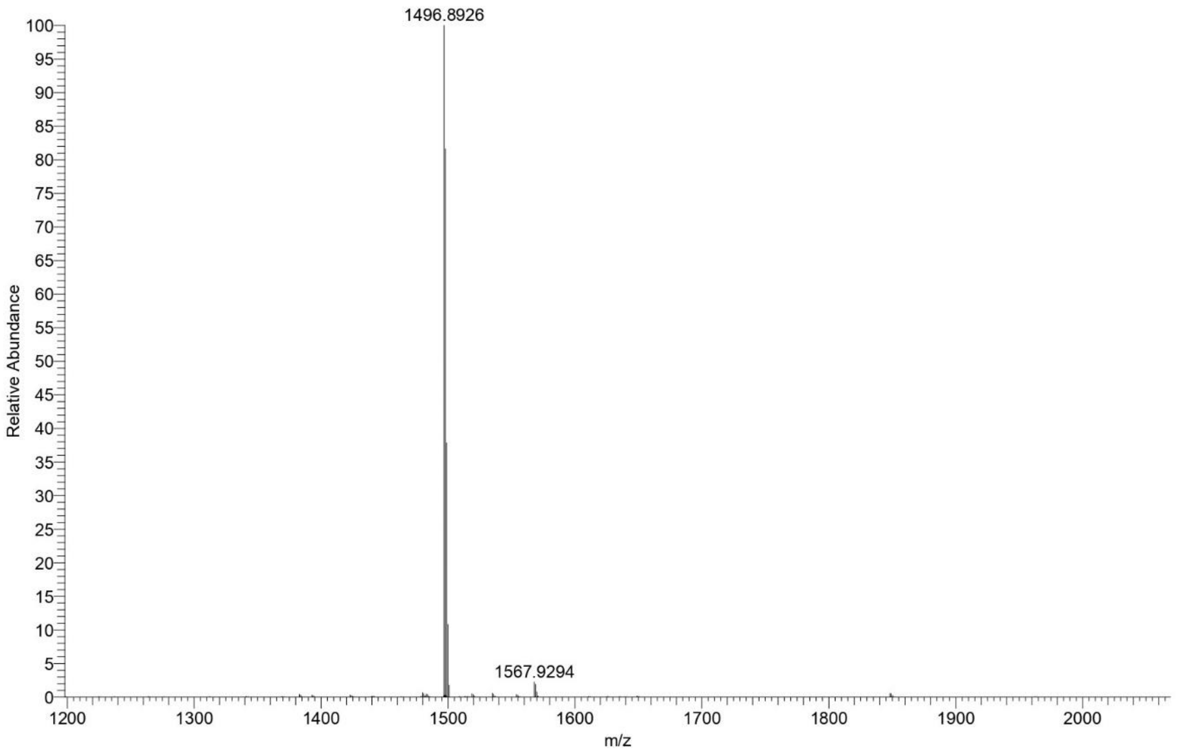
1496.8926
1567.9294
Relative Abundance
m/z

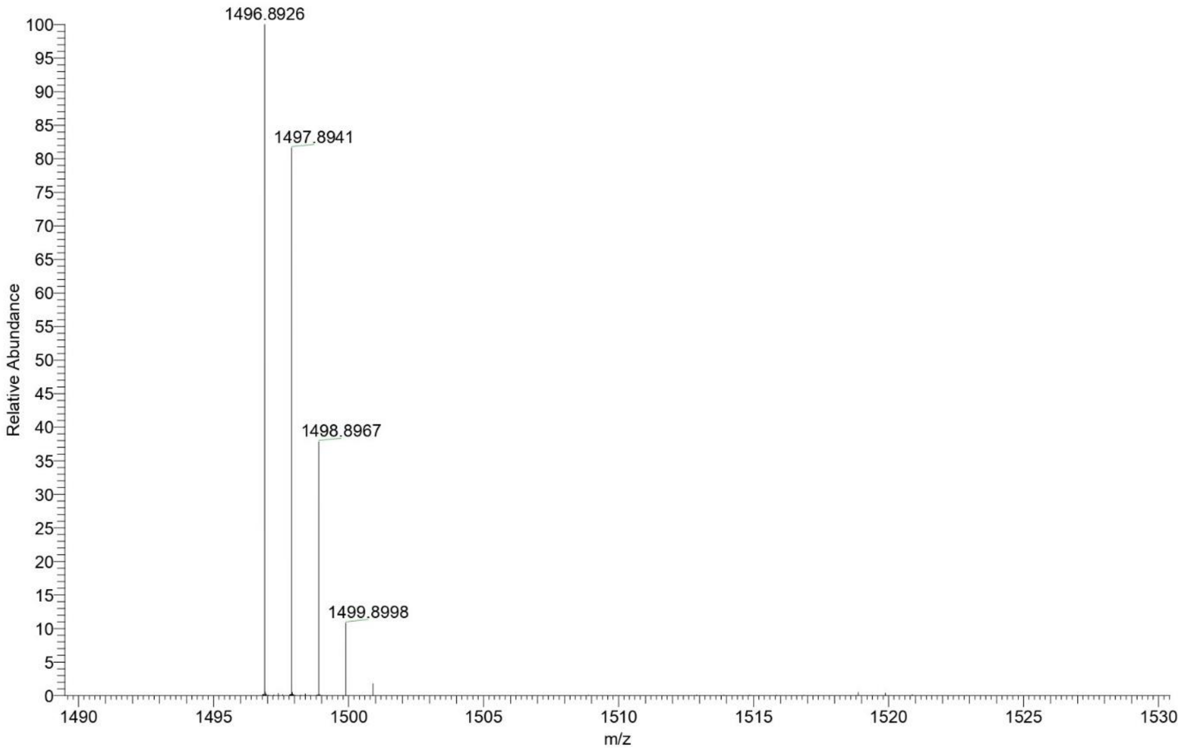
1496.8926
1497.8941
1498.8967
1499.8998
Relative Abundance
m/z

B18 (KNWKKIVHDIKNS-NH_2) was obtained after manual synthesis from Rink Amide AM resin LL (300 mg, 0.29 mmol/g), the peptide was obtained as a white foamy solid after preparative RP-HPLC purification (33 mg, 18.4%). Analytical RP-HPLC: t_R = 1.20 min (100% A to 100% D in 3.5 min, λ= 214 nm). MS (ESI+): $C_{73}H_{121}N_{23}O_{18}$ calc./obs. 1607.93/1607.93[M]$^+$

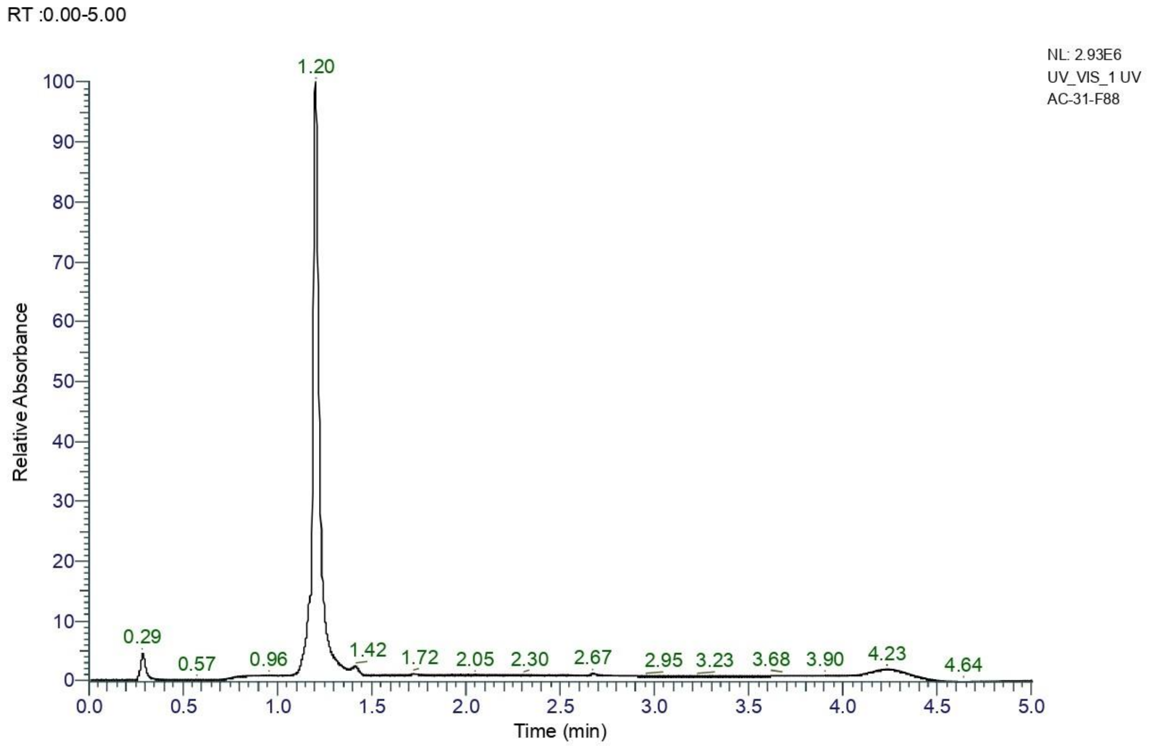

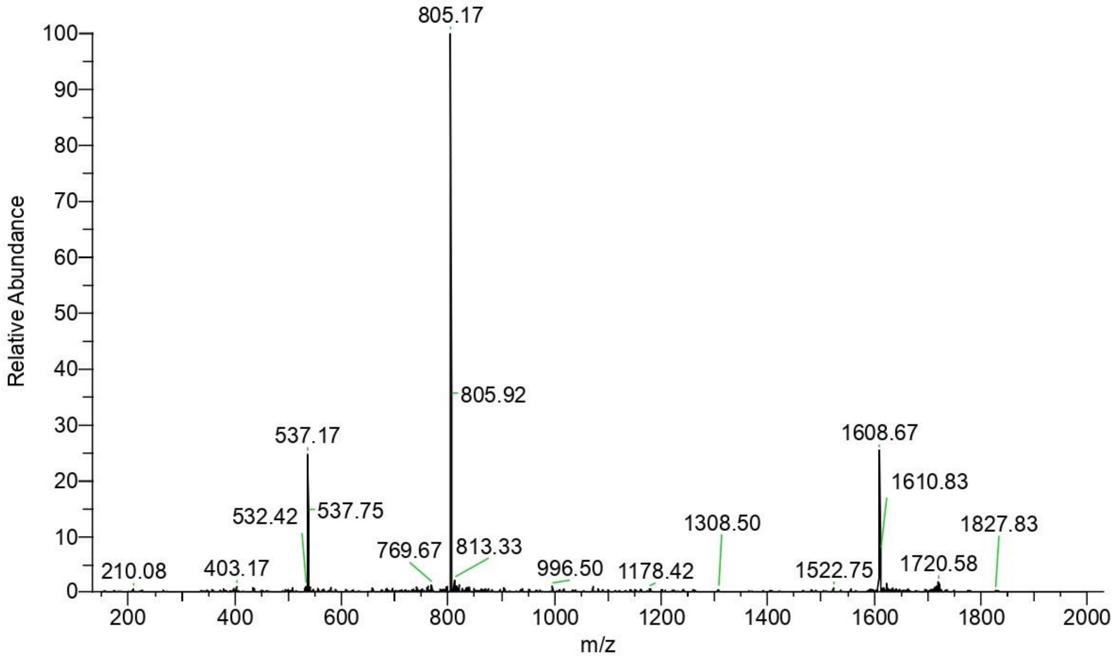

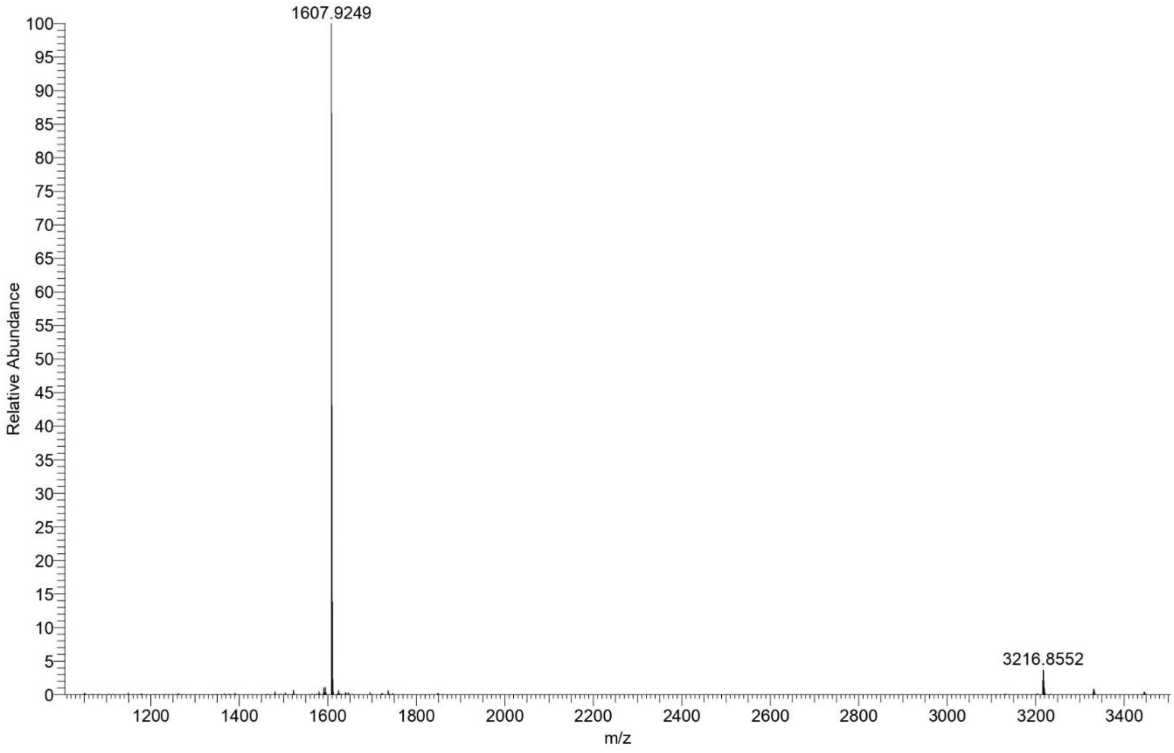
1607.9249
3216.8552
Relative Abundance
m/z

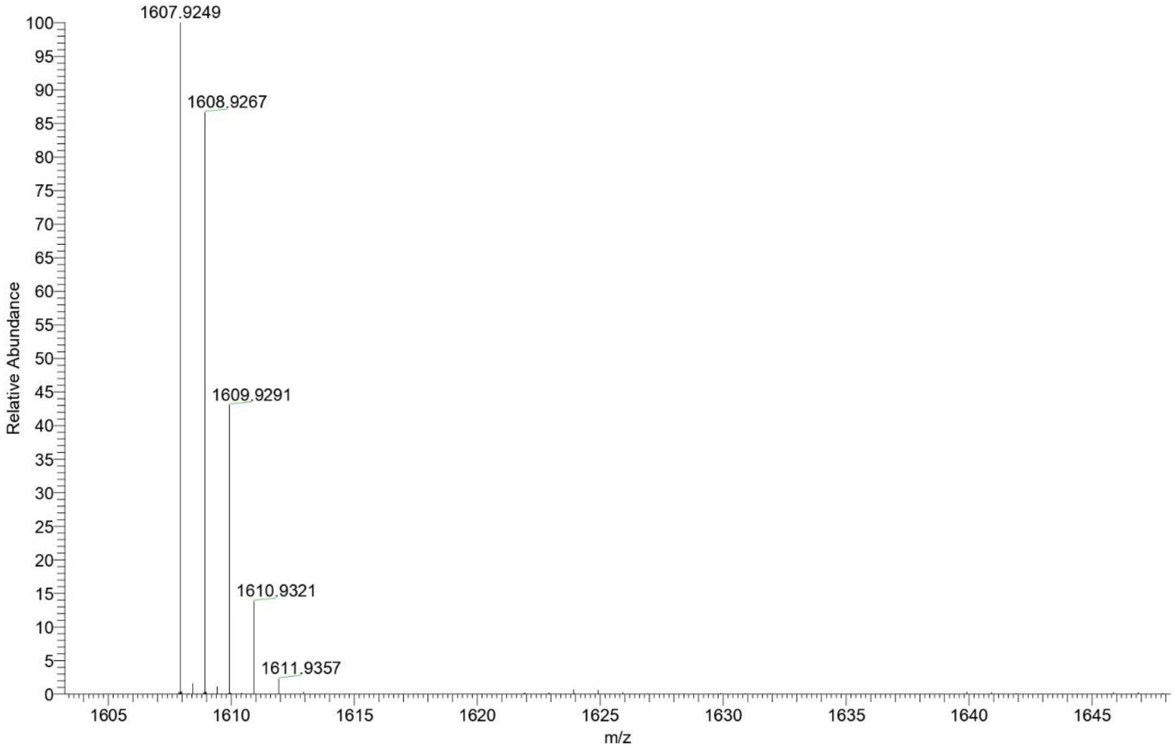
1607.9249
1608.9267
1609.9291
1610.9321
1611.9357
Relative Abundance
m/z

B19 (NWKKILGKVIDDMKM-NH_2) was obtained after manual synthesis from Rink Amide AM resin LL (300 mg, 0.29 mmol/g), the peptide was obtained as a white foamy solid after preparative RP-HPLC purification (40 mg, 22.5%). Analytical RP-HPLC: t_R = 1.56 min (100% A to 100% D in 3.5 min, λ= 214 nm). MS (ESI+): $C_{82}H_{140}N_{22}O_{20}S_2$ calc./obs. 1817.00/1817.00 $[M]^+$

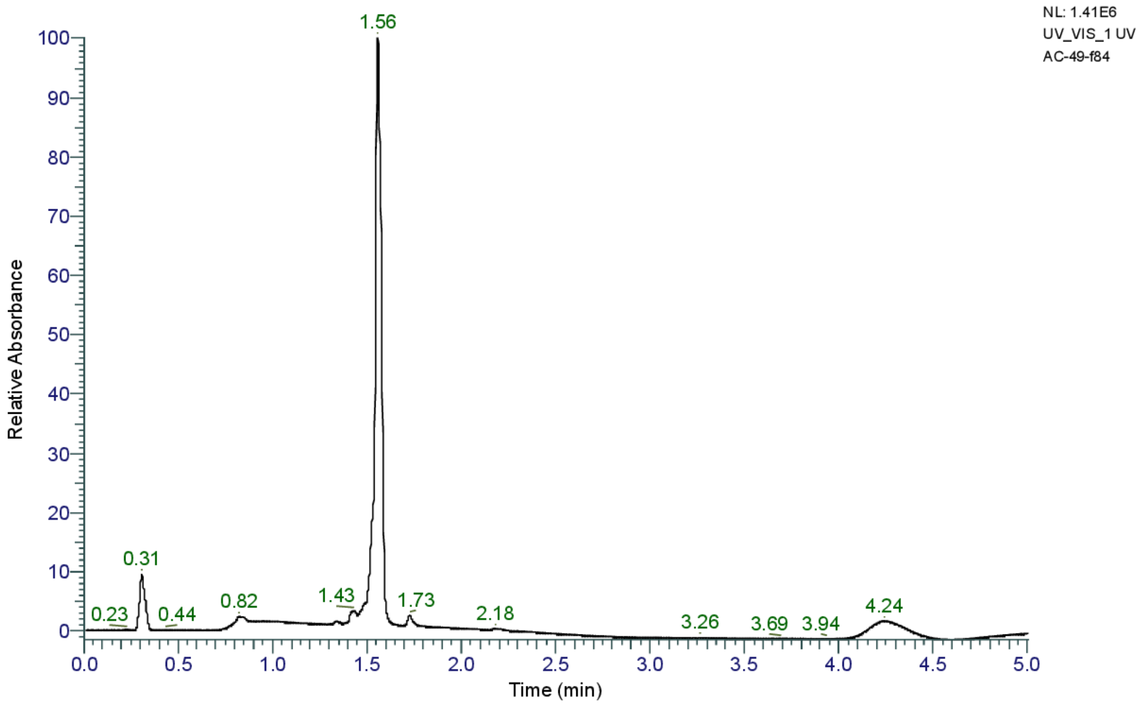

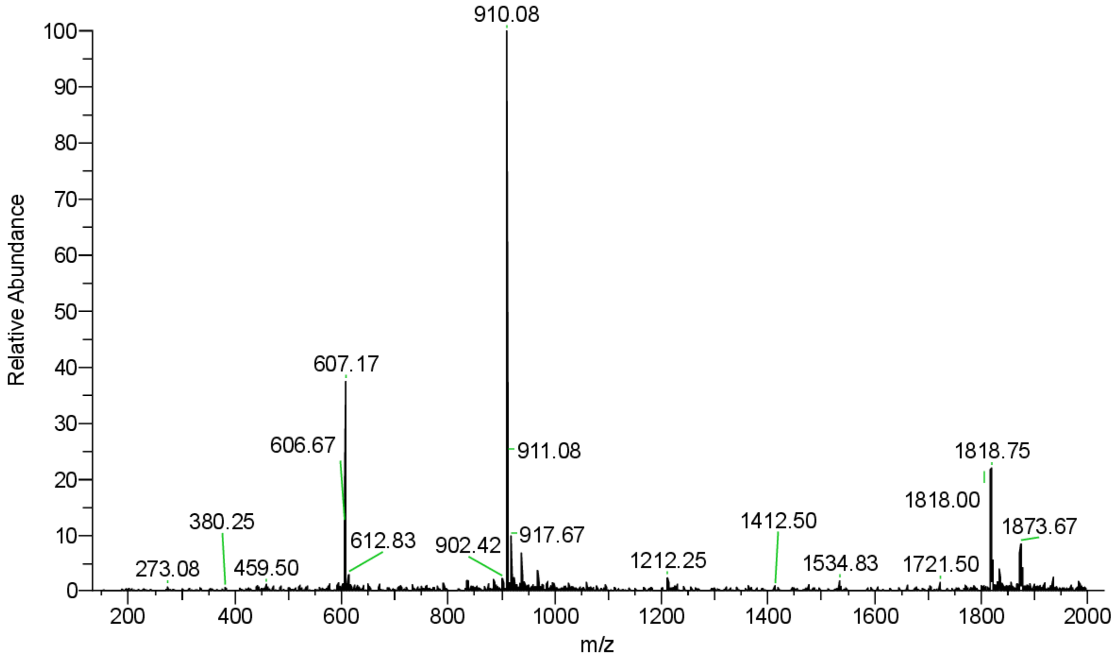

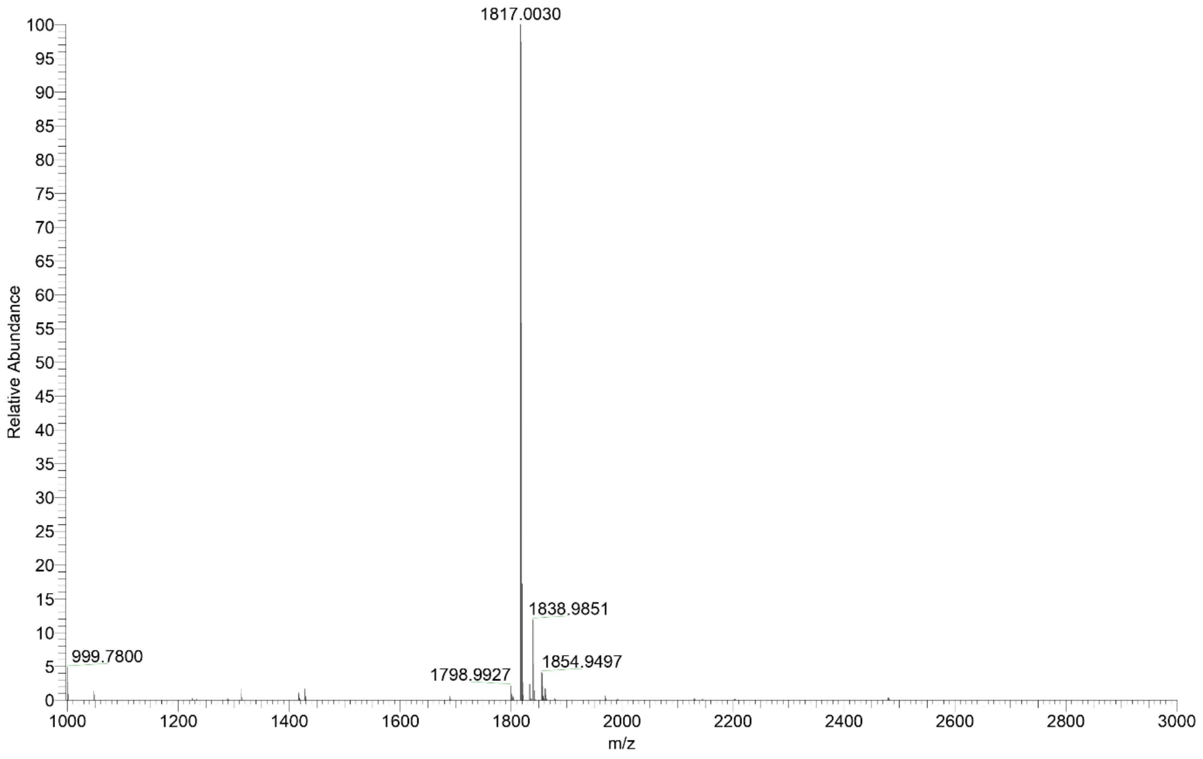
1817.0030
1838.9851
999.7800
1798.9927
1854.9497
Relative Abundance
m/z

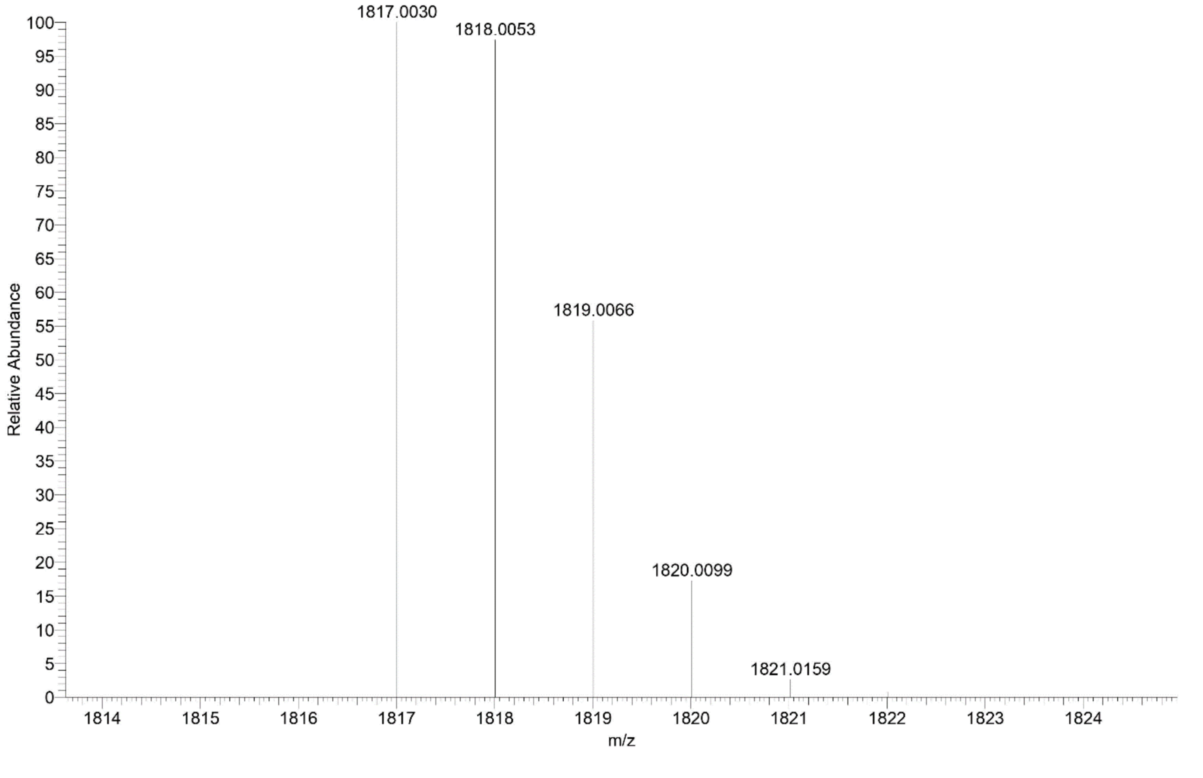
1817.0030
1818.0053
1819.0066
1820.0099
1821.0159
Relative Abundance
m/z

B20 (DKFSEKLGKIIKIVK-NH_2) was obtained after manual synthesis from Rink Amide AM resin LL (300 mg, 0.29 mmol/g), the peptide was obtained as a white foamy solid after preparative RP-HPLC purification (30 mg, 16.5%). Analytical RP-HPLC: t_R = 1.42 min (100% A to 100% D in 3.5 min, λ= 214 nm). MS (ESI+): $C_{82}H_{145}N_{21}O_{20}$ calc./obs. 1744.01/1744.01 $[M]^+$

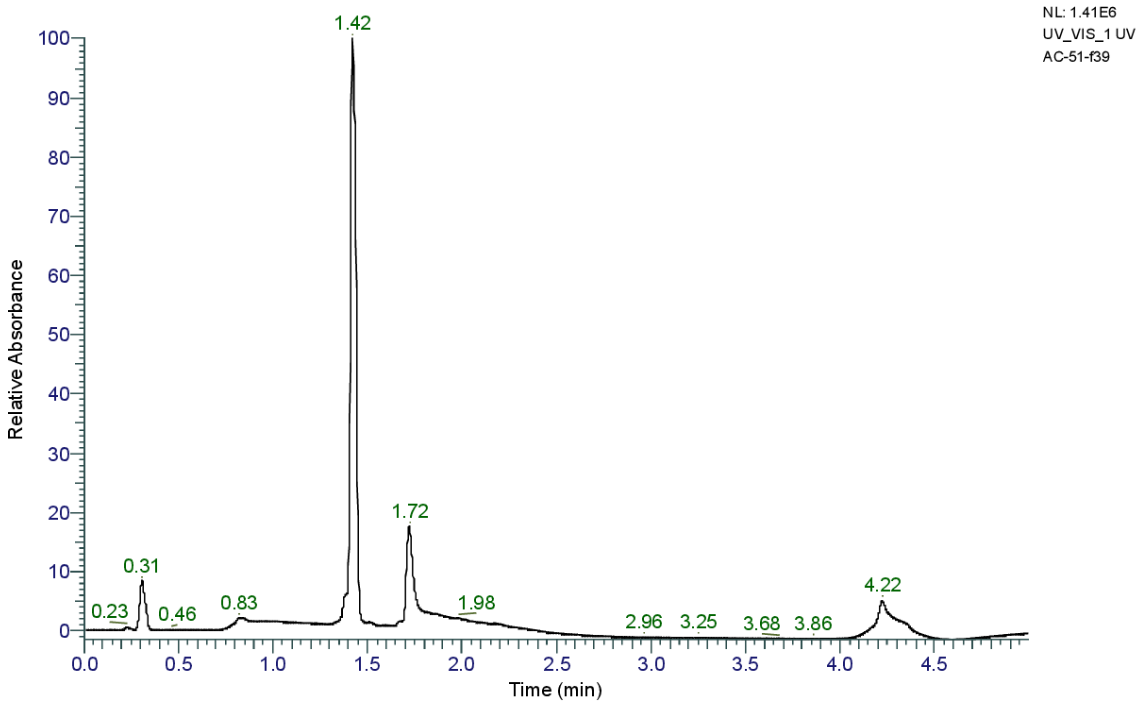

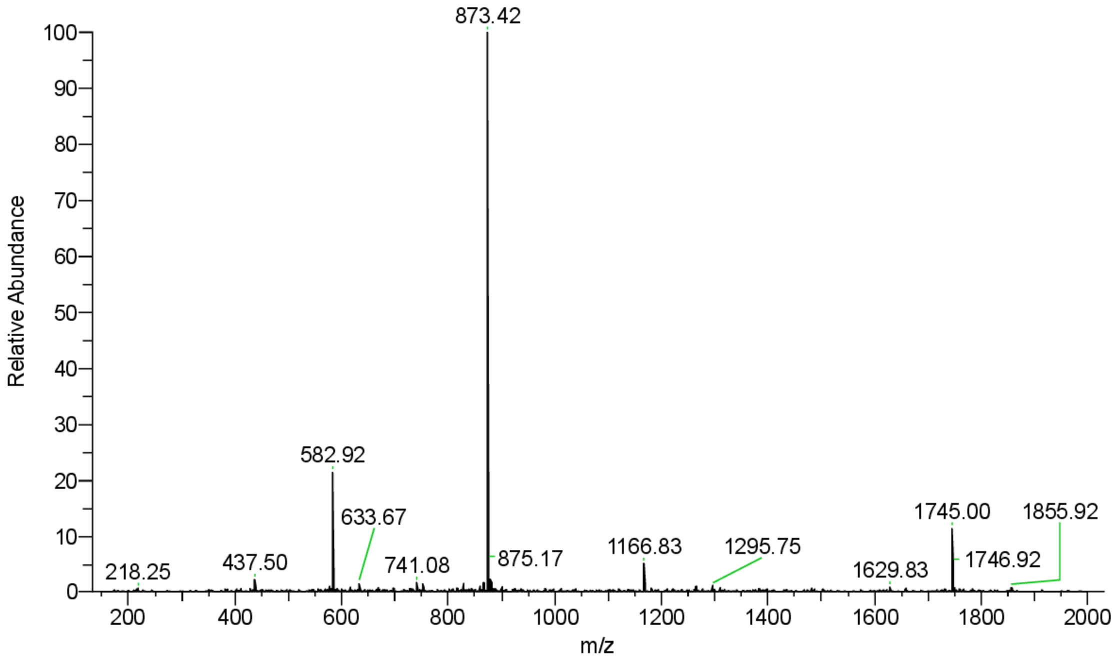

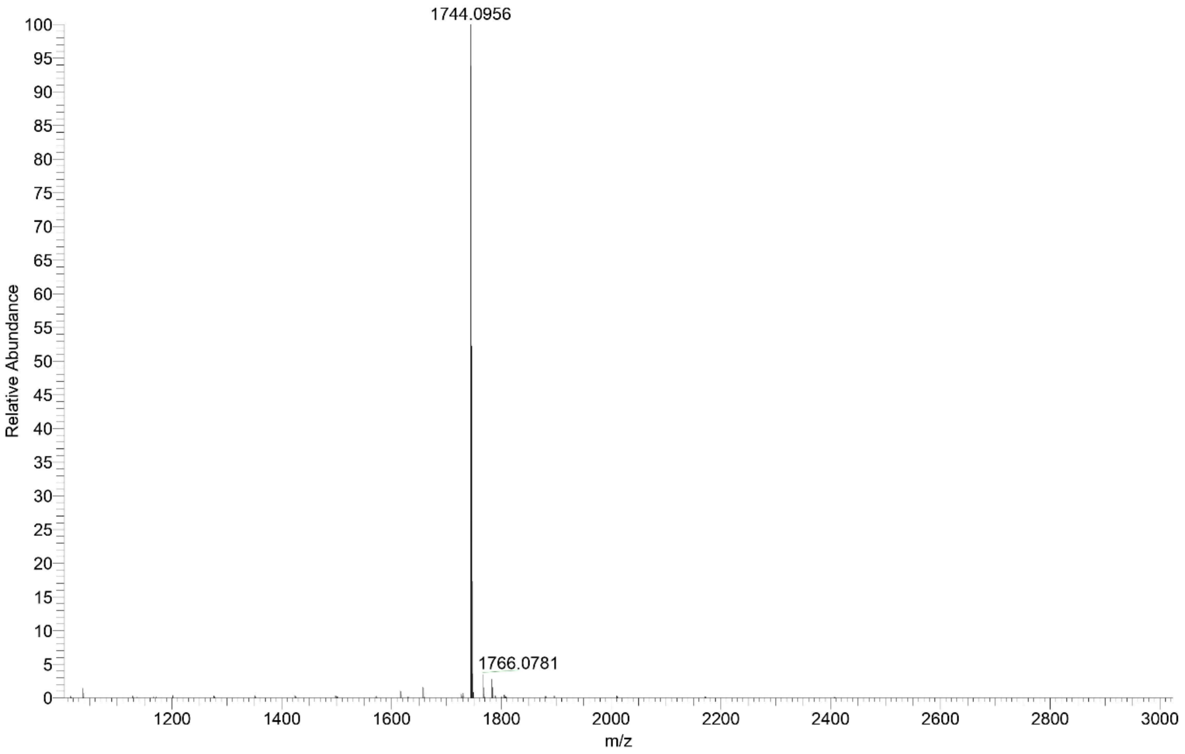

1744.0956
1766.0781
Relative Abundance
m/z

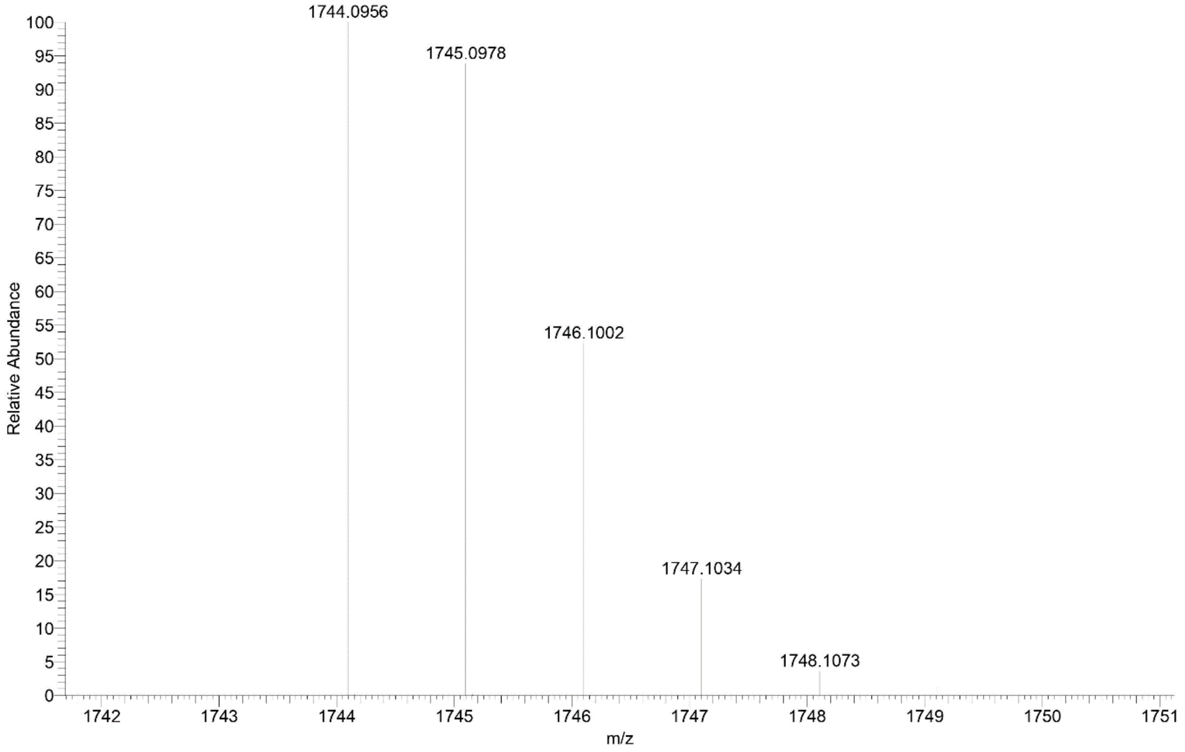

1744.0956
1745.0978
1746.1002
1747.1034
1748.1073
Relative Abundance
m/z

DA1 (fakkffkkfakfafk -NH_2) was obtained from D-enantiomeric amino acids after manual synthesis from Rink Amide AM resin LL (300 mg, 0.29 mmol/g), the peptide was obtained as a white foamy solid after preparative RP-HPLC purification (25.5 mg, 23.0%). Analytical RP-HPLC: t_R = 1.39 min (100% A to 100% D in 3.5 min, λ= 214 nm). MS (ESI+): $C_{99}H_{144}N_{22}O_{15}$ calc./obs. 1881.12/1881.12 $[M]^+$

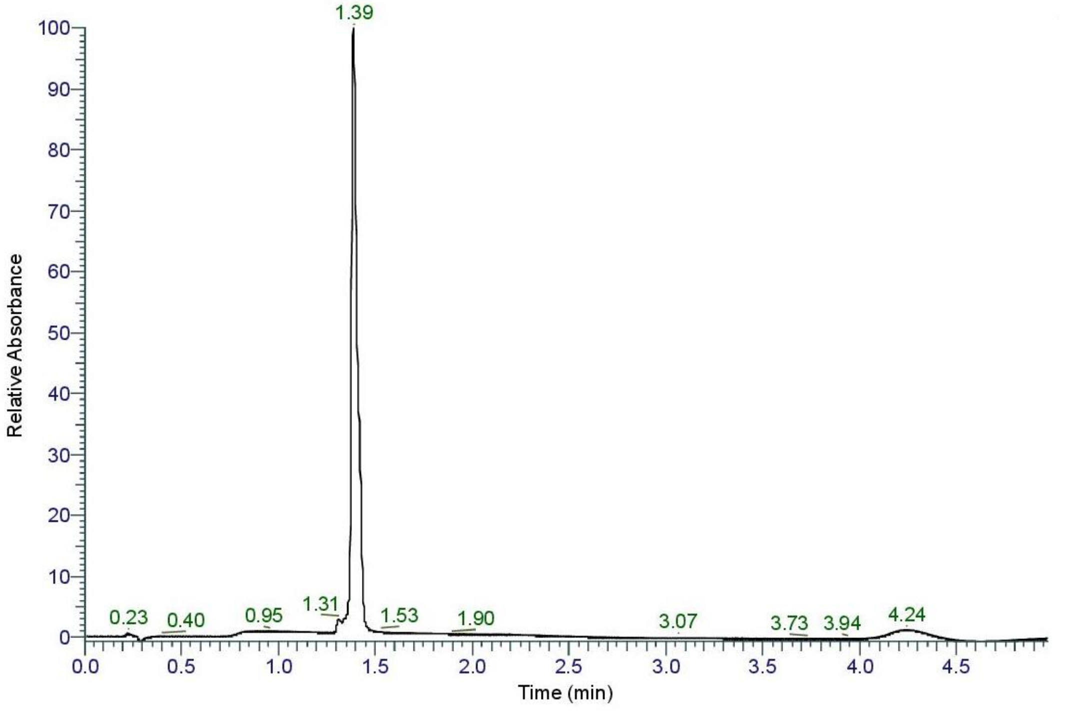

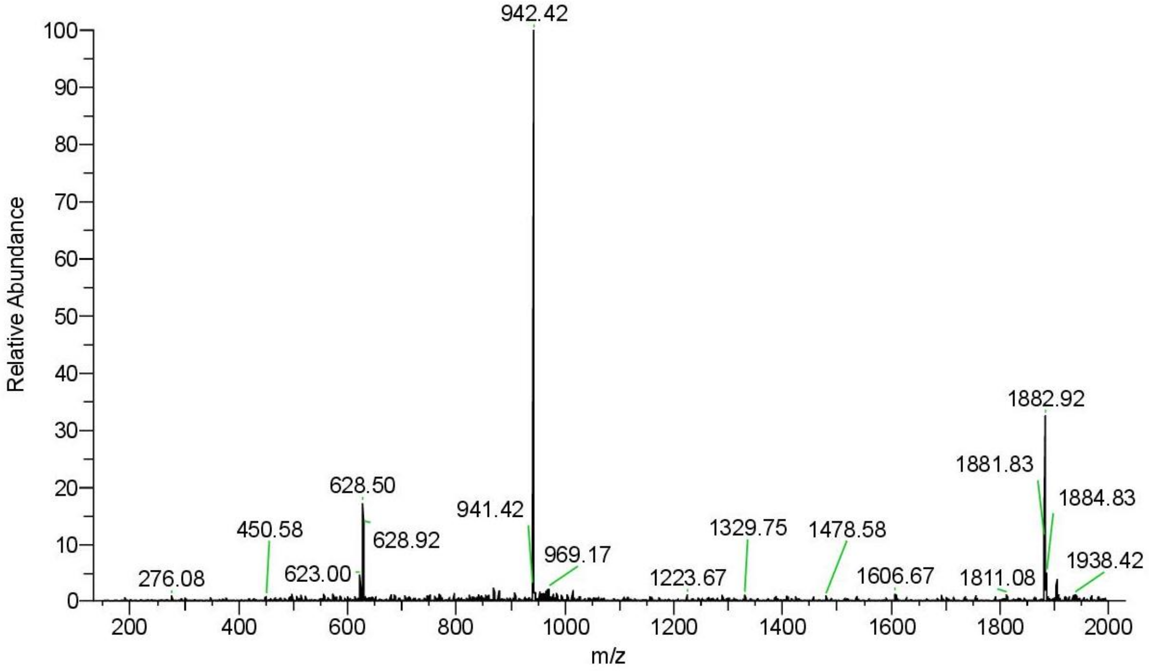

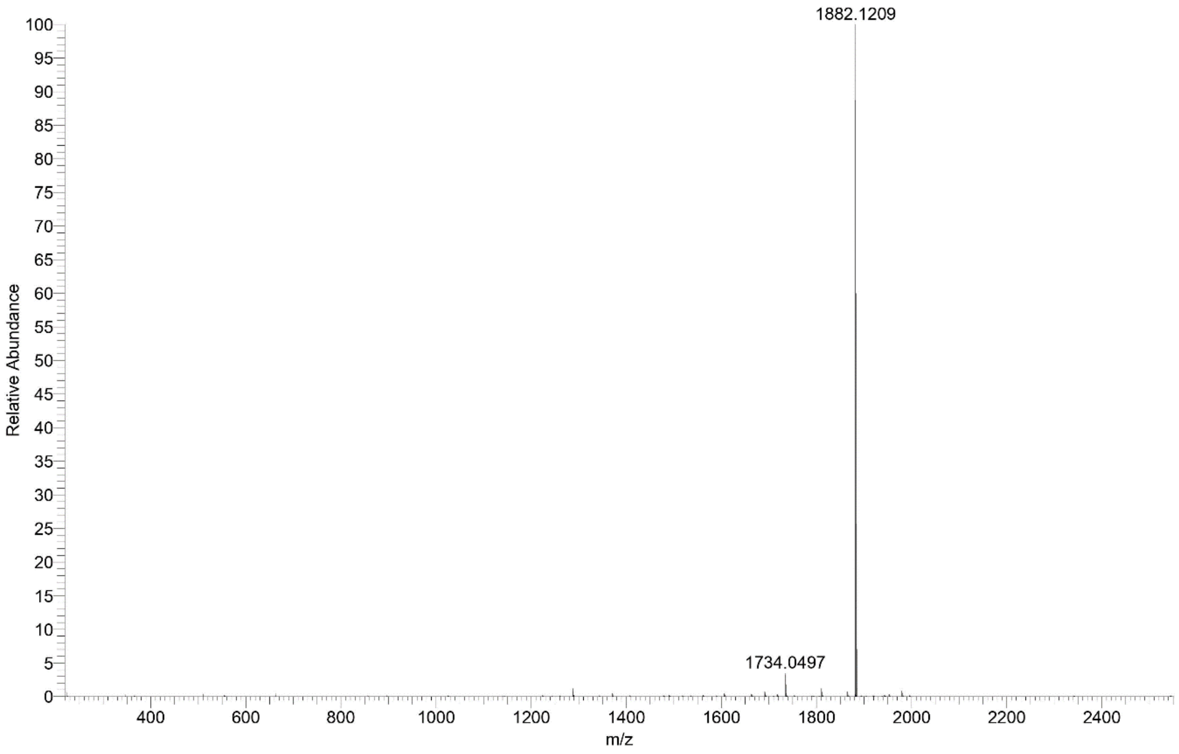

1882.1209
1734.0497
Relative Abundance
m/z

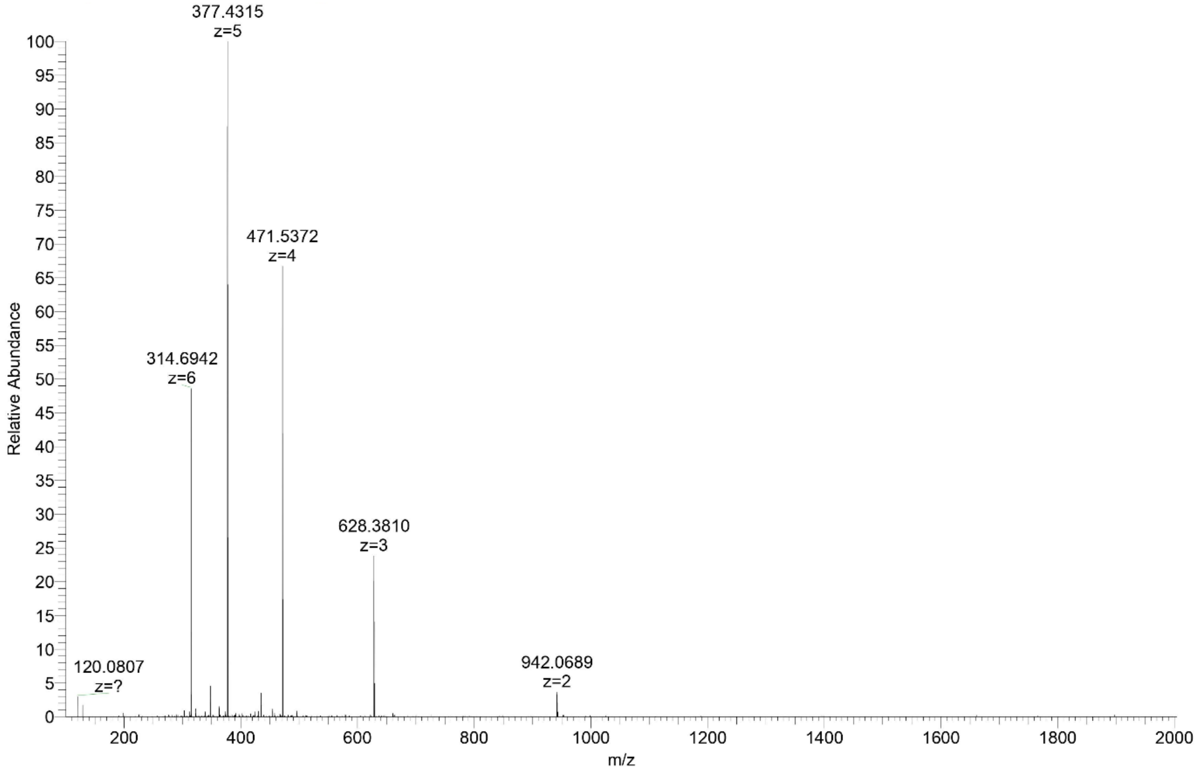

377.4315
z=5
471.5372
z=4
314.6942
z=6
628.3810
z=3
120.0807
z=?
942.0689
z=2
Relative Abundance
m/z

DLL-III (vnwkkilgkiikvvk-NH_2) was obtained from D-enantiomeric amino acids after manual synthesis from Rink Amide AM resin LL (300 mg, 0.29 mmol/g), the peptide was obtained as a white foamy solid after preparative RP-HPLC purification (26 mg,17.0%). Analytical RP-HPLC: t_R = 1.53 min (100% A to 100% D in 3.5 min, λ= 214 nm). MS (ESI+): $C_{86}H_{153}N_{23}O_{16}$ calc./obs. 1764.19/1764.20 $[M]^+$

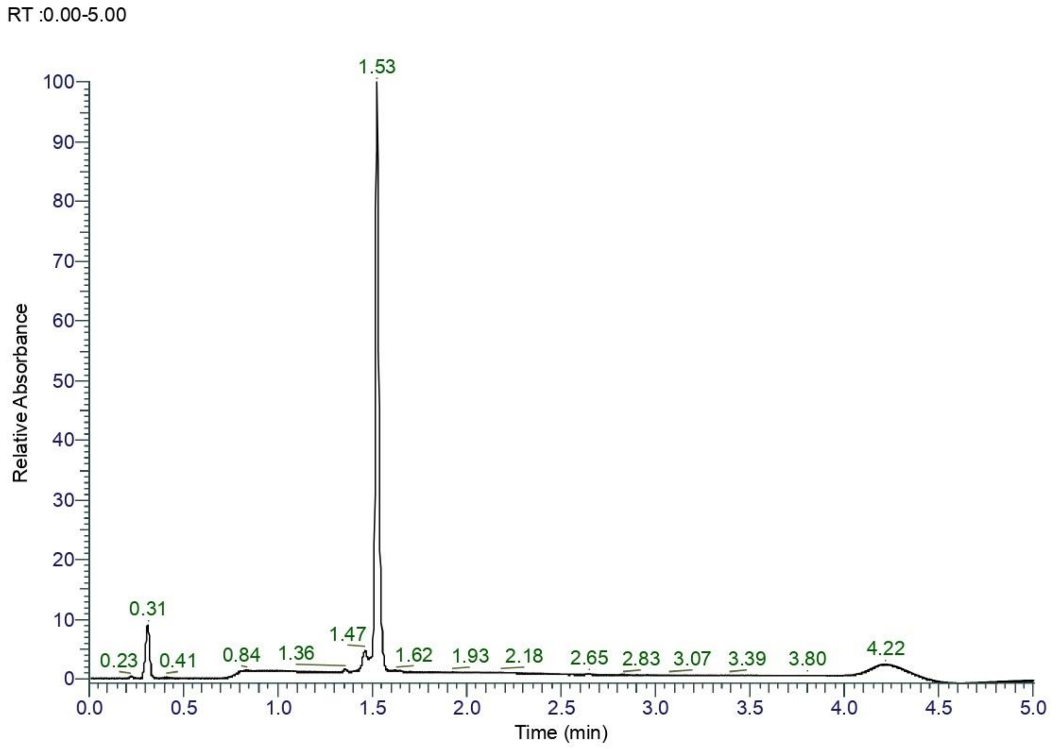

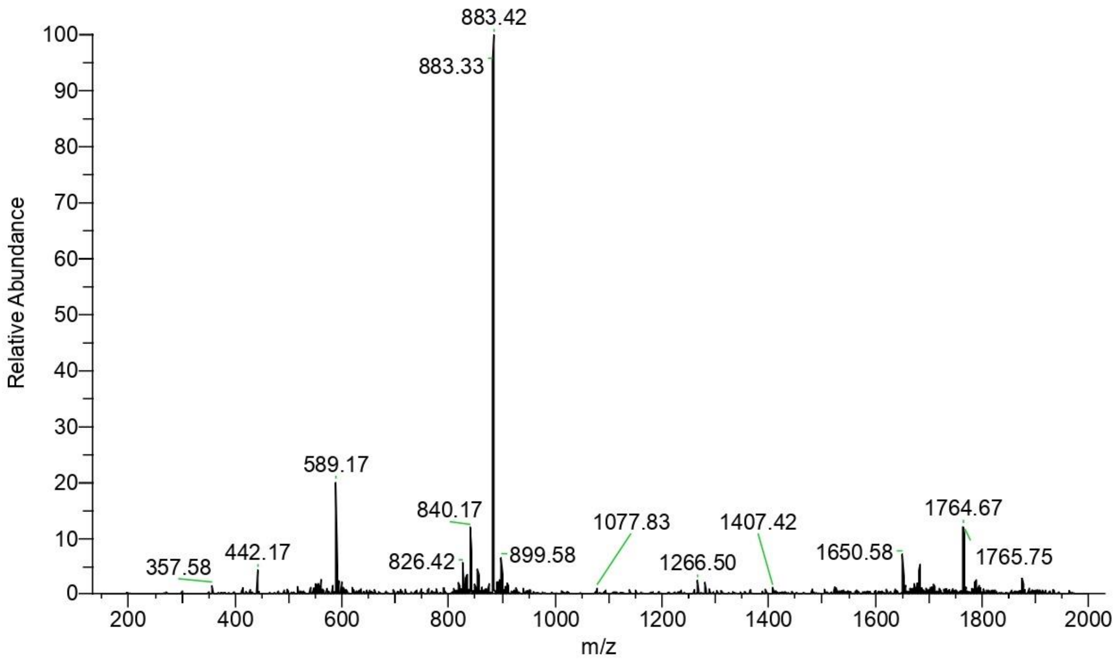

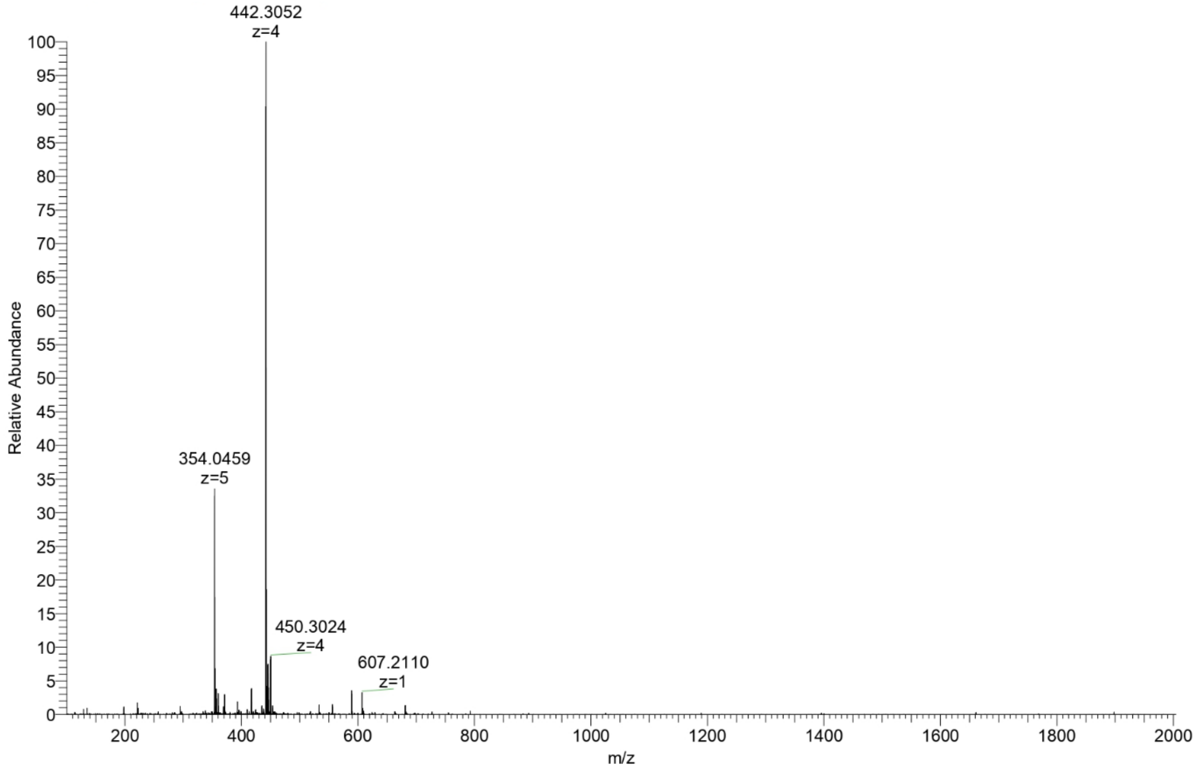
442.3052
z=4
354.0459
z=5
450.3024
z=4
607.2110
z=1
Relative Abundance
m/z

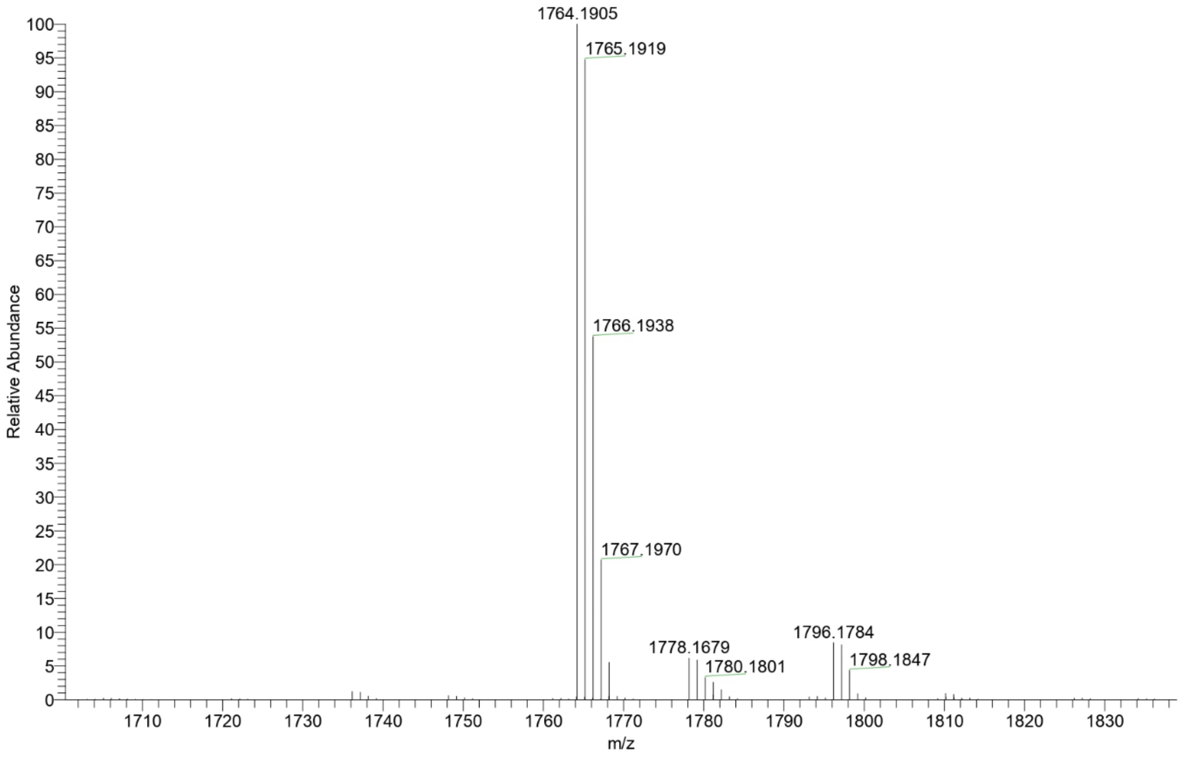
1764.1905
1765.1919
1766.1938
1767.1970
1778.1679
1780.1801
1796.1784
1798.1847
Relative Abundance
m/z

DB1 (vnwkkilgkiikvvk-NH_2) was obtained from D-enantiomeric amino acids after manual synthesis from Rink Amide AM resin LL (300 mg, 0.29 mmol/g), the peptide was obtained as a white foamy solid after preparative RP-HPLC purification (33 mg, 16.0%). Analytical RP-HPLC: t_R = 1.45 min (100% A to 100% D in 3.5 min, λ= 214 nm). MS (ESI+): $C_{89}H_{148}N_{24}O_{16}$ calc./obs. 1809.15/1809.16 $[M]^+$

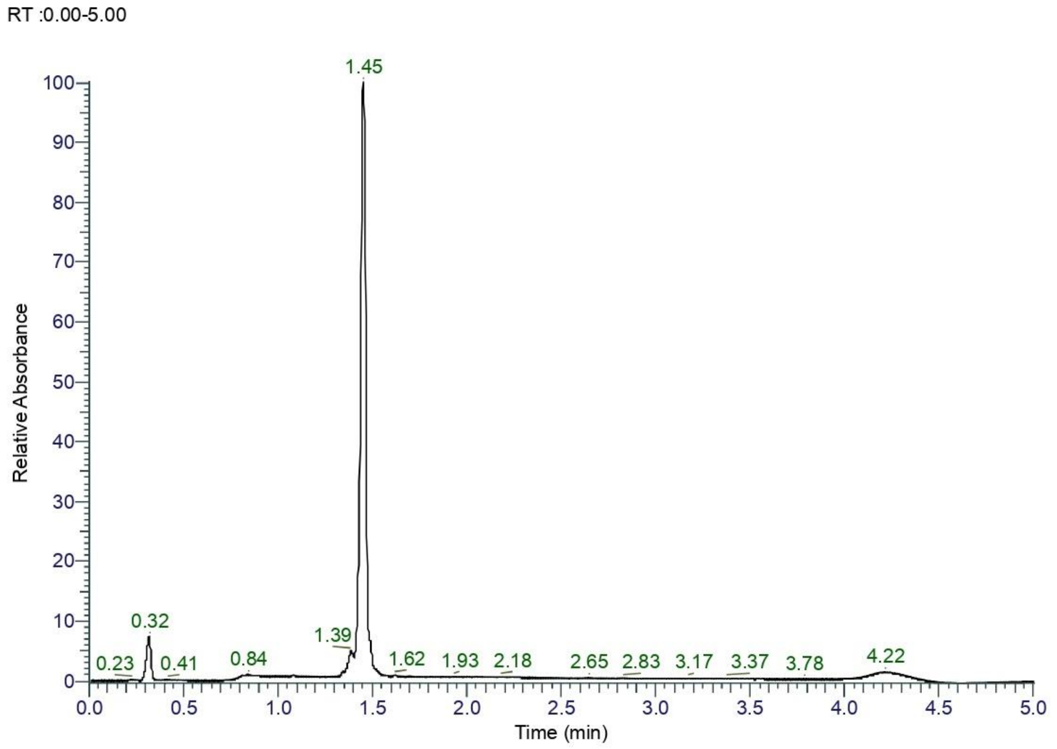

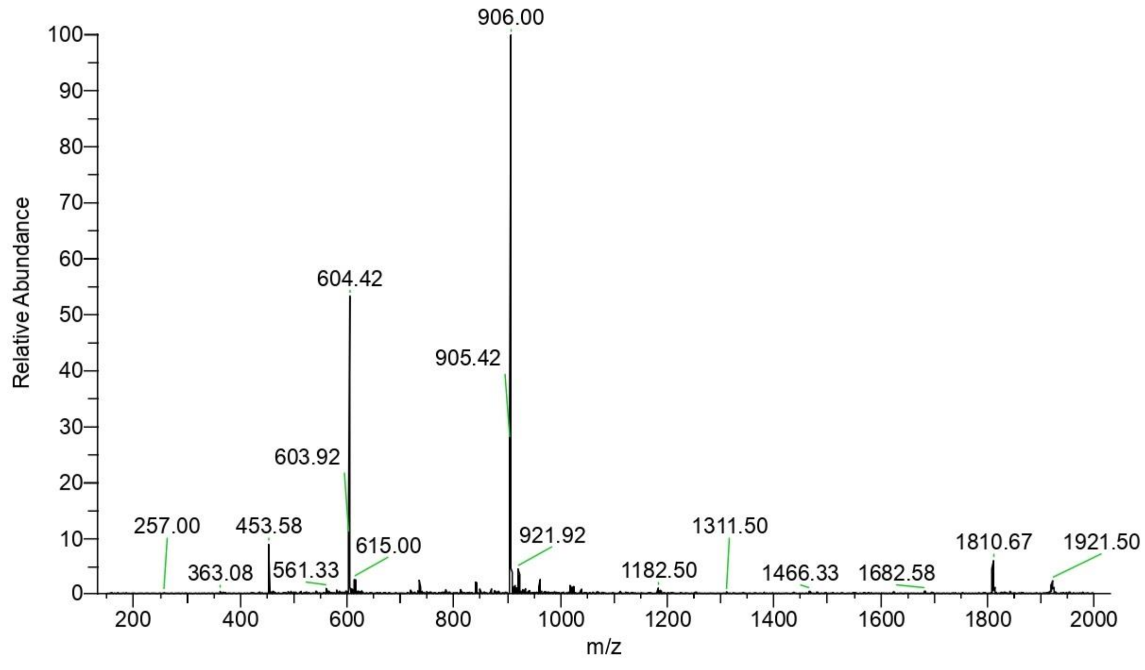

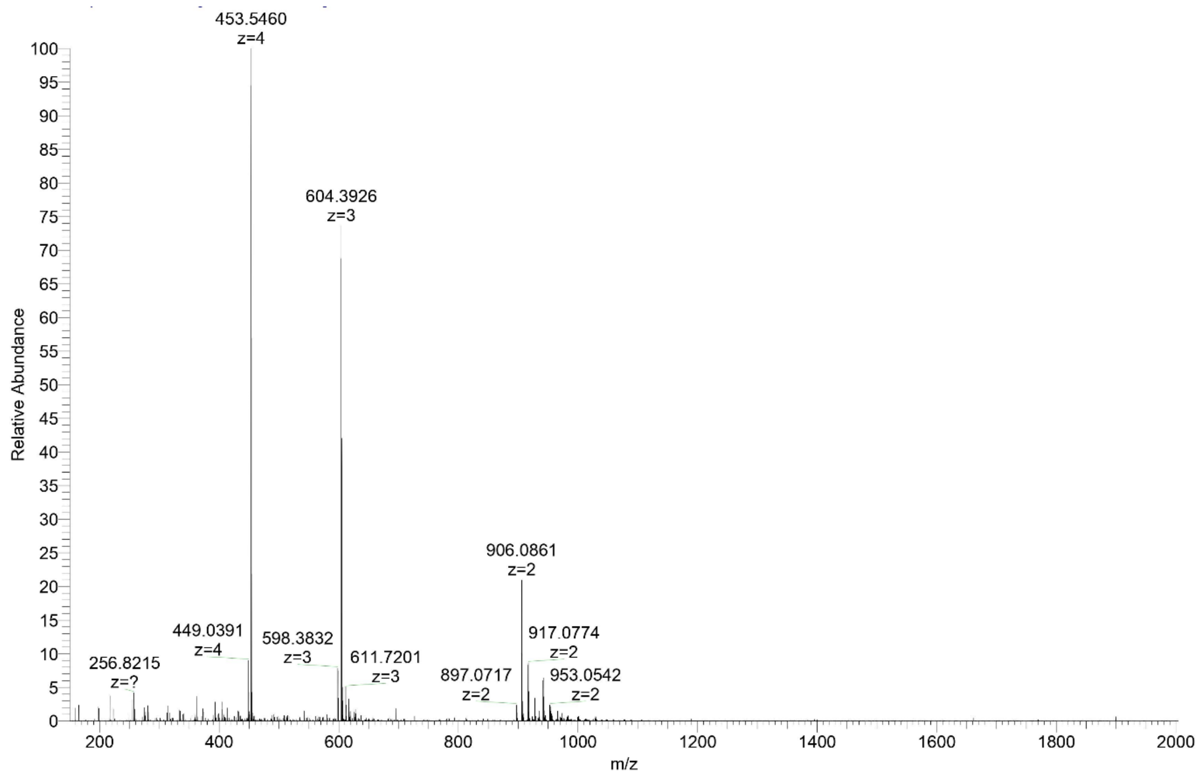
453.5460
z=4
604.3926
z=3
906.0861
z=2
449.0391
z=4
598.3832
z=3
611.7201
z=3
917.0774
z=2
897.0717
z=2
953.0542
z=2
256.8215
z=?
Relative Abundance
m/z

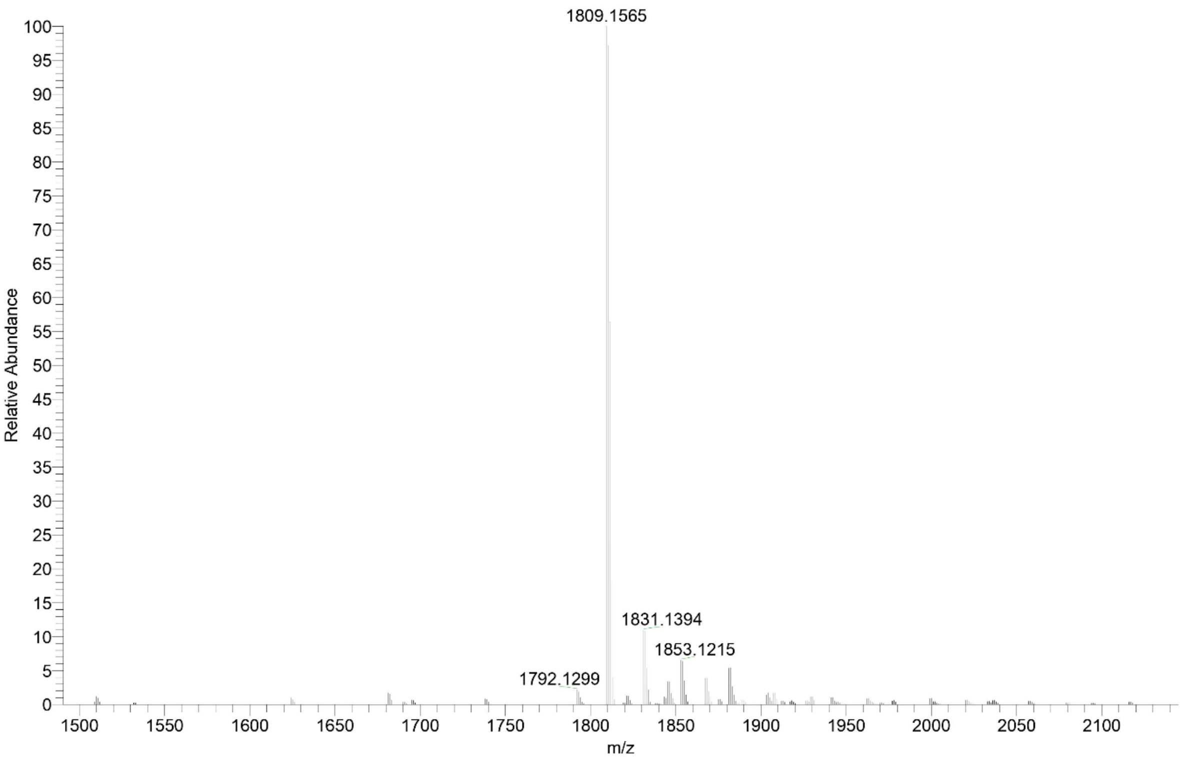
1809.1565
1831.1394
1853.1215
1792.1299
Relative Abundance
m/z

FB1 was obtained from **B1** by procedure mentioned above. Rink Amide AM resin LL (100 mg, 0.29 mmol/g) was used, and the product was obtained as a bright yellow foamy solid after preparative RP-HPLC purification (16.8 mg, 27.6%). Analytical RP-HPLC: t_R = 1.70 min (100% A to 100% D in 3.5 min, λ= 214 nm). MS (ESI+): $C_{110}H_{158}N_{24}O_{22}$ calc./obs. 2167.20/2167.21[M]$^+$

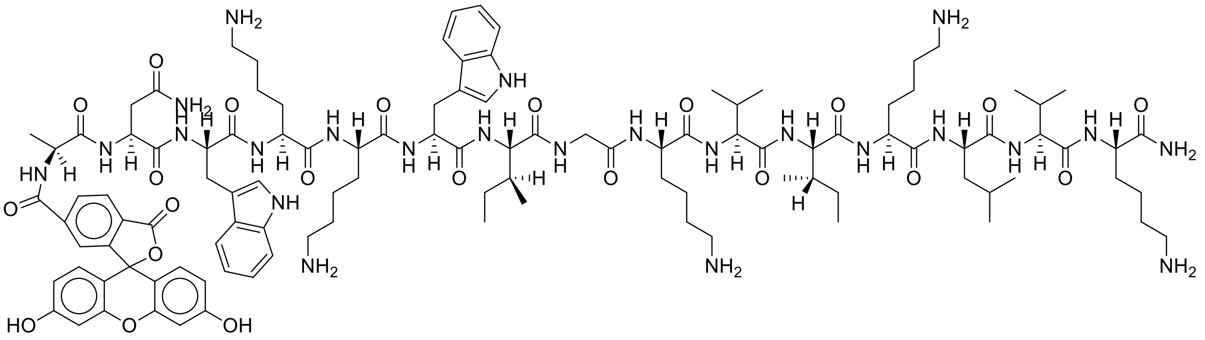

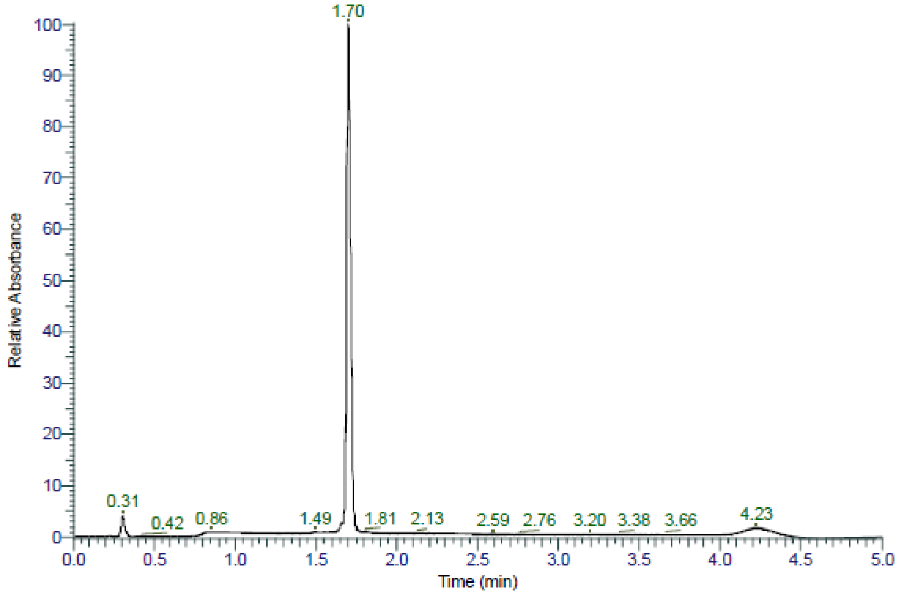

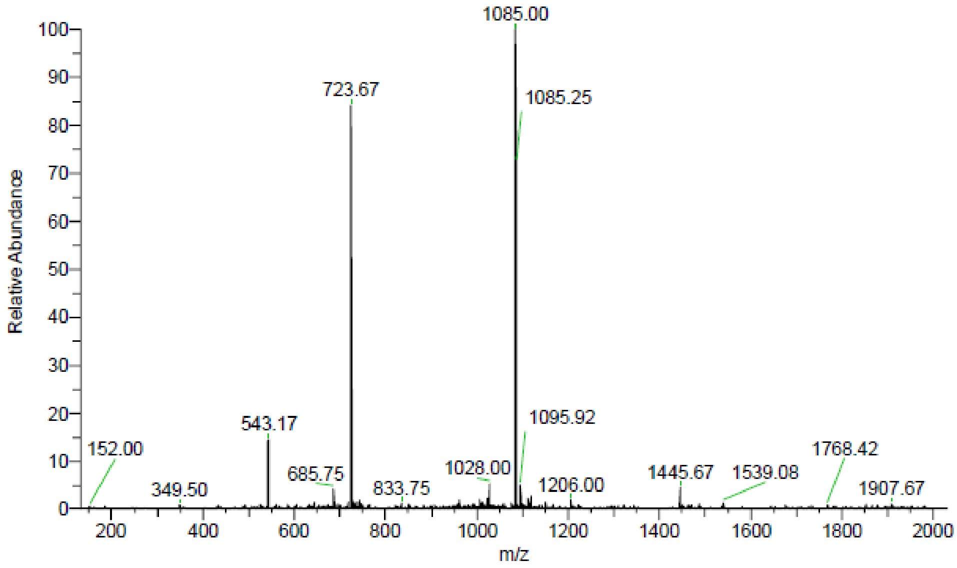

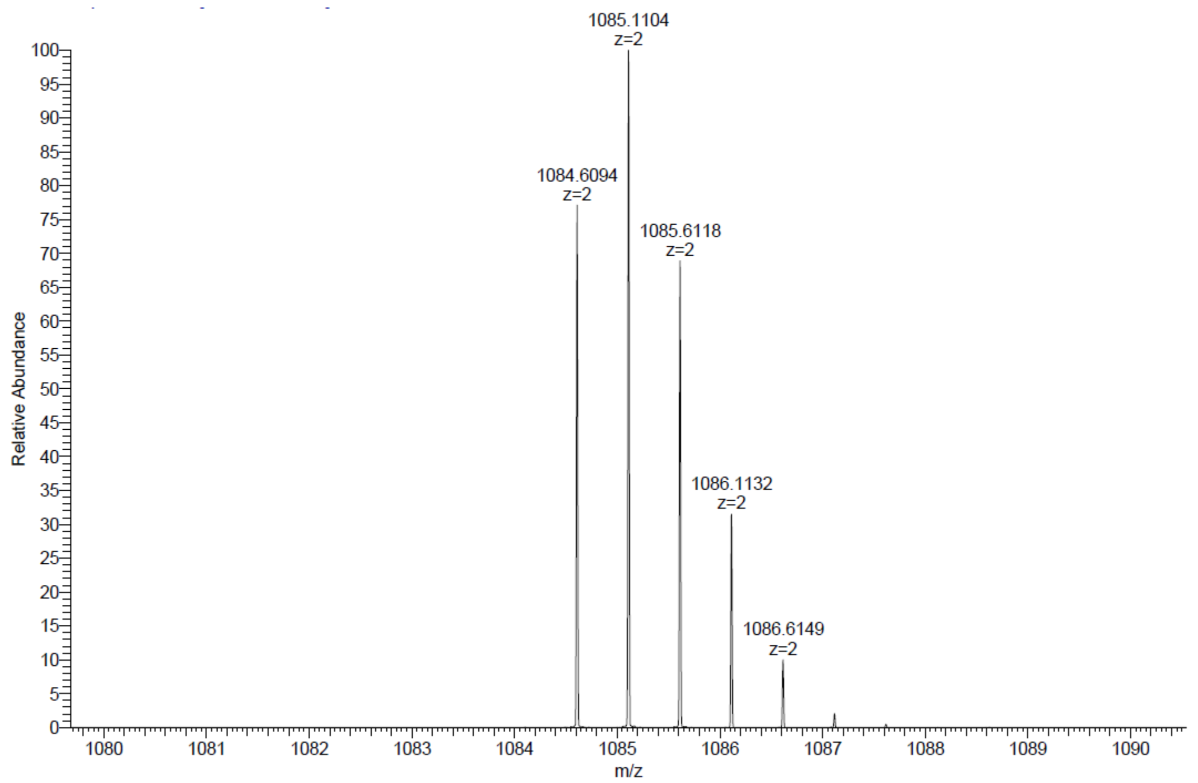
1085.1104
z=2
1084.6094
z=2
1085.6118
z=2
1086.1132
z=2
1086.6149
z=2
Relative Abundance
m/z

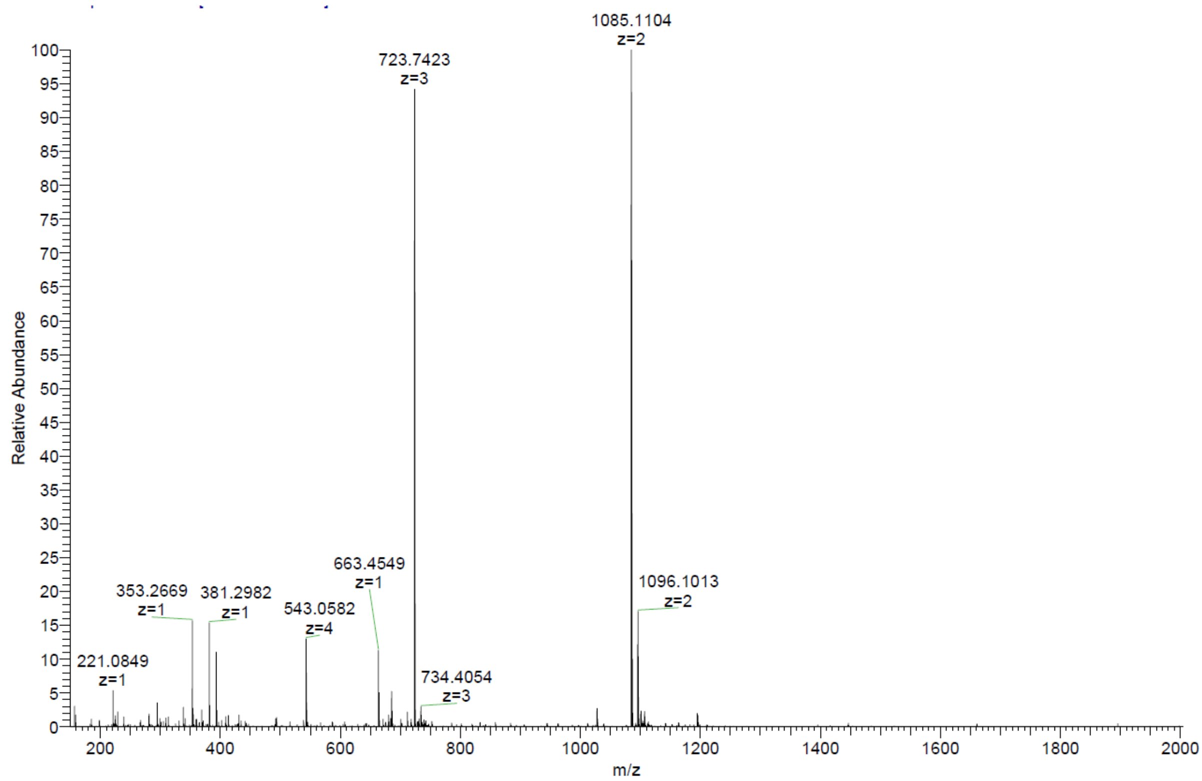
1085.1104
z=2
723.7423
z=3
353.2669
z=1
381.2982
z=1
663.4549
z=1
543.0582
z=4
1096.1013
z=2
221.0849
z=1
734.4054
z=3
Relative Abundance
m/z

FDB1 was obtained from **DB1** by procedure mentioned above. Rink Amide AM resin LL (100 mg, 0.29 mmol/g) was used, and the product was obtained as a bright yellow foamy solid after preparative RP-HPLC purification (9.3 mg, 12.1%). Analytical RP-HPLC: t_R = 1.60 min (100% A to 100% D in 3.5 min, λ= 214 nm). MS (ESI+): $C_{110}H_{158}N_{24}O_{22}$ calc./obs. 2167.20/2167.22 $[M]^+$

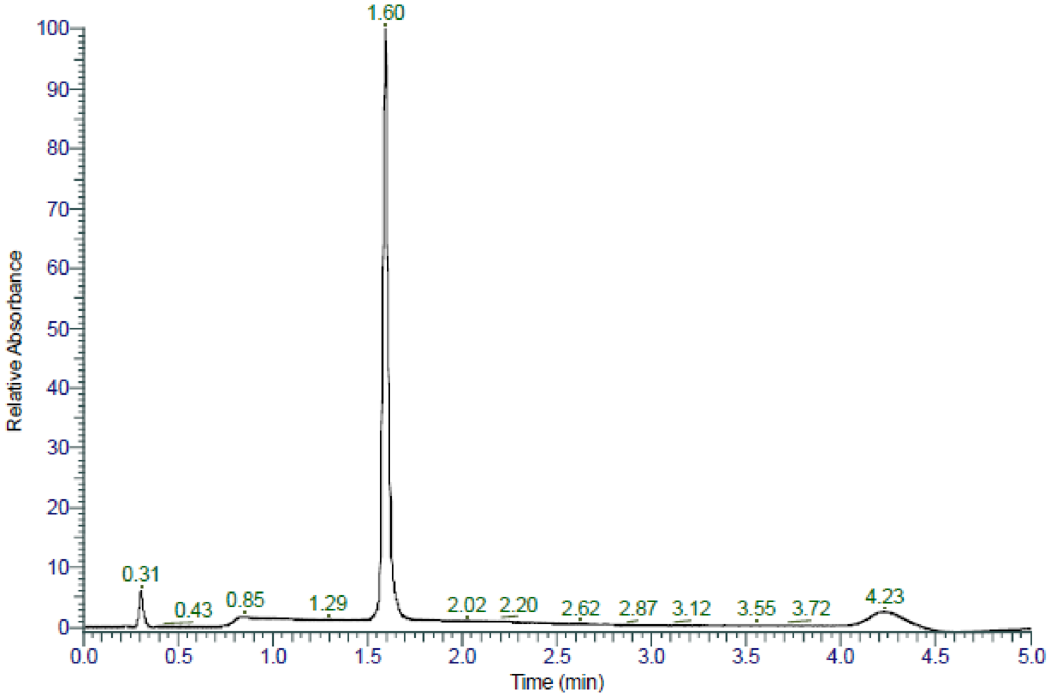

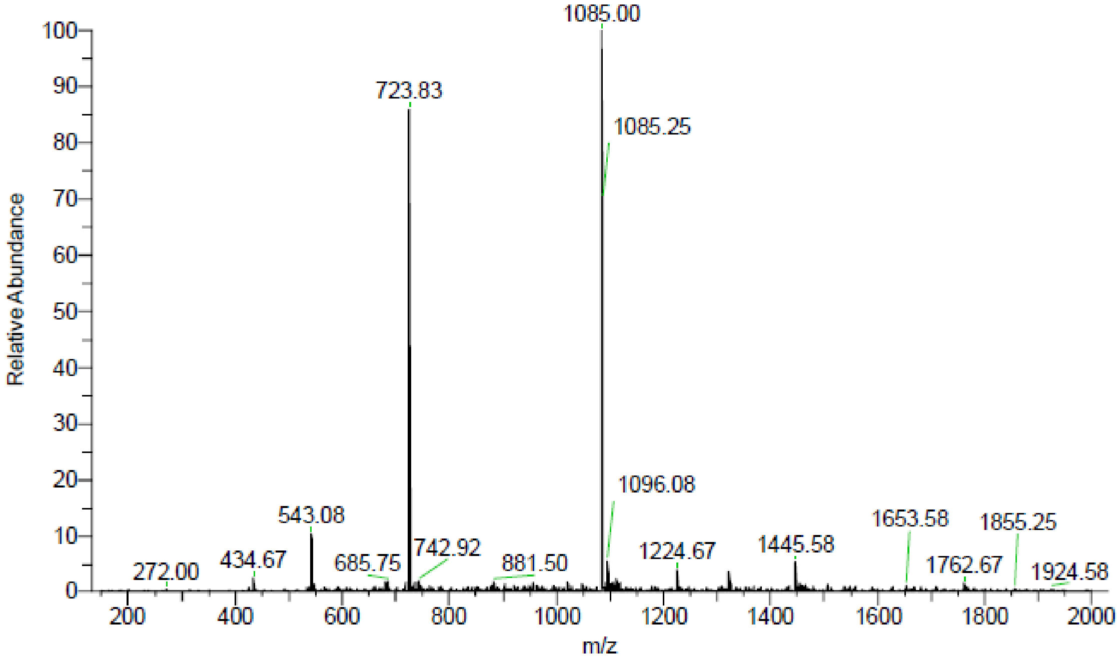

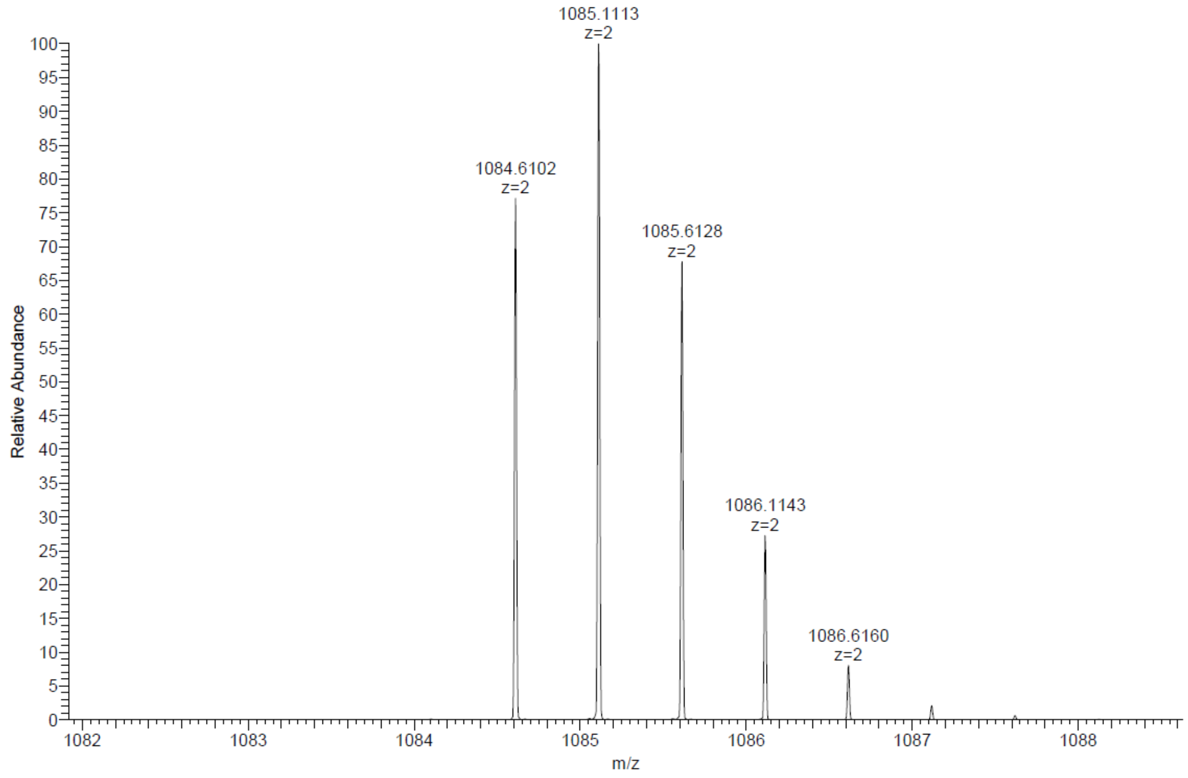
1085.1113
z=2
1084.6102
z=2
1085.6128
z=2
1086.1143
z=2
1086.6160
z=2
Relative Abundance
m/z

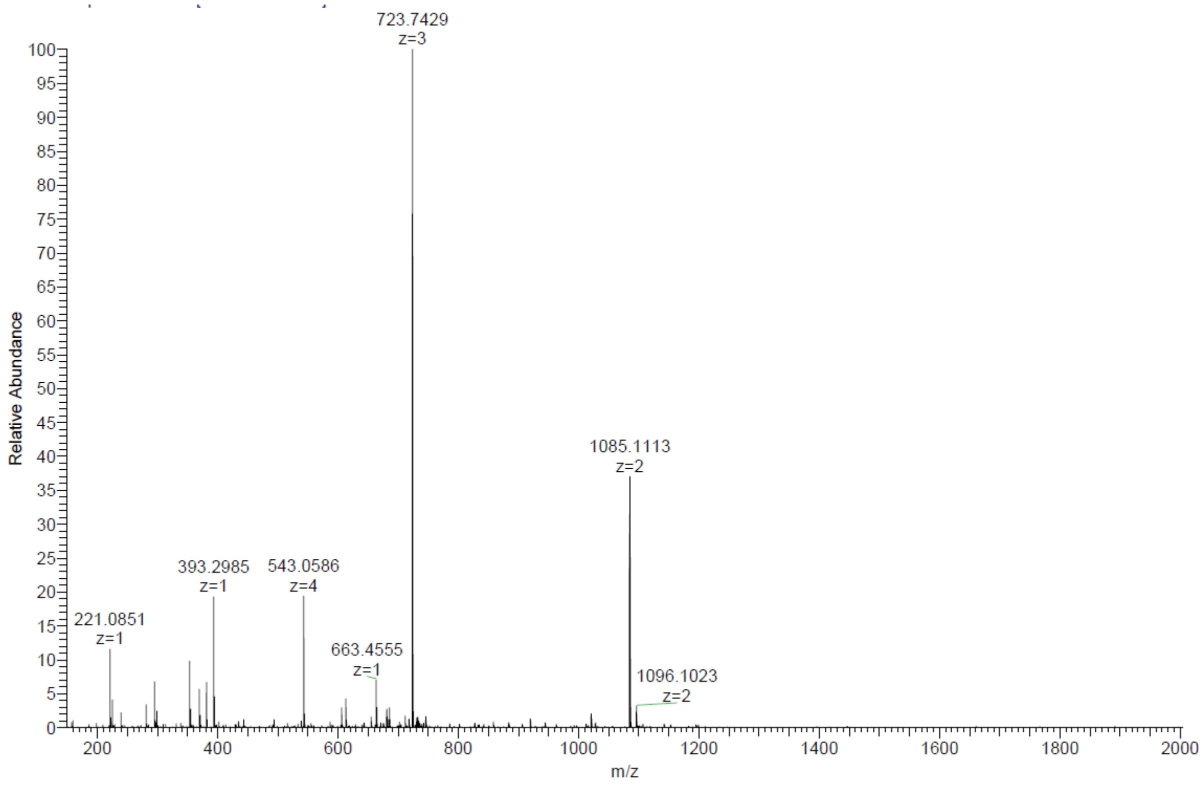
723.7429
z=3
1085.1113
z=2
393.2985
z=1
543.0586
z=4
221.0851
z=1
663.4555
z=1
1096.1023
z=2
Relative Abundance
m/z

FLL-III was obtained from **LL-III** by procedure mentioned above. Rink Amide AM resin LL (100 mg, 0.29 mmol/g) was used, and the product was obtained as a bright yellow foamy solid after preparative RP-HPLC purification (15.5 mg, 26.0%). Analytical RP-HPLC: t_R = 1.77 min (100% A to 100% D in 3.5 min, λ= 214 nm). MS (ESI+): $C_{107}H_{163}N_{23}O_{22}$ calc./obs. 2122.23/2122.25 $[M]^+$

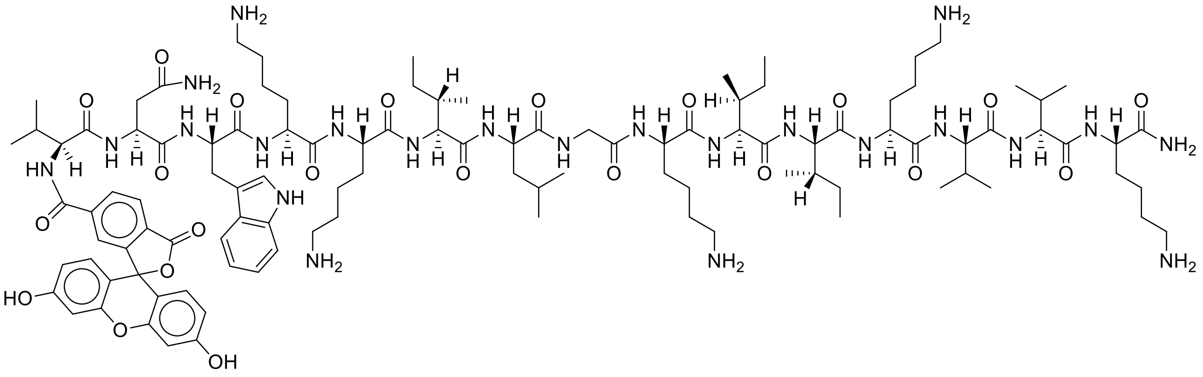

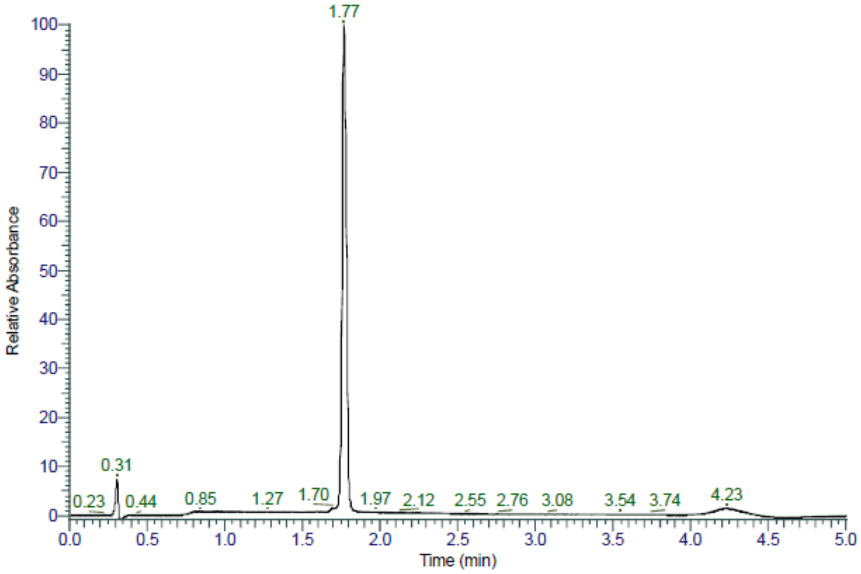

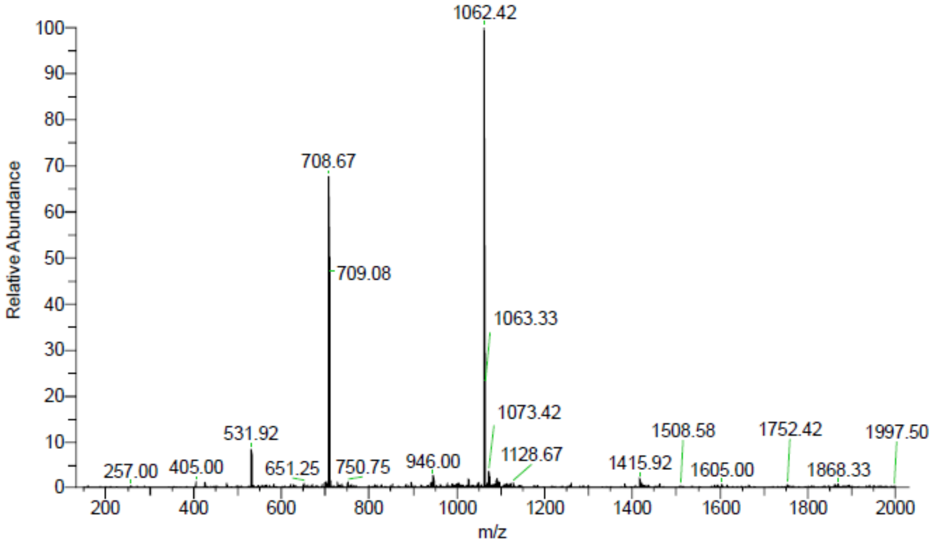

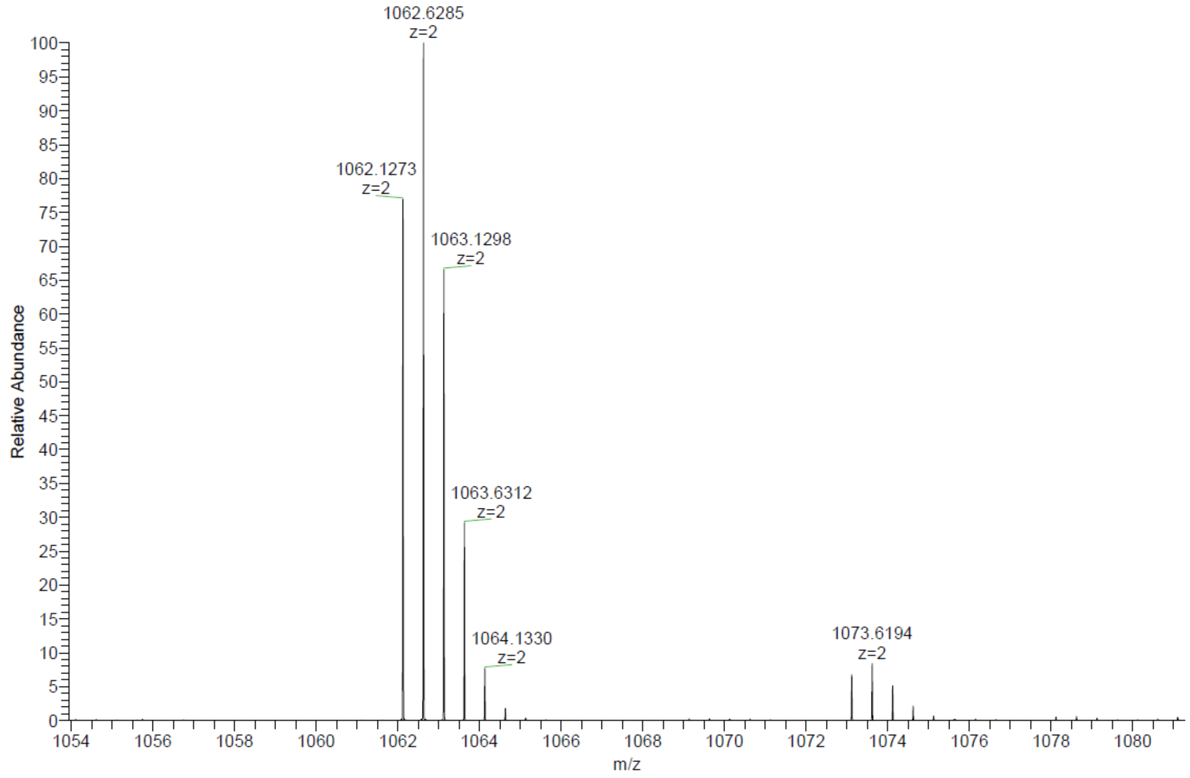
1062.6285
z=2
1062.1273
z=2
1063.1298
z=2
1063.6312
z=2
1064.1330
z=2
1073.6194
z=2
Relative Abundance
m/z

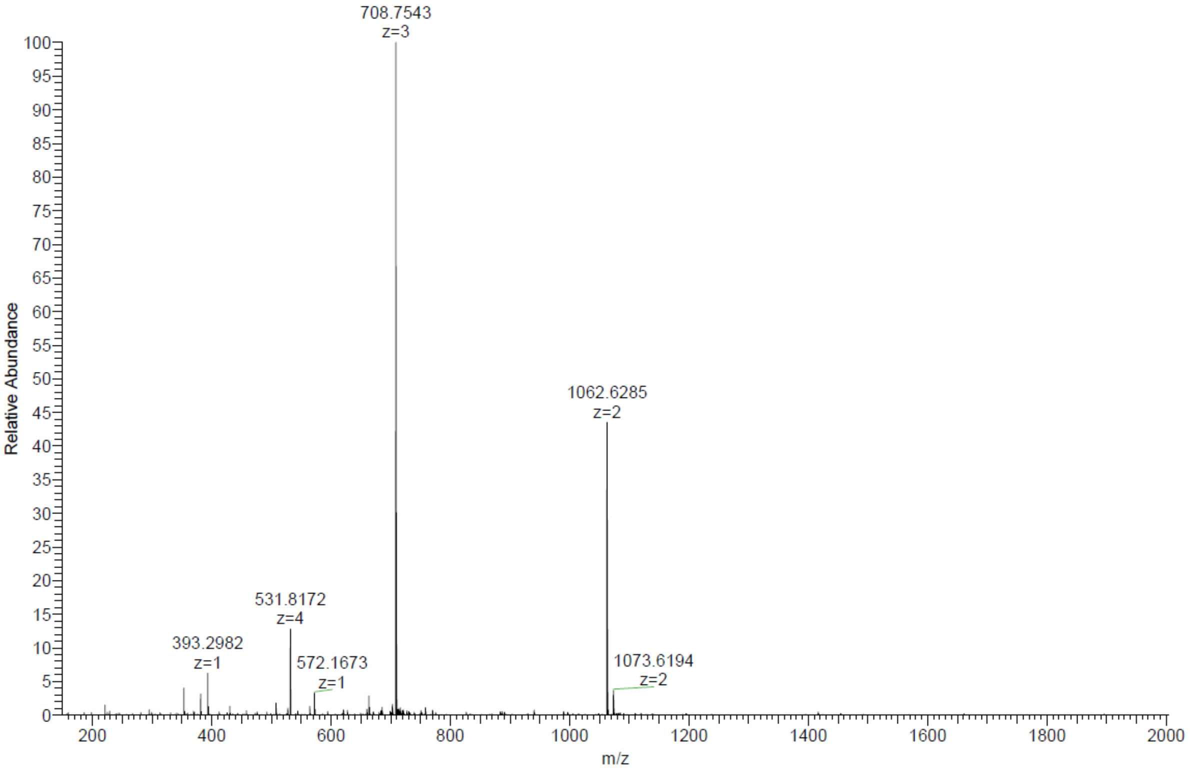
708.7543
z=3
1062.6285
z=2
531.8172
z=4
393.2982
z=1
572.1673
z=1
1073.6194
z=2
Relative Abundance
m/z

FDLL-III was obtained from **DL-III** by procedure mentioned above. Rink Amide AM resin LL (100 mg, 0.29 mmol/g) was used, and the product was obtained as a bright yellow foamy solid after preparative RP-HPLC purification (13 mg,17.2%). Analytical RP-HPLC: t_R = 1.68 min (100% A to 100% D in 3.5 min, λ= 214 nm). MS (ESI+): $C_{107}H_{163}N_{23}O_{22}$ calc./obs. 2122.23/2122.25 $[M]^+$

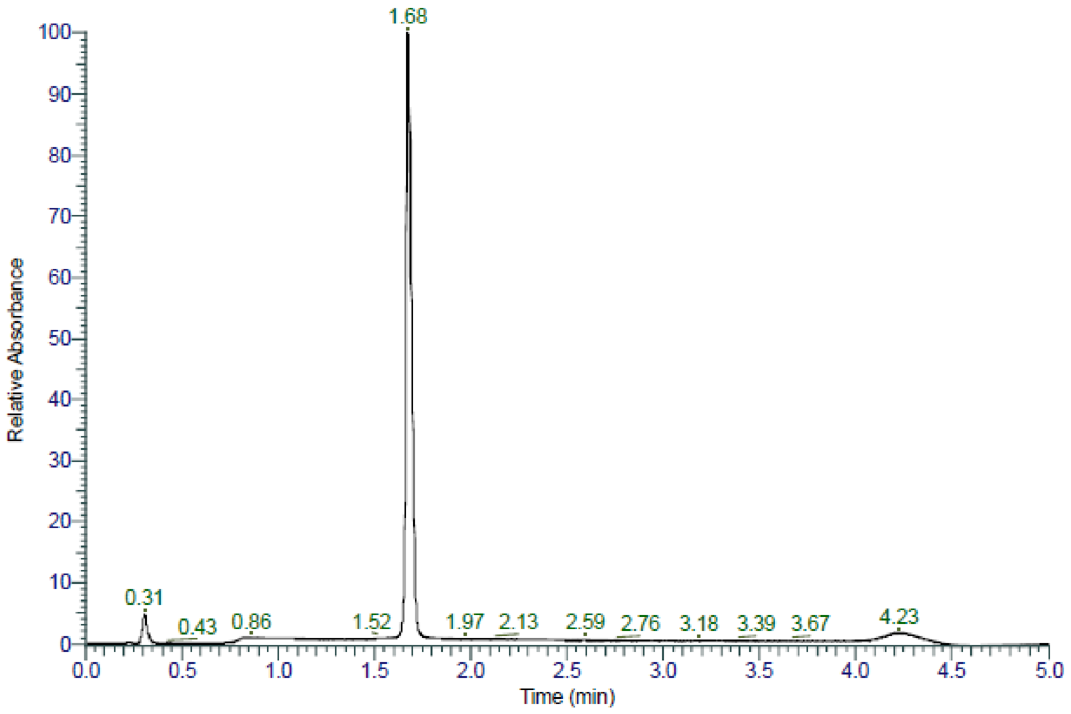

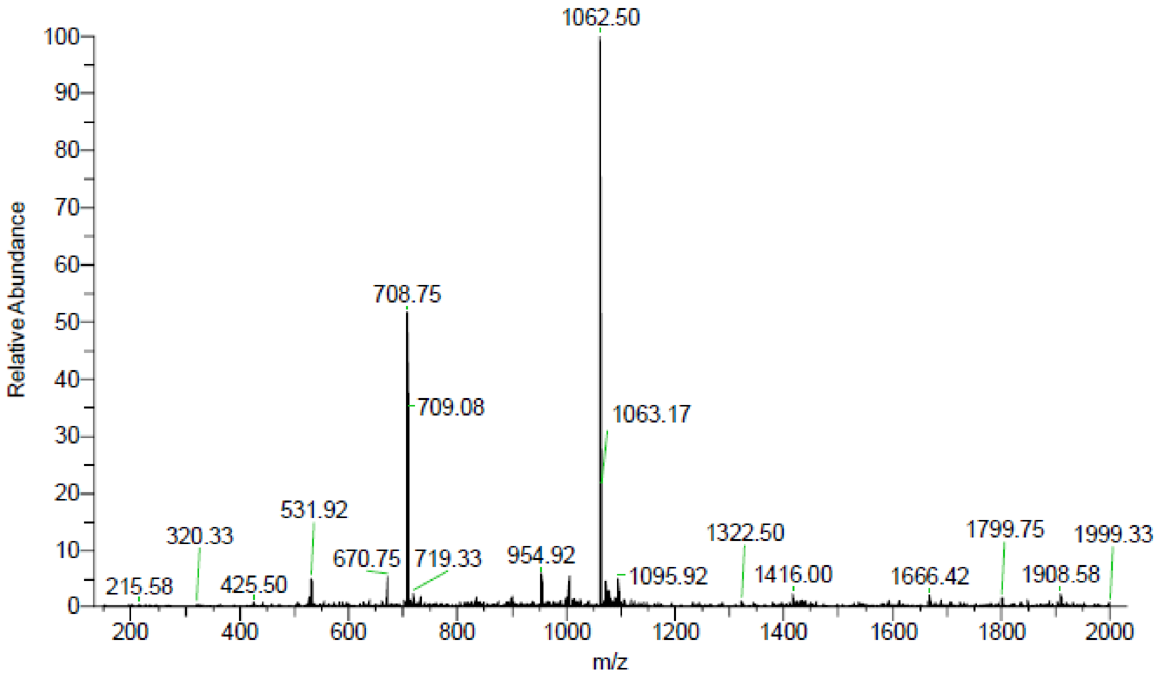

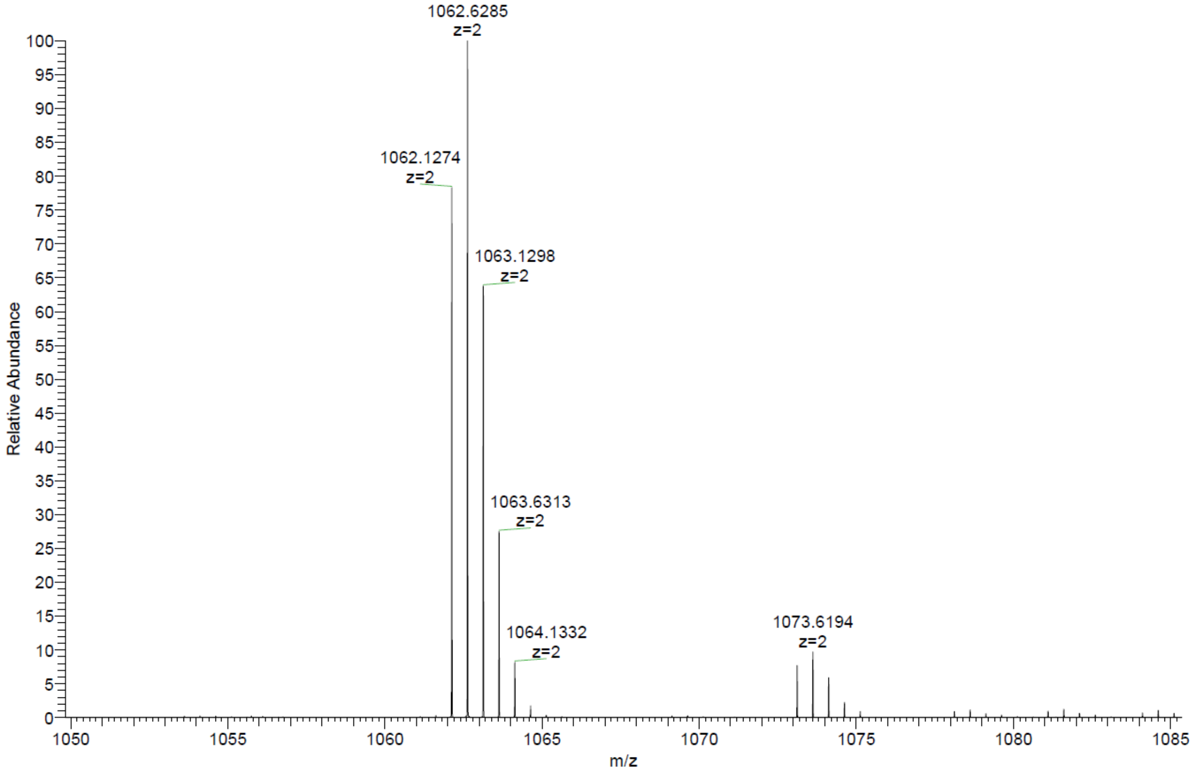

1062.6285
z=2
1062.1274
z=2
1063.1298
z=2
1063.6313
z=2
1064.1332
z=2
1073.6194
z=2
Relative Abundance
m/z

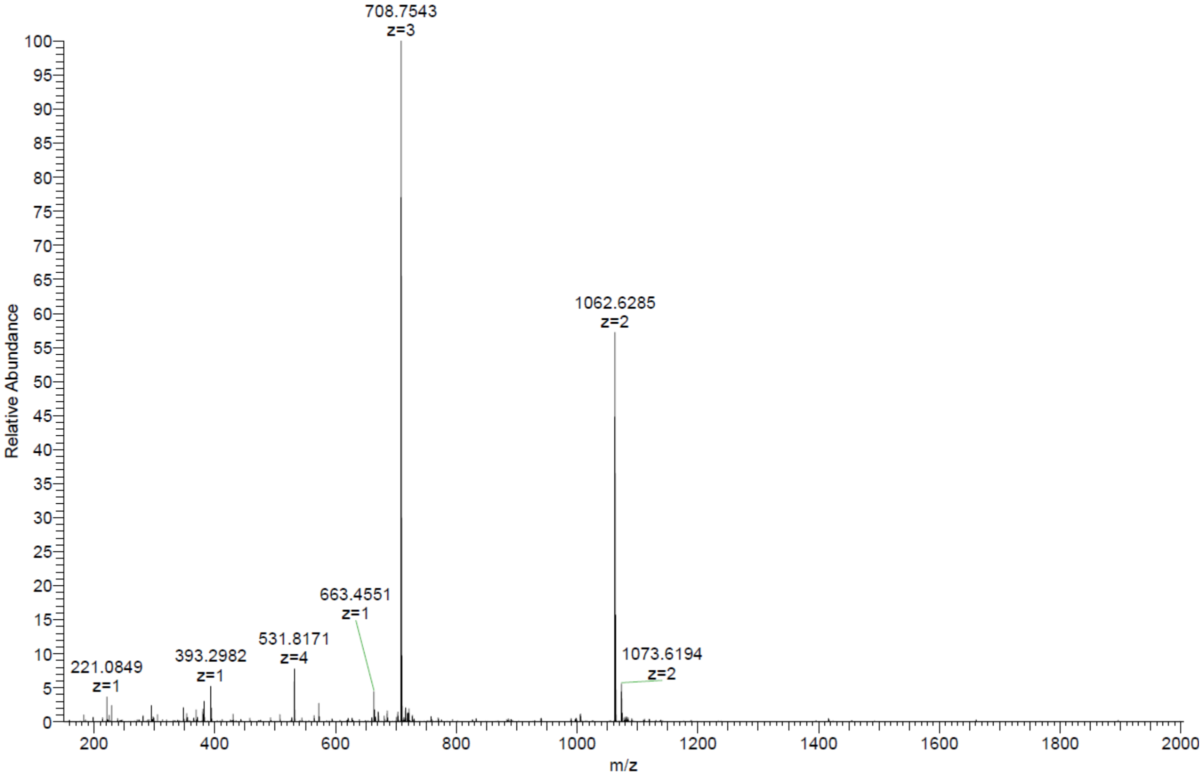

708.7543
z=3
1062.6285
z=2
663.4551
z=1
531.8171
z=4
393.2982
z=1
221.0849
z=1
1073.6194
z=2
Relative Abundance
m/z

FA1 was obtained from **A1** by procedure mentioned above. Rink Amide AM resin LL (100 mg, 0.29 mmol/g) was used, and the product was obtained as a bright yellow foamy solid after preparative RP-HPLC purification (10.3 mg, 16.4%). Analytical RP-HPLC: t_R = 1.60 min (100% A to 100% D in 3.5 min, λ= 214 nm). MS (ESI+): $C_{120}H_{154}N_{22}O_{21}$ calc./obs. 2239.17/2239.16 $[M]^+$

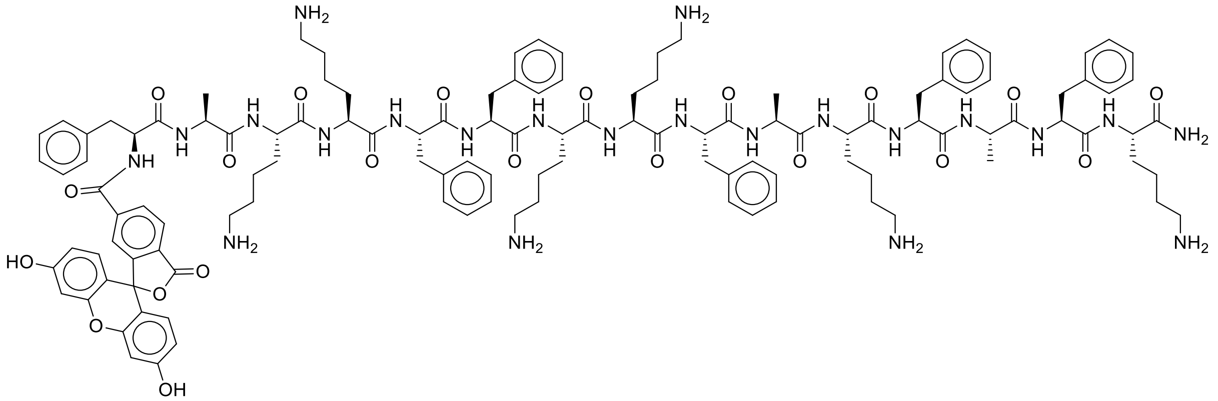

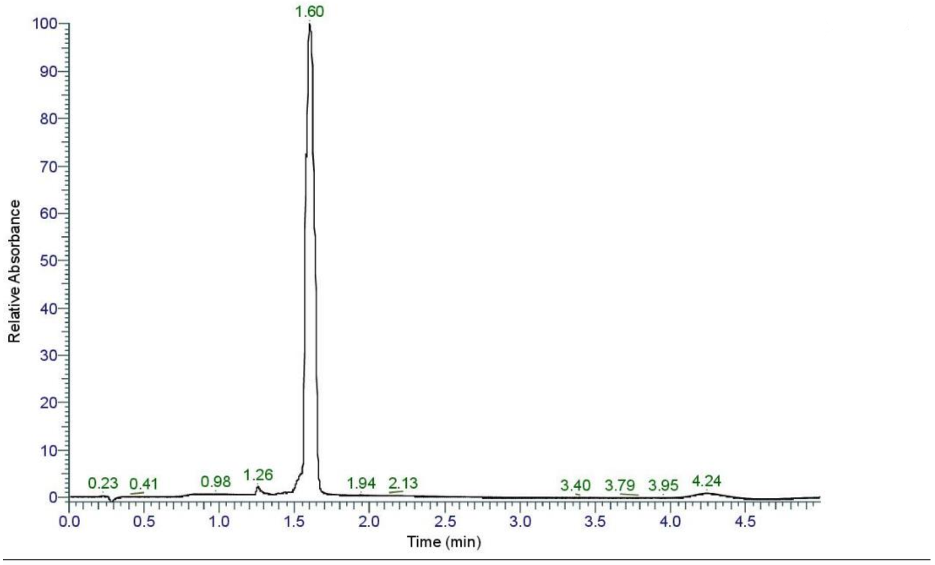

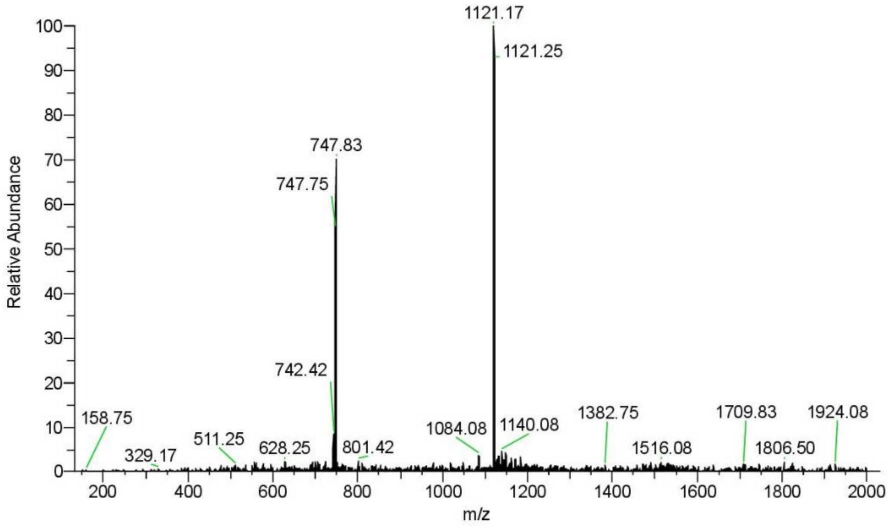

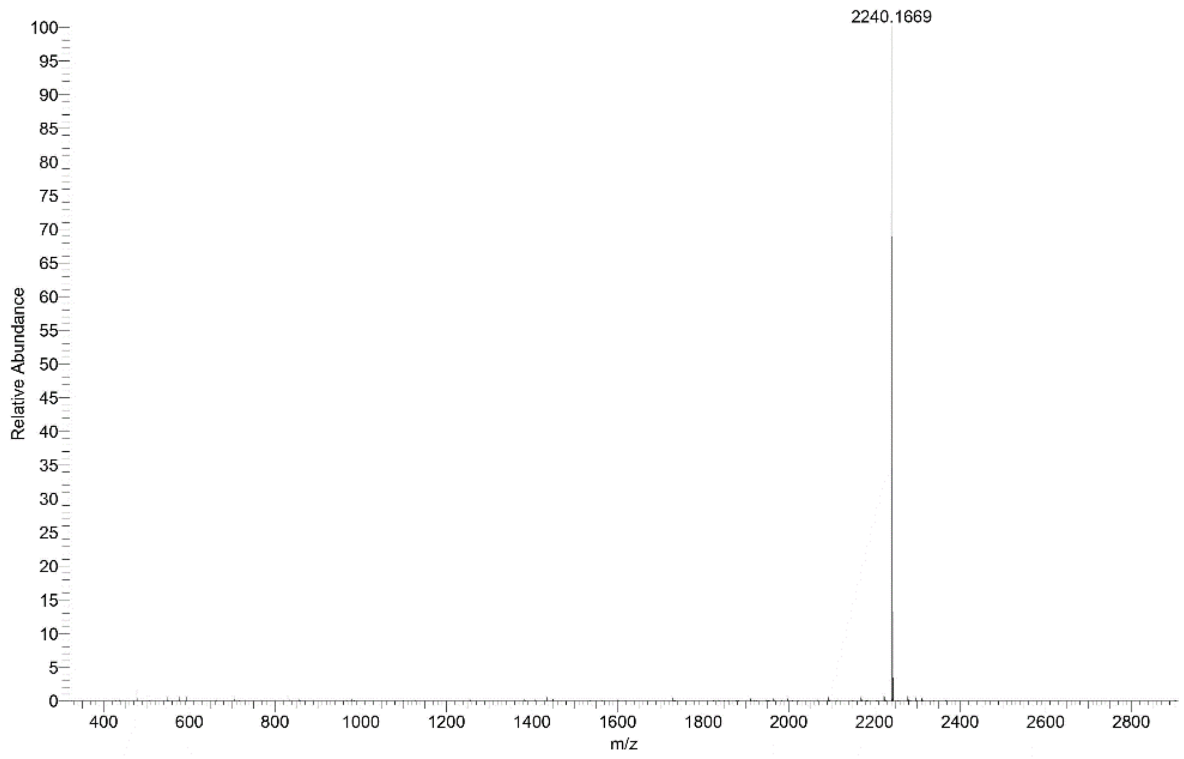
2240.1669
Relative Abundance
m/z

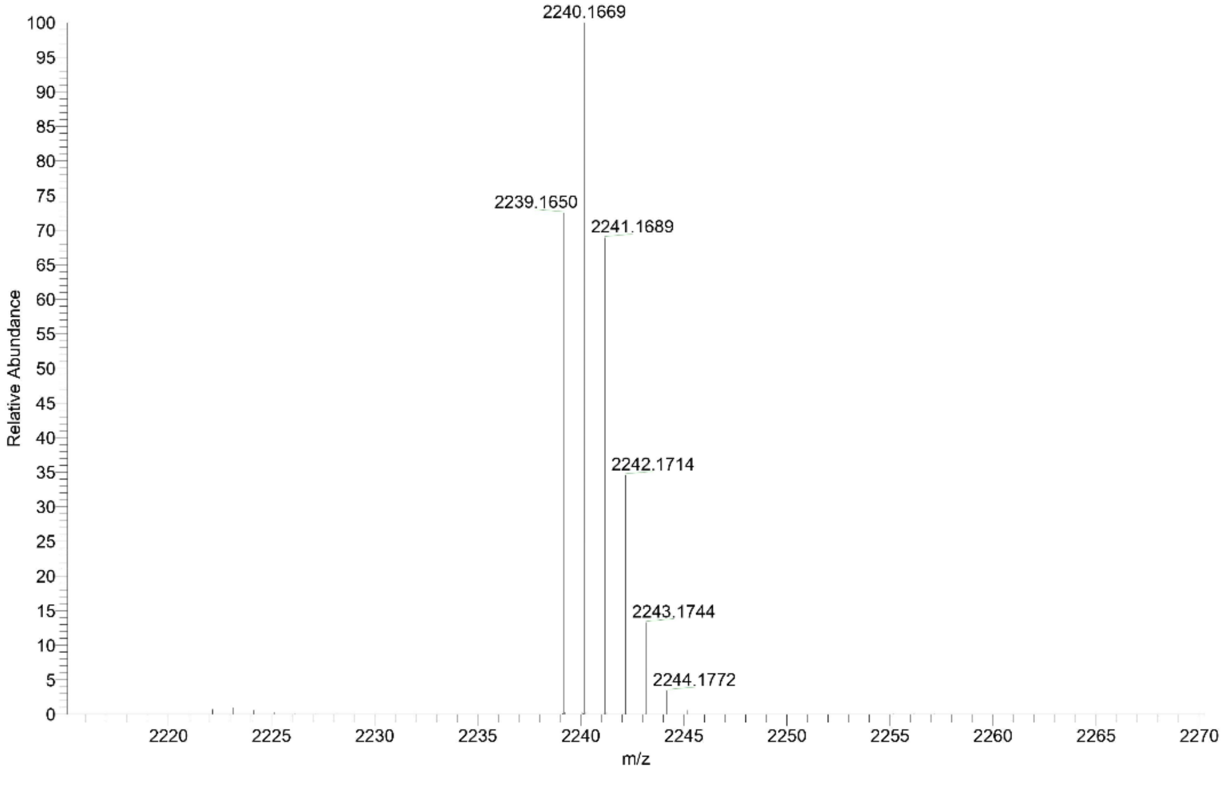
2240.1669
2239.1650
2241.1689
2242.1714
2243.1744
2244.1772
Relative Abundance
m/z

FDA1 was obtained from **DA1** by procedure mentioned above. Rink Amide AM resin LL (100 mg, 0.29 mmol/g) was used, and the product was obtained as a bright yellow foamy solid after preparative RP-HPLC purification (14.1 mg, 17.2%). Analytical RP-HPLC: t_R = 1.61 min (100% A to 100% D in 3.5 min, λ= 214 nm). MS (ESI+): $C_{120}H_{154}N_{22}O_{21}$ calc./obs. 2239.17/2239.16 $[M]^+$

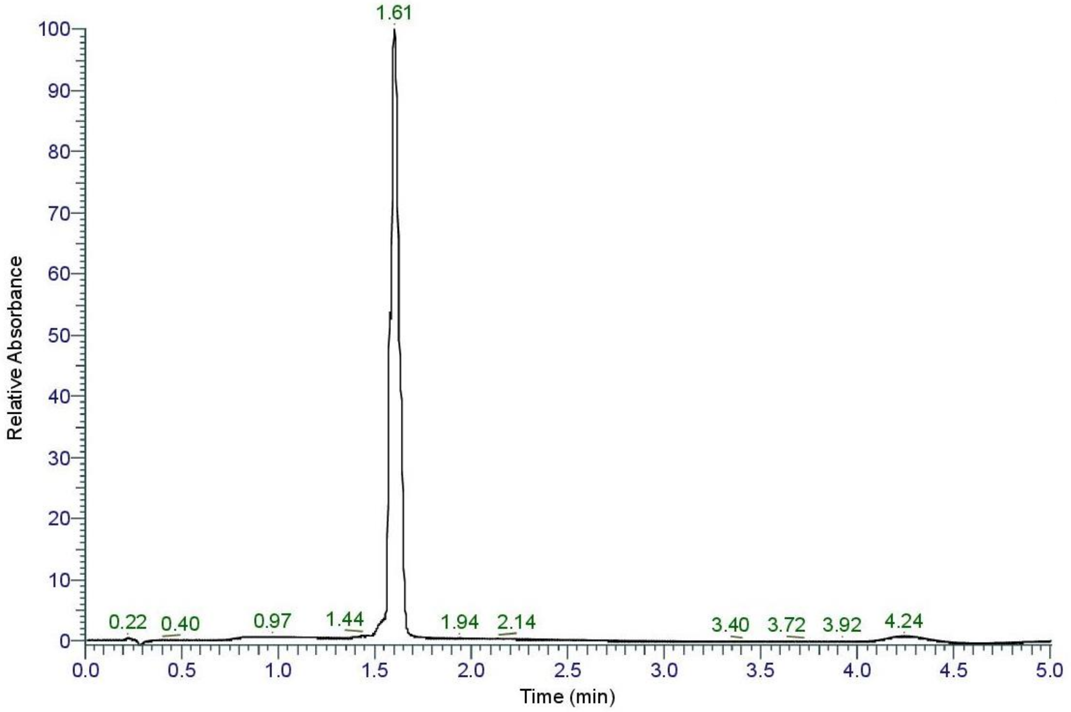

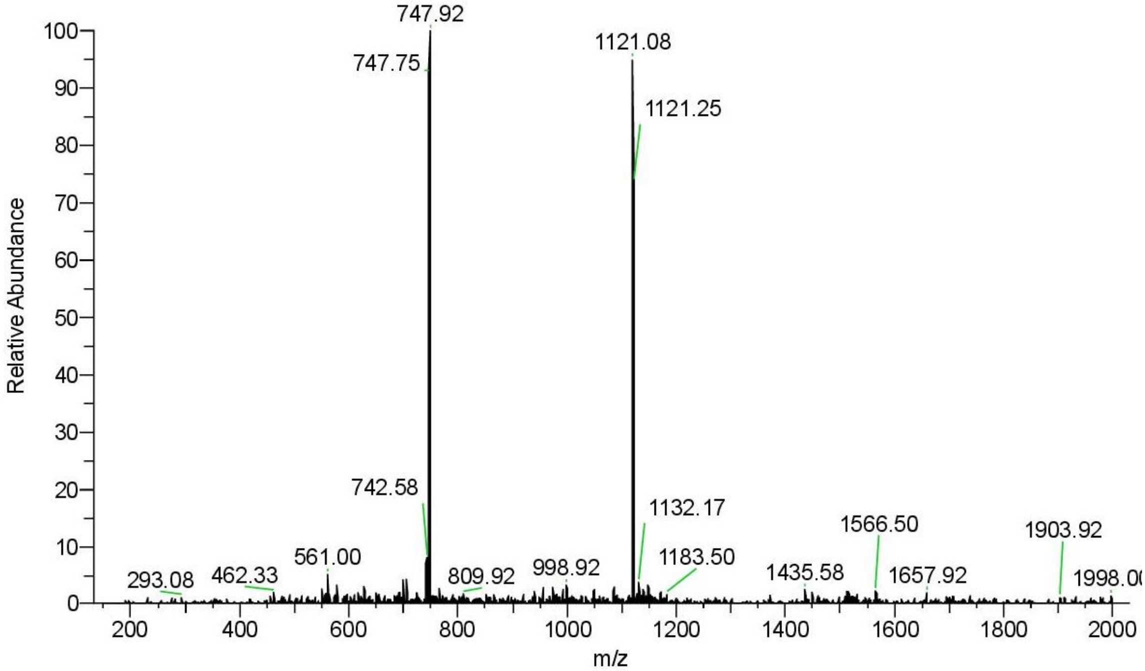

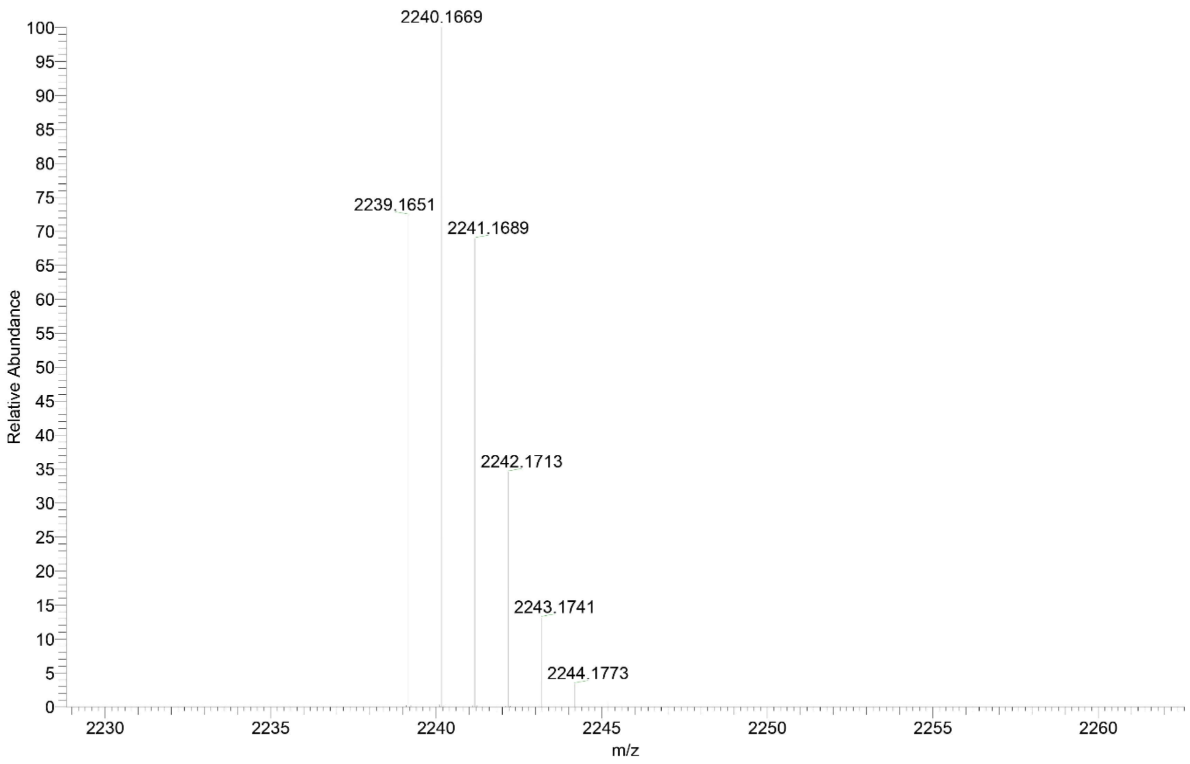
2240.1669
2239.1651
2241.1689
2242.1713
2243.1741
2244.1773
Relative Abundance
m/z

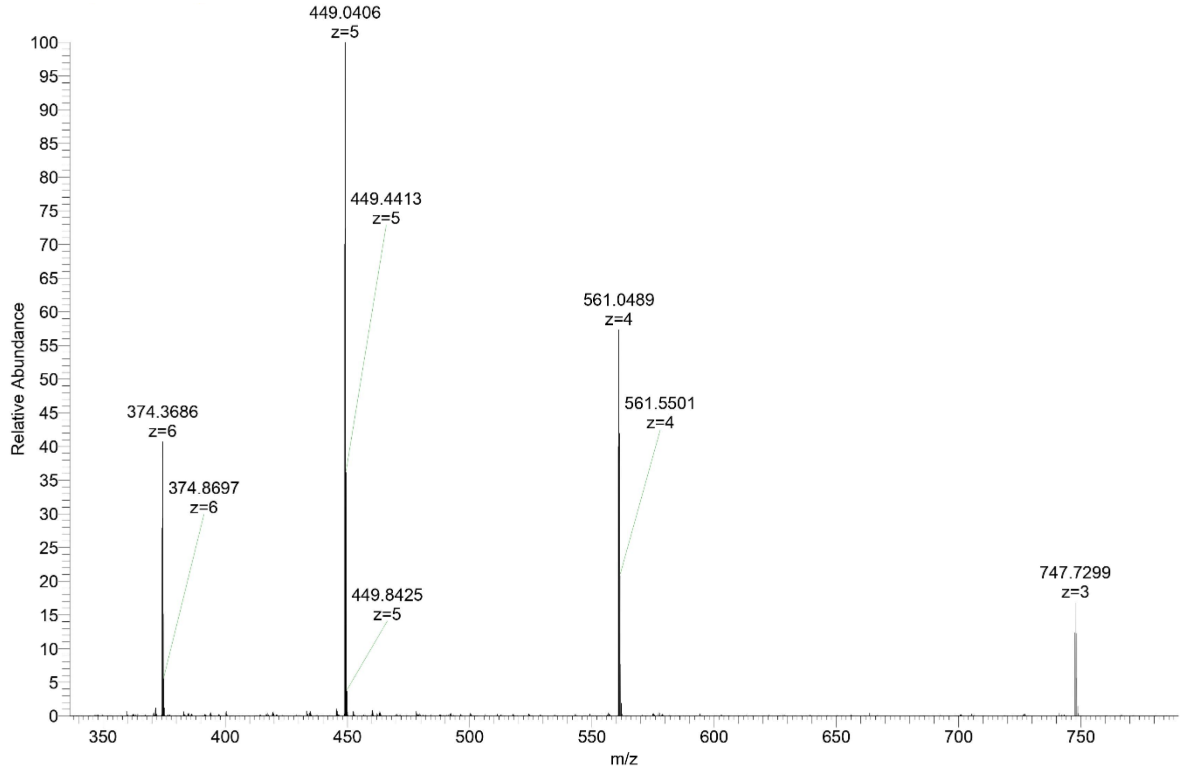
449.0406
z=5
449.4413
z=5
561.0489
z=4
561.5501
z=4
374.3686
z=6
374.8697
z=6
449.8425
z=5
747.7299
z=3
Relative Abundance
m/z

6. References

(1) Heffernan, R.; Paliwal, K.; Lyons, J.; Singh, J.; Yang, Y.; Zhou, Y. Single-Sequence-Based Prediction of Protein Secondary Structures and Solvent Accessibility by Deep Whole-Sequence Learning. *J. Comput. Chem.* **2018**, *39* (26), 2210–2216. https://doi.org/10.1002/jcc.25534.

(2) Eisenberg, D.; Weiss, R. M.; Terwilliger, T. C. The Helical Hydrophobic Moment: A Measure of the Amphiphilicity of a Helix. *Nature* **1982**, *299* (5881), 371–374. https://doi.org/10.1038/299371a0.

(3) Gautier, R.; Douguet, D.; Antonny, B.; Drin, G. HELIQUEST: A Web Server to Screen Sequences with Specific α-Helical Properties. *Bioinformatics* **2008**, *24* (18), 2101–2102. https://doi.org/10.1093/bioinformatics/btn392.
